# An intrinsically disordered antimicrobial peptide dendrimer from stereorandomized virtual screening

**DOI:** 10.1016/j.xcrp.2022.101161

**Published:** 2022-12-21

**Authors:** Xingguang Cai, Markus Orsi, Alice Capecchi, Thilo Köhler, Christian van Delden, Sacha Javor, Jean-Louis Reymond

**Affiliations:** 1 Department of Chemistry, Biochemistry and Pharmaceutical Sciences, University of Bern, Freiestrasse 3, 3012 Bern, Switzerland; 2 Department of Microbiology and Molecular Medicine, University of Geneva, Service of Infectious Diseases, University Hospital of Geneva, Geneva, Switzerland

**Keywords:** antimicrobial peptides, dendrimers, stereorandomization, virtual screening, multidrug resistance, intrinsically disordered proteins, multi-drug resistant bacteria, solid-phase peptide synthesis, membrane disruption

## Abstract

Membrane-disruptive amphiphilic antimicrobial peptides behave as intrinsically disordered proteins by being unordered in water and becoming α-helical in contact with biological membranes. We recently discovered that synthesizing the α-helical antimicrobial peptide dendrimer L-**T25** ((KL)_8_(*K*KL)_4_(*K*LL)_2_*K*KLL) using racemic amino acids to form stereorandomized *sr*-**T25**, an analytically pure mixture of all possible diastereoisomers of L-**T25**, preserved antibacterial activity but abolished hemolysis and cytotoxicity, pointing to an intrinsically disordered antibacterial conformation and an α-helical cytotoxic conformation. In this study, to identify non-toxic intrinsically disordered homochiral antimicrobial peptide dendrimers (AMPDs), we surveyed sixty-three *sr*-analogs of *sr*-**T25** selected by virtual screening. One of the analogs, *sr*-**X18** ((KL)_8_(*K*LK)_4_(*K*LL)_2_*K*LLL), lost antibacterial activity as L-enantiomer and became hemolytic due to α-helical folding. By contrast, the L- and D-enantiomers of *sr*-**X22** ((KL)_8_(KL)_4_(*K*KLL)_2_*K*LKK) were equally antibacterial, non-hemolytic, and non-toxic, implying an intrinsically disordered bioactive conformation. Screening stereorandomized libraries may be generally useful to identify or optimize intrinsically disordered bioactive peptides.

## Highlights

• Stereorandomized antimicrobial peptide dendrimers are identified by virtual screening
• A switch to L residues induces α-helical folding and strong toxicity in one *sr*-AMPD
• Another AMPD remains intrinsically disordered, active, and non-toxic as L-AMPD

## Graphical abstract

**Figure:**
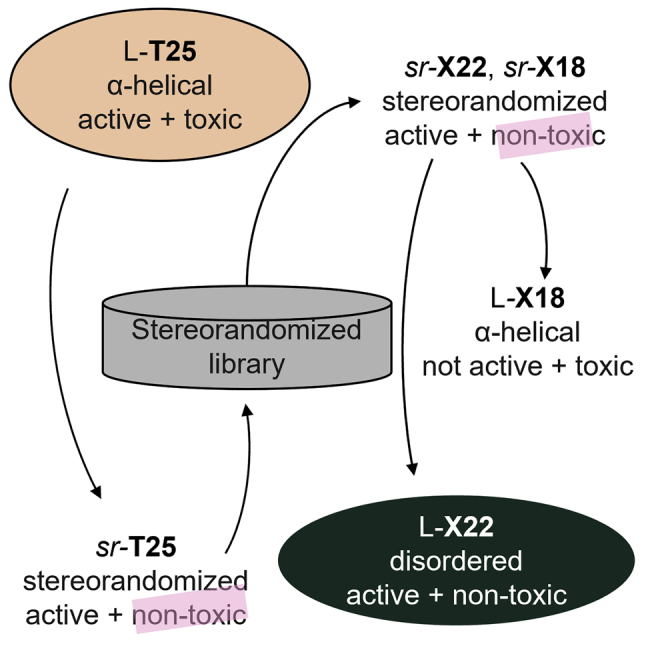

## Teaser

To discover intrinsically disordered bioactive peptides, Cai et al. screen libraries of stereorandomized peptides, each one obtained by synthesis using racemic amino acids to form an analytically pure mixture of all possible stereoisomers. This yields an intrinsically disordered non-toxic antimicrobial peptide dendrimer active against multidrug-resistant bacteria.

## Introduction

Intrinsically disordered proteins (IDPs) are proteins that exist as random coils and whose bioactive conformation can either be unordered or become ordered in presence of their biological target.^1^^,^^2^^,^^3^ Many antimicrobial peptides (AMPs),^4^^,^^5^^,^^6^ which are being investigated as an attractive option to fight multidrug resistant (MDR) bacteria,^7^^,^^8^ formally belong to the second IDP class as they exist as random coils that fold to an amphiphilic α-helix in contact with the bacterial membrane, inducing membrane destabilization or pore formation and eventually killing the bacterium.^9^

**Figure 1:**
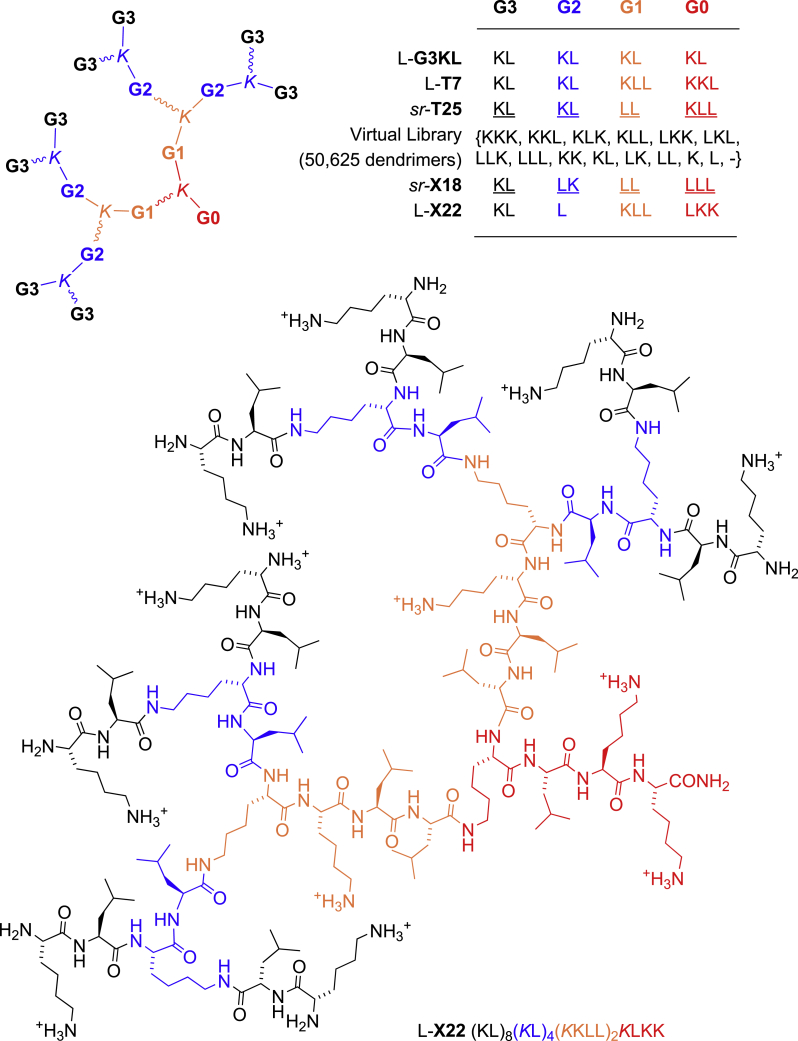
Sequence of AMPDs, dendrimer virtual library, and structural formula of AMPD L-**X22** Racemic residues are underlined. Branching lysines are L-chirality in L-sequences and racemic in *sr* sequences.

In our own efforts to develop antibacterial agents,^10^^,^^11^^,^^12^^,^^13^ we recently discovered that AMP dendrimers (AMPDs; e.g. L-**G3KL**; Figure 1)^14^^,^^15^ are potent antibacterial agents acting by a membrane disruptive mechanism similar to AMPs,^16^ as do various cationic amphiphiles such as cyclic peptides,^17^ polymers,^18^ peptidomimetics,^19^ foldamers,^20^ and dendrimers.^21^^,^^22^^,^^23^^,^^24^ L-**G3KL** kills Gram-negative MDR bacteria, including polymyxin-resistant clinical isolates in a pH- and ionic-strength-dependent manner without hemolysis of human red blood cells,^25^^,^^26^^,^^27^ and shows antibiofilm^28^ and wound-healing properties^29^ and partial synergy with classical antibiotics,^30^ as well as retention of activity as chitosan conjugate.^31^

Circular dichroism (CD) and molecular dynamics (MD) studies with L-**G3KL** and analogs L-**T7** and L-**T25** showed that, similar to linear α-helical AMPs, these dendrimers fold into an α-helical conformation in contact with the membrane.^32^ However, their antibacterial activity was unaffected by stereorandomization, which consists of synthesizing the dendrimers using racemic amino acids to yield an analytically pure mixture of all possible diastereoisomers, suggesting that the bioactive antibacterial conformation of all L- or all D-AMPDs might not be α-helical but intrinsically disordered and therefore belong to the first class of IDPs.^33^

Here, we set out to identify an intrinsically disordered AMPD by screening a library of stereorandomized dendrimers to discover active stereorandomized (*sr*)-AMPDs and later testing their activity and conformational behavior in homochiral form. Starting with *sr*-**T25**, we composed a focused library of analogs (*sr*-**X1** – *sr*-**X63**; Figure S1) by ligand-based virtual screening (LBVS)^34^^,^^35^ of a virtual library of 50,625 G3 peptide dendrimers featuring all possible permutations of up to three lysines or leucines in each generation.^32^ We computed similarities to *sr*-**T25** using macromolecule-extended atom-pair fingerprint (MXFP), a molecular fingerprint counting atom pairs at increasing topological distances measured in bonds along the shortest path.^36^ Atom-pair fingerprints encode molecular shape and pharmacophores^36^^,^^37^^,^^38^^,^^39^^,^^40^ and were used previously to identify antimicrobial bicyclic peptides^41^^,^^42^ and AMPDs.^32^ As for most molecular fingerprints used in LBVS, MXFP is calculated from the two-dimensional (2D) structure without stereochemistry and is therefore suitable for stereorandomized sequences.

In summary, 63 *sr-*AMPD analogs have been synthesized and tested and many of them are readily active and non-hemolytic, which leads to the identification of *sr*-**X18** and *sr*-**X22** as two particularly potent AMPDs. Structure-activity relationship, mechanistic, and modeling studies show that one of them, *sr*-**X22**, retains its high activity, low hemolysis, and low cell toxicity in the form of its pure L- or D-enantiomers, implying that its bioactive conformation is intrinsically disordered.

## Results and discussion

### Virtual screening, synthesis, and testing reveal potent non-hemolytic *sr*-AMPDs

**Figure 2:**
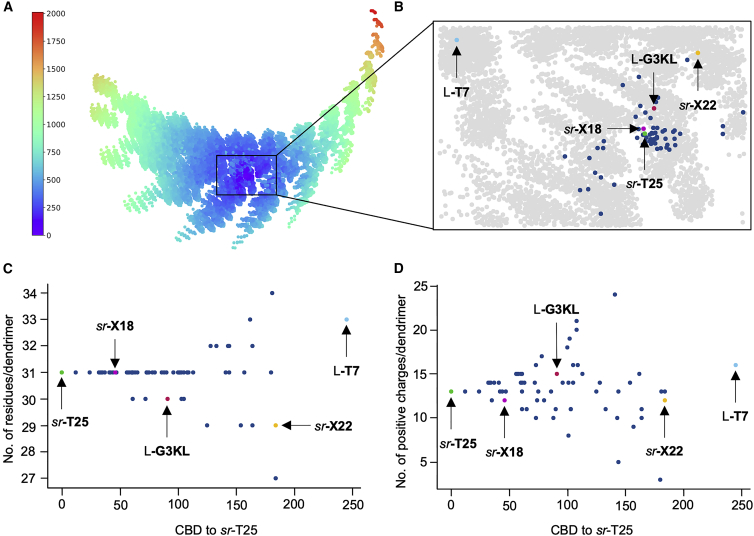
Selecting a stereorandomized-focused library by virtual screening (A) Principal-component analysis of the virtual library by MXFP values colored by similarity to *sr-***T25** as calculated using the city-block distance (CBD). See also https://tm.gdb.tools/map4/dendrimers_mxfp_pca/. (B) Close-up view around *sr-***T25**. (C and D) Size distribution (C) and positive charges of dendrimers (D) in the focused stereorandomized library as function of CBD to *sr-***T25.**

To compose a focused library of *sr*-**T25** analogs, we sorted our virtual dendrimer library using the MXFP pharmacophore fingerprint.^36^ We selected the 20 MXFP-nearest neighbors of *sr*-**T25** and 43 additional sequences by clustering among the first 200 and 1,000 closest sequences (see experimental procedures for details). This selection sampled 2% of the virtual library covering a narrow range of the chemical space surrounding *sr*-**T25**, as illustrated by a principal-component analysis of the MXFP property space (Figures 2A and 2B). The selected dendrimers differed from *sr*-**T25** in size (36–41 residues; Figure 2C) and overall positive charges (+3 to +24; Figure 2D), with increasingly different values as the similarity to *sr*-**T25** decreased, as measured by an increase in the city-block distance (CBD)^43^ calculated from the MXFP values. These positive charges are contributed by the ε-amino groups of the free lysine side chains (p*K*_a_ > 9) because the eight N termini of the peptide dendrimers have a lowered p*K*_a_ of approximately 6.5 and occur as neutral amino groups at neutral pH (Figure S2).^27^

We synthesized the stereorandomized library of 63 *sr*-**T25** analogs by high-temperature solid-phase peptide synthesis (SPPS) using racemic amino acid building blocks (*sr*-**X1** – *sr*-**X63**; Table S1). All products were obtained as homogeneous products after preparative high-performance liquid chromatography (HPLC) purification. Most dendrimers were non-hemolytic yet showed very substantial activity against the Gram-negative bacterium *Pseudomonas aeruginosa* and, to a lesser extent, against *Escherichia coli*, *Acinetobacter baumannii*, and *Klebsiella pneumoniae*, in line with the activity profile of the parent AMPD *sr*-**T25**.^33^

The ratio of lysine to leucine side chains, which determines the ratio between positive charges and hydrophobic groups, was a key determinant of activity. From the 26 most active and least hemolytic dendrimers found at intermediate lysine (Lys)/leucine (Leu) ratio values (*sr*-**X7** – *sr*-**X32**, Lys/Leu = 0.45–0.81; Figure S1), we selected two dendrimers for closer study due to their particularly good activity and interesting amino acid sequence. The first one was *sr*-**X18**, one of the most active close analogs of *sr*-**T25** (CBD = 22) with a hydrophobic dendrimer core composed of only Leu residues in G1 and G0 and an inverted sequence in the G2 branch (KL → LK) compared with most AMPDs. The second one was *sr*-**X22**, one of the most distant yet very active analogs of *sr*-**T25** (CBD = 144), in particular showing good activity against *K. pneumoniae*. Compared with *sr*-**T25**, *sr*-**X22** featured a shorter and less cationic G2 branch (KL → L) but a more cationic G1-G0 core ((LL)_2_*K*KLL → (KLL)_2_*K*LKK).

### *sr*-, L-, and D-**X22** combine strong antibacterial activity with low toxicity

To gain a broader insight into the effect of stereorandomization on AMPD activity, we prepared all L and all D versions of *sr*-**T25**, *sr-***X18**, and *sr-***X22**, as well as analogs *sr*-**aT25**, *sr-,* L-, and D-**aX18**, and *sr*-, L-, and D-**aX22**, where N termini, which have an apparent p*K*_a_ ∼ 6.5 (Figure S2), have been removed, an effect that may increase AMPD activity.^27^ We measured MICs (minimum inhibitory concentrations) of all compounds at a physiological pH of 7.4, as well as at pH 5, which occurs in skin infections, and at pH 8.0, relevant to the slightly alkaline conditions in chronic wounds.^44^^,^^45^ We considered four Gram-negative bacteria and additionally included methicillin-resistant *Staphylococcus aureus* (MRSA) since our AMPDs as well as polymyxin become active against this Gram-positive bacterium at pH 8.0 (Table 1).^27^ In these assays, the parent AMPD *sr*-**T25**, which is non-hemolytic compared with the pure enantiomers L-**T25** or D-**T25**,^33^ was equally active against bacteria at all three pH values measured and was even more active than the pure enantiomers against MRSA at pH 8.0. Analog *sr*-**aT25** with N termini removed was slightly more antibacterial than *sr*-**T25** while retaining a low hemolysis.

**Table 1:** Antibacterial activity and cytotoxicity of AMPD *sr*-**X22**, *sr*-**X18**, and analogs

	*E. coli* W3110	MIC at pH 5.0/7.4/8.0^a^				MHC^b^	IC_50_ HEK293^c^
		*A. baumannii* ATCC 19606	*P. aeruginosa* PAO1	*K. pneumoniae* NCTC 418	*S aureus* COL		
*sr*-**T25**	8/4/2	8/8/2	8/8/2	>64/64/8	>64/>64/4	1,000	197 ± 27
L-**T25**	32/8/8	16/4/4	32/8/8	>64/32/16	>64/>64/32	62.5	92 ± 3
D-**T25**	32/8/4	32/8/4	32/8/8	>64/16/16	>64/>64/32	125	33 ± 3
*sr-***aT25**	2/2–4/2	2/2/2	8/8/2	>64/32/4	>64/>64/2	1,000	5.8 ± 0.3
*sr-***X18**	32/4/2	2/2/2	16/4/2	>64/32/8	>64/>64/2	>2,000	117 ± 12
L-**X18**	16/16/16	16/32/16	16/32/16	>64/>64/32	>64/>64/16	7.8	52 ± 4
D-**X18**	32/64/32	32/64/32	32/64/32	>64/>64/>64	>64/>64/32	7.8	47 ± 12
*sr-***aX18**	4/4/2	2/2/2	16/8/4	>64/16/8	>64/16/2	>2,000	18 ± 1
L- **aX18**	32/>64/32	32/64/32	64/>64/64	>64/>64/>64	>64/>64/32	<3.9	14 ± 0.6
D- **aX18**	32/>64/32	32/>64/16	64/>64/64	>64/>64/>64	>64/>64/16	<3.9	21 ± 2
*sr*-**X22**	32/4/2	32/4/2	16/2/2	>64/64/8	>64/>64/16	>2,000	575 ± 30
L-**X22**	8/4/2	8/4/2	8/4/4	>64/>64/8	>64/>64/8	>2,000	575 ± 23
D-**X22**	8/4/4	16/8/4	16/4/4	>64/>64/16	>64/>64/4	2,000	514 ± 21
*sr-***aX22**	2/2/2	2/2/2	64/8/4	>64/64/8	64/16/2	>2,000	21 ± 3
L- **aX22**	2/4/4	2/4/4	16/8/16	64/8/8	64/16/4	>2,000	24 ± 3
D- **aX22**	4/8/4	4/4/4	32/8/16	64/16/16	>64/16/4	>2,000	17 ± 2
L-**G3KL**	32/8/1–2	8/8/1	16/4/1	>64/>64/4	>64/>64/2	>2,000	460 ± 17
L-**T7**	16/4/2	16/8/2–4	16/8/2–4	>64/32/8	>64/>64/4	>2,000	70 ± 6
**PMB**	0.02/0.25/0.13	1/0.25/0.25	0.03/0.5/0.5	8/0.25/0.25	>64/>64/4	>2,000	346 ± 20

a MIC in μg/mL was measured in Müller-Hinton (MH) medium after incubation for 16–20 h at 37°C. Each result represents two independent experiments performed in duplicate.

b MHC (minimum hemolytic concentration) in μg/mL was measured on human red blood cells in PBS (pH 7.4) at room temperature after incubation for 4 h at 37°C. Each result represents two independent experiments performed in duplicate.

c IC_50_ (mean ± SD, μg/mL) was measured on HEK293 cell line after incubation for 48 h at 37°C. Data present results from three independent experiments in triplicates.

The non-hemolytic *sr*-**X18** revealed a surprising effect because its pure enantiomers L-**X18** and D-**X18** were strongly hemolytic and twice as cytotoxic but showed much weaker antibacterial activities, implying that this sequence would not have been selected in a screen with pure enantiomers. This switch from an antibacterial non-hemolytic *sr* form to an inactive but strongly hemolytic L or D form occurred even more strongly with **aX18** lacking N termini. In contrast to *sr*-**X18**, the antibacterial activity of *sr*-**X22** remained constant or even slightly increased in the pure enantiomers L-**X22** or D-**X22**, while hemolysis remained absent and cytotoxicity at a comparably low level. This activity pattern was also preserved upon removal of N termini to form *sr*-, L-, or D-**aX22**.

We also measured AMPD activity against HEK293 cells as an indication of toxicity, which, among previous AMPDs, was problematic for L-**T7** and L- and D-**T25** compared with L-**G3KL**. While toxicity was also quite high for dendrimers lacking N termini (IC_50_ = 5.8–24 μg/mL) and for L-, D-, and *sr*-**X18** (IC_50_ = 47–117 μg/mL), L-, D-, and *sr-***X22** showed even lower toxicity (IC_50_ > 500 μg/mL) than L-**G3KL** and polymyxin B (**PMB**).

We performed additional profiling against MDR bacteria to compare the best AMPDs *sr*-**X18** and L-, D-, and *sr*-**X22** with our previous AMPDs *sr*-**T25**, L-**G3KL**, and L-**T7** (Table 2). The dendrimers were all similarly active against *Stenotrophomonas maltophilia* (MIC = 4–8 μg/mL), *P. aeruginosa* PA14, and MDR clinical isolate ZEM-1A (MIC = 4–16 μg/mL), and all AMPDs except *sr*-**X22** were active against *Enterobacter cloacae* (MIC = 8–32 μg/mL).

**Table 2:** Activity of selected AMPDs against MDR bacteria

	*S. maltophilia*^a^	*P. aeruginosa* PA14	*P. aeruginosa Z*EM-1A	*E. cloacae*	*K. pneumoniae* OXA-48	*P. aeruginosa* ZEM9A	*B. cenocepacia*	*S. aureus* Newman
*sr-***X18**	4	8	8	16	16	64	>64	>64
*sr-***X22**	4	4	4	>64	32	>64	>64	>64
L-**X22**	4	4	4	4	8	32	>64	>64
D-**X22**	4	4	4	8	4	8	>64	64
*sr-***T25**	8	4	4	8	>64	16	>64	>64
L-**G3KL**	8	2–4	4	8	>64	16	>64	>64
L-**T7**	4	8	4	8	16	8	>64	>64
**PMB**	4	0.5	0.5	0.5	1	64	>64	>64

a MIC in μg/mL was measured in MH medium at pH 7.4 after incubation for 16–20 h at 37°C. Each result represents two independent experiments performed in duplicate.

Furthermore, all except *sr*-**T25** and L-**G3KL** were active against the carbapenem-resistant *K. pneumoniae* strain OXA-48 (MIC = 4–32 μg/mL), with D-**X22** standing out as the most active AMPD against this bacterium. Like the positive control **PMB**, none of these dendrimers showed significant activity against the clinical isolate *P. aeruginosa* ZEM9A, against *Burkholderia cenocepacia*, or against *S. aureus* Newman. Taken together, these data showed that *sr*-, L-, and D-**X22** combined the best overall antibacterial effects with low toxicity compared with our previously best AMPDs L-**G3KL**, L-**T7**, and *sr*-**T25**. In terms of stability in human serum, degradation was acceptable for L-**X22** (70% remaining at 24 h), very low for D-**X22** (90% remaining at 24 h), and undetectable for *sr*-**X22** (Figure S3).

### AMPDs rapidly kill bacteria by membrane disruption

**Figure 3:**
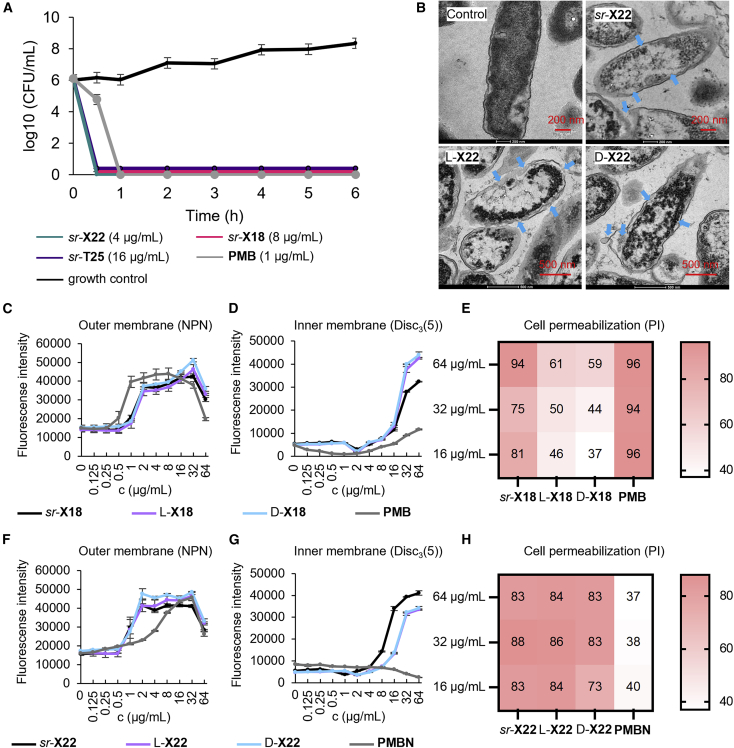
AMPDs rapidly kill bacteria by membrane disruption (A) Bacteria-killing assay against *P. aeruginosa* PAO1 at 2× MIC. Data are presented in mean ± SD, n = 3. Experiments were performed ≥2 times in triplicates. (B) TEM images of PAO1 (OD_600_ = 1) 2 h after treatment at 10× MIC in Müller-Hinton (MH) medium at pH 7.4. Scale bar is 200 nm. Blue arrows indicate disrupted membrane. (C and F) NPN outer membrane permeability assay of PAO1 treated with **X18** (C) and **X22** (F) in the presence of 10 μM NPN. Fluorescent intensity (*λ*_exc_ = 340 nm, *λ*_em_ = 415 nm) was measured within 5 min after the treatment. Data are presented in mean ± SD, n = 3. (D and G) DiSC_3_(5) inner membrane depolarization assay of PAO1 treated with **X18** (D) and **X22** (G) in the presence of 2 μM DiSC_3_(5). Fluorescence intensity (*λ*_exc_ = 610 nm, *λ*_em_ = 660 nm) was measured within 5 min after treatment. Data are presented in mean ± SD, n = 3. (E and H) PI cell permeabilization assay with of PAO1 treated with **X18** (E) and **X22** (H). The percentage of PI-positive cells is indicated after incubating PAO1 (OD_600_ = 1) for 20 min.

Time-kill experiments with *P. aeruginosa* confirmed that AMPDs *sr*-**X18** and L-, D-, and *sr*-**X22** rapidly killed bacteria, like the parent AMPD *sr*-**T25** (Figures 3A and S4). Transmission electron microscopy (TEM) images showed damage to the bacterial membrane and partial emptying of cell contents (Figures 3B and S5–S12). To our surprise, both active (L-, D-, and *sr*-**X22**, *sr*-**X18,** L-, D-, and *sr*-**T25**) and inactive (L- and D-**X18**) dendrimers permeabilized the outer membrane of *P. aeruginosa* cells as measured by fluorescence assay with *N*-phenylnaphthylamine (NPN), a small molecule that cannot effectively cross the outer membrane but has strong fluorescence when binding to phospholipids (Figures 3C, 3F, and S13).^46^ The same AMPDs also depolarized the inner membrane as measured by fluorescence assay with the membrane potential sensitive dye DiSC_3_(5) (Figures 3D, 3G, and S14).^47^ However, only the active AMPDs fully permeabilized *P. aeruginosa* cells as measured by the uptake of propidium iodide (PI) using flow cytometry. PI is a membrane non-permeable compound that shows strong fluorescence when binding to DNA.^30^^,^^48^ By contrast, the inactive L-**X18** and D-**X18** had much lower permeabilizing effects (Figures 3E, 3H, and S15–S30).

By comparison, the control antibiotic **PMB**, which binds to lipid A of the outer bacterial membrane,^49^^,^^50^ permeabilized the outer membrane but did not depolarize the inner membrane. Nevertheless, **PMB** induced a strong uptake of PI. On the other hand, inactive **PMB** nonapeptide (**PMBN**) permeabilized the outer membrane,^51^^,^^52^ but had no effect on inner membranes, and showed no PI uptake (Figures 3F–3H).

Taken together, these experiments indicated that our AMPDs killed bacteria by disruption of the outer and inner membranes leading to permeabilization and partial emptying of the cell content. Most strikingly, the effects of *sr*-, L-, and D-**X22** were indistinguishable, while in the case of **X18**, stereochemical purity controlled the overall permeabilization effect as measured by PI uptake, which only occurred with *sr*-**X18**.

### Antibacterial dendrimer L-**X22** and hemolytic dendrimer L-**X18** show different folding and membrane interactions

**Figure 4:**
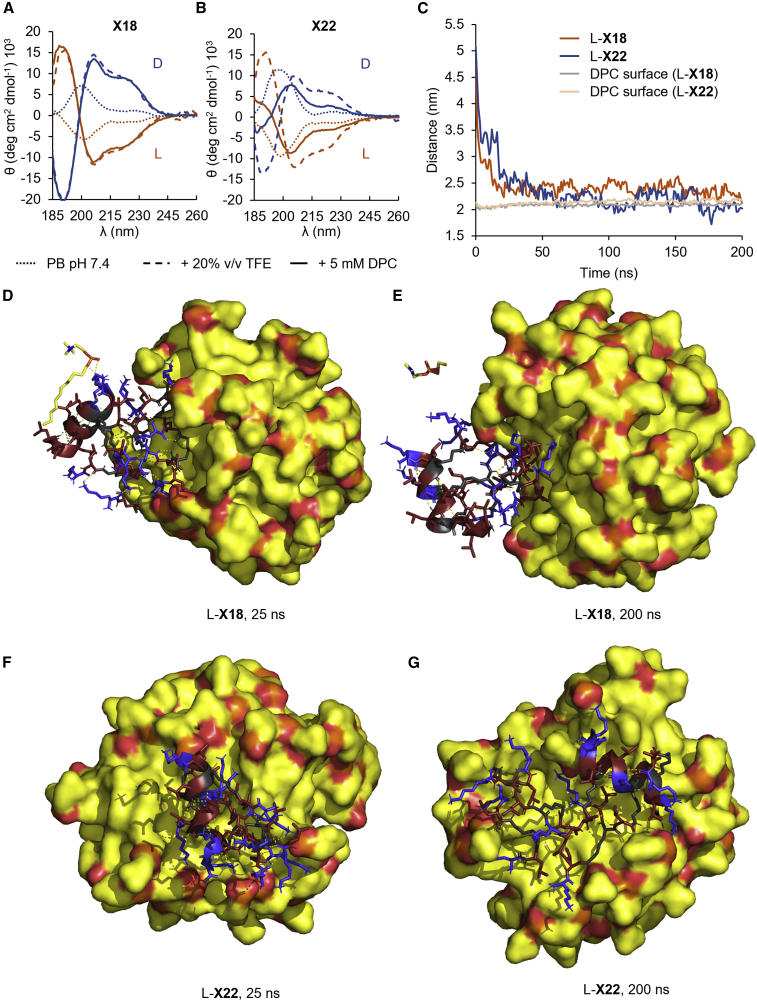
Antibacterial dendrimer L-**X22** and hemolytic dendrimer L-**X18** show different folding and membrane interactions (A and B) CD spectra of L-/D-**X18** (A) and L-/D-**X22** (B) at 0.100 mg/mL in 8 mM phosphate (pH 7.4) with 20% TFE or 5 mM DPC. (C) Time course of the distance from center of mass of dendrimers to center of mass of DPC micelle, and average radius of DPC micelle. (D–G) MD simulation of L-**X18** and L-**X22** with a DPC micelle after 25 and 200 ns at 300 K. Initially, the dendrimers were placed at ∼3 nm of the preformed micelle surface and reached the micelle by passive diffusion.

To better understand the difference between the non-hemolytic, antibacterial L-**X22** and the hemolytic, non-antibacterial L-**X18**, we investigated their conformational behavior. CD spectra of L-**X18** (and its enantiomer D-**X18**) showed a transition from an unordered conformation in water to a more α-helical conformation in the presence of 20% v/v trifluoroethanol (TFE) as folding inducer or 5 mM *n-*dodecylphosphocholine (DPC) as a micelle-forming additive mimicking the membrane environment (Figures 4A, 4B, and S31).^13^^,^^53^^,^^54^ L-**X22** (and its D-enantiomer) was also disordered in water and partially α-helical with TFE. However, its CD trace in the presence of DPC has a shape intermediate between the unordered trace in water and the α-helical trace with TFE, indicating a less extensive α-helical folding with DPC micelles (Figure 4B). These data showed that the antibacterial, non-hemolytic L-**X22** was less prone to α-helical folding in a membrane environment than the hemolytic and non-antibacterial L-**X18**. We observed similar effects in the CD spectra of analogs L-/D-**aX18** and L-/D-**aX22** lacking the N termini (Figure S32).

We next performed MD using GROMACS^55^ to gain an insight into how the α-helical conformation of our AMPDs might look. We performed MD simulations starting with α-helically prefolded dendrimer models of L-**X18** and L-**X22** in either water, 20% TFE, or with a DPC micelle (Figures 4C–4G and S33). Both dendrimers were conformationally quite flexible under all three conditions; however, their branch comprising the α-peptide backbone of 12 residues in length extending from G0 to G3 mostly retained an α-helical conformation with 3 of the maximum possible 3.5 turns, thus showing a slightly stronger folding propensity than in our previous MD studies of AMPD L-**G3KL**, L-**T7**, and L-**T25**.^32^^,^^33^ The α-helix was less well formed in water compared with the simulation with TFE or with a DPC micelle, in line with the CD data (Figure S34).

The MD simulation with the DPC micelle was most interesting. AMPD L-**X18** and L-**X22** both rapidly approached the micelle surface (Figure 4C); however, the two dendrimers interacted very differently with the micelle in a manner consistent with their different folding behavior. The more α-helical L-**X18** remained rather compact, sitting at the water interface, with its α-peptide backbone as a fully folded, extensively solvent exposed, α-helix spanning residues G3-G0. The α-helix formed a binding motive that quickly extracted a DPC molecule from the micelle (<20 ns) before releasing it to the solvent (>165 ns) (Figures 4D and 4E). The binding mode involved lateral hydrophobic contacts between the DPC lipid tail and the Leu side chains in G0, G1, and G2, combined with hydrogen bonding and electrostatic interactions involving the phosphocholine head group and the N-terminal Lys residue in G3.

On the other hand, AMPD L-**X22**, which was less α-helical than L-**X18** as seen by CD, became flattened at the micelle surface and sank below the micelle surface (Figure 4C), with its α-helix directly in contact with the DPC micelle, engaging in multiple hydrophobic and H-bonding interactions over an extended patch of the micelle surface (Figures 4F and 4G). Despite these extensive contacts, the interaction between L-**X22** and the micelle was quite stable and did not result in the extraction of a DPC molecule as seen with L-**X18**.

### Why does α-helical folding favor hemolysis and why does conformational disorder favor antibacterial effects?

The switch from the hemolytic, non-antibacterial L- and D-**X18** to a non-hemolytic, antibacterial *sr*-**X18** must be triggered by a conformational effect. Our MD studies indicate α-helical folding along the α-peptide backbone of L-**X18** combining a strongly hydrophobic dendrimer core (*K*LL)_2_*K*LLL with cationic outer branches (KL)_8_(*K*LK)_4_. This α-helix seems capable of extracting a lipid from the zwitterionic DPC micelles, which could be interpreted as a model for the destabilization of eukaryotic membranes (hemolysis). The DPC extraction in the MD with L-**X18** might represent a general model for hemolytic AMPs presenting hydrophobic patches on their surface.^56^

**Figure 5:**
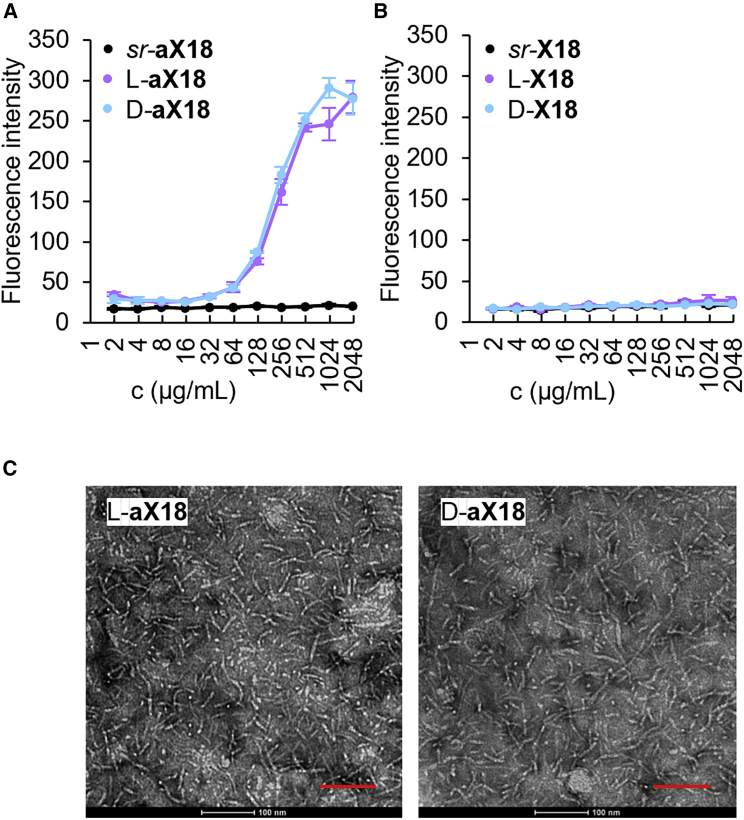
Aggregation of peptide dendrimers (A and B) AMPD *sr*-, L-, and D-**aX18** (A) and *sr*-, L-, and D-**aX18** (B) in PBS (pH 7.4) in presence of 0.2 μM Nile red. Fluorescence measured at *λ*_ex_ = 540 nm, *λ*_em_ = 615 nm. Data are presented in mean ± SD, n = 3. Experiments were performed ≥2 times in triplicates. (C) TEM images of AMPD (10 mg/mL) in PBS deposited on glow-discharged 400 mesh copper grids, dried, and stained by uranyl acetate. Scale bars are 100 nm.

The more generally accepted hypothesis to explain hemolysis involves aggregation of α-helically folded AMPs at the membrane surface leading to pore formation.^9^ This effect has been observed by atomic force microscopy on supported lipid bilayers and did not occur with peptide mixtures containing both L and D residues.^57^ Similarly, the exposed hydrophobic patch formed by the α-helix in L-**X18** could trigger aggregation, which would be blocked in *sr*-**X18**. We previously observed that peptide dendrimer aggregation can be controlled by stereochemistry with siRNA transfection dendrimers.^58^ Although we did not detect any aggregation of L-, D-, or *sr*-**X18** using Nile red,^59^ the close analogs **aX18** lacking N termini, which similarly switched from non-hemolytic in *sr* form to hemolytic in L or D form, indeed showed aggregation as pure enantiomers but not in *sr* form (Figures 5A, 5B, and S35). In addition, TEM images of L- and D-**aX18** showed filamentous aggregates, which were not observed with *sr*-**aX18** solutions (Figures 5C and S36).

The above models linking hydrophobic patches to hemolysis, either by enabling the extraction of lipid molecules from the membrane or via aggregation and pore formation, do not account for the loss of antibacterial effects when converting *sr*-**X18** to pure L- or D-enantiomers or for the antibacterial activity of the non-hemolytic L-, D- and *sr*-**X22**. The above mentioned study with L-/D-peptide mixtures^57^ proposes that bacterial membrane destabilization occurs by the carpet model, i.e., homogeneous spreading on the membrane surface, a generally accepted mechanism for AMPs.^9^ This mechanism would be accessible to *sr*-AMPD in similar manner to L-/D-peptide mixtures but also to the homochiral L- or D-**X22**, which would act independently of folding in an intrinsically disordered bioactive conformation. The more extensive membrane coverage and deeper insertion predicted by MD for L-**X22** could contribute to a better membrane perturbation via the carpet model and thus its higher antimicrobial activity compared with L-**X18** while remaining unaffected by stereorandomization. The MD study showing that L-**X18** folds but interacts less extensively with the DPC micelle than L-**X22** might indicate that L-**X18** cannot homogeneously coat the membrane, explaining its lack of antibacterial activity by the carpet model. On the other hand, the absence of a hydrophobic dendrimer core in *sr*-**X22** probably prevents it from becoming hemolytic in the homochiral form L-**X22**.

To conclude, we explored the possibility of discovering an intrinsically disordered AMPD by investigating a library of stereorandomized analogs of AMPD *sr*-**T25** selected by virtual screening using a fingerprint approach that does not take stereochemistry into account. HPLC and high-resolution mass spectrometry (HRMS) spectra are available in Figures S37–S190. Many of the *sr*-dendrimers were strongly antibacterial and non-hemolytic, confirming the favorable effect of stereorandomization on the therapeutic index previously discovered with *sr*-**T25**.

Investigating two of the most active AMPDs, *sr*-**X18** and *sr*-**X22**, revealed unexpected effects. In the first case, *sr*-**X18** lost its antibacterial activity while becoming strongly hemolytic and cytotoxic as pure enantiomers L- or D-**X18**, probably because α-helical folding of the α-peptide backbone of the dendrimer enabled a lipid extraction as observed by MD or aggregation at the membrane surface. This hypothesis was supported by observing aggregates with the close and similarly active analogs L- and D-**aX18**. In the second case, L- and D-**X22** were similarly antibacterial and non-hemolytic as *sr*-**X22**. Although CD spectra of L- and D-**X22** show partial α-helical content in a membrane-like environment, the similar activity of *sr*-, L-, and D-**X22** suggests that their bioactive antibacterial conformation is intrinsically disordered and probably favors the carpet model of membrane disruption as suggested by MD simulations with DPC micelles.

Screening stereorandomized libraries might be generally useful to identify homochiral peptides possessing an intrinsically disordered bioactive conformation or to optimize disordered peptides. Note that stereorandomization is distinct from using achiral amino acids, which may stabilize helical conformations (α-amino-isobutyric acid) or not (glycine).^20^ Our proof of concept focused on AMPD *sr*-**T25** because folding was known to be unnecessary for its antibacterial effects and the cause of its toxicity. The approach might be further applicable to optimize the sequence of intrinsically disordered linear peptides, for example the AMP indolicidin,^60^^,^^61^^,^^62^^,^^63^ or Leu-Lys oligomers known to tolerate mixed chirality arrangements for membrane disruption.^13^^,^^64^^,^^65^^,^^66^ The discovery of homochiral, intrinsically disordered AMPDs such as L- and D-**X22** matching the high activity and low toxicity of the parent *sr*-AMPD overcomes the intrinsic difficulty of characterizing the billions of stereoisomers composing *sr*-sequences (68,719,476,736 in the case of *sr*-**X22**) and therefore opens the way for further development of these compounds.

## Experimental procedures

### Resource availability

#### Lead contact

Further information and requests for resources should be directed to and will be fulfilled by the lead contact, Prof. Dr. Jean-Louis Reymond (jean-louis.reymond@unibe.ch).

#### Materials availability

All materials generated in this study are available from the lead contact.

## Data Availability

All data mentioned in this study will be made available upon request from the research community. The source code and dataset used for this study are available at GitHub: https://github.com/reymond-group/T25_analogs.

## Supplemental information

Document S1. Supplemental experimental procedures and Figures S1–S190

Document S2. Article plus supplemental information
