# Supplementary material for: An intrinsically disordered antimicrobial peptide dendrimer from stereorandomized virtual screening
Source: Cell Rep Phys Sci. 2022 Dec 21;3(12):101161. doi: 10.1016/j.xcrp.2022.101161 (PMC9780108; doi:10.1016/j.xcrp.2022.101161)
Supplement: Document S1. Supplemental experimental procedures and Figures S1–S190 [file mmc1.pdf]

Cell Reports Physical Science, Volume *3*

Supplemental information

An intrinsically disordered antimicrobial peptide dendrimer from stereorandomized virtual screening

Xingguang Cai, Markus Orsi, Alice Capecchi, Thilo Köhler, Christian van Delden, Sacha Javor, and Jean-Louis Reymond

Supplemental Experimental Procedures

An intrinsically disordered antimicrobial peptide dendrimer from stereorandomized virtual screening

Xingguang Cai,[a] *Markus Orsi,*[a] *Alice Capecchi,*[a] *Thilo Köhler,*[b] *Christian van Delden,*[b] *Sacha Javor*[a] *and Jean-Louis Reymond*[a]*

[a] Department of Chemistry, Biochemistry and Pharmaceutical Sciences, University of Bern, Freiestrasse 3, 3012 Bern, Switzerland; [b] Department of Microbiology and Molecular Medicine, University of Geneva; Service of Infectious Diseases, University Hospital of Geneva, 24 rue du Général-Dufour, 1211 Genève, Switzerland

E-Mail: jean-louis.reymond@unibe.ch

Table Of Contents

1. Cheminformatics....3

2. Solid phase synthesis of peptide dendrimers....4

3. MIC determination at different pH values....7

4. Hemolysis assay....11

5. Acid-base titration....12

6. Cellular Toxicity....13

7. Human serum stability....14

8. Time kill kinetics assay....15

9. Transmission electron microscopy (TEM) for bacterial samples....17

10. NPN Membrane Permeability Assay....26

11. DiSC$_3$(5) Membrane Depolarization Assay....28

12. Cell Membrane Permeability Assay....30

13. Circular dichroism (CD) spectroscopic measurements....47

14. Molecular Dynamics (MD)....50

15. Critical Micellar Concentration (CMC)....56

16. Aggregation of peptide dendrimers....57

1. Cheminformatics

50,625 dendrimer sequences with all possible permutations of up to three residues of Lys or Leu in the branches and Lys as branching diamino acid was enumerated and encoded using MXFP. The CBD between the MXFP of each dendrimer to the MXFP of **T25** was calculated, and the sequences were sorted from the lowest to the highest MXFP CBD values. Then, four different sequences selection were performed. (i) The best 20 sequences were selected. (ii) The 200 sequences with lowest MXFP CBD were selected and clustered in 20 clusters, then the closest sequence to **T25** and one randomly picked sequence were selected from each cluster. (iii) The 1000 sequences with lowest MXFP CBD were selected and only the one with a Lys/Leu ratio between 0.7 and 1.5 were kept. In the Lys/Leu ratio calculation branching Lys were excluded. The resulting 443 sequences were clustered in 20 clusters, and the closest sequence from each cluster was selected. (iv) The 1000 sequences with lowest MXFP CBD were selected and clustered in 20 clusters, and the closest sequence from each cluster was selected. In the last three mentioned approaches, clusters were formed with k-means clustering and the sequences MXFP CBD. The selection process resulted in 80 sequences, 63 of which were unique and were selected for synthesis.

2. Solid phase synthesis of peptide dendrimers

Dimethylformamide (DMF) was purchased from Thommen-Furler AG, Buren, Switzerland. Dichloromethane (DCM), methanol and *tert*-butylmethylether (TBME) were purchased from Dr. Grogg Chemmie AG, Stettlen-Deisswil, Switzerland. Piperidine was purchased from Acros Organics, Geel, Belgium. *N, N′*-Diisopropylcarbodiimid (DIC) and Boc-*6*-aminocaproic acid-OH was purchased from Iris biotech GMBH Markredwitz, Germany. Trifluoroacetic acid (TFA) and triisopropylsilane (TIS) was purchased from fluorochem Ltd., Hadfield, U. K. *2,4,6*-trinitrobenzenesulfonic acid (TNBS) was purchased from Sigma-Aldrich cheimie GmbH, Steinheim, Germany.

Manual solid phase peptide synthesis

Peptide synthesis was carried out manually with TentaGel S RAM resin (0.22 mmol/g). Firstly, resin was swelled in DCM and the Fmoc-protecting groups of the resin were removed with a solution of 20% piperidine in DMF (2 × 10 min). For further couplings, the resin was acylated with one of the protected amino acids (5 eq./amine), OxymaPure (6 eq./amine) and DIC (6 eq./amine) in DMF. Fmoc-protected amino acids, derivatives or diamino acids were coupled for two times 1 h (G0), two times 1 h (G1), three times 2 h (G2) and three times 2 h + one time overnight (G3). The completion of the reaction was checked using TNBS. The coupling was repeated after a positive test. After each coupling, the resin was deprotected with 20% piperidine in DMF (first time 1 min, second time 5 min). Before each coupling and deprotection step, the resin was washed with DMF 3 times, MeOH 3 times and DCM 3 times.

Automated solid phase peptide synthesis

Automated microwave synthesis was performed with Liberty Blue CEM synthesizer. Rink Amide MBHA resin (0.25 mmol·g^{-1}) was swelled in DCM before transported into the reaction pot. the Fmoc-protecting groups of the resin were removed with a solution of 20% piperidine

in DMF (first time 1 min, second time 5 min) at 75 °C. For further couplings, the resin was acylated with one of the protected amino acids (3 eq./amine), OxymaPure (3 eq./amine) and DIC (3 eq./amine) in DMF. Fmoc-protected amino acids, derivatives or diamino acids were coupled for two times 8 min (G0), two times 8 min (G1), three times 8 min (G2) at 75 °C. After each coupling, the resin was deprotected with 20% piperidine in DMF (first time 1 min, second time 5 min) at 75 °C. Before each coupling and deprotection step, the resin was washed with DMF 5 times. G3 was performed manually in the procedure as described before.

Semi-automated solid phase peptide synthesis

Semi-automated synthesis was performed with an in-house built synthetiser in comprise of a heating element, keeping the temperature at 50 °C, glass reaction vessels and a vacuum operated filtration system. Rink Amide MBHA resin (0.38 $mmol\cdot g^{-1}$) was swelled in DCM before transported into the reaction vessels. the Fmoc-protecting groups of the resin were removed with a solution of 20% piperidine in DMF (first time 1 min, second time 5 min) at 75 °C. For further couplings, the resin was acylated with one of the protected amino acids (3 eq./amine), OxymaPure (3 eq./amine) and DIC (3 eq./amine) in DMF. Fmoc-protected amino acids, derivatives or diamino acids were coupled for two times 8 min (G0), two times 8 min (G1), three times 8 min (G2) and six times (G3) at 60 °C. After each coupling, the resin was deprotected with 20% piperidine in DMF (first time 1 min, second time 5 min) at 60 °C. Before each coupling and deprotection step, the resin was washed with DMF 5 times.

Cleavage and Purification

The cleavage was carried out by treating the resins with TFA/TIS/H_2O (94:5:1 v/v/v) solution for 4.5 h. After filtration, the peptide solutions were precipitated with 35 mL of TBME, centrifuged for 10 min at 4400 rpm, and washed twice with TBME. For purification, the crude peptide was dissolved in A (100% mQ-H_2O, 0.05% TFA), subjected to preparative RP-HPLC

and obtained as TFA salt after lyophilization. B was 10% mQ-water, 90% acetonitrile, 0.05% TFA. The fractions of the crudes were then lyophilized. Yields are given as SPPS total yields. In all cases, yields are calculated for the corresponding TFA salts.

Determination

Analytical RP-HPLC-MS was performed with an Ultimate 3000 Rapid Separation LC-MS System (DAD-3000RS diode array detector) using an Acclaim RSLC 120 C18 column (2.2 μm, 120 Å, 3×50 mm, flow 1.2 mL/min) from Dionex. Data recording and processing was done with Dionex Chromeleon Management System Version 6.80 (analytical RP-HPLC). All RP-HPLC were using HPLC-grade acetonitrile and Milli-Q deionized water. The elution solutions were: A Milli-Q deionized water containing 0.05% TFA; B Milli-Q deionized water/acetonitrile (10:90, v/v) containing 0.05% TFA. MS spectra were recorded on a Thermo Scientific LTQ OrbitrapXL.

HRMS spectra were provided by the MS analytical service of the Department of Chemistry, Biochemistry and Pharmaceutical Sciences at the University of Bern (group PD Dr. Stefan Schürch). [1]

3. MIC determination at different pH values

Mueller-Hinton (MH) medium was prepared at different pH. MH broth (Sigma Aldrich, Steinheim, Germany) was dissolved in 1 L of mQ water, adjust with 1 M NaOH or 1 M HCl until final pH is 5.0, 7.4 or 8.0. 0.1 M NaOH and 0.1 M HCl were used for precise adjustments. Medium was sterilized by autoclaving at 121 °C for 15 minutes.

Antimicrobial activity was assayed against *E. coli* W3110, *Acinetobacter baumannii* ACTT 19606, *P. aeruginosa* PAO1, *K. pneumoniae* NCTC 418, methicillin-resistant *Staphylococcus aureus* COL. To determine MIC, broth microdilution method was used. A colony of bacteria was grown in LB (Lysogeny broth) medium overnight at 37 °C. The compounds were prepared as stock solutions of 8 mg/mL in mQ-H_2O, diluted to the initial concentration of 64 µg/mL in 300 µL MH medium, added to the first well of 96-well microtiter plate (TPP, untreated) and diluted serially by ½. The concentration of the bacteria was quantified by measuring OD_{600} and diluted to OD_{600} = 0.022 in MH medium. The sample solutions (150 µL) were mixed with 4 µL diluted bacterial suspension with a final inoculation of about of 5×10^5 CFU. The plates were incubated at 37 °C until satisfactory growth (~18 h). For each test, two columns of the plate were kept for sterility control (broth only) and growth control (broth with bacterial, no antibiotics). The MIC was defined as the lowest concentration of the peptide dendrimer that inhibited visible growth of the tested bacteria, as detected after treatment with MTT. The assay was performed in the biosafety level 2 lab and was repeated at least two times. [2]

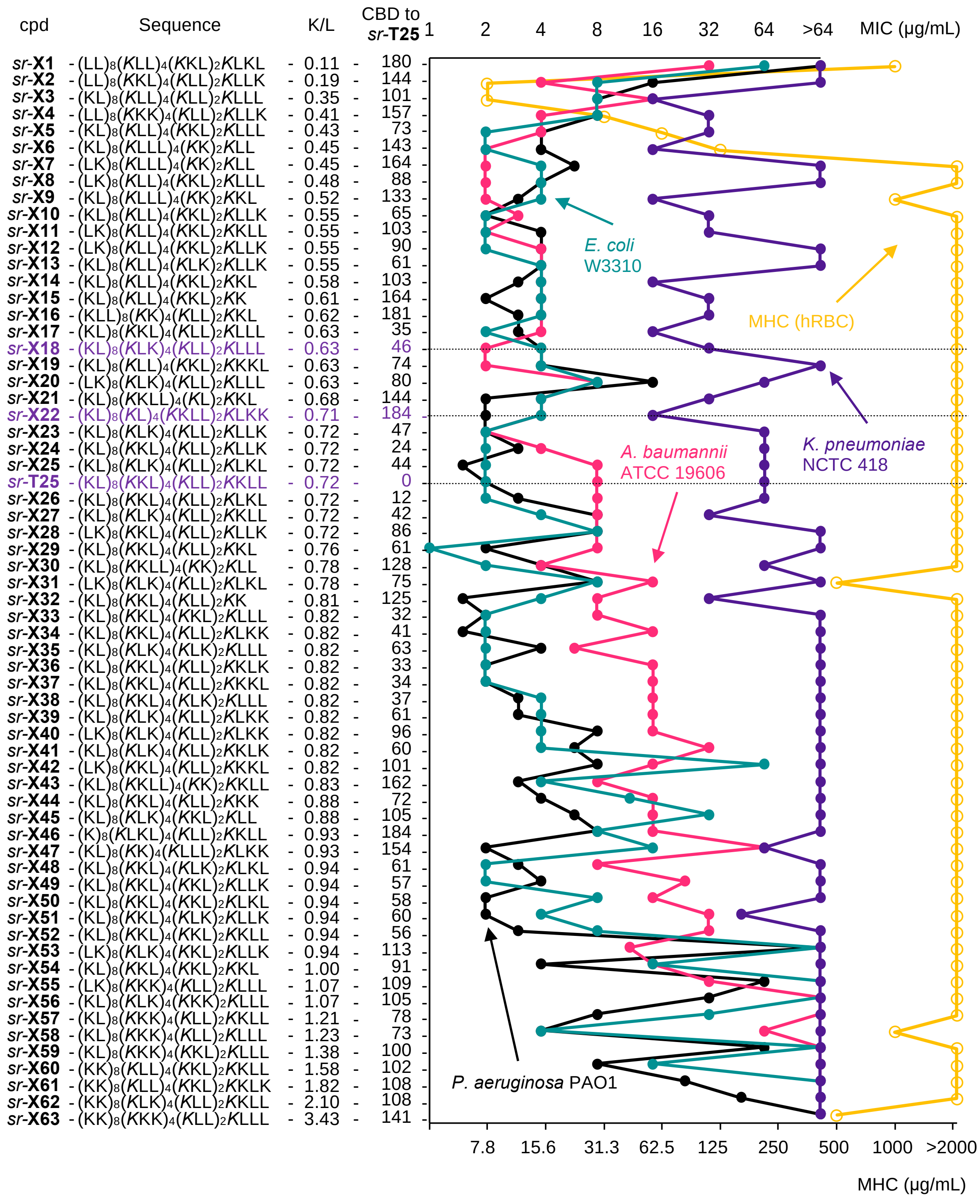

Figure S1. Sequences and activity profiling of *sr*-AMPDs. The sequence notation uses italics *K* to denote branching lysine residues. The K/L ratio is computed excluding branching lysines. CBD = city-block distance to *sr*-**T25** in MXFP fingerprint. MIC in µg/mL were measured in Müller–Hinton (MH) medium at pH 7.4 on *E. coli* W3110, *P. aeruginosa* PAO1, *A. baumannii* ATCC 19606, and *K. pneumoniae* NCTC 418 after incubation for 16–20 h at 37 °C. MHC in µg/mL were measured on human red blood cells in phosphate buffered saline pH 7.4 at room temperature after incubation for 4 h. Each result represents two independent experiments performed in duplicate. See **Table S1** for values and additional data.

Table S1. antimicrobial activities and hemolysis of AMPDs [a]

		***P. aeruginosa* PA01**	***A. baumannii* ATCC 19606**	***E. coli* W3310**	***K. pneumoniae* NCTC 418**	**MHC [b]**
**PMB**		0.25	0.125	0.125	0.5	>2000
***sr*-T25**	(*K*L)$_8$(*K*KL)$_4$(*K*LL)$_2$*K*KLL	2	8	2	64	>2000
***sr*-X1**	(LL)$_8$(*K*LL)$_4$(*K*KL)$_2$*K*LKL	128	32	64	>64	1000
***sr*-X2**	(LL)$_8$(*K*KL)$_4$(*K*LL)$_2$*K*LLK	16	4	8	>128	<15.6
***sr*-X3**	(*K*L)$_8$(*K*LL)$_4$(*K*LL)$_2$*K*LLL	8	16	8	16	<15.6
***sr*-X4**	(LL)$_8$(*K*KK)$_4$(*K*LL)$_2$*K*LLK	8	4	8	32	31.3
***sr*-X5**	(*K*L)$_8$(*K*LL)$_4$(*K*KL)$_2$*K*LLL	4	4	2	32	62.5
***sr*-X6**	(*K*L)$_8$(*K*LLL)$_4$(*K*K)$_2$*K*LL	4	2	2	16	125
***sr*-X7**	(LK)$_8$(*K*LLL)$_4$(*K*K)$_2$*K*LL	4-8	2	4	128	>2000
***sr*-X8**	(LK)$_8$(*K*LL)$_4$(*K*KL)$_2$*K*LLL	4	2	4	>64	>2000
***sr*-X9**	(*K*L)$_8$(*K*LLL)$_4$(*K*K)$_2$*K*KL	2-4	2	4	16	1000
***sr*-X10**	(*K*L)$_8$(*K*LL)$_4$(*K*KL)$_2$*K*LLK	2	2-4	2	32	>2000
***sr*-X11**	(LK)$_8$(*K*LL)$_4$(*K*KL)$_2$*K*KLL	4	2	2	32	>2000
***sr*-X12**	(LK)$_8$(*K*LL)$_4$(*K*KL)$_2$*K*LLK	4	4	2	128	>2000
***sr*-X13**	(*K*L)$_8$(*K*LL)$_4$(*K*LK)$_2$*K*LLK	4	4	4	128	>2000
***sr*-X14**	(*K*L)$_8$(*K*LL)$_4$(*K*KL)$_2$*K*KL	2-4	4	4	16	>2000
***sr*-X15**	(*K*L)$_8$(*K*LL)$_4$(*K*KL)$_2$*K*K	2	4	4	32	>2000
***sr*-X16**	(*K*LL)$_8$(*K*K)$_4$(*K*LL)$_2$*K*KL	2-4	4	4	32	>2000
***sr*-X17**	(*K*L)$_8$(*K*KL)$_4$(*K*LL)$_2$*K*LLL	2-4	4	2	16	>2000
***sr*-X18**	(*K*L)$_8$(*K*LK)$_4$(*K*LL)$_2$*K*LLL	4	2	4	32	>2000
***sr*-X19**	(*K*L)$_8$(*K*LL)$_4$(*K*KL)$_2$*K*KKL	4	2	4	>64	>2000
***sr*-X20**	(LK)$_8$(*K*LK)$_4$(*K*LL)$_2$*K*LLL	16	8	8	64	>2000
***sr*-X21**	(*K*L)$_8$(*K*KLL)$_4$(*K*L)$_2$*K*KL	2	4	4	32	>2000
***sr*-X22**	(*K*L)$_8$(*K*L)$_4$(*K*KLL)$_2$*K*LKK	2	4	4	16	>2000
***sr*-X23**	(*K*L)$_8$(*K*LK)$_4$(*K*LL)$_2$*K*LLK	2	2	2	64	>2000
***sr*-X24**	(*K*L)$_8$(*K*KL)$_4$(*K*LL)$_2$*K*LLK	2-4	4	2	64	>2000
***sr*-X25**	(*K*L)$_8$(*K*LK)$_4$(*K*LL)$_2$*K*LKL	1-2	8	2	64	>2000
***sr*-X26**	(*K*L)$_8$(*K*KL)$_4$(*K*LL)$_2$*K*LKL	2-4	8	2	64	>2000
***sr*-X27**	(*K*L)$_8$(*K*LK)$_4$(*K*LL)$_2$*K*KLL	8	8	4	32	>2000
***sr*-X28**	(LK)$_8$(*K*KL)$_4$(*K*LL)$_2$*K*LLK	8	8	8	>64	>2000
***sr*-X29**	(*K*L)$_8$(*K*KL)$_4$(*K*LL)$_2$*K*KL	2	8	1	64-128	>2000
***sr*-X30**	(*K*L)$_8$(*K*KLL)$_4$(*K*K)$_2$*K*LL	4	4	2	64	>2000
***sr*-X31**	(LK)$_8$(*K*LK)$_4$(*K*LL)$_2$*K*LKL	8	16	8	>64	>2000
***sr*-X32**	(*K*L)$_8$(*K*KL)$_4$(*K*LL)$_2$*K*K	1-2	8	4	32	>2000
***sr*-X33**	(*K*L)$_8$(*K*KL)$_4$(*K*KL)$_2$*K*LLL	2	8	2	128	>2000
***sr*-X34**	(*K*L)$_8$(*K*KL)$_4$(*K*LL)$_2$*K*LKK	1-2	16	2	128	>2000
***sr*-X35**	(*K*L)$_8$(*K*LK)$_4$(*K*LK)$_2$*K*LLL	4	4-8	2	>128	>2000
***sr*-X36**	(*K*L)$_8$(*K*KL)$_4$(*K*LL)$_2$*K*KLK	2	16	2	128	>2000
***sr*-X37**	(*K*L)$_8$(*K*KL)$_4$(*K*LL)$_2$*K*KKL	2	16	2	128	>2000
***sr*-X38**	(*K*L)$_8$(*K*KL)$_4$(*K*LK)$_2$*K*LLL	2-4	16	4	>128	>2000
***sr*-X39**	(*K*L)$_8$(*K*LK)$_4$(*K*LL)$_2$*K*LKK	2-4	16	4	128	>2000
***sr*-X40**	(LK)$_8$(*K*LK)$_4$(*K*LL)$_2$*K*LKK	8	16	4	>128	>2000
***sr*-X41**	(*K*L)$_8$(*K*LK)$_4$(*K*LL)$_2$*K*KLK	4-8	32	4	>128	>2000
***sr*-X42**	(LK)$_8$(*K*KL)$_4$(*K*LL)$_2$*K*KKL	8	16	64	>128	>2000
***sr*-X43**	(*K*L)$_8$(*K*KLL)$_4$(*K*K)$_2$*K*KLL	2-4	8	4	>64	>2000
***sr*-X44**	(*K*L)$_8$(*K*KL)$_4$(*K*LL)$_2$*K*KK	4	16	8-16	>64	>2000
***sr*-X45**	(*K*L)$_8$(*K*LK)$_4$(*K*KL)$_2$*K*LL	4-8	16	32	>64	>2000
***sr*-X46**	(*K*)$_8$(*K*LKL)$_4$(*K*LL)$_2$*K*KLL	8	16	8	>64	500
***sr*-X47**	(*K*L)$_8$(*K*K)$_4$(*K*LLL)$_2$*K*LKK	2	64	16	64	>2000
***sr*-X48**	(*K*L)$_8$(*K*KL)$_4$(*K*LK)$_2$*K*LKL	2-4	8	2	>64	>2000
***sr*-X49**	(*K*L)$_8$(*K*KL)$_4$(*K*KL)$_2$*K*LLK	4	16-32	2	>128	>2000
***sr*-X50**	(*K*L)$_8$(*K*KL)$_4$(*K*KL)$_2$*K*LKL	2	16	8	>128	>2000
***sr*-X51**	(*K*L)$_8$(*K*KL)$_4$(*K*LK)$_2$*K*LLK	2	32	4	32-64	>2000
***sr*-X52**	(*K*L)$_8$(*K*KL)$_4$(*K*KL)$_2$*K*KLL	2-4	32	8	>128	>2000
***sr*-X53**	(LK)$_8$(*K*LK)$_4$(*K*KL)$_2$*K*LLK	128	8-16	128	>128	>2000
***sr*-X54**	(*K*L)$_8$(*K*KL)$_4$(*K*KL)$_2$*K*KL	4	16	16	>64	>2000
***sr*-X55**	(LK)$_8$(*K*KK)$_4$(*K*LL)$_2$*K*LLL	64	32	128	>128	>2000
***sr*-X56**	(*K*L)$_8$(*K*LK)$_4$(*K*KK)$_2$*K*LLL	32	128	128	>64	>2000
***sr*-X57**	(*K*L)$_8$(*K*KK)$_4$(*K*LL)$_2$*K*KLL	8	128	32	>64	1000
***sr*-X58**	(*K*L)$_8$(*K*KK)$_4$(*K*LL)$_2$*K*LLL	4	64	4	>64	>2000
***sr*-X59**	(*K*L)$_8$(*K*KK)$_4$(*K*KL)$_2$*K*LLL	64	128	128	>64	>2000

***sr*-X60**	(*K*K)$_{8}$(*K*LL)$_{4}$(*K*KL)$_{2}$*K*KLL	8	128	16	>64	>2000
***sr*-X61**	(*K*K)$_{8}$(*K*LL)$_{4}$(*K*KL)$_{2}$*K*KLK	16-32	128	128	>64	>2000
***sr*-X62**	(*K*K)$_{8}$(*K*LK)$_{4}$(*K*LL)$_{2}$*K*KLL	32-64	128	128	>64	>2000
***sr*-X63**	(*K*K)$_{8}$(*K*KK)$_{4}$(*K*LL)$_{2}$*K*LLL	128	128	128	>64	500

a) MIC = minimal inhibitory concentration in µg/mL, measured in Müller–Hinton (MH) medium at pH 7.4 *E. coli* W3110, *P. aeruginosa* PAO1, *A. baumannii* ATCC 19606, and *K. pneumoniae* NCTC 418 after incubation for 16–20 h at 37 °C. Each result represents two independent experiments performed in duplicate. b) Minimum hemolytic concentration (MHC) measured on human red blood cells in phosphate buffered saline pH 7.4 at room temperature for 4 h. Each result represents two independent experiments performed in duplicate.

4. Hemolysis assay

Compounds were subjected to a hemolysis assay to assess the hemolytic effect on human red blood cells (hRBCs). The blood was obtained from Interregionale Blutspende SRK AG, Bern, Switzerland. 1.5 mL of whole blood was centrifuged at 3000 rpm for 15 minutes at 4 °C. The plasma was discarded, and the hRBC pellet was re-suspended in 5 mL of PBS (pH 7.4) then centrifuged at 3000 rpm for 5 minutes at 4 °C. The washing of hRBC was repeated three times and the remaining pellet was re-suspended in 10 mL of PBS.

The peptide samples were prepared at the initial concentration of 4 mg/mL in PBS, 100 µL of initial concentration was added to the first well of 96-well microtiter plate (TPP, V-bottomed, untreated) and diluted serially by ½. Controls on each plate included a blank medium control (PBS 50 µL) and a hemolytic activity control (0.1% Triton™ X-100). 50 µL of hRBC suspension was incubated with 50 µL of each sample in PBS in 96-well plate (Nunc 96-Well Polystyrene Conical Bottom MicroWell Plates). The final concentration of dendrimer in the first well is 2 mg/ml. After the plates were incubated for 4 h at room temperature, minimal hemolytic concentration (MHC) was determined by visual inspection of the wells. [2]

.

5. Acid-base titration

Peptide samples (13.00-16.00 mg) were diluted in Milli-Q water 10.0 mL (final concentration of dendrimers is 1.00 mg/mL) and acidified to pH ~3 with 1 M HCl. Then, 0.1 M NaOH was added in step of 2 µL with a Dosimat plus (Metrohm, Zofingen, Switzerland) and pH was measured on a Metrohm 692 pH/ion meter.[2][3]

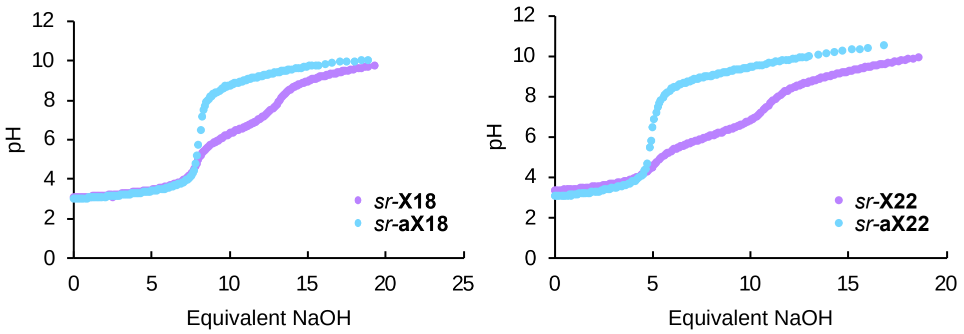

Figure S2. Acid-base titration curves of *sr*-**X18**, *sr*-**aX18**, *sr*-**X22** and *sr*-**aX22**.

6. Cellular Toxicity

HEK 293 cell line were cultured and maintained in DMEM (Dulbeccos modified Eagle medium, Sigma Aldrich) supplemented with 10% FBS (Sigma Aldrich) and 1% penicillin/streptomycin. Cells were incubated in a humidified incubator at 37°C in the presence of 5% CO_2.

HEK 293 cell were seeded into 96 well plates at the density of 2 × 103 cells/well and incubated in DMEM containing 10% FBS for 10-14 hours at 37°C in the presence of 5% CO_2. The next day, stock solution of compounds were prepared at concentration of 2 mg/mL in DMEM containing 10% FBS. 1/2 or 1/3 serial dilution of the peptide dendrimers from 2 mg/mL or 200 µg/mL were performed in DMEM containing 10% FBS. The medium was removed from cell culture and replaced with the compounds at different concentration after washing with PBS. The cells were incubated in DMEM containing 10% FBS at 37°C in the presence of 5% CO_2. Negative control was untreated cell culture without compounds and blank control was wells without cell but only medium. After 48 hours, a final concentration of 10% AlamarBlue® (Thermo Fisher Scientific, Reinach, CH) was added to each well. The cells were incubated for 3-5 hours at 37°C with 5% CO_2. The fluorescence was then measured on a Tecan Infinite M1000 Pro plate reader at λex = 560 nm and λem = 590 nm. The value was normalized according to the untreated cells.

7. Human serum stability

Peptide dendrimers were prepared as 400 µM stock solutions in 0.1 M Tris-HCl pH 7.4 buffer with 4-hydroxybenzoic acid as internal standard (100 µg/mL). 25% Human serum was prepared in 0.1 M Tris-HCl pH 7.4 buffer. Proteolysis was initiated upon addition of 50 µL of the test peptide dendrimer to 50 µL to human serum (25%) and shaking at 350 rpm and 37 °C. The final peptide concentration was 200 µM. The reactions were analyzed at 0, 1, 6, 12 and 24 hours after addition of 100 µL of 0.1 M $ZnSO_4$/acetonitrile (1:1) solution. The samples were cooled for 10 min and the supernatant was collected for each sample after centrifugation at 12 000 rpm for 10 minutes. Supernatants were carefully taken out and centrifuged again at 12 000 rpm for 10 minutes. Supernatants after second centrifuge was analyzed by RP-UPLC (flow rate: 1.2 $mL \cdot min^{-1}$. gradient: A/B=100/0 to 0/100 in 3.5 min). Conversions were calculated by quantification of the remaining peptide and peptide dendrimers determined by integration of the area of the chromatogram peak in analytical RP-HPLC. Experiments were done in triplicates. [4]

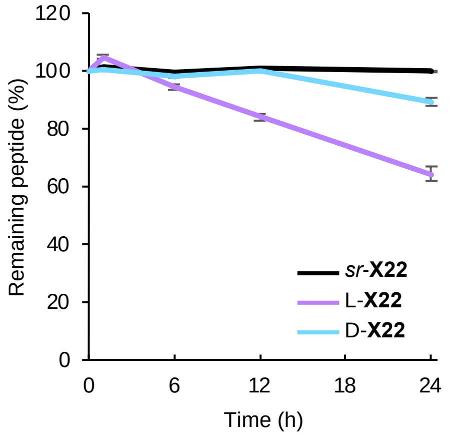

Figure S3. Serum stability of *sr*-**X22**, L-**X22**, D-**X22** and **G3KL.**

8. Time kill kinetics assay

A single colony of *P. aeruginosa* PAO1 was picked and grown overnight with shaking (180 rpm) in LB (Sigma Aldrich, Buchs, Switzerland) medium 5 mL overnight at 37 °C. The overnight bacterial culture was diluted to OD_{600} 0.002 (2 x 10^6 CFU/mL) in fresh MH (Sigma Aldrich, Buchs, Switzerland) medium. Stock solutions of AMPDs in sterilized milliQ water were prepared in 1 mg/mL and were diluted to two times more than required concentration in fresh MH (Sigma Aldrich, Buchs, Switzerland) medium at pH 7.4. 100 µL prepared bacteria solution in MH and 100 µL samples in MH were mixed in 96-well microtiter plate (TPP, untreated, Corning Incorporated, Kennebunk, USA). Untreated bacteria at 1 x 10^6 CFU/mL were used as a growth control.

96-well microtiter plates were incubated in 37 °C with shaking (180 rpm). Surviving bacteria were quantified at 0, 0.5, 1, 2, 3, 4, 5 and 6 hours by plating 10-fold dilutions of sample in sterilized normal saline on LB agar plates. LB agar plates were incubated at 37 °C for 10 hours and the number of individual colonies was counted at each time-point. The assay was performed in triplicate in the biosafety level 2 lab and repeated at least twice. [2]

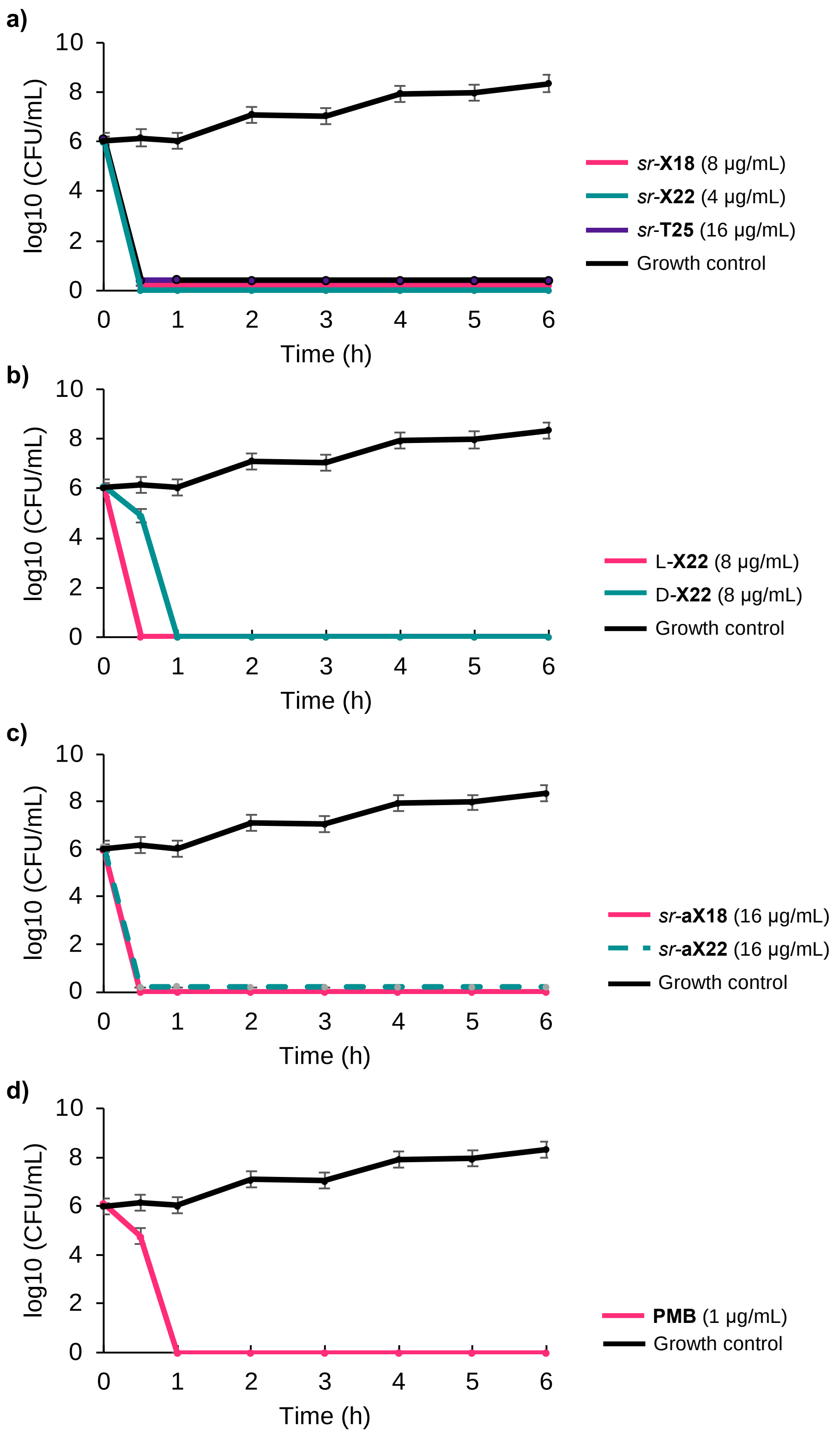

Figure S4. Bacteria killing assay at pH 7.4 against *P. aeruginosa* PAO1 at a concentration of 2 × MIC.

9. Transmission electron microscopy (TEM) for bacterial samples

Exponential phase (1 mL, OD_{600} = 1) of bacteria were washed with MH medium and treated with **AMPDs** in MH medium at pH 7.4. Each time, 1 mL of the bacteria were centrifuged after 2 hours at 12 000 rpm for 3 min and fixed overnight with 2.5% glutaraldehyde (Agar Scientific, Stansted, Essex, UK) in 0.15 M HEPES (Fluka, Buchs, Switzerland) with an osmolarity of 670 mOsm and adjusted to a pH of 7.35. The next day, samples were washed with 0.15 M HEPES three times for 5 min, postfixed with 1% OsO_4 (SPI Supplies, West Chester, USA) in 0.1 M Na-cacodylate-buffer (Merck, Darmstadt, Germany) at 4 °C for 1 h. Thereafter, bacteria cells were washed in 0.1 M Na-cacodylate-buffer three times for 5 min and dehydrated in 70, 80, and 96% ethanol (Alcosuisse, Switzerland) for 15 min each at room temperature. Subsequently, they were immersed in 100% ethanol (Merck, Darmstadt, Germany) three times for 10 min, in acetone (Merck, Darmstadt, Germany) two times for 10 min, and finally in acetone-Epon (1:1) overnight at room temperature. The next day, bacteria cells were embedded in Epon (Fluka, Buchs, Switzerland) and hardened at 60 °C for 5 days. Sections were produced with an ultramicrotome UC6 (Leica Microsystems, Vienna, Austria), first semithin sections (1µm) for light microscopy which were stained with a solution of 0.5% toluidine blue O (Merck, Darmstadt, Germany) and then ultrathin sections (70-80 nm) for electron microscopy. The sections, mounted on single slot copper grids, were stained with 1% uranyl acetate at 40 °C for 30 min and 3% lead citrate at RT for 20 min or UranyLess (Electron Microscopy Sciences, Hatfield, UK) at 40 °C for 10 min and 3% lead citrate at 25 °C for 10 min with an ultrostainer (Leica Microsystems, Vienna, Austria). Sections were then examined with a Tecnai Spirit transmission electron microscope equipped with two digital cameras (FEI Eagle CCD Camera). The growth, incubation and fixation were performed in the biosafety level 2 lab.[2,5]

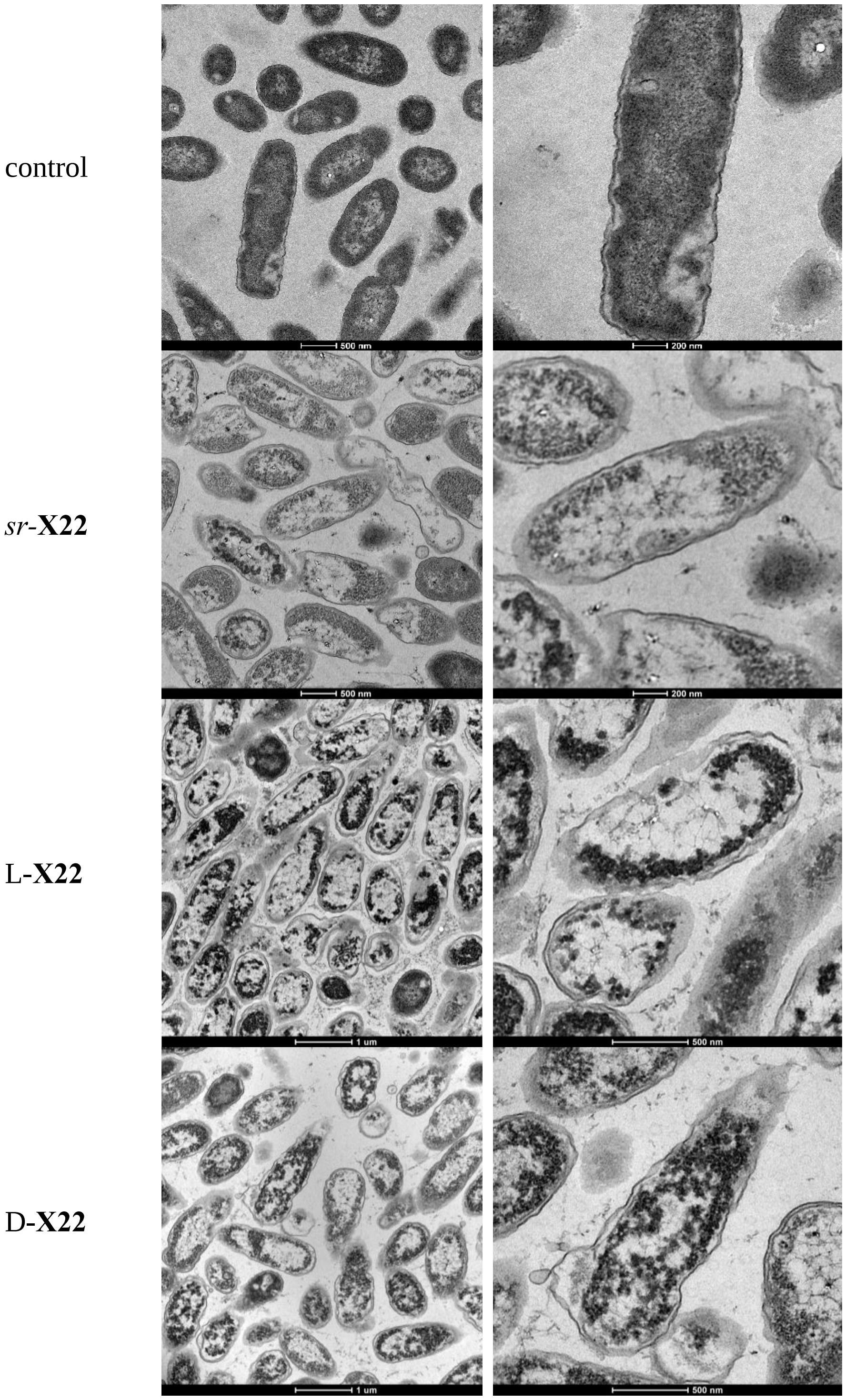

Figure S5. TEM images of *P. aeruginosa* PAO1, 2 h after treatment with *sr*-**X22** (20 µg/mL), L-**X22** (40 µg/mL), D-**X22** (40 µg/mL) and non-treated control in MH medium at pH 7.4.

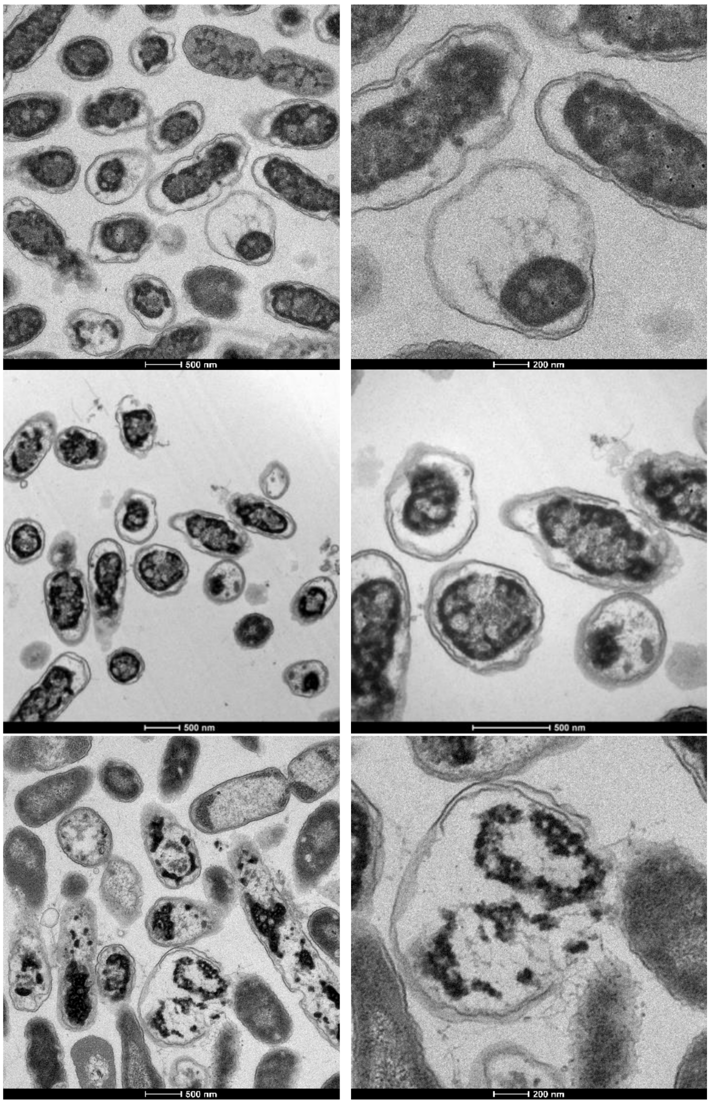

sr-**aX18**

sr-**aX22**

sr-**T25**

Figure S6. TEM images of *P. aeruginosa* PAO1, 2 h after treatment with *sr*-**aX18** (80 µg/mL), *sr*-**aX22** (80 µg/mL), and *sr*-**T25** (80 µg/mL) in MH medium at pH 7.4.

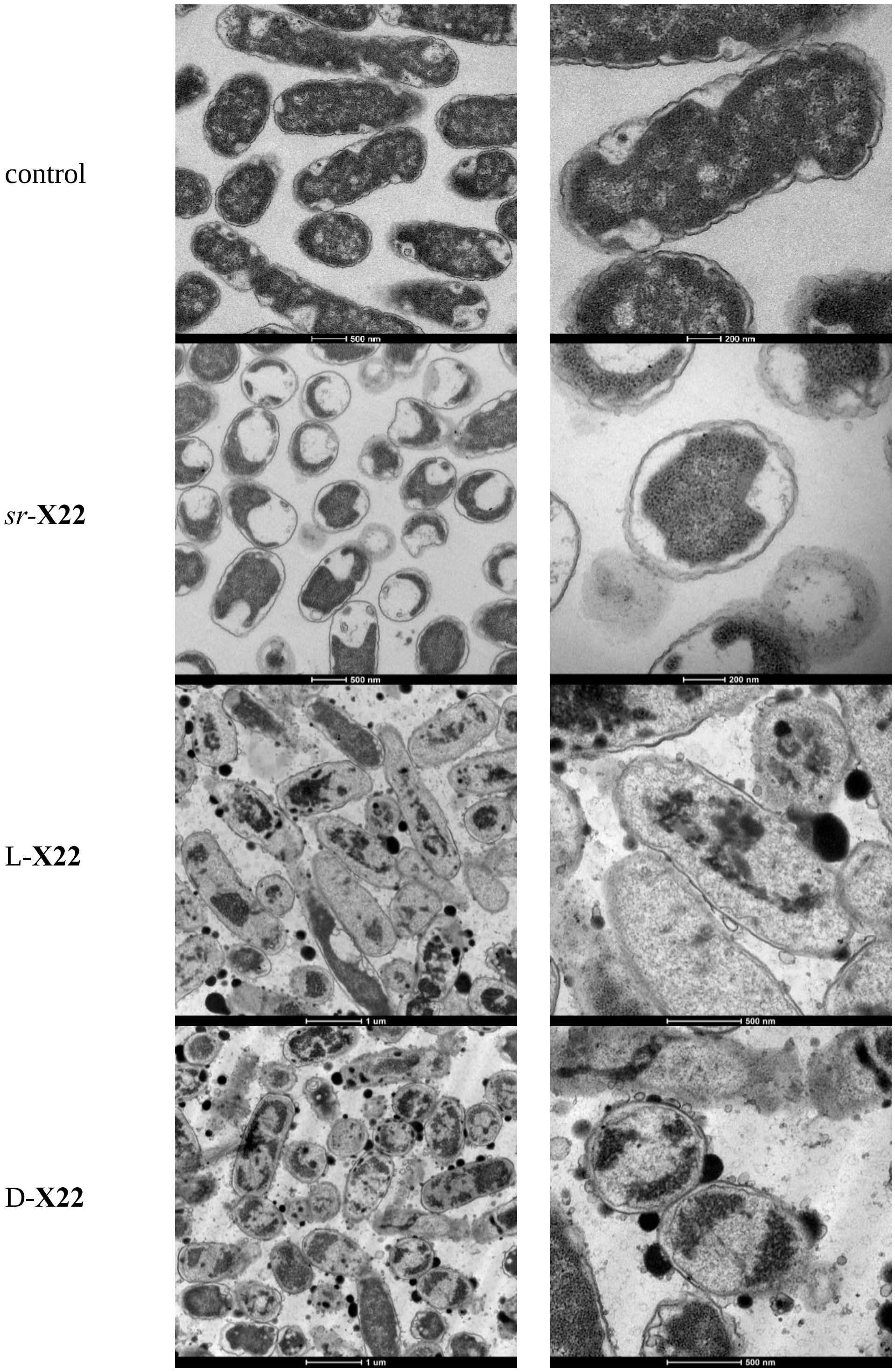

Figure S7. TEM images of *E. coli*, 2 h after treatment with *sr*-**X22** (40 µg/mL), L-**X22** (40 µg/mL), D-**X22** (40 µg/mL) and non-treated control in MH medium at pH 7.4.

sr-**aX18**

sr-**aX22**

sr-**T25**

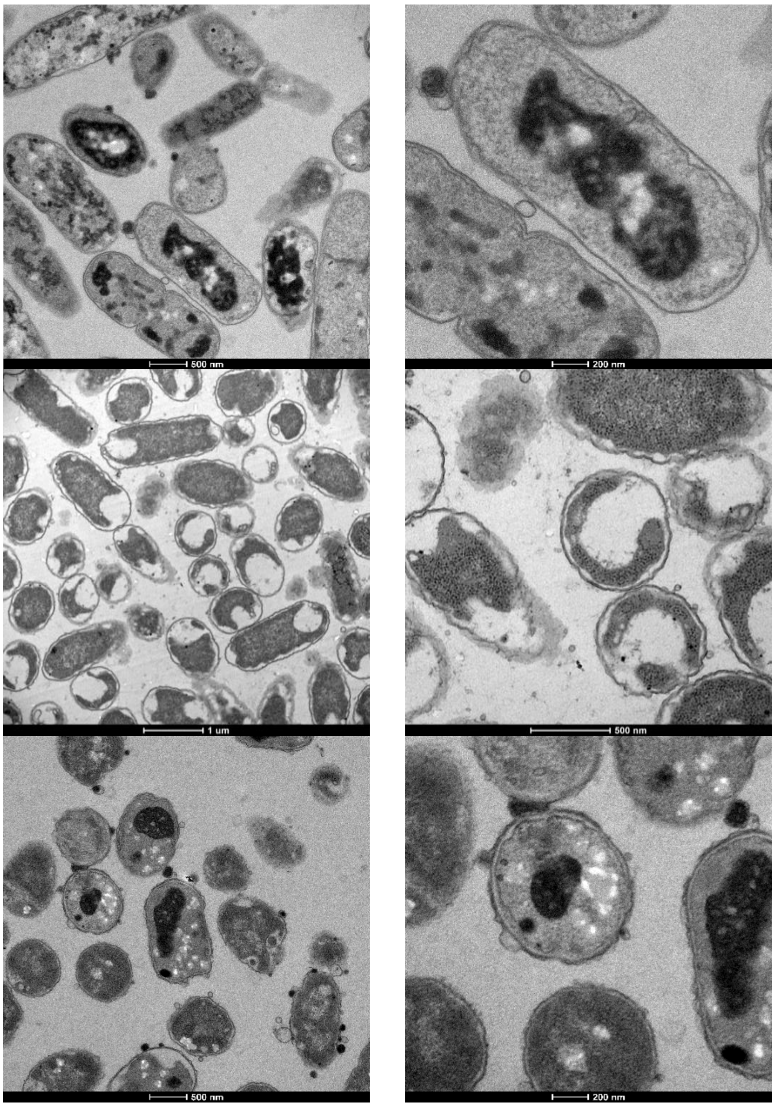

Figure S8. TEM images of *E. coli*, 2 h after treatment with *sr*-**aX18** (40 µg/mL), *sr*-**aX22** (20 µg/mL), and *sr*-**T25** (40 µg/mL) in MH medium at pH 7.4.

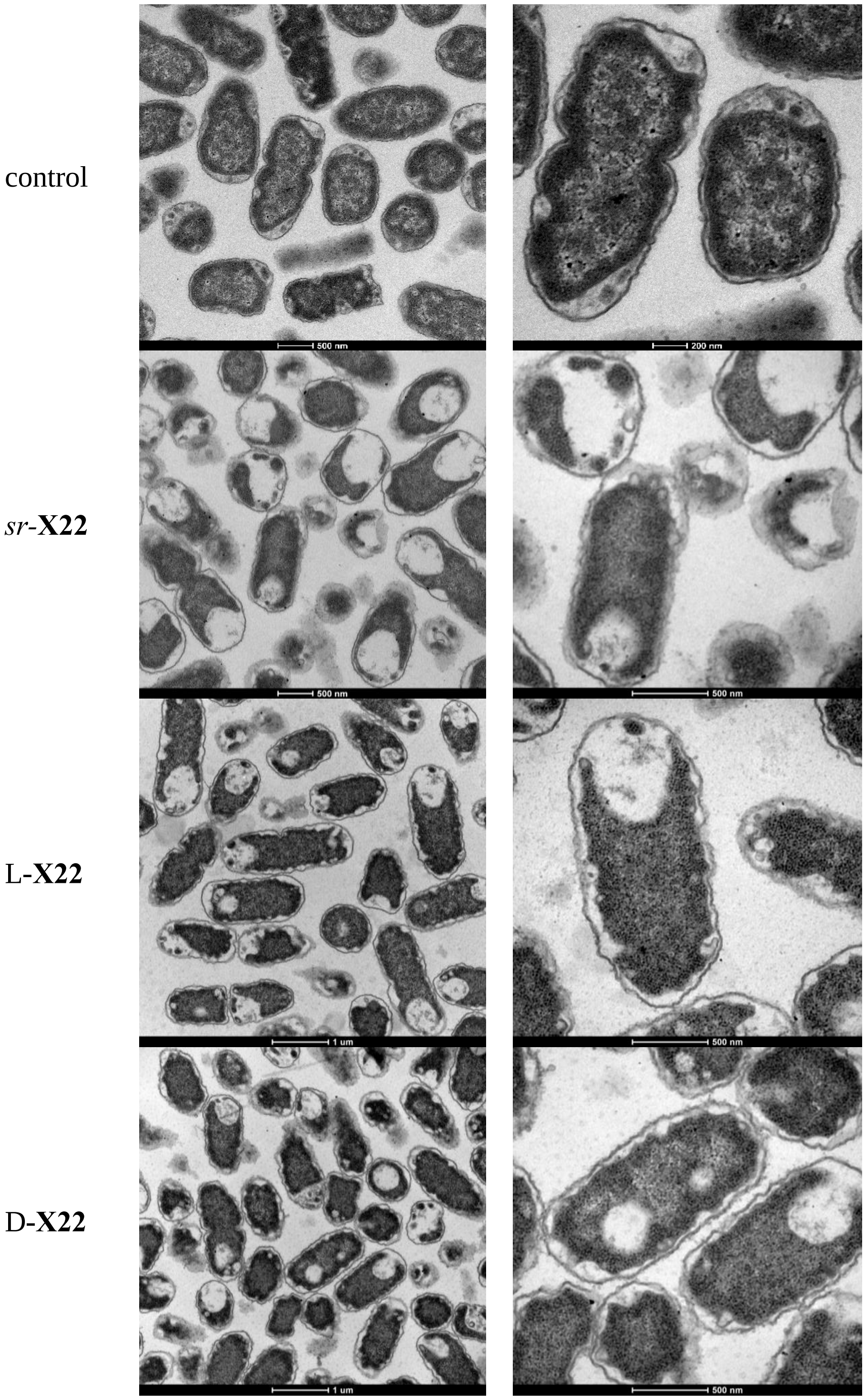

Figure S9. TEM images of *K. pneumoniae*, 2 h after treatment with *sr*-**X22** (64 µg/mL), L-**X22** (64 µg/mL), D-**X22** (64 µg/mL) and non-treated control in MH medium at pH 7.4.

sr-**aX18**

sr-**aX22**

sr-**T25**

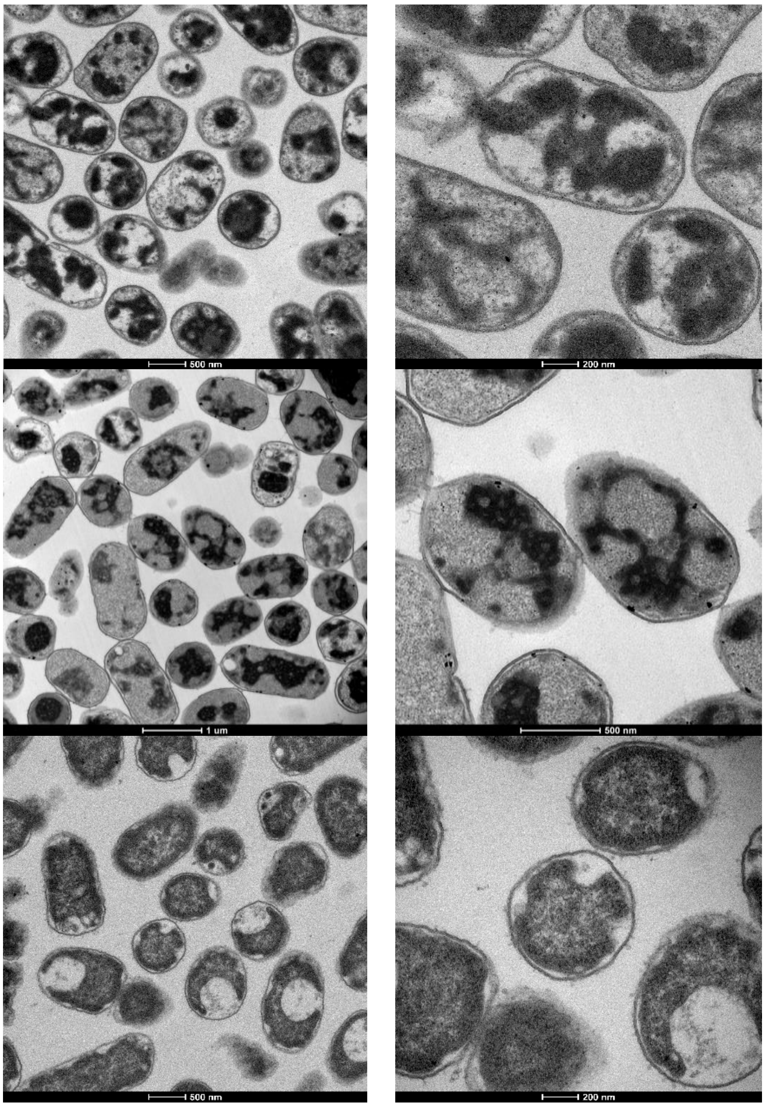

Figure S10. TEM images of *K. pneumoniae*, 2 h after treatment with *sr*-**aX18** (160 µg/mL), *sr*-**aX22** (64 µg/mL), and *sr*-**T25** (64 µg/mL) in MH medium at pH 7.4.

control

sr-**X22**

L-**X22**

D-**X22**

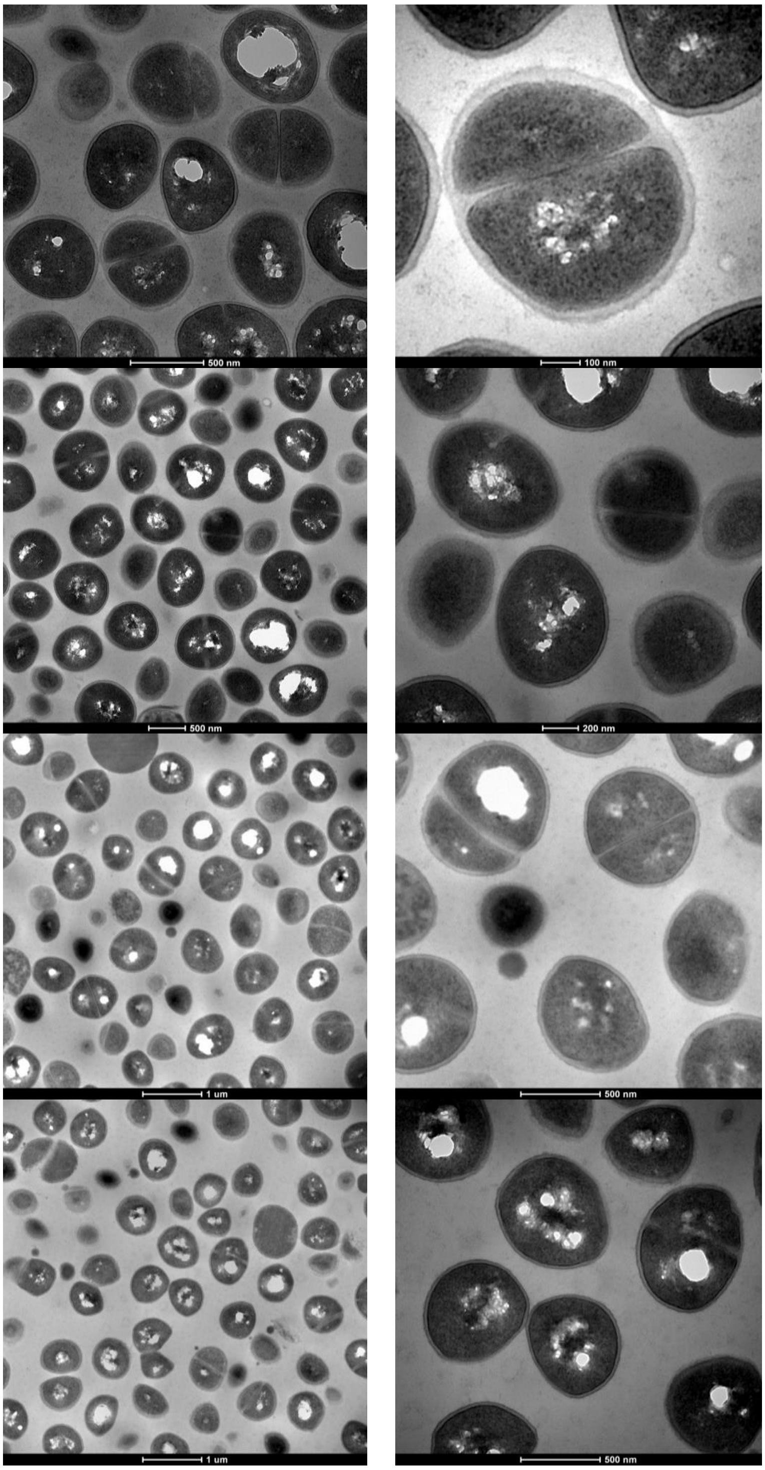

Figure S11. TEM images of MRSA, 2 h after treatment with *sr*-**X22** (64 µg/mL), L-**X22** (64 µg/mL), D-**X22** (64 µg/mL) and non-treated control in MH medium at pH 7.4.

sr-**aX18**

sr-**aX22**

sr-**T25**

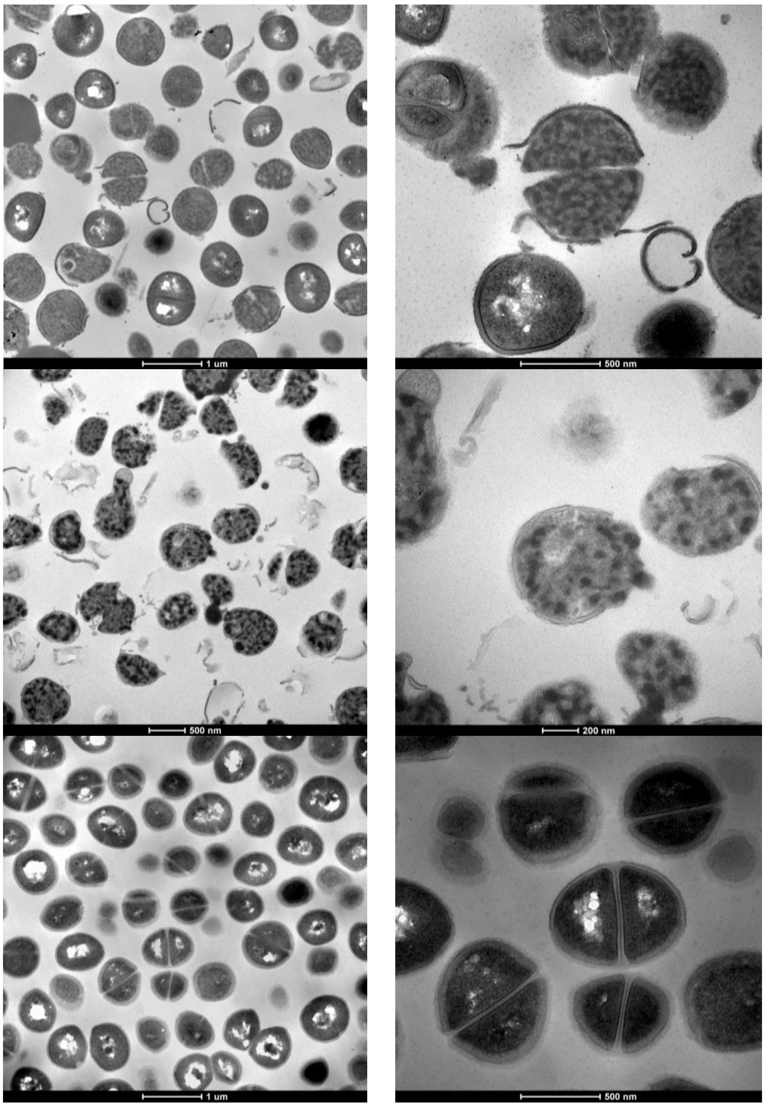

Figure S12. TEM images of MRSA, 2 h after treatment with *sr*-**aX18** (160 µg/mL), *sr*-**aX22** (160 µg/mL), and *sr*-**T25** (64 µg/mL) in MH medium at pH 7.4.

10. NPN Membrane Permeability Assay

A single colony of *Pseudomonas aeruginosa* PAO1 was grown overnight with shaking (150 rpm) in LB-broth (5 mL) at 37 °C. 100 µL of the overnight culture was regrown in 10 mL LB-broth with shaking (200 rpm) to the exponential phase OD_{600} = 1 (10^9 CFU/mL). Bacteria were washed once with HEPES buffer (5 mM HEPES, 5 mM glucose, pH 7.4) and diluted to OD_{600} = 0.5. Stock solutions of 1 mg/mL of the samples were prepared in sterilized milli-Q water and diluted to the beginning concentration of 128 µg/mL in 200 µL HEPES buffer containing 20 µM of the fluorescent probe 1-*N*-phenylnaphthylamine NPN (Sigma Aldrich). 200 µL peptide samples were added to the first well of 96-well plates (black wells, flat bottom, BRAND® GmbH Wertheim, Germany) and diluted serially by 1/2. 100 µL of the bacterial suspension in HEPES buffer (without NPN) were added to each well. In this case, the final OD of bacteria was 0.25, the final concentration of the desired compound 64 µg/mL and NPN 10 µM. The control wells are buffer containing NPN and bacterial suspension containing NPN in HEPES buffer. The plate was measured with a Tecan instrument Infinite M1000 within 5 min. The plate was enabled to shake for 5 sec before measurement. The excitation wavelength used was 340 ± 5 nm and emission wavelength 415 ± 5 nm. The assay was repeated at least three times. [6]

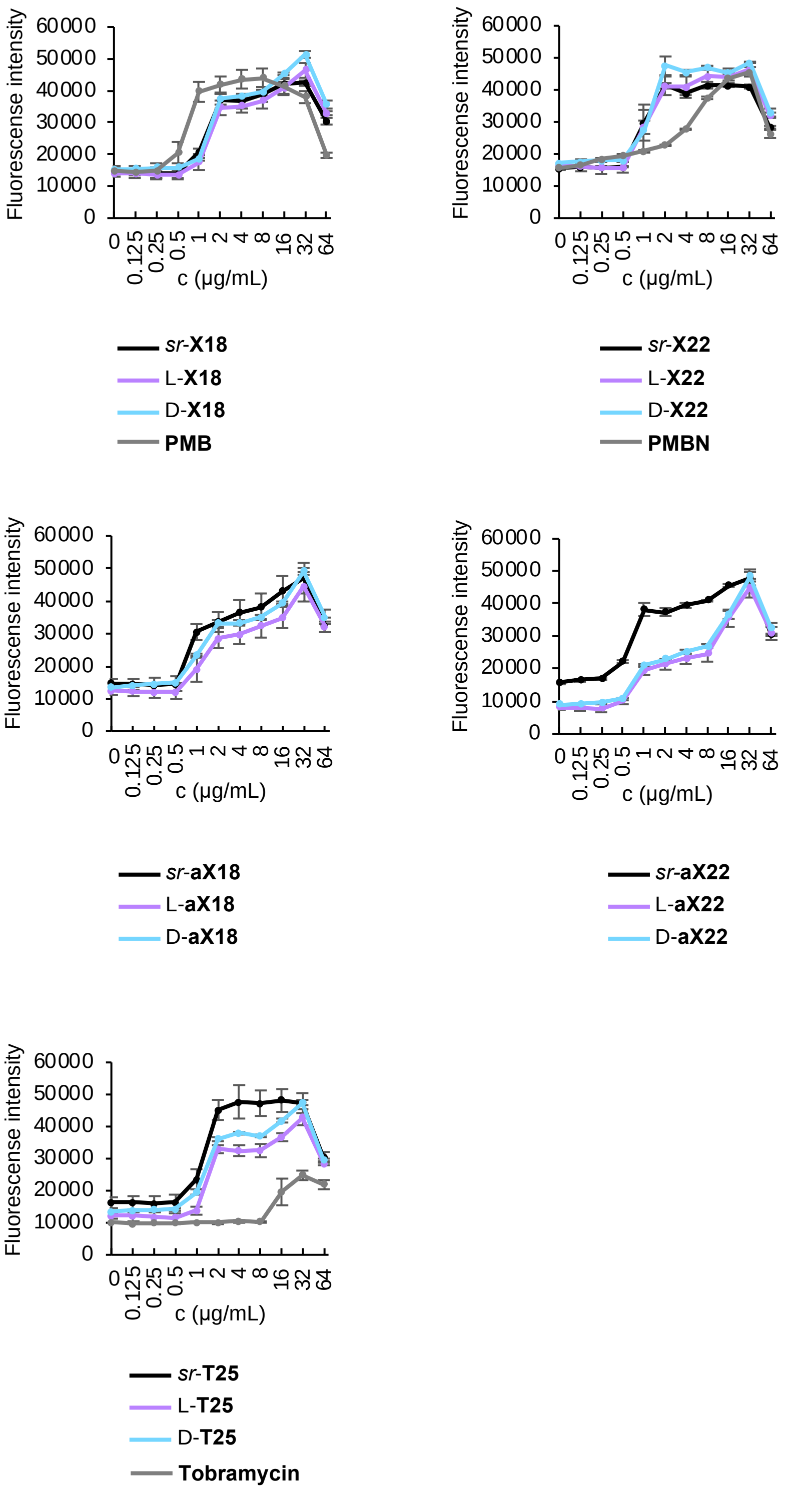

Figure S13. Membrane permeability changes of *P. aeruginosa* PAO1 induced by AMPDs by the NPN assay.

11. DiSC$_3$(5) Membrane Depolarization Assay

A single colony of *Pseudomonas aeruginosa* PAO1 was grown overnight with shaking (150 rpm) in LB-broth (5 mL) at 37 °C. 100 µL of the overnight culture was regrown in 10 mL LB-broth with shaking (200 rpm) to the exponential phase $OD_{600} = 1$ (10^9 CFU/mL). Bacteria were washed once with HEPES buffer (5 mM HEPES, 5 mM glucose, pH 7.4) and diluted to $OD_{600} = 0.4$. Stock solution of 10 mM of DiSC$_3$(5) was prepared in DMSO. Stock solutions of 1 mg/mL of the compounds were prepared in sterilized milli-Q water and diluted to the beginning concentration of 128 µg/mL in 200 µL HEPES buffer containing 20 µM of the fluorescent probe DiSC$_3$(5) 4 µM. The diluted samples were added to the first well of 96-well plates (black wells, flat bottom, BRAND® GmbH Wertheim, Germany) and diluted serially by 1/2. 100 µL of the bacterial suspension in HEPES buffer (without DiSC$_3$(5)) were added to each well. In this case, the final OD of bacteria was 0.2, the final concentration of peptide in the first column is 64 µg/mL and DiSC$_3$(5) 2 µM. The control wells are buffer containing DiSC$_3$(5) and bacterial suspension containing DiSC$_3$(5) in HEPES buffer. The plate was measured with a Tecan instrument Infinite M1000 within 5 min. The plate was enabled to shake for 5 sec before measurement. The excitation wavelength used was 610 nm ± 5 nm and the emission wavelength 660 nm ± 5 nm. The assay was repeated at least three times. [6]

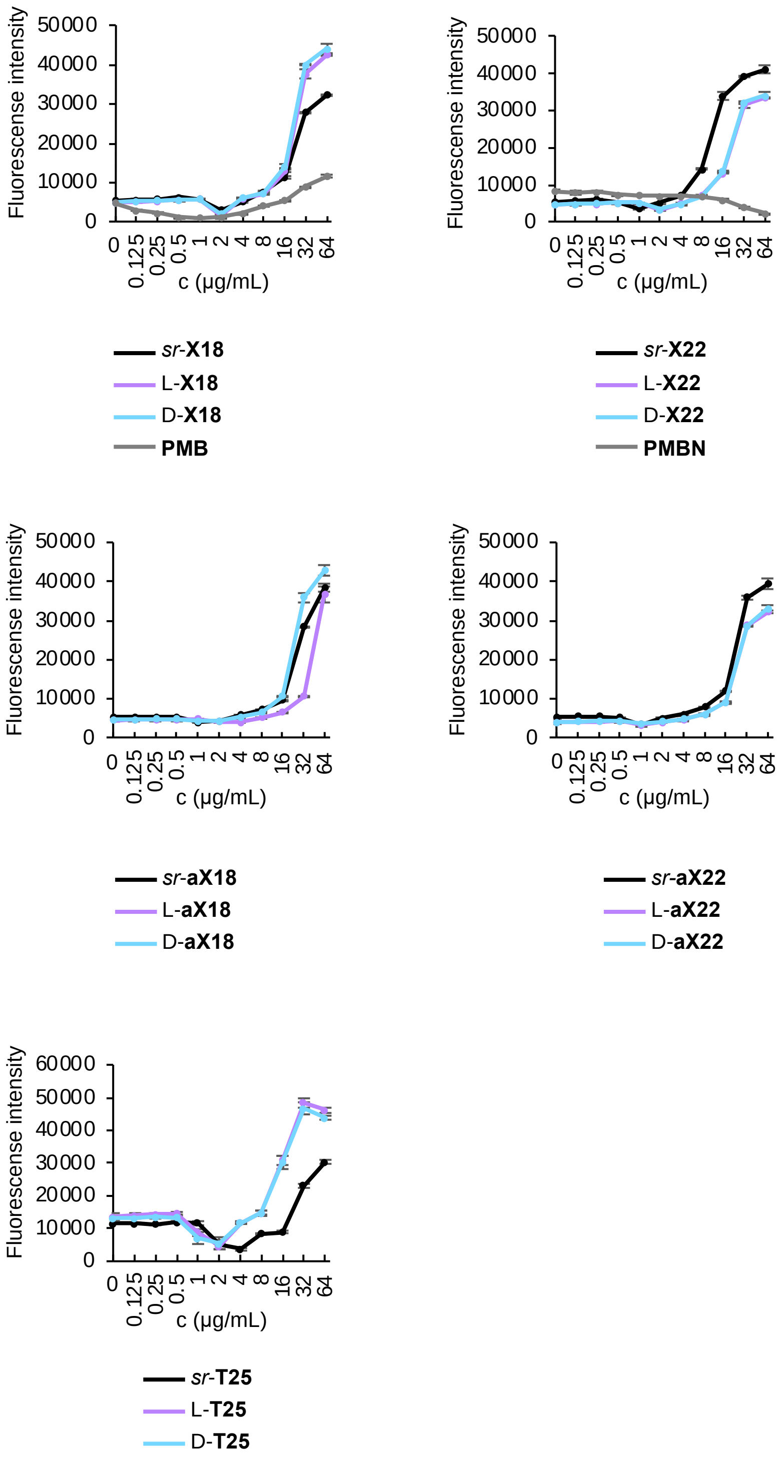

Figure S14. Inner membrane depolarization of *P. aeruginosa* PAO1 with AMPDs by the $DiSC_3(5)$ assay

12. Cell Membrane Permeability Assay

Propidium iodide (PI) was used as the fluorescent dye to evaluate integrity of bacterial membranes. A single colony of *Pseudomonas aeruginosa* PAO1 was grown overnight with shaking (150 rpm) in LB-broth (5 mL) at 37 °C. 100 µL of the overnight culture was regrown in 10 mL LB-broth to the exponential phase OD_{600} 1 (10^9 CFU/mL).

Bacteria was washed with MH and then re-suspended to a working concentration of OD_{600} = 1. AMPDs and polymyxin B (stock solution 1 mg/mL) were added at different final concentrations (16, 32 and 64 µg/mL). Samples were incubated with shaking (150 rpm) at 37°C for 20 minutes before samples were centrifuged at 14 000 rpm for 3 min. Bacteria sample was washed twice with PBS and stained with PI 5 µg/mL in PBS with shaking (150 rpm) at 37°C for 20 minutes. Bacteria sample was washed 5 times with PBS and the fluorescence intensity of bacteria suspensions was observed and recorded on a ImageStream® X Mark II Imaging Flow Cytometer with a 488 nm laser, bacteria cells with fluorescent intensity higher than 2×10^4 is defined as PI positive cells. Percentage of PI positive cells was calculated by IDEAS workstation.

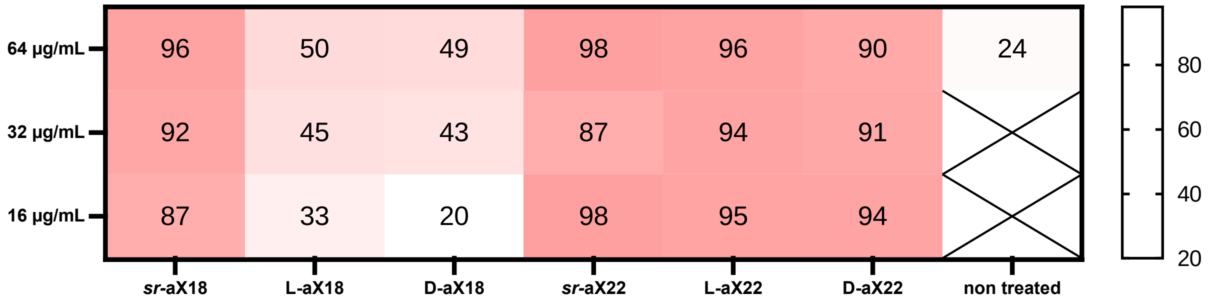

Figure S15. Percentage of PI positive *P. aeruginosa* PAO1 cells after treatment of AMPDs.

a)

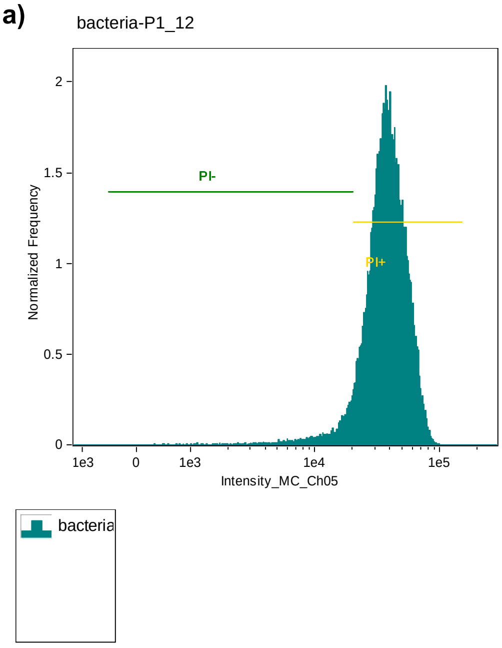

Intensity_MC_Ch05

Population	Count	%Total	%Gated
bacteria	34566	86.1	100
PI- & bacteria	2079	5.18	6.01
PI+ & bacteria	32487	80.9	94

b)

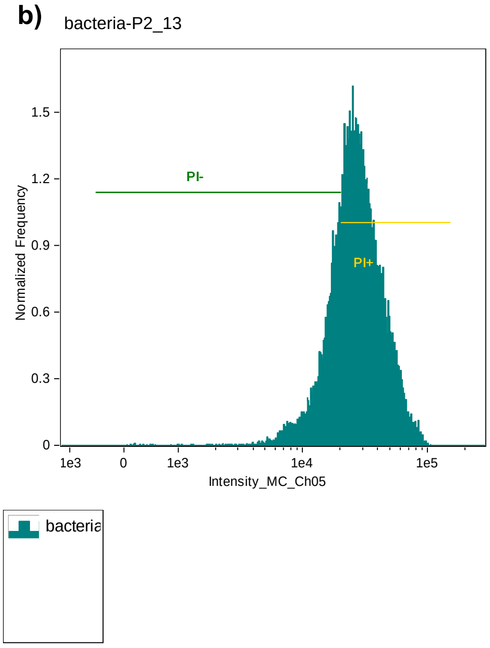

Intensity_MC_Ch05

Population	Count	%Total	%Gated
bacteria	29610	91.6	100
PI- & bacteria	7559	23.4	25.5
PI+ & bacteria	22051	68.2	74.5

c)

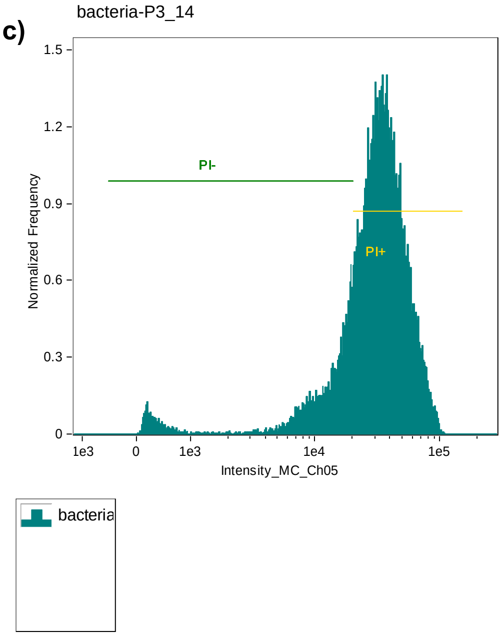

Intensity_MC_Ch05

Population	Count	%Total	%Gated
bacteria	29690	86	100
PI- & bacteria	5711	16.5	19.2
PI+ & bacteria	23979	69.5	80.8

Figure S16. FACS analysis of *P. aeruginosa* PAO1 cells after treatment of *sr*-**X18** at 64 (a), 32 (b) and 16 (c) μg/mL.

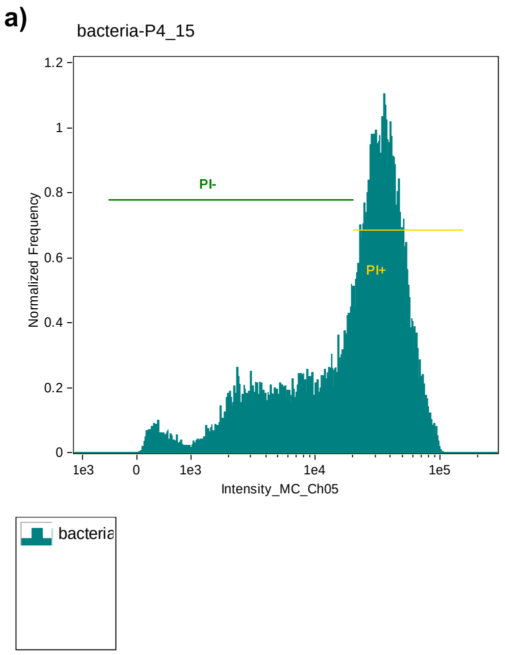

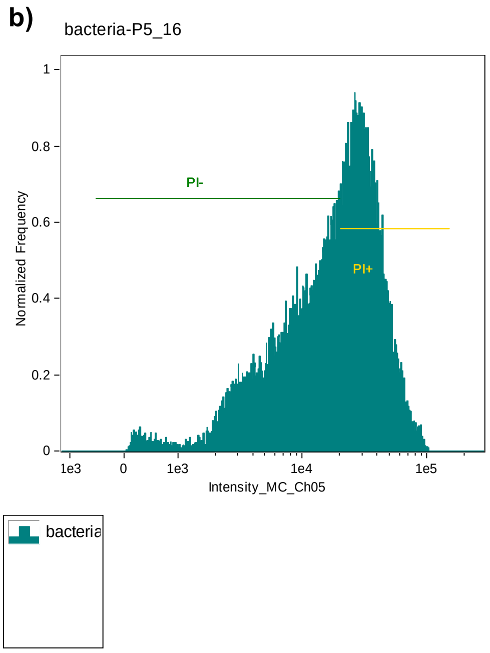

Intensity_MC_Ch05 (a)

Population	Count	%Total	%Gated
bacteria	30092	72.3	100
PI- & bacteria	11621	27.9	38.6
PI+ & bacteria	18471	44.4	61.4

Intensity_MC_Ch05 (b)

Population	Count	%Total	%Gated
bacteria	28860	73.1	100
PI- & bacteria	14429	36.6	50
PI+ & bacteria	14431	36.6	50

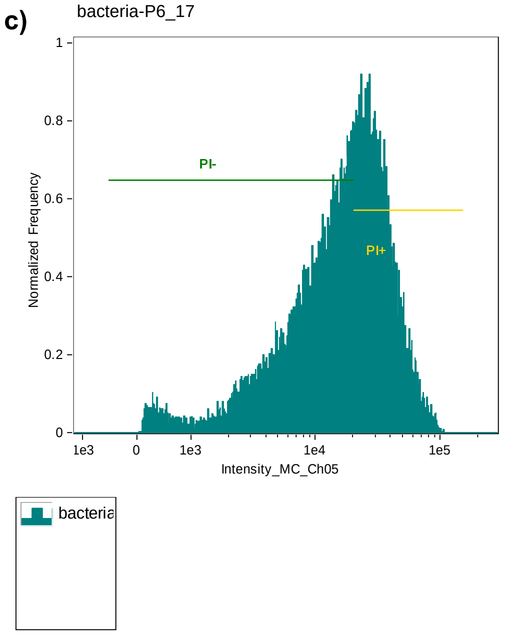

Intensity_MC_Ch05

Population	Count	%Total	%Gated
bacteria	27675	74.2	100
PI- & bacteria	15096	40.5	54.5
PI+ & bacteria	12579	33.7	45.5

Figure S17. FACS analysis of *P. aeruginosa* PAO1 cells after treatment of L-**X18** at 64 (a), 32 (b) and 16 (c) μg/mL.

a)

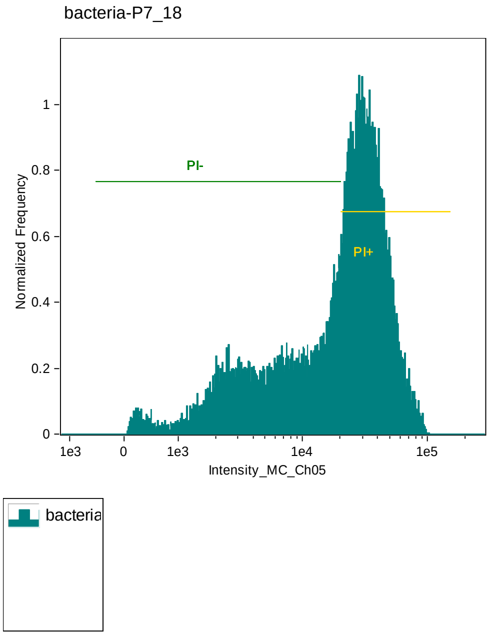

Intensity_MC_Ch05

Population	Count	%Total	%Gated
**bacteria**	29132	69.9	100
**PI- & bacteria**	12084	29	41.5
**PI+ & bacteria**	17048	40.9	58.5

b)

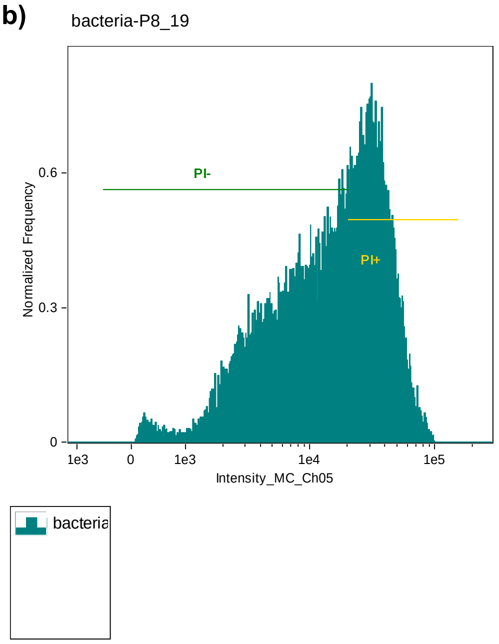

Intensity_MC_Ch05

Population	Count	%Total	%Gated
**bacteria**	28828	71.6	100
**PI- & bacteria**	16266	40.4	56.4
**PI+ & bacteria**	12562	31.2	43.6

c)

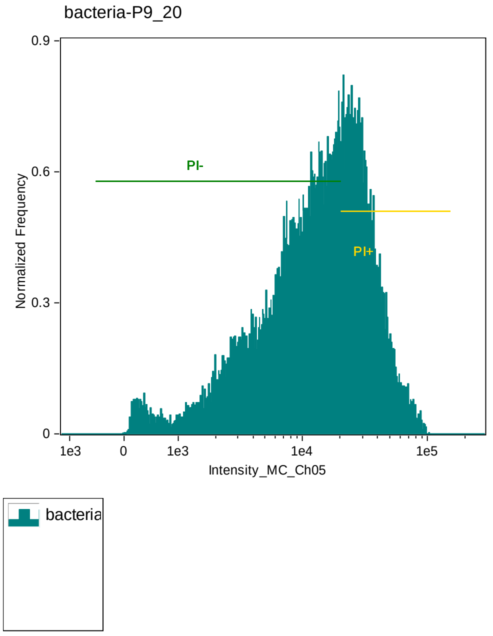

Intensity_MC_Ch05

Population	Count	%Total	%Gated
**bacteria**	28055	71.5	100
**PI- & bacteria**	17678	45.1	63
**PI+ & bacteria**	10377	26.5	37

Figure S18. FACS analysis of *P. aeruginosa* PAO1 cells after treatment of D-**X18** at 64 (a), 32 (b) and 16 (c) µg/mL.

a)

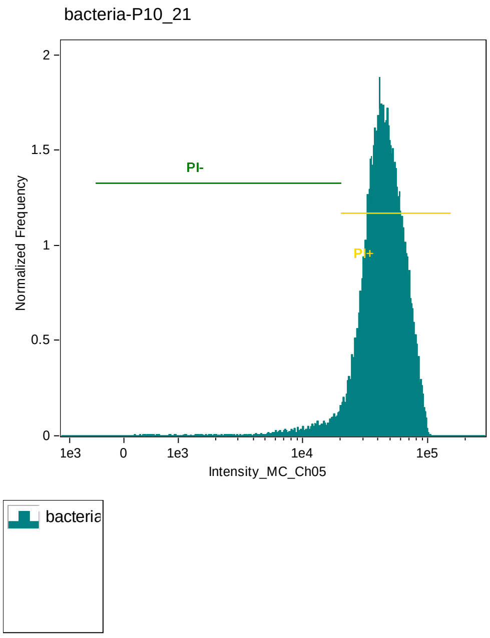

Intensity_MC_Ch05

Population	Count	%Total	%Gated
bacteria	33798	83.2	100
PI- & bacteria	1430	3.52	4.23
PI+ & bacteria	32368	79.7	95.8

b)

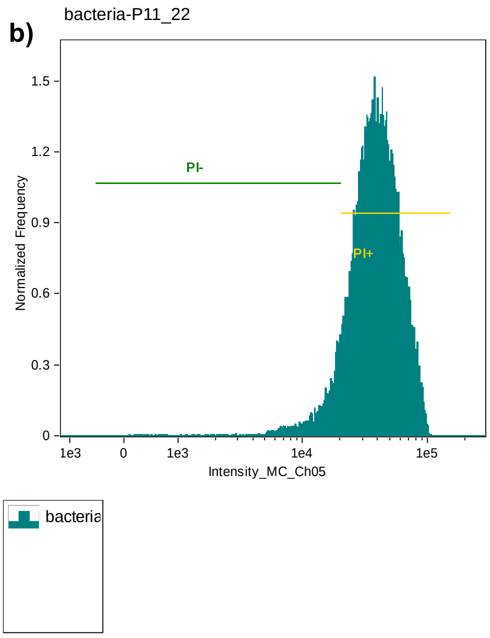

Intensity_MC_Ch05

Population	Count	%Total	%Gated
bacteria	31757	87.4	100
PI- & bacteria	2710	7.46	8.53
PI+ & bacteria	29047	80	91.5

c)

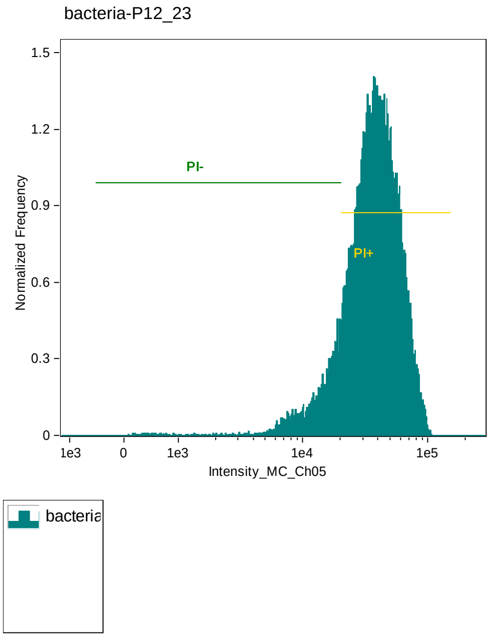

Intensity_MC_Ch05

Population	Count	%Total	%Gated
bacteria	30811	87	100
PI- & bacteria	3920	11.1	12.7
PI+ & bacteria	26891	75.9	87.3

Figure S19. FACS analysis of *P. aeruginosa* PAO1 cells after treatment of *sr*-**aX18** at 64 (a), 32 (b) and 16 (c) µg/mL.

a)

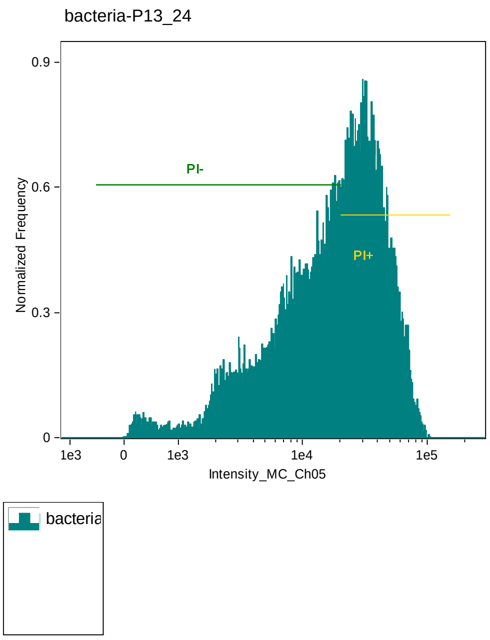

Intensity_MC_Ch05

Population	Count	%Total	%Gated
bacteria	27195	65.9	100
PI- & bacteria	13549	32.9	49.8
PI+ & bacteria	13646	33.1	50.2

b)

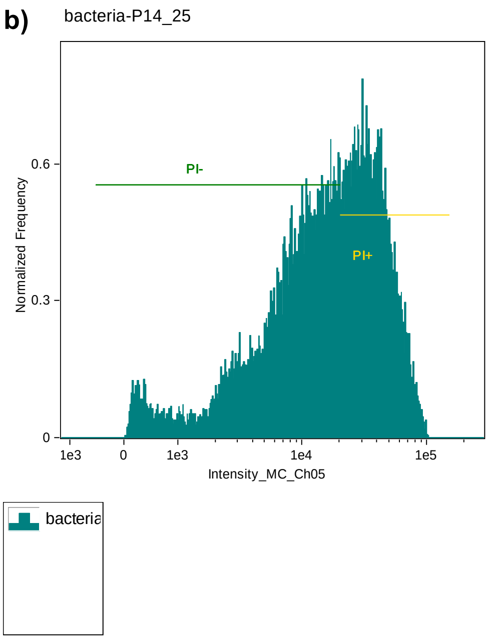

Intensity_MC_Ch05

Population	Count	%Total	%Gated
bacteria	26435	63.1	100
PI- & bacteria	14604	34.9	55.2
PI+ & bacteria	11831	28.2	44.8

c)

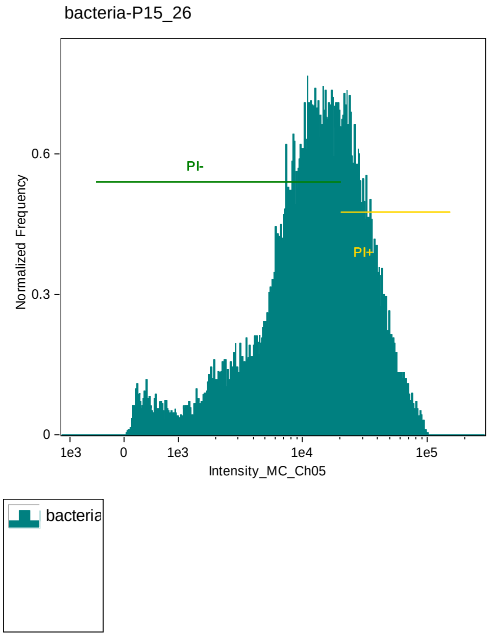

Intensity_MC_Ch05

Population	Count	%Total	%Gated
bacteria	25650	65.8	100
PI- & bacteria	17151	44	66.9
PI+ & bacteria	8499	21.8	33.1

Figure S20. FACS analysis of *P. aeruginosa* PAO1 cells after treatment of L-**aX18** at 64 (a), 32 (b) and 16 (c) µg/mL.

a)

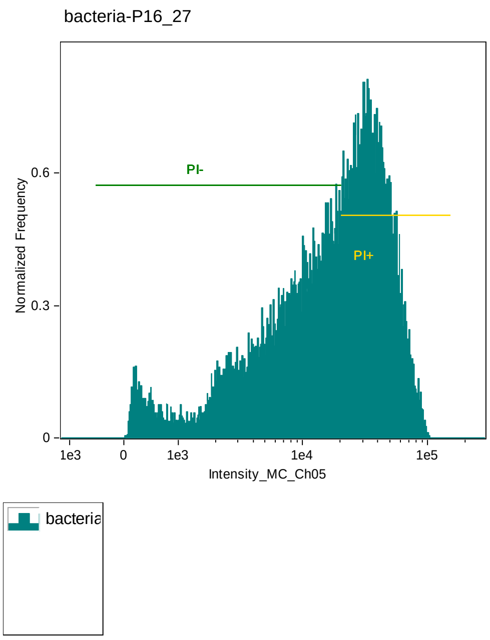

Intensity_MC_Ch05

Population	Count	%Total	%Gated
bacteria	26928	63.8	100
PI- & bacteria	13620	32.3	50.6
PI+ & bacteria	13308	31.5	49.4

b)

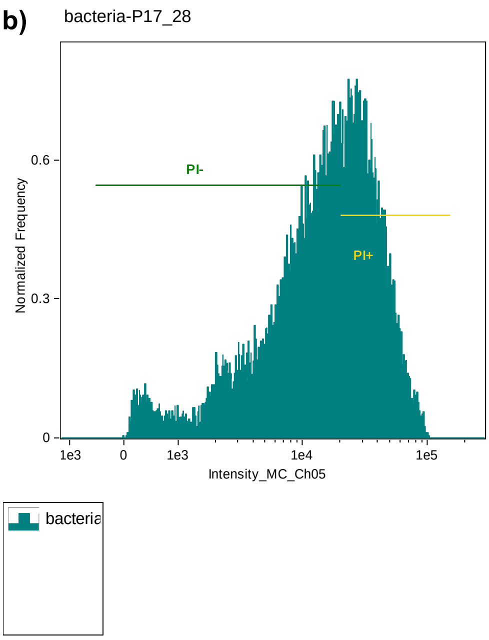

Intensity_MC_Ch05

Population	Count	%Total	%Gated
bacteria	27217	68	100
PI- & bacteria	15485	38.7	56.9
PI+ & bacteria	11732	29.3	43.1

c)

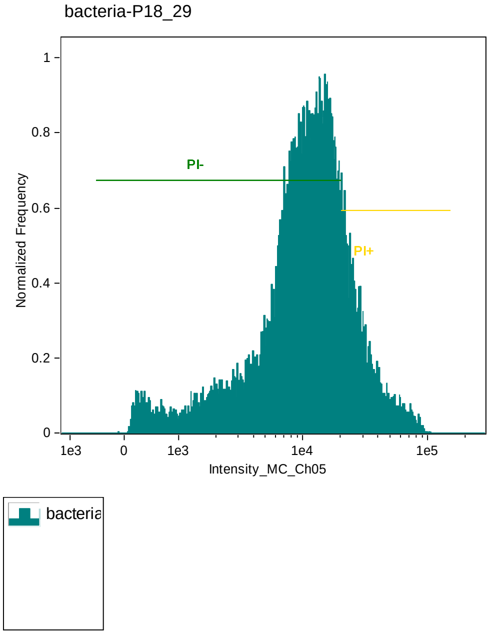

Intensity_MC_Ch05

Population	Count	%Total	%Gated
bacteria	24646	66	100
PI- & bacteria	19851	53.1	80.5
PI+ & bacteria	4795	12.8	19.5

Figure S21. FACS analysis of *P. aeruginosa* PAO1 cells after treatment of D-**aX18** at 64 (a), 32 (b) and 16 (c) µg/mL.

a)

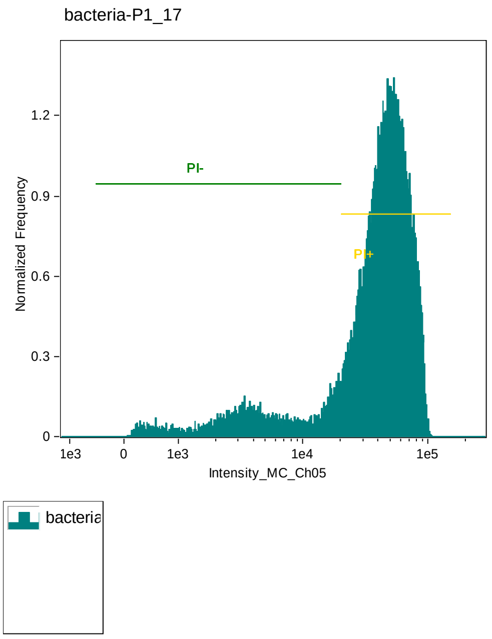

Intensity_MC_Ch05

Population	Count	%Total	%Gated
**bacteria**	35425	72.2	100
**PI- & bacteria**	5946	12.1	16.8
**PI+ & bacteria**	29479	60.1	83.2

b)

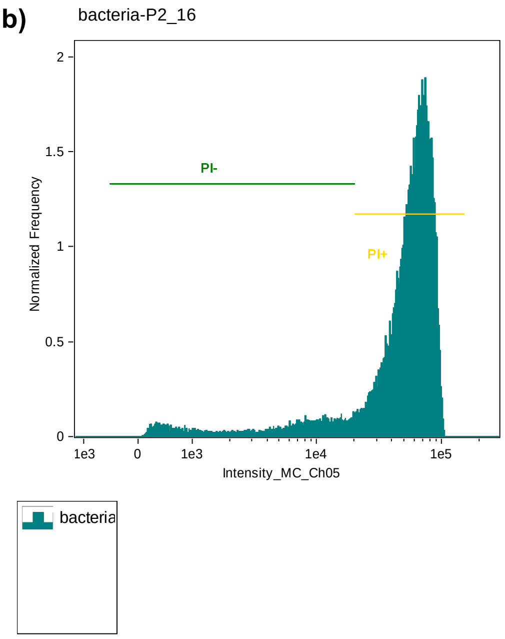

Intensity_MC_Ch05

Population	Count	%Total	%Gated
**bacteria**	45964	65.1	100
**PI- & bacteria**	5747	8.14	12.5
**PI+ & bacteria**	40217	57	87.5

c)

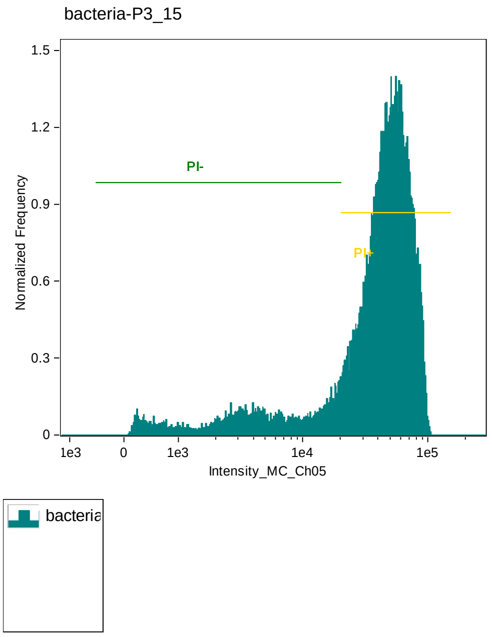

Intensity_MC_Ch05

Population	Count	%Total	%Gated
**bacteria**	36153	76.3	100
**PI- & bacteria**	6098	12.9	16.9
**PI+ & bacteria**	30055	63.4	83.1

Figure S22. FACS analysis of *P. aeruginosa* PAO1 cells after treatment of *sr*-**X22** at 64 (a), 32 (b) and 16 (c) µg/mL.

a)

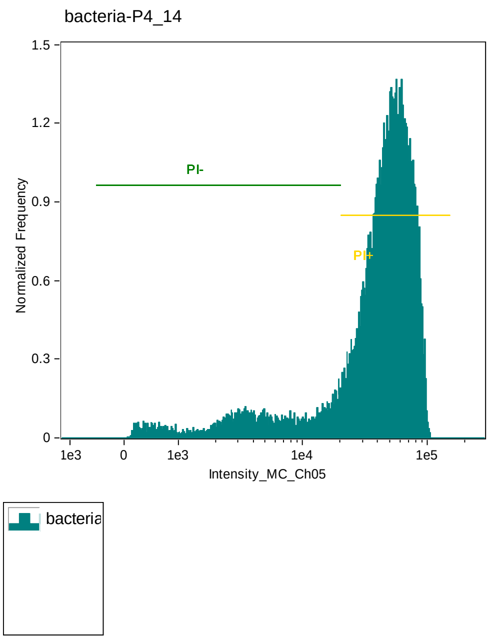

Intensity_MC_Ch05

Population	Count	%Total	%Gated
bacteria	37100	73	100
PI- & bacteria	6033	11.9	16.3
PI+ & bacteria	31067	61.1	83.7

b)

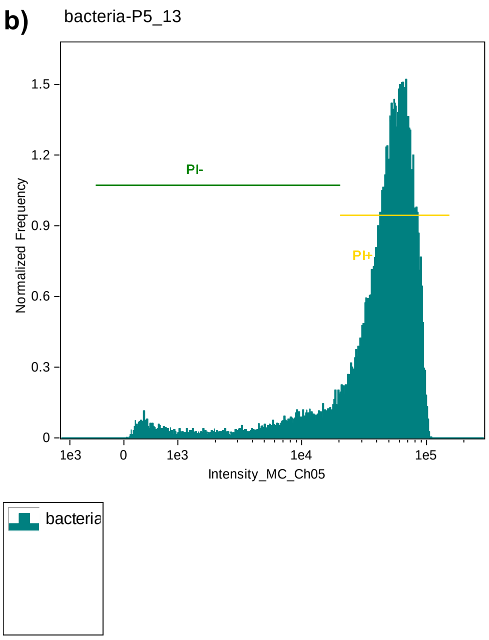

Intensity_MC_Ch05

Population	Count	%Total	%Gated
bacteria	39311	74.6	100
PI- & bacteria	5401	10.3	13.7
PI+ & bacteria	33910	64.4	86.3

c)

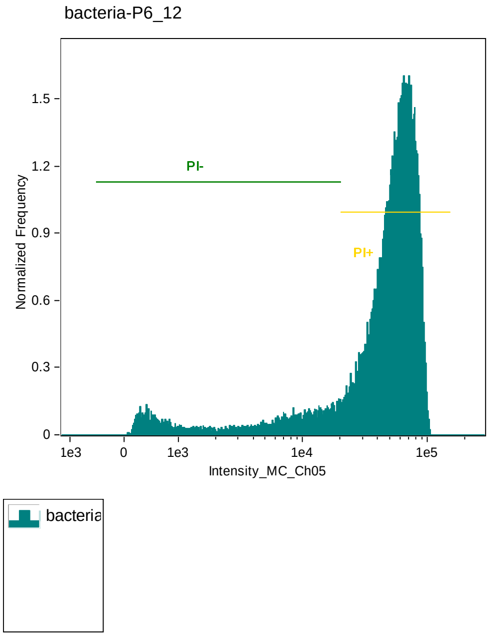

Intensity_MC_Ch05

Population	Count	%Total	%Gated
bacteria	41882	68.2	100
PI- & bacteria	6357	10.4	15.2
PI+ & bacteria	35525	57.9	84.8

Figure S23. FACS analysis of *P. aeruginosa* PAO1 cells after treatment of L-**X22** at 64 (a), 32 (b) and 16 (c) μg/mL.

a)

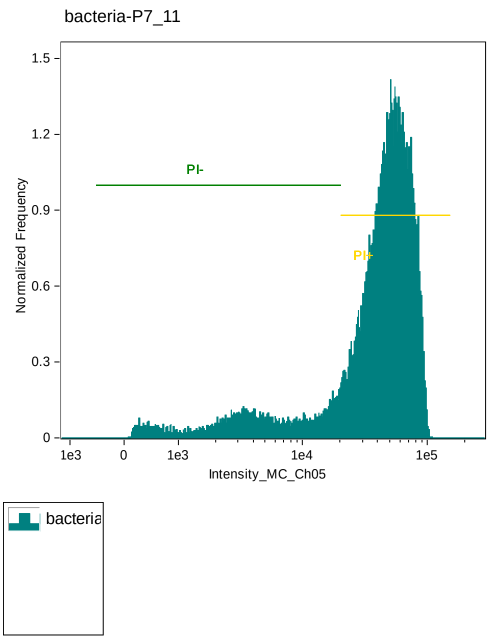

Intensity_MC_Ch05

Population	Count	%Total	%Gated
bacteria	37086	73.8	100
PI- & bacteria	6155	12.3	16.6
PI+ & bacteria	30931	61.6	83.4

b)

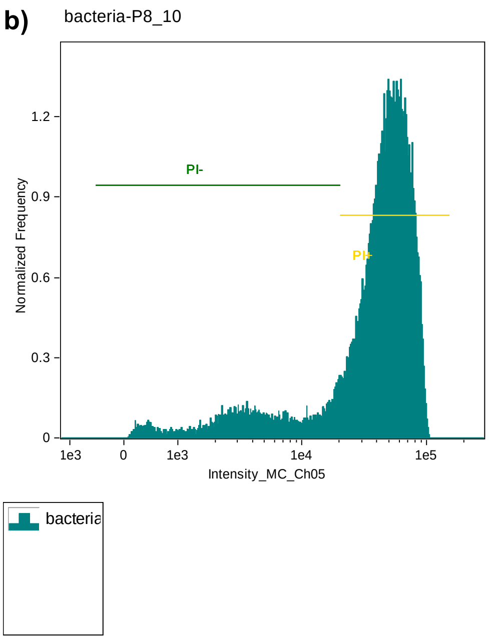

Intensity_MC_Ch05

Population	Count	%Total	%Gated
bacteria	37093	71.8	100
PI- & bacteria	6351	12.3	17.1
PI+ & bacteria	30742	59.5	82.9

c)

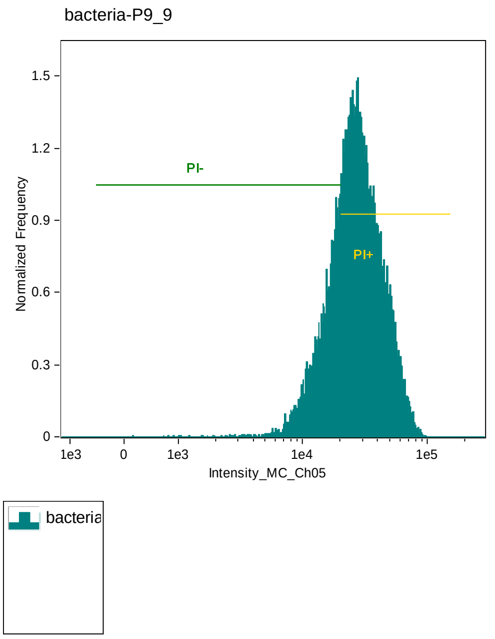

Intensity_MC_Ch05

Population	Count	%Total	%Gated
bacteria	30444	84.6	100
PI- & bacteria	8200	22.8	26.9
PI+ & bacteria	22244	61.8	73.1

Figure S24. FACS analysis of *P. aeruginosa* PAO1 cells after treatment of D-**X22** at 64 (a), 32 (b) and 16 (c) μg/mL.

a)

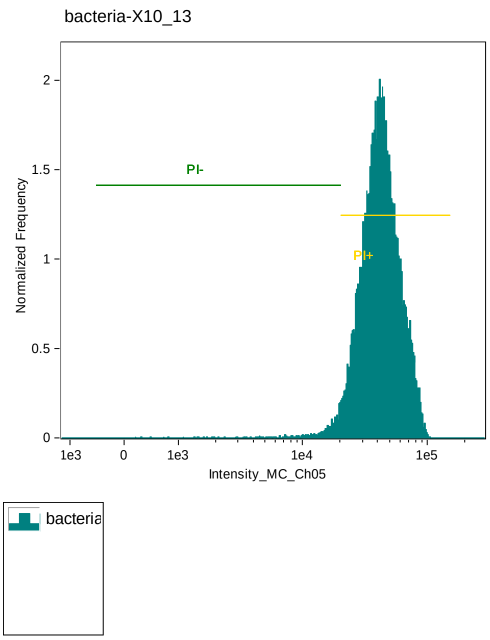

Intensity_MC_Ch05

Population	Count	%Total	%Gated
bacteria	33279	81.5	100
PI- & bacteria	838	2.05	2.52
PI+ & bacteria	32441	79.4	97.5

b)

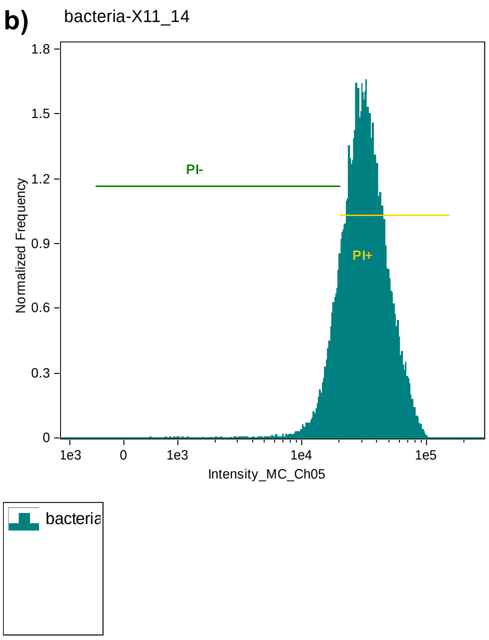

Intensity_MC_Ch05

Population	Count	%Total	%Gated
bacteria	30149	83.5	100
PI- & bacteria	4049	11.2	13.4
PI+ & bacteria	26100	72.3	86.6

c)

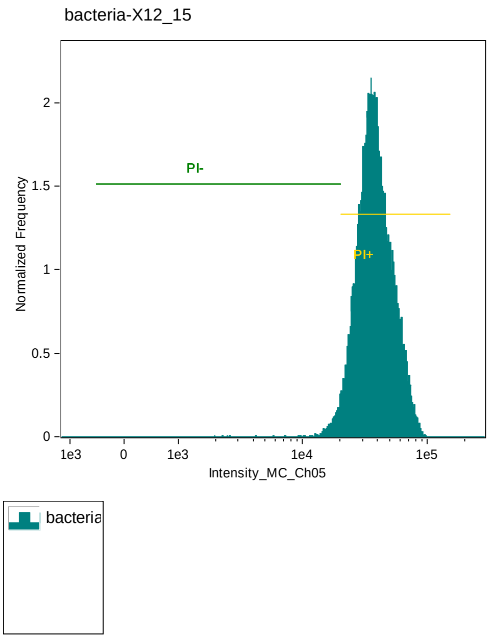

Intensity_MC_Ch05

Population	Count	%Total	%Gated
bacteria	34129	79.9	100
PI- & bacteria	826	1.93	2.42
PI+ & bacteria	33303	77.9	97.6

Figure S25. FACS analysis of *P. aeruginosa* PAO1 cells after treatment of *sr*-**aX22** at 64 (a), 32 (b) and 16 (c) μg/mL.

a)

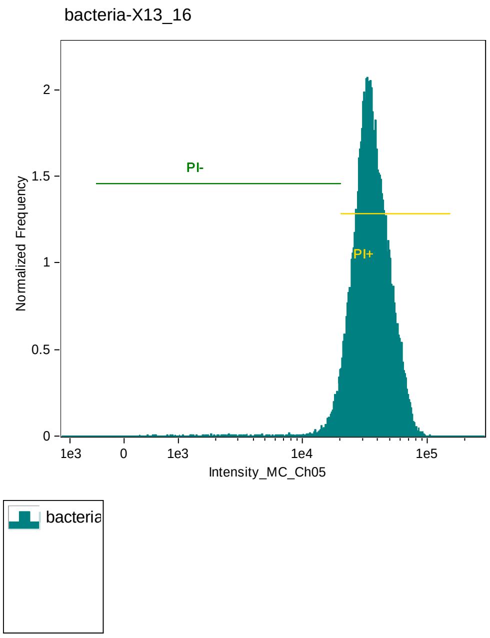

Intensity_MC_Ch05

Population	Count	%Total	%Gated
bacteria	32138	88.4	100
PI- & bacteria	1297	3.57	4.04
PI+ & bacteria	30841	84.8	96

b)

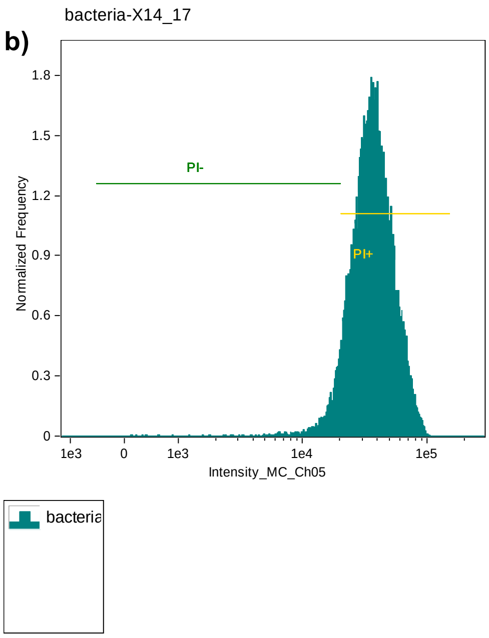

Intensity_MC_Ch05

Population	Count	%Total	%Gated
bacteria	31478	89.5	100
PI- & bacteria	1948	5.54	6.19
PI+ & bacteria	29530	83.9	93.8

c)

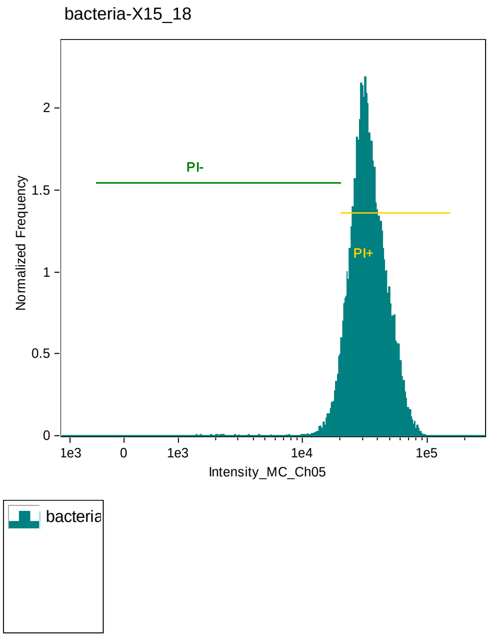

Intensity_MC_Ch05

Population	Count	%Total	%Gated
bacteria	32582	83.7	100
PI- & bacteria	1669	4.29	5.12
PI+ & bacteria	30913	79.5	94.9

Figure S26. FACS analysis of *P. aeruginosa* PAO1 cells after treatment of L-**aX22** at 64 (a), 32 (b) and 16 (c) μg/mL.

a)

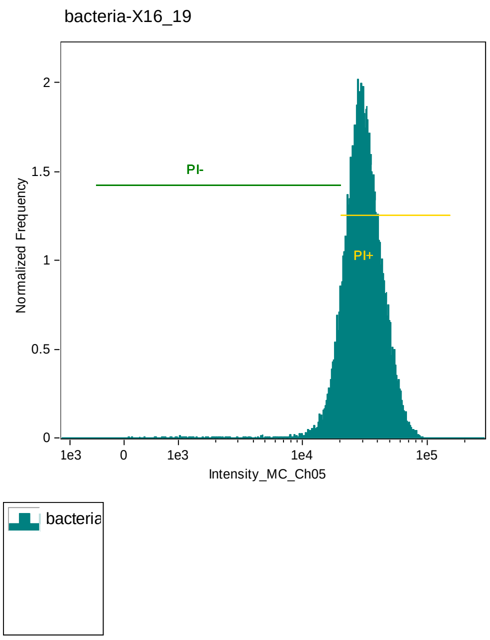

Intensity_MC_Ch05

Population	Count	%Total	%Gated
bacteria	30597	92.8	100
PI- & bacteria	3056	9.27	9.99
PI+ & bacteria	27541	83.5	90

b)

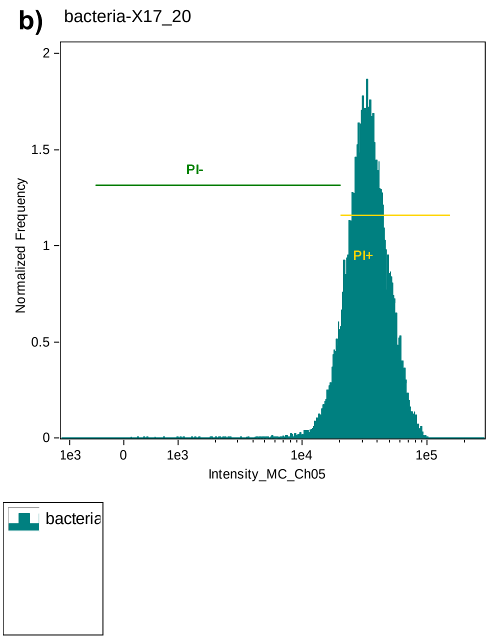

Intensity_MC_Ch05

Population	Count	%Total	%Gated
bacteria	30809	90.1	100
PI- & bacteria	2788	8.15	9.05
PI+ & bacteria	28021	81.9	91

c)

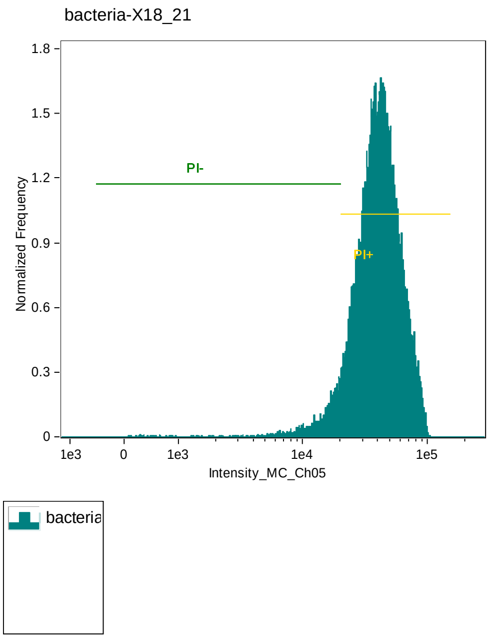

Intensity_MC_Ch05

Population	Count	%Total	%Gated
bacteria	32332	80.5	100
PI- & bacteria	2072	5.16	6.41
PI+ & bacteria	30260	75.3	93.6

Figure S27. FACS analysis of *P. aeruginosa* PAO1 cells after treatment of D-**aX22** at 64 (a), 32 (b) and 16 (c) µg/mL.

a)

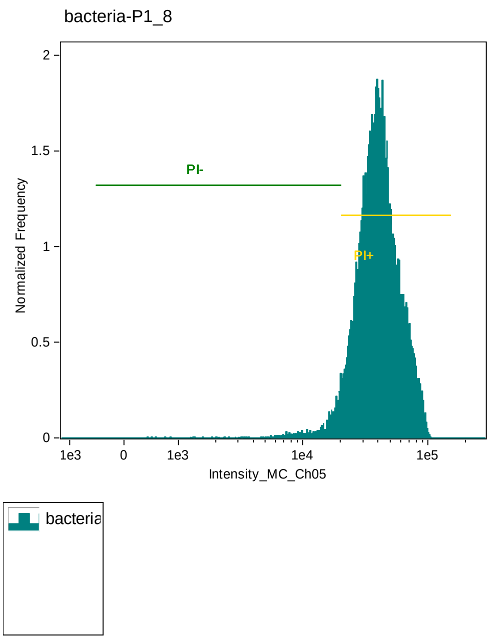

Intensity_MC_Ch05

Population	Count	%Total	%Gated
bacteria	32672	86.4	100
PI- & bacteria	1361	3.6	4.17
PI+ & bacteria	31311	82.8	95.8

b)

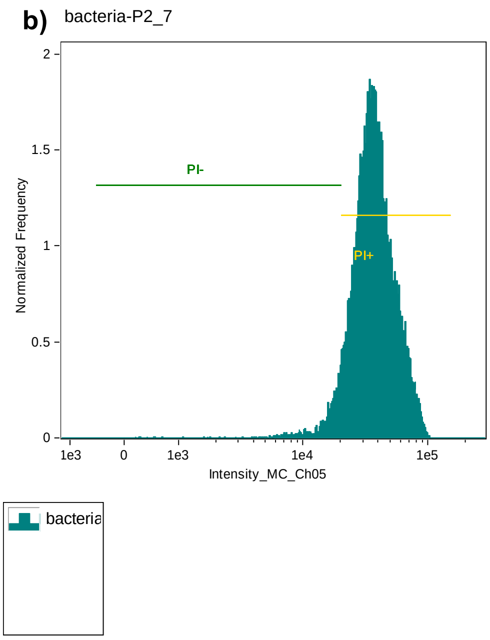

Intensity_MC_Ch05

Population	Count	%Total	%Gated
bacteria	31735	90.3	100
PI- & bacteria	1800	5.12	5.67
PI+ & bacteria	29935	85.2	94.3

c)

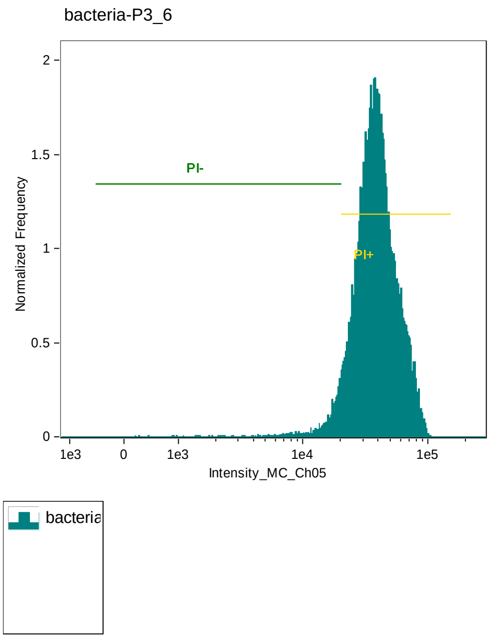

Intensity_MC_Ch05

Population	Count	%Total	%Gated
bacteria	32354	83.7	100
PI- & bacteria	1436	3.71	4.44
PI+ & bacteria	30918	80	95.6

Figure S28. FACS analysis of *P. aeruginosa* PAO1 cells after treatment of **PMB** at 64 (a), 32 (b) and 16 (c) µg/mL.

a)

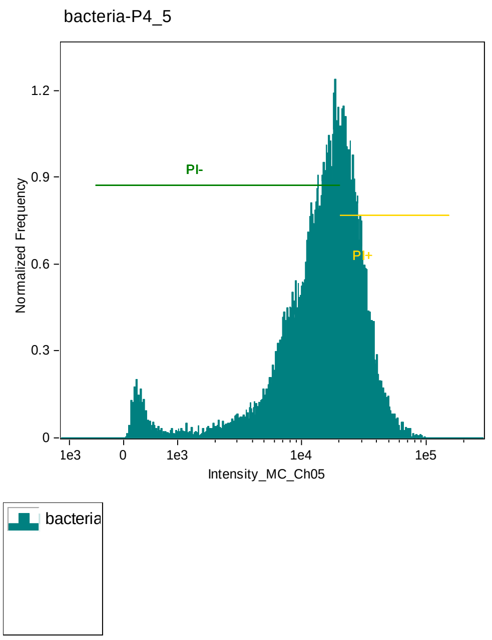

Intensity_MC_Ch05

Population	Count	%Total	%Gated
bacteria	26223	75.4	100
PI- & bacteria	16544	47.6	63.1
PI+ & bacteria	9679	27.8	36.9

b)

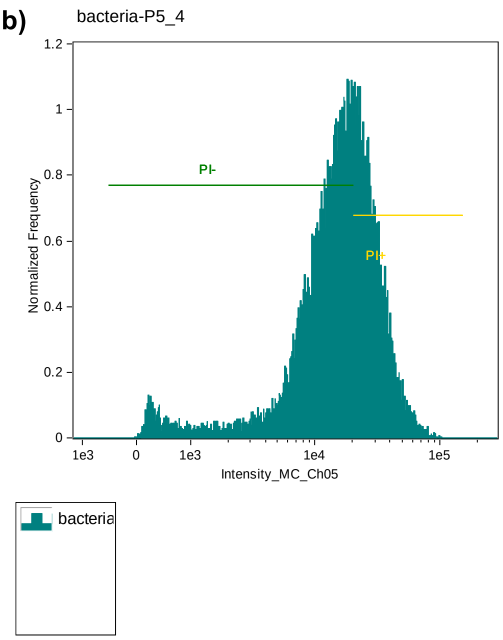

Intensity_MC_Ch05

Population	Count	%Total	%Gated
bacteria	26862	78.5	100
PI- & bacteria	16586	48.5	61.7
PI+ & bacteria	10276	30	38.3

c)

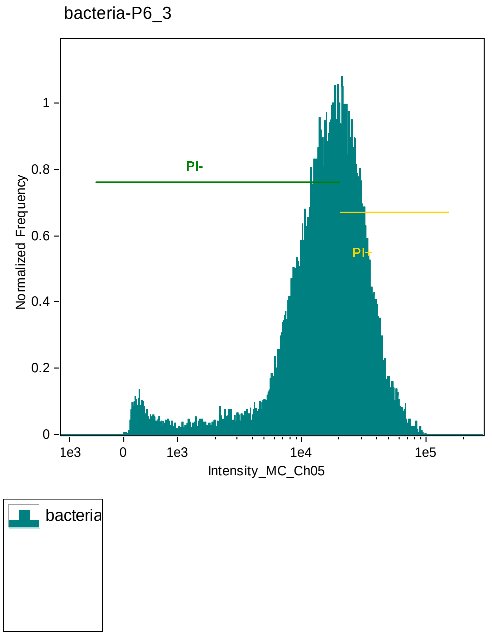

Intensity_MC_Ch05

Population	Count	%Total	%Gated
bacteria	26956	80.2	100
PI- & bacteria	16126	48	59.8
PI+ & bacteria	10830	32.2	40.2

Figure S29. FACS analysis of *P. aeruginosa* PAO1 cells after treatment of nona **PMB** at 64 (a), 32 (b) and 16 (c) µg/mL.

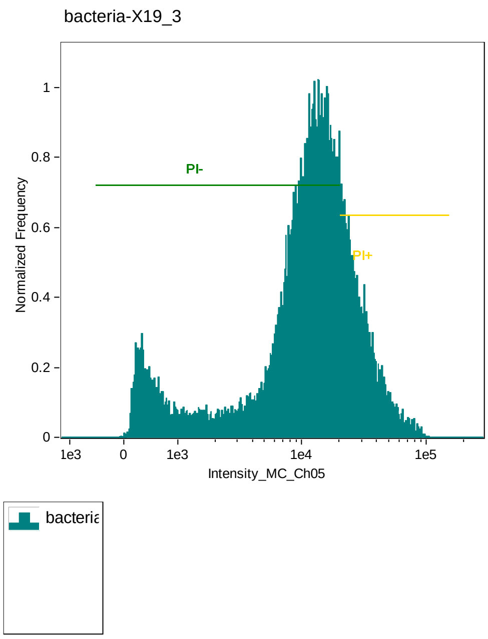

Intensity_MC_Ch05

Population	Count	%Total	%Gated
**bacteria**	25949	76.2	100
**PI- & bacteria**	19643	57.7	75.7
**PI+ & bacteria**	6306	18.5	24.3

Figure S30. FACS analysis of *P. aeruginosa* PAO1 cells with no treatment of AMPD.

13. Circular dichroism (CD) spectroscopic measurements

CD spectra were recorded using a Jasco J-715 spectrometer equipped with a PFD-350S temperature controller and a PS-150J power supply. All experiments were measured using a Hellma Suprasil 100QS 0.1 cm cuvette. Stock solution (1.00 mg/mL) of dendrimers were freshly prepared in mQ-H_2O. Stock solution (50 mM) of dodecylphosphocholine (DPC, Avanti Polar Lipids, Inc., USA) was prepared in mQ-H2O. For the measurement, the peptides were diluted to 0.100 mg/mL with 10 mM phosphate buffer at pH 7.4. A final concentration of 5 mM DPC or 20% (v/v) 2,2,2-trifluoroethanol (TFE, Sigma, Steinheim, Germany) was added when specified. The range of measurement was 185-260 nm, scan rate was 20 nm/min, pitch 0.5 nm, response 16 sec. and band 1.0 nm. The nitrogen flow was kept above 10 L/min. The baseline was recorded under the same conditions and subtracted manually. The cuvettes were washed with 1M HCl, mQ-H_2O and phosphate buffer before each measurement. [2][5]

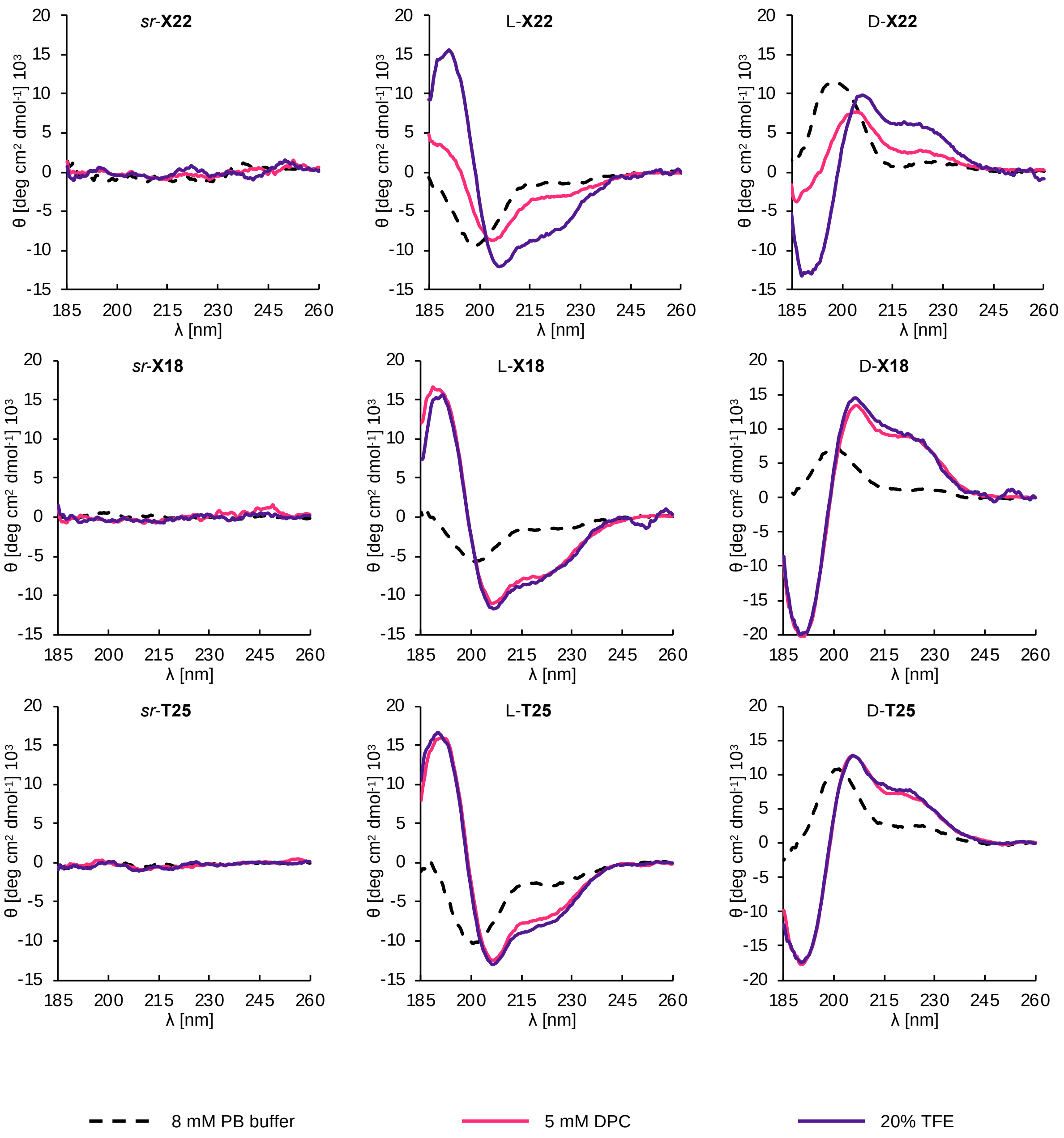

Figure S31. CD spectrum of dendrimers (0.100 mg/mL) in aq. phosphate buffer (8 mM) at different pH upon addition of DPC or TFE.

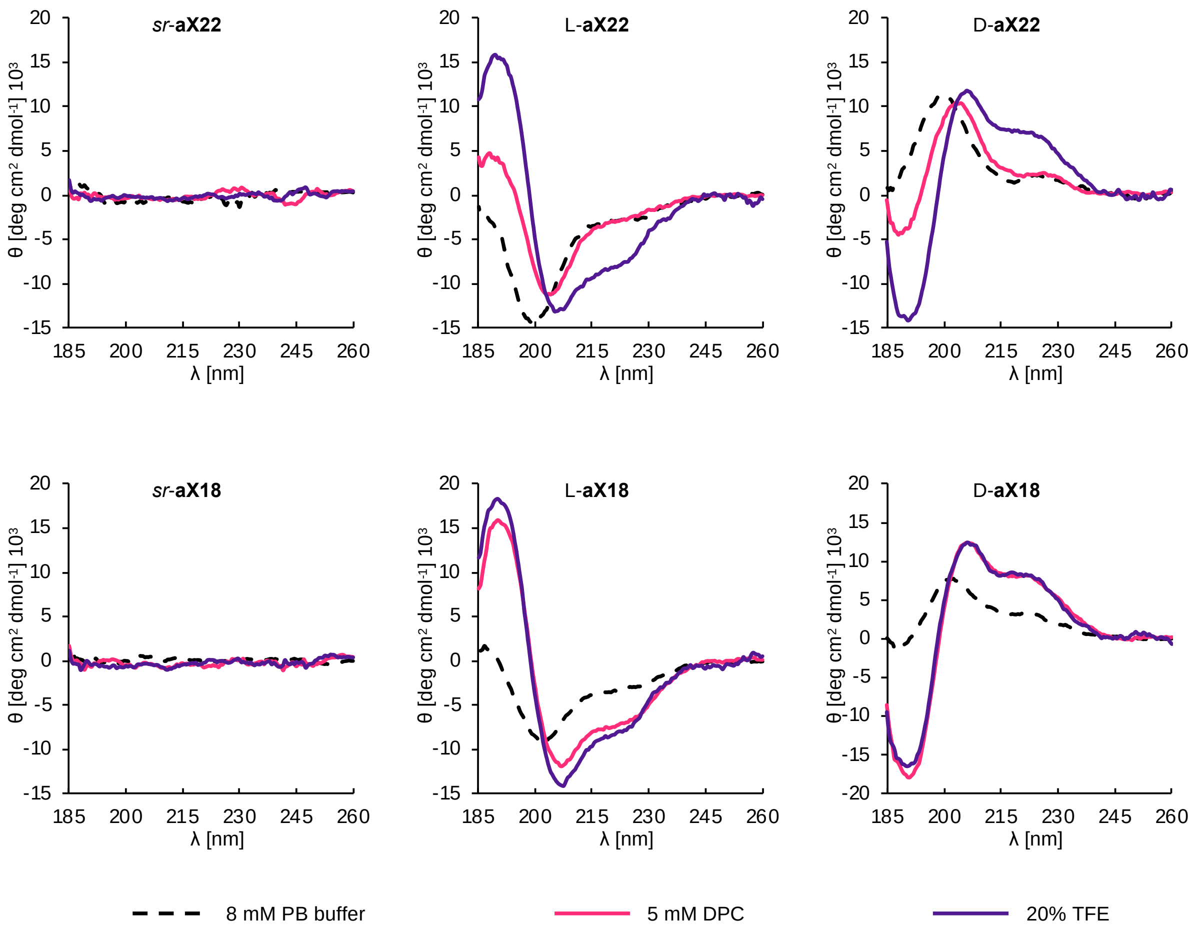

Figure S32. CD spectrum of dendrimers (0.100 mg/mL) in aq. phosphate buffer (8 mM) at different pH upon addition of DPC or TFE.

14. Molecular Dynamics (MD)

MD simulations were performed for dendrimers L-**X18** and L-**X22** using GROMACS software version 2022 and the gromos53a6 force field. The dendrimer topologies were built by combining topologies of two linear peptides with the same sequence, one with alpha and one with epsilon connectivity at the branching lysines, using in house scripts. The starting confirmation was built by hand in PyMol software by setting all the dihedral angles to α-helix conformation. A dodecahedral box was created around the peptide 1.0 nm from the edge of the system and filled with extended simple point charge water molecules. Sodium and chloride ions were added to produce an electroneutral solution at a final concentration of 0.15 M NaCl. The energy was minimized using a steepest gradient method to remove any close contacts before the system was subjected to a two-phase position-restrained MD equilibration procedure. The system was first allowed to evolve for 100 ps in a canonical NVT (N is the number of particles, V the system volume, and T the temperature) ensemble at 300 K before pressure coupling was switched on and the system was equilibrated for an additional 100 ps in the NPT (P is the system pressure) ensemble at 1.0 bar and used for production runs.

14.1 MD in the presence of a DPC micelle

MD simulations in the presence of a DPC micelle were performed as follows. Parameters (itp for GROMOS53a6) and references for the DPC molecule are given below. Dendrimers were manually placed at a distance from the pre-equilibrated micelle (of 65 DPC molecules) approximatively equal to the diameter of said peptide. Box, solvation and NVT equilibration procedures were performed as explained above. For each peptide/micelle system, multiple runs of 20 ns were generated to show the possibility for the peptide to either interact or diffuse away from the micelle. Then, runs of interest where the dendrimer was interacting with the micelle, were extended to 200 ns.

```
; Charge from Chiu et al.
; Chiu, S. W.; Clark, M.; Balaji, V.; Subramaniam, S.; Scott, H. L.; Jakobsson, E. Incorporation
of surface tension into molecular dynamics simulation of an interface: a fluid phase lipid
bilayer membrane. Biophys. J. 1995, 69, 1230-1245.
; Atom types from GROMOS53A6
; Oostenbrink, C.; Soares, T. A.; van der Vegt, N. F. A.; van Gunsteren, W. F. Validation of
the 53A6 GROMOS force field. Eur. Biophys. J. 2005, 34, 273-284.

[ moleculetype ]
; Name   nrexcl
DPC      3

[ atoms ]
;   nr    type   resnr  residu    atom    cgnr        charge          mass
     1     CH3       1    DPC      C1       1         0.40  15.035  ; qtot: 0.25
     2     CH3       1    DPC      C2       2         0.40  15.035  ; qtot: 0.50
     3     CH3       1    DPC      C3       3         0.40  15.035 ; qtot: 0.75
     4     NL        1    DPC      N4       4        -0.5  14.0067 ; qtot: 0.75
     5     CH2       1    DPC      C5       5         0.30  14.027 ; qtot:  1.0
     6     CH2       1    DPC      C6       6         0.40  14.027 ; qtot: 1.0
     7      OA       1    DPC      O7       7        -0.80  15.999 ; qtot: 0.64
     8       P       1    DPC      P8       8         1.7  30.973 ; qtot : 1.63
     9      OM       1    DPC      O9       9        -0.8 15.999 ; qtot: 0.995
    10      OM       1    DPC     O10      10        -0.8 15.999 ; qtot: 0.36
    11      OA       1    DPC     O11      11        -0.7  15.999 ; qtot: 0.0
    12     CH2       1    DPC     C12      12         0.0   14.027  ; qtot: 0
    13     CH2       1    DPC     C13      13         0.0   14.027  ; qtot: 0
    14     CH2       1    DPC     C14      14         0.0   14.027  ; qtot: 0
    15     CH2       1    DPC     C15      15         0.0   14.027  ; qtot: 0
    16     CH2       1    DPC     C16      16         0.0   14.027  ; qtot: 0
    17     CH2       1    DPC     C17      17         0.0   14.027  ; qtot: 0
    18     CH2       1    DPC     C18      18         0.0   14.027  ; qtot: 0
    19     CH2       1    DPC     C19      19         0.0   14.027  ; qtot: 0
    20     CH2       1    DPC     C20      20         0.0   14.027  ; qtot: 0
    21     CH2       1    DPC     C21      21         0.0   14.027  ; qtot: 0
    22     CH2       1    DPC     C22      22         0.0   14.027  ; qtot: 0
    23     CH3       1    DPC     C23      23         0.0   15.035  ; qtot: 0


[ bonds ]
;  ai    aj funct            c0            c1            c2            c3

    1     4     2    gb_21
    2     4     2    gb_21
    3     4     2    gb_21
    4     5     2    gb_21
    5     6     2    gb_27
```

```
    6     7     2    gb_18
    7     8     2    gb_28
    8     9     2    gb_24
    8    10     2    gb_24
    8    11     2    gb_28
   11    12     2    gb_18
   12    13     2    gb_27
   13    14     2    gb_27
   14    15     2    gb_27
   15    16     2    gb_27
   16    17     2    gb_27
   17    18     2    gb_27
   18    19     2    gb_27
   19    20     2    gb_27
   20    21     2    gb_27
   21    22     2    gb_27
   22    23     2    gb_27

[ pairs ]
;  ai    aj funct
    1     6     1
    2     6     1
    3     6     1
    4     7     1
    5     8     1
    6     9     1
    6    10     1
    6    11     1
    7    12     1
    8    13     1
    9    12     1
   10    12     1
   11    14     1
;   12    15     1
;   13    16     1
;   14    17     1
;   15    18     1
;   16    19     1
;   17    20     1
;   18    21     1
;   19    22     1
;   20    23     1

[ angles ]
;  ai    aj    ak funct
    1     4     2     2    ga_13
    1     4     3     2    ga_13
    1     4     5     2    ga_13
    2     4     3     2    ga_13
    2     4     5     2    ga_13
    3     4     5     2    ga_13
    4     5     6     2    ga_15
    5     6     7     2    ga_15
    6     7     8     2    ga_26
    7     8     9     2    ga_14
    7     8    10     2    ga_14
    7     8    11     2    ga_5
    9     8    10     2    ga_29
   10     8    11     1    ga_14
    8    11    12     1    ga_26
   11    12    13     1    ga_15
   12    13    14     1    ga_15
   13    14    15     1    ga_15
   14    15    16     1    ga_15
   15    16    17     1    ga_15
   16    17    18     1    ga_15
   17    18    19     1    ga_15
   18    19    20     1    ga_15
   19    20    21     1    ga_15
   20    21    22     1    ga_15
   21    22    23     1    ga_15

[ dihedrals ]
;  ai    aj    ak    al funct
    1     4     5     6     1 gd_29
    4     5     6     7     1 gd_4
```

```
     4      5      6      7      1 gd_36
     5      6      7      8      1 gd_29
;
; define gd_20     0.000       5.09          2
; O-P-O- (dna, lipids)  1.2
     6      7      8      9      1 gd_20
     7      8     11     12      1 gd_27
     8     11     12     13      1 gd_29
    11     12     13     14      1 gd_1
    12     13     14     15      1 gd_34
    13     14     15     16      1 gd_34
    14     15     16     17      1 gd_34
    15     16     17     18      1 gd_34
    16     17     18     19      1 gd_34
    17     18     19     20      1 gd_34
    18     19     20     21      1 gd_34
    19     20     21     22      1 gd_34
    20     21     22     23      1 gd_34
```

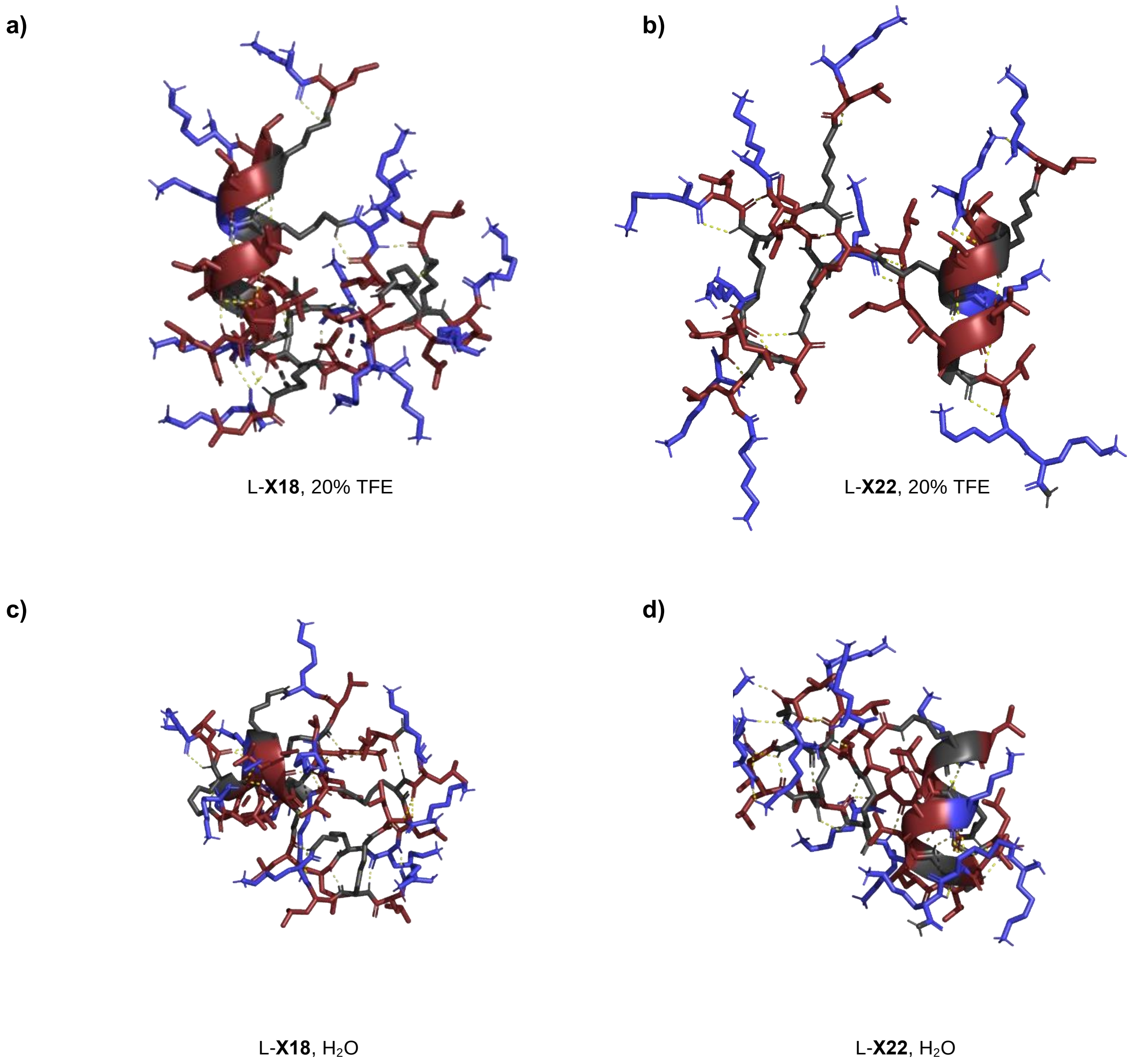

Figure S33. MD simulation of L-**X18** and L-**X22** in 20% TFE and water environment.

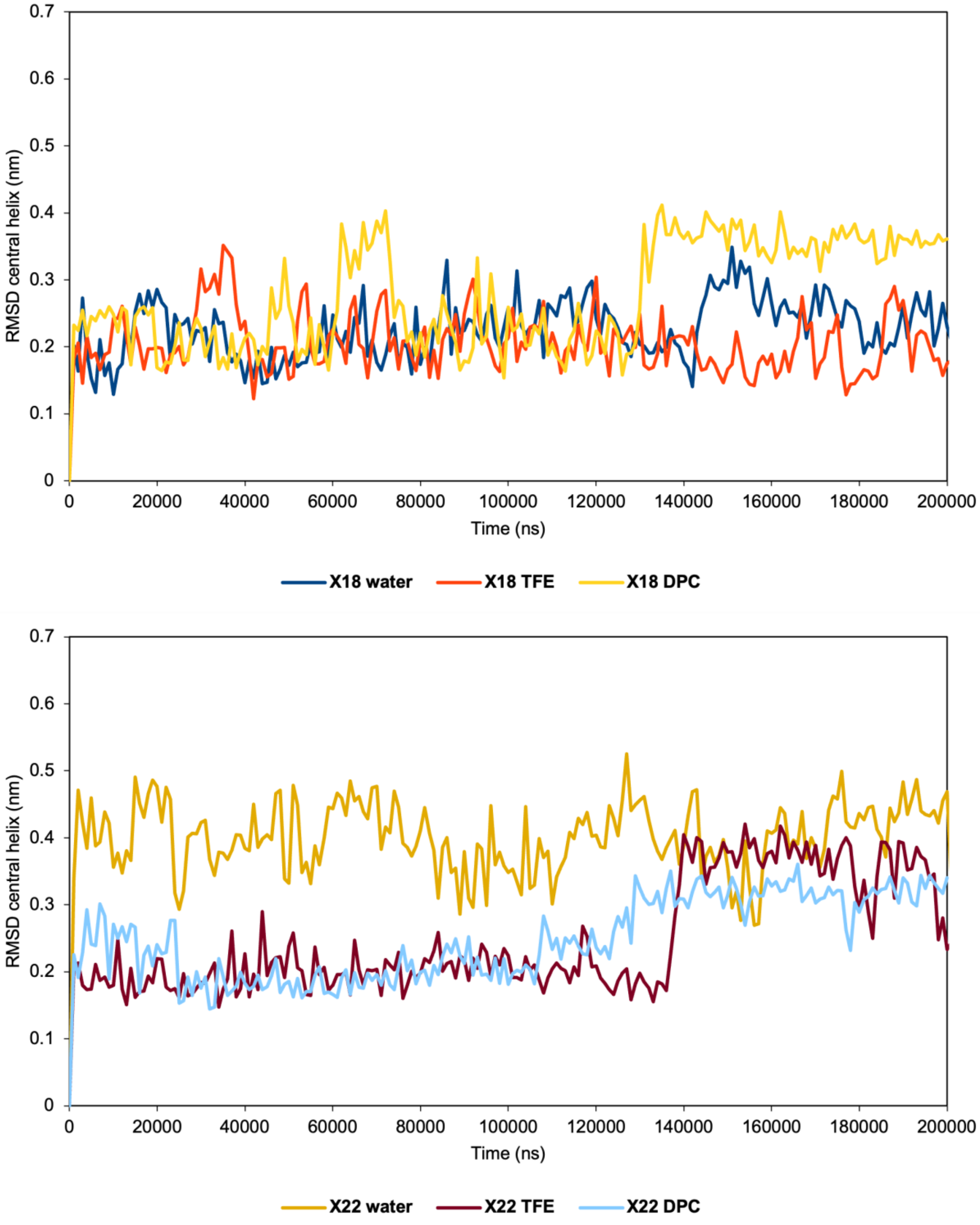

Figure S34. RMSD of the central a-helix over the course of the MD simulations in water, 20% TFE and DPC.

15. Critical Micellar Concentration (CMC)

Nile red (Sigma Aldrich, Buchs, CH) was desolved in methanol at a concentration of 2 µM and 5 µL were added to each well of a TPP 96-well plate (Faust Laborbedarf AG, Schaffhausen) and dry under the fume hood air flow at room temperature for 1 h. ½ serial dilution of the peptide dendrimers, were performed in 10 mM PBS (pH 7.4) starting from 2 mg/mL in an additional plate. 50 µL of diluted peptide solution was added to the plate containing the dried Nile red fluorophore (final concentration 0.2 µM). The plates were incubated for 2 h before measurement of fluorescence at λex= 540 nm and λem= 615 nm on a Tecan Infinite M1000 Pro plate reader.[3]

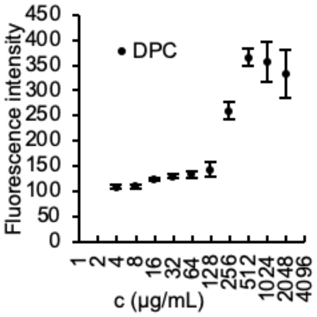

Figure S35. DPC in PBS (pH 7.4) at varying concentration in presence of 0.2 µM Nile red.

16. Aggregation of peptide dendrimers

For imaging of negatively stained samples, 5ul of each peptide dendrimer solution (10 mg/mL) in PBS pH 7.4 were adsorbed on glow discharged and carbon coated 400 mesh copper grids (Plano, Wetzlar, Germany) for 2 minutes. After washing them 3 times by dipping in pure water, grids were stained with 2% uranyl acetate solution (Electron Microscopy Science, Hatfield, USA) for 30 seconds. The excess fluid was removed by gently pushing them sideways to filter paper.

Samples were then examined with a transmission electron microscope (Tecnai Spirit, FEI, Hillsboro, USA) at 80kV and equipped with a digital camera (Veleta, Olympus, Münster, Germany).

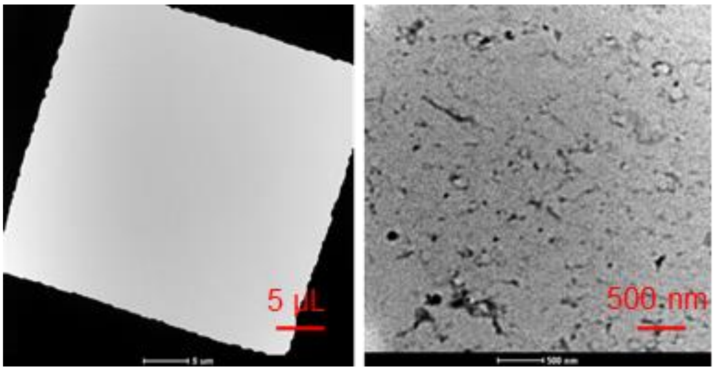

Figure S36. Example of TEM images of non-aggregating peptide dendrimers (10 mg/mL) in PBS deposited on glow discharged 400 mesh copper grids, dried and stained by uranyl acetate.

[1] D. Erzina, A. Capecchi, S. Javor, J. Reymond, *Angew. Chem. Int. Ed.* **2021**, *60*, 26403–26408.
[2] X. Cai, S. Javor, B. H. Gan, T. Köhler, J.-L. Reymond, *Chem. Commun.* **2021**, *57*, 5654–5657.
[3] M. Heitz, S. Javor, T. Darbre, J.-L. Reymond, *Bioconjug. Chem.* **2019**, *30*, 2165–2182.
[4] T. N. Siriwardena, A. Capecchi, B.-H. Gan, X. Jin, R. He, D. Wei, L. Ma, T. Köhler, C. van Delden, S. Javor, J.-L. Reymond, *Angew. Chem. Int. Ed.* **2018**, *57*, 8483–8487.
[5] A. Capecchi, X. Cai, H. Personne, T. Köhler, C. van Delden, J.-L. Reymond, *Chem. Sci.* **2021**, *12*, 9221–9232.
[6] B.-H. Gan, T. N. Siriwardena, S. Javor, T. Darbre, J.-L. Reymond, *ACS Infect. Dis.* **2019**, *5*, 2164–2173.

sr-**X1** ((LL)$_8$(*K*LL)$_4$(*K*KL)$_2$*K*LKL) was manually synthesized using TentaGel S RAM resin (393.4 mg, 0.09 mmol, 0.22 mmol·g^{-1}), the dendrimer was obtained as a white foamy solid after preparative RP-HPLC purification (153.1 mg, 33.5%). Analytical RP-HPLC: t_R = 1.82 min (100% A to 100% B in 3.5 min, λ= 214 nm). MS (ESI+): $C_{228}H_{431}N_{49}O_{38}$ calc./obs. 4464.33/4464.33 $[M]^+$.

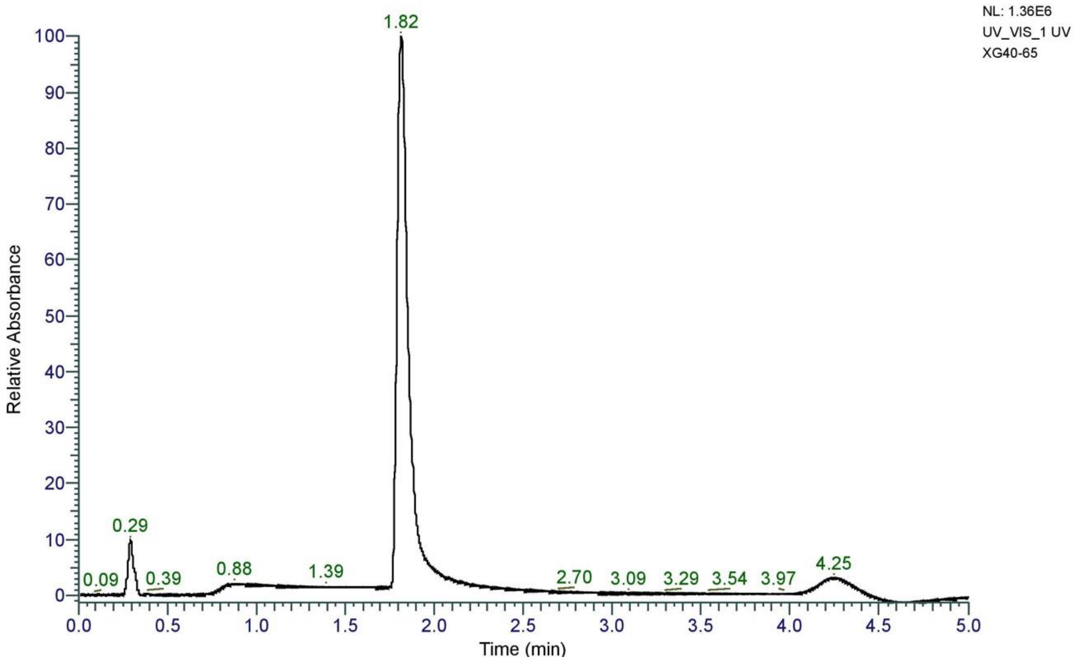

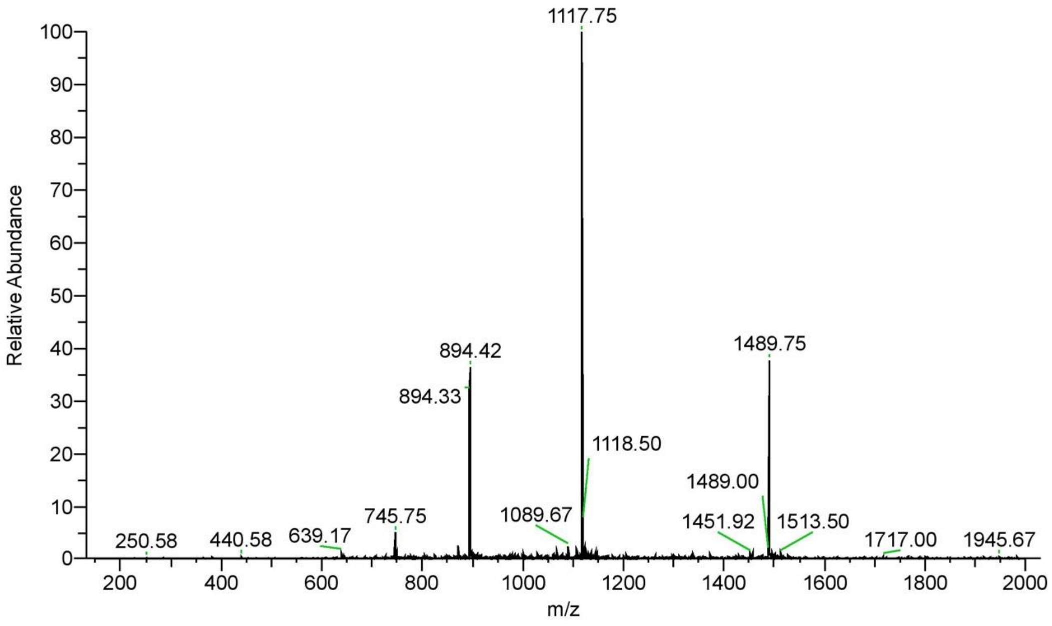

Figure S37. LCMS spectrum.

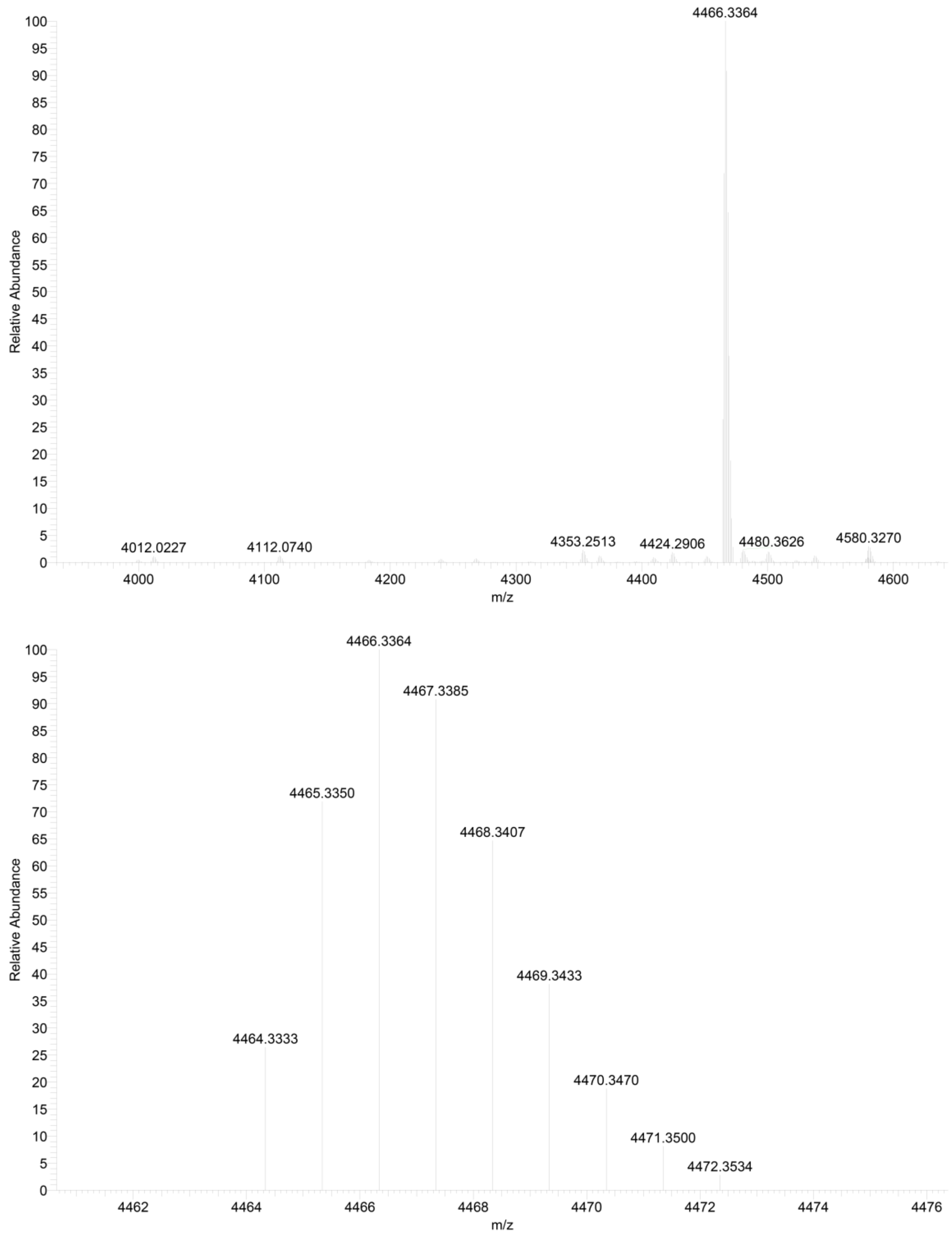

Figure S38. HRMS spectrum.

sr-**X2** ((LL)$_8$(*K*KL)$_4$(*K*LL)$_2$*K*LLK) was manually synthesized using TentaGel S RAM resin (393.4 mg, 0.09 mmol, 0.22 mmol·g^{-1}), the dendrimer was obtained as a white foamy solid after preparative RP-HPLC purification (160.3 mg, 33.5%). Analytical RP-HPLC: t_R = 1.70 min (100% A to 100% B in 3.5 min, λ= 214 nm). MS (ESI+): $C_{228}H_{433}N_{51}O_{38}$ calc./obs. 4494.35/4494.39 $[M]^+$.

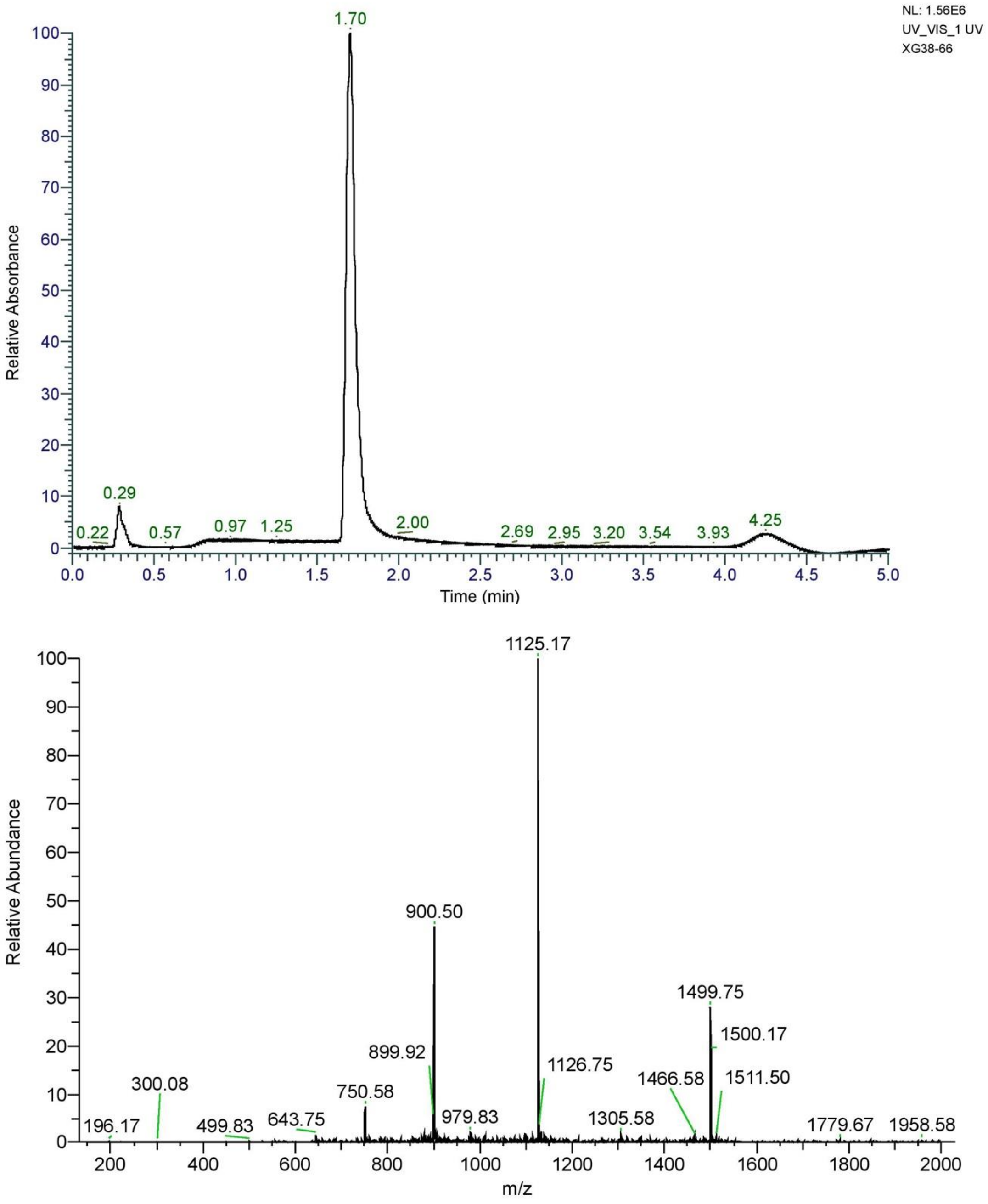

Figure S39. LCMS spectrum.

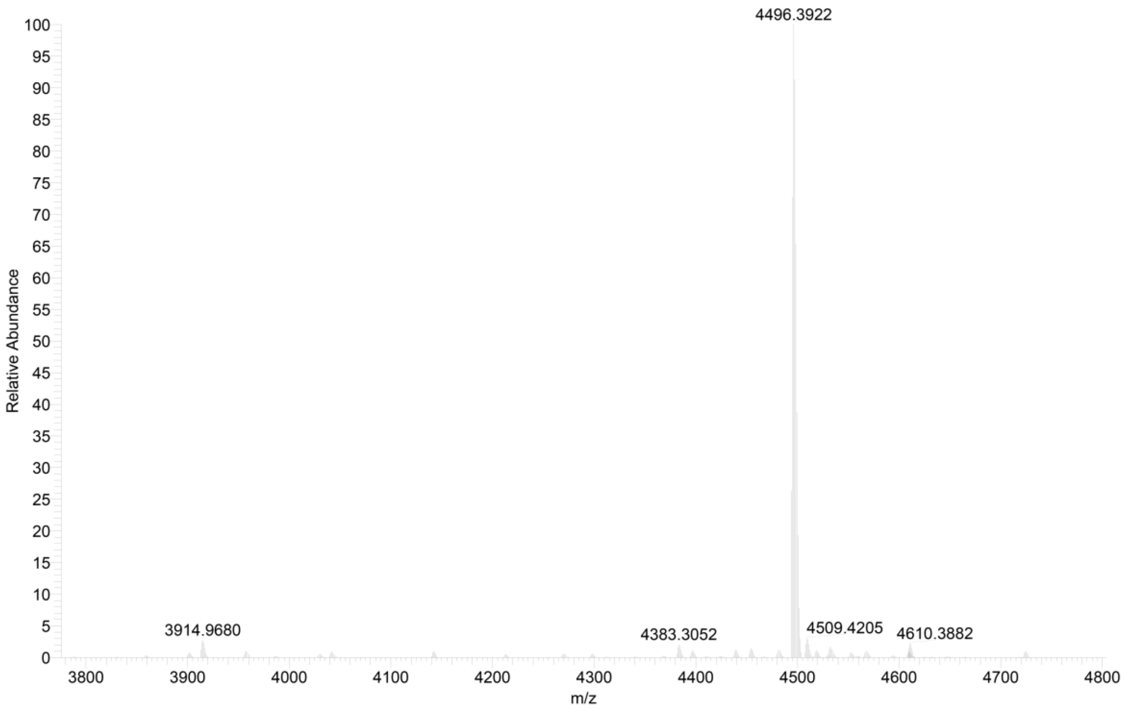

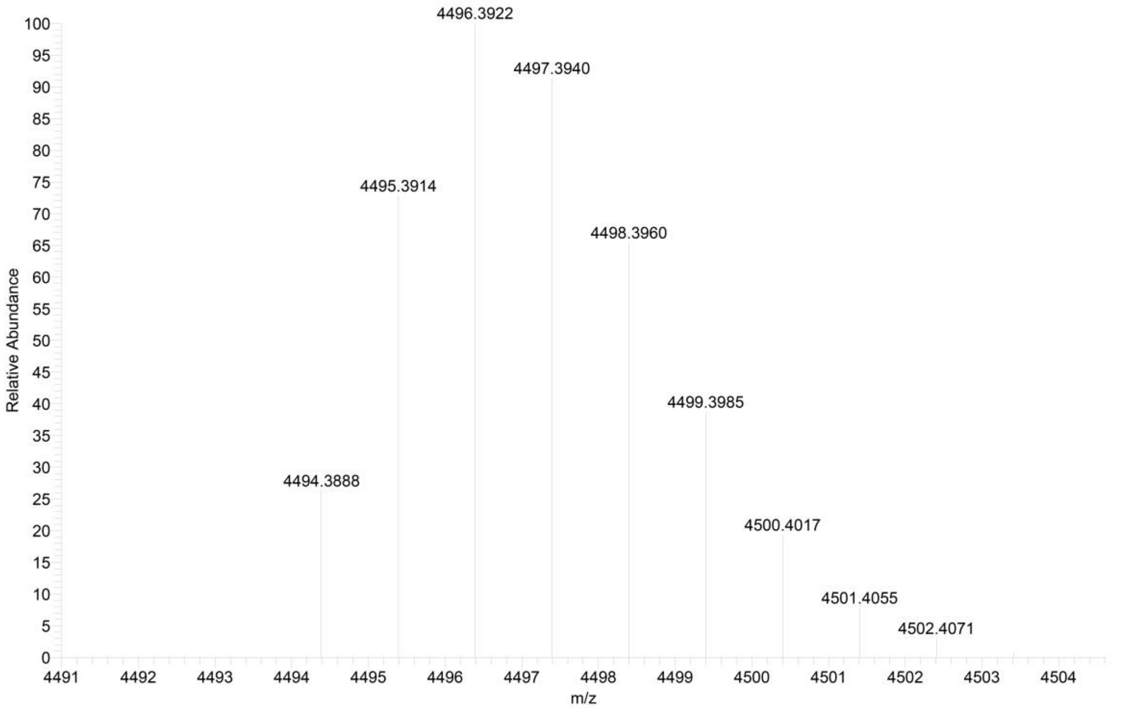

Figure S40. HRMS spectrum.

sr-**X3** ((*K*L)$_8$(*K*LL)$_4$(*K*LL)$_2$*K*LLL) was synthesized by CEM Liberty Blue synthesizer using Rink Amide MBHA resin (320.0 mg, 0.08 mmol, 0.25 mmol·g^{-1}), the dendrimer was obtained as a white foamy solid after preparative RP-HPLC purification (52.0 mg, 10.2%). Analytical RP-HPLC: t_R = 1.62 min (100% A to 100% B in 3.5 min, λ= 214 nm). MS (ESI+): $C_{228}H_{436}N_{54}O_{38}$ calc./obs. 4539.38/4539.37 [M]$^+$.

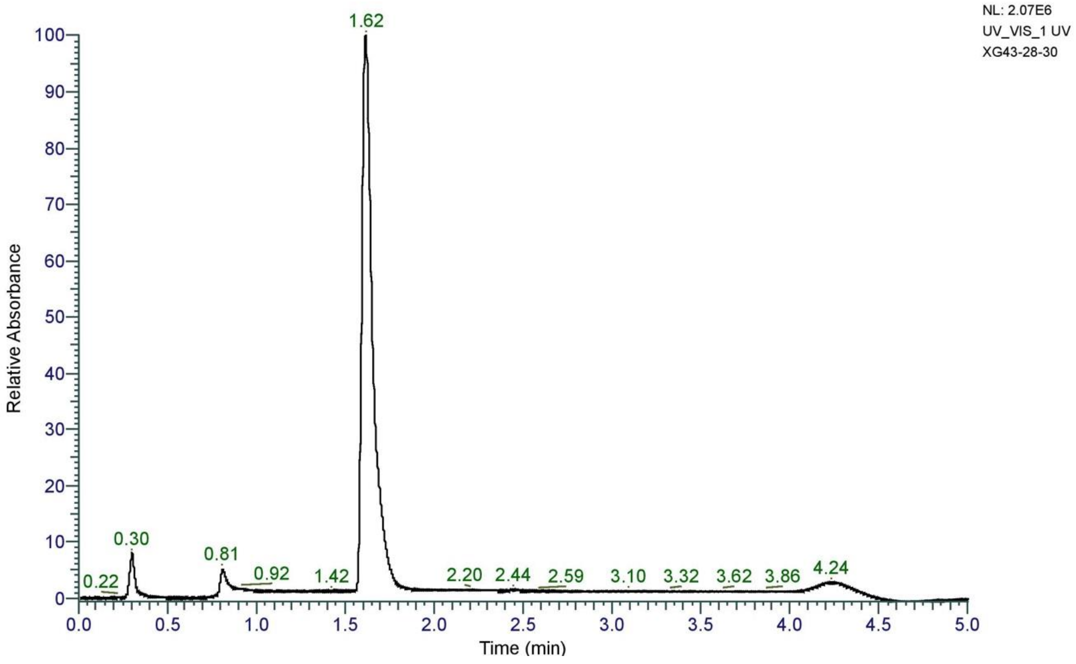

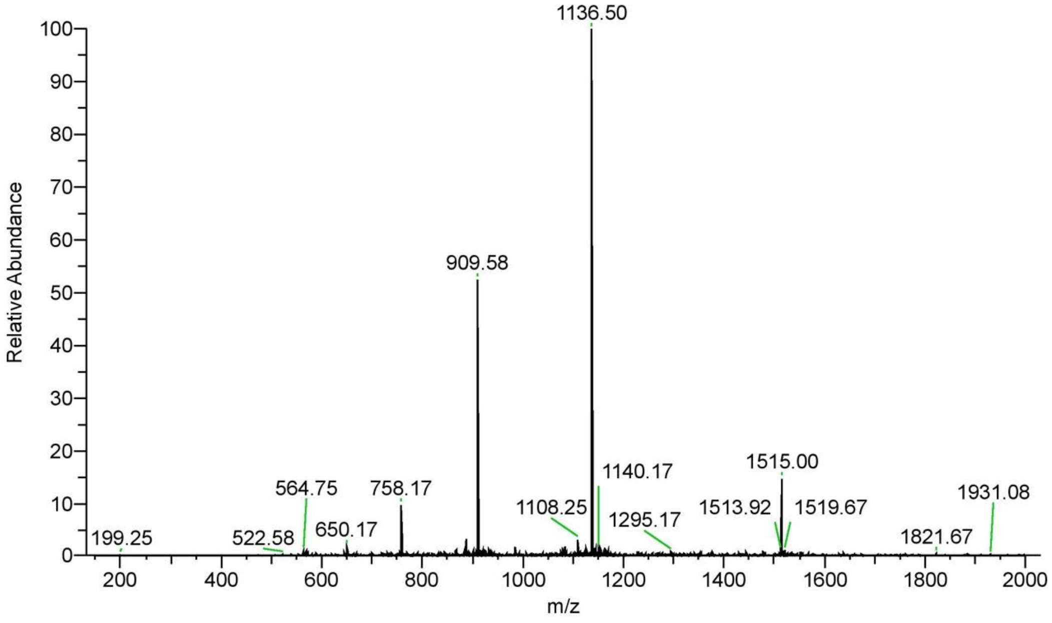

Figure S41. LCMS spectrum.

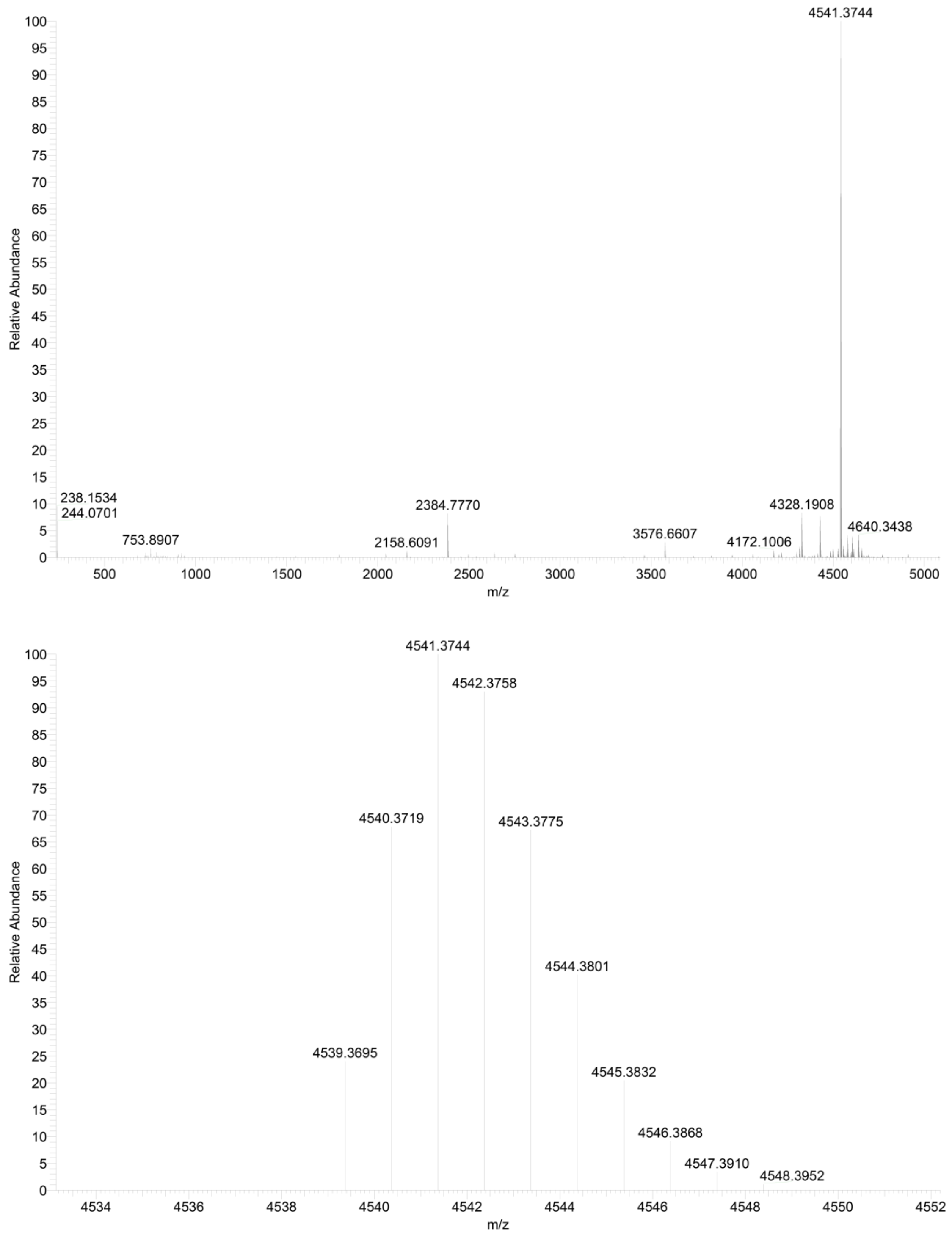

Figure S42. HRMS spectrum.

sr-**X4** ((LL)$_8$(*K*KK)$_4$(*K*LL)$_2$*K*LLK) was manually synthesized using TentaGel S RAM resin (393.4 mg, 0.09 mmol, 0.22 mmol·g^{-1}), the dendrimer was obtained as a white foamy solid after preparative RP-HPLC purification (141.9 mg, 27.3%). Analytical RP-HPLC: t_R = 1.59 min (100% A to 100% B in 3.5 min, λ= 214 nm). MS (ESI+): $C_{228}H_{437}N_{55}O_{38}$ calc./obs. 4554.40/4554.41 $[M]^+$.

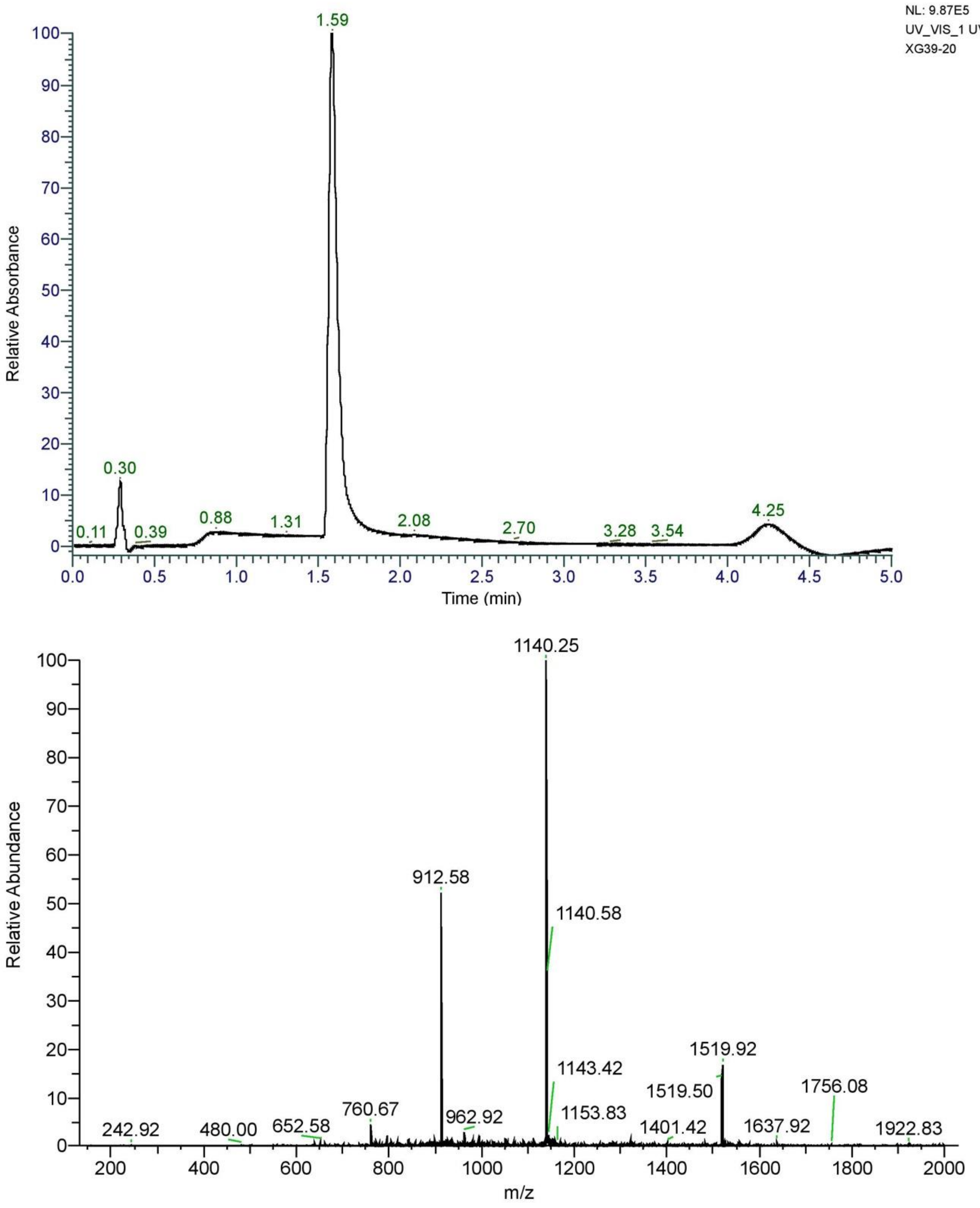

Figure S43. LCMS spectrum.

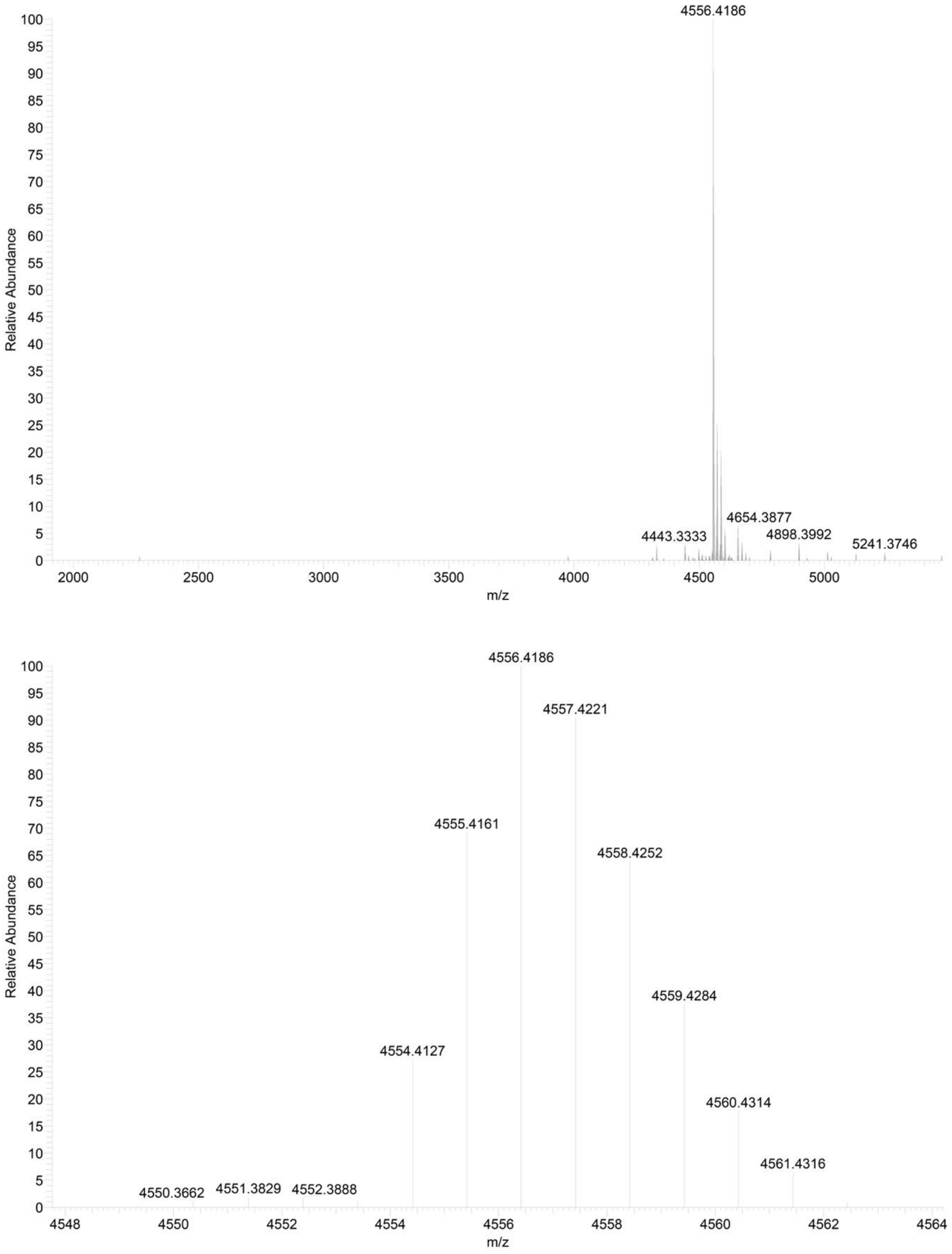

Figure S44. HRMS spectrum.

sr-**X5** ((*K*L)$_8$(*K*LL)$_4$(*K*KL)$_2$*K*LLL) was manually synthesized using TentaGel S RAM resin (393.4 mg, 0.09 mmol, 0.22 mmol·g^{-1}), the dendrimer was obtained as a white foamy solid after preparative RP-HPLC purification (152.2 mg, 28.7%). Analytical RP-HPLC: t_R = 1.52 min (100% A to 100% B in 3.5 min, λ= 214 nm). MS (ESI+): $C_{228}H_{438}N_{56}O_{38}$ calc./obs. 4569.41/4569.40 [M]$^+$.

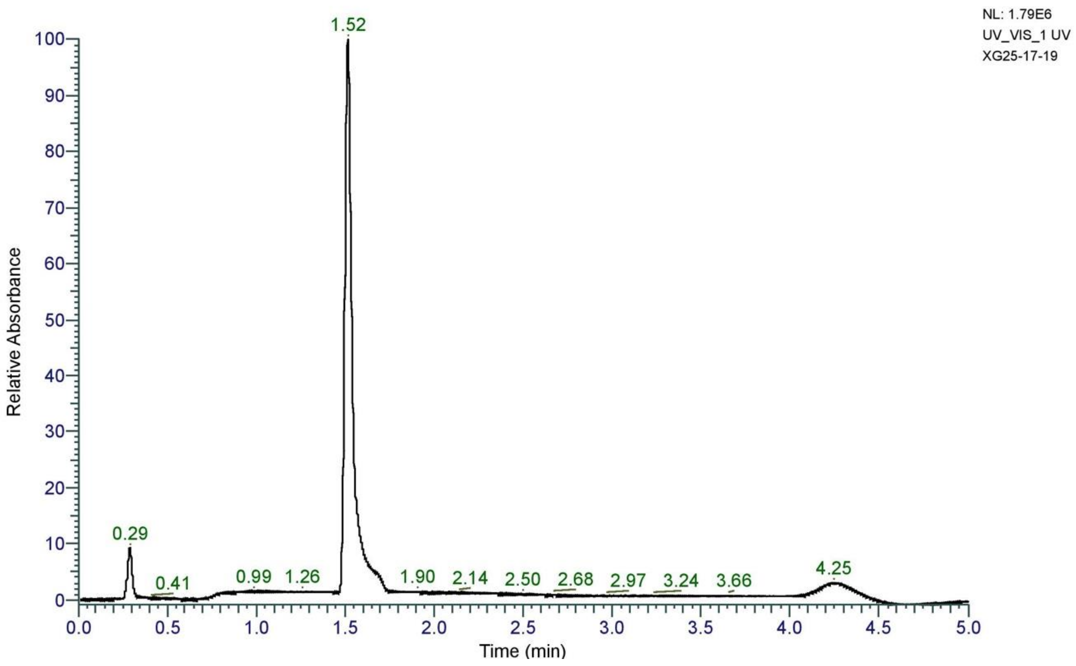

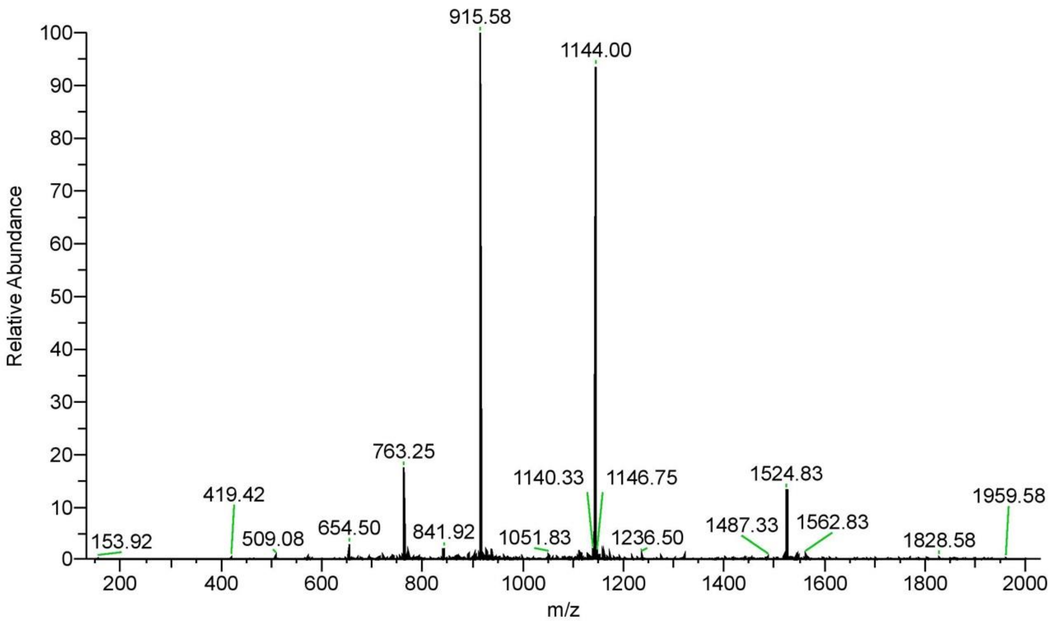

Figure S45. LCMS spectrum.

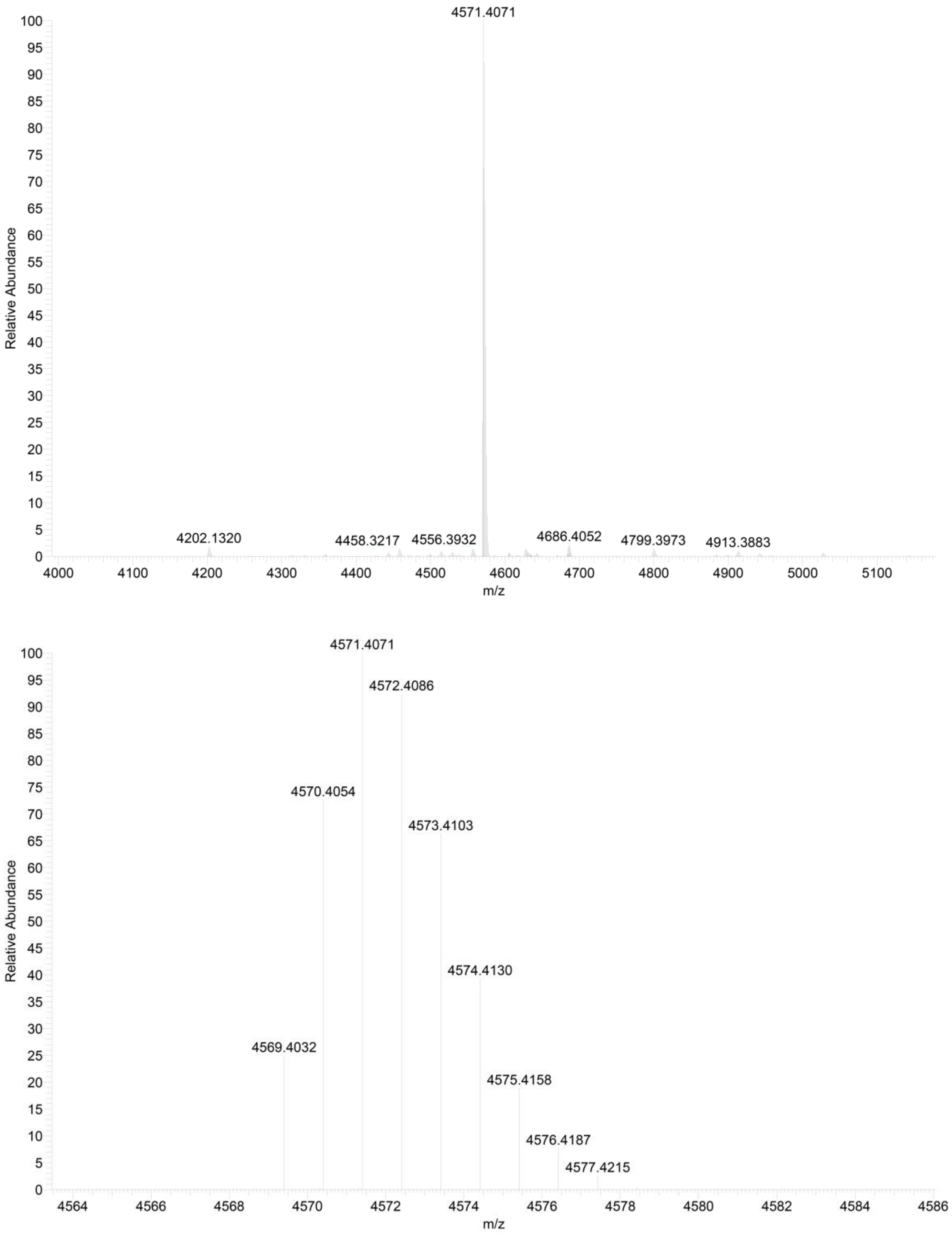

Figure S46. HRMS spectrum.

sr-**X6** ((*K*L)$_8$(*K*LLL)$_4$(*K*K)$_2$*K*LL) was manually synthesized using TentaGel S RAM resin (393.4 mg, 0.09 mmol, 0.22 mmol·g^{-1}), the dendrimer was obtained as a white foamy solid after preparative RP-HPLC purification (69.6 mg, 12.9%). Analytical RP-HPLC: t_R = 1.55 min (100% A to 100% B in 3.5 min, λ= 214 nm). MS (ESI+): $C_{234}H_{449}N_{57}O_{39}$ calc./obs. 4682.49/4682.48 [M]$^+$.

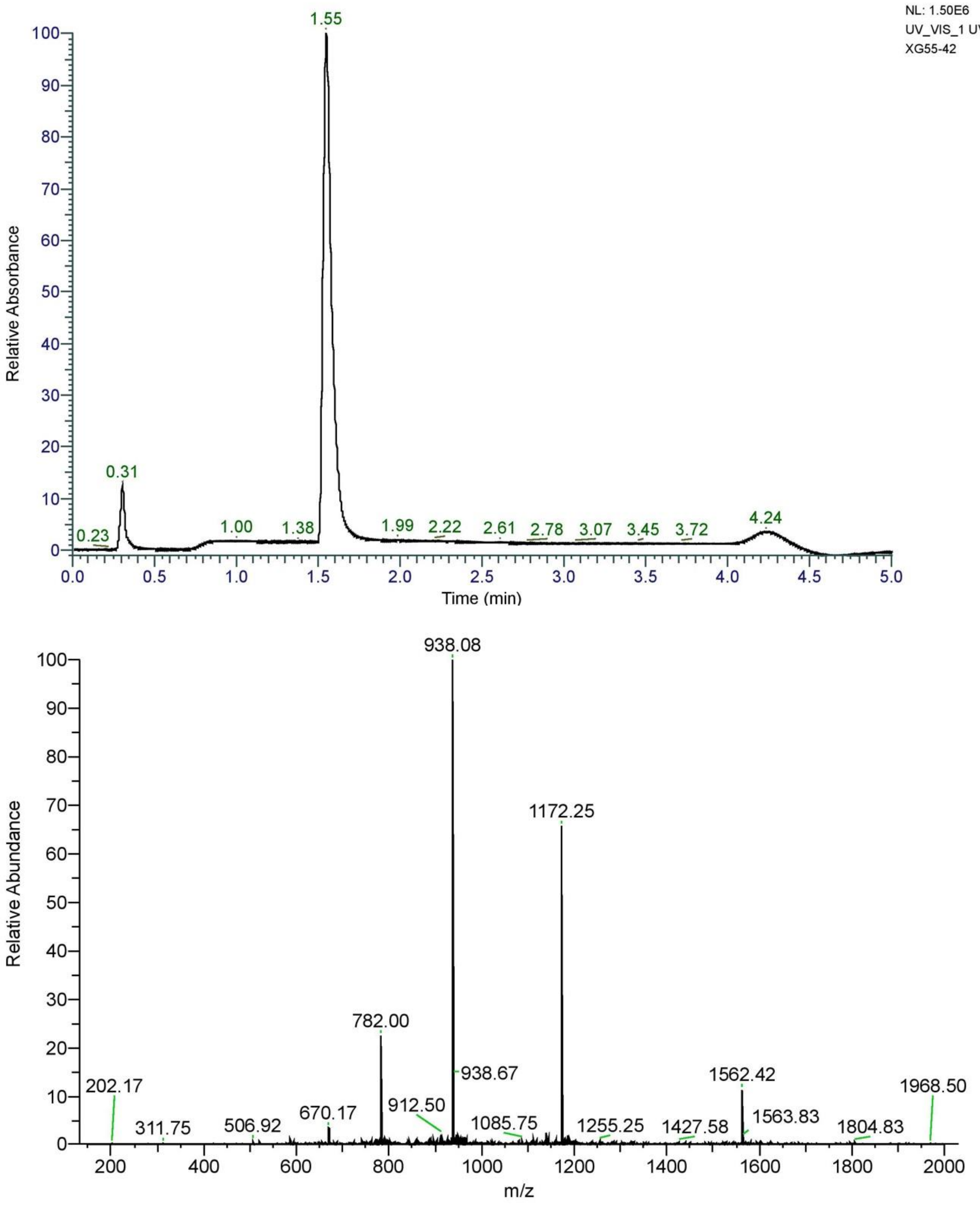

Figure S47. LCMS spectrum.

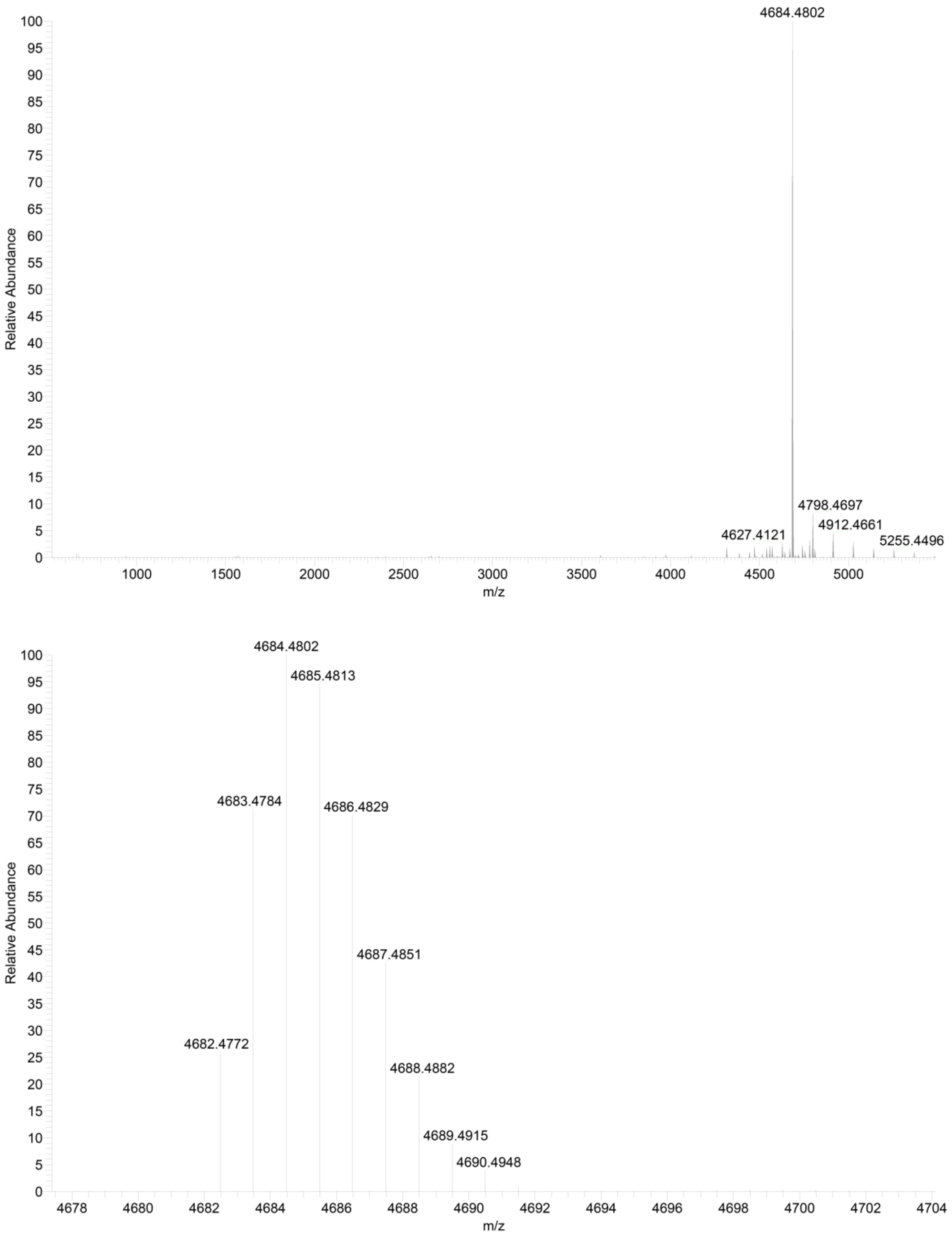

Figure S48. HRMS spectrum.

sr-**X7** ((LK)$_8$(*K*LLL)$_4$(*K*K)$_2$*K*LL) was manually synthesized using TentaGel S RAM resin (393.4 mg, 0.09 mmol, 0.22 mmol·g^{-1}), the dendrimer was obtained as a white foamy solid after preparative RP-HPLC purification (136.6 mg, 25.4%). Analytical RP-HPLC: t_R = 1.49 min (100% A to 100% B in 3.5 min, λ= 214 nm). MS (ESI+): $C_{234}H_{449}N_{57}O_{39}$ calc./obs. 4682.49/4682.48 [M]$^+$.

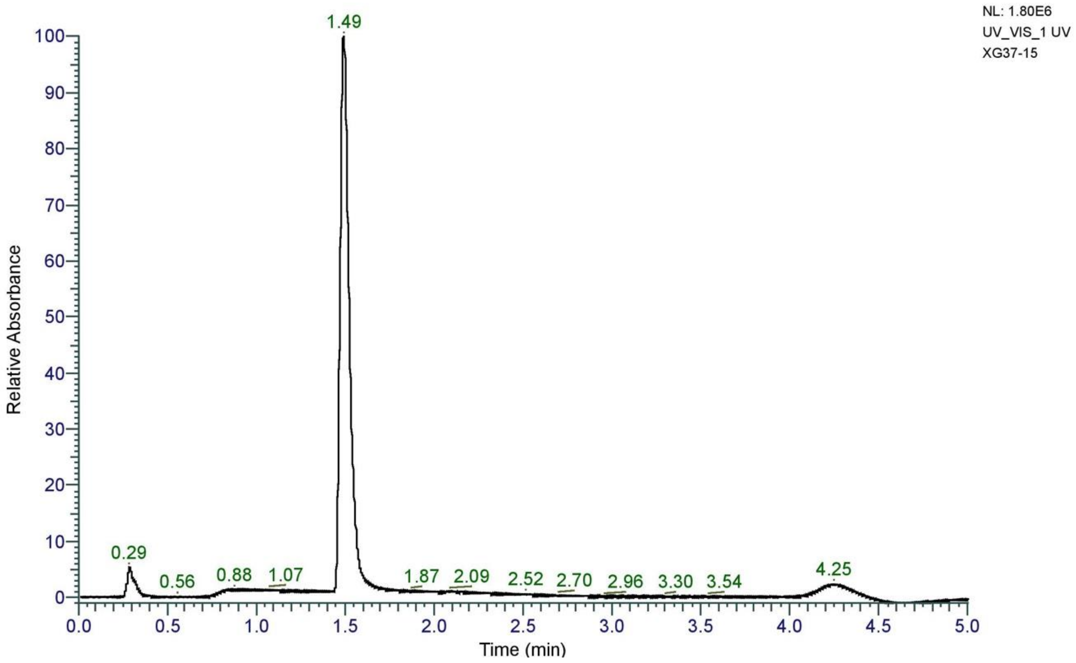

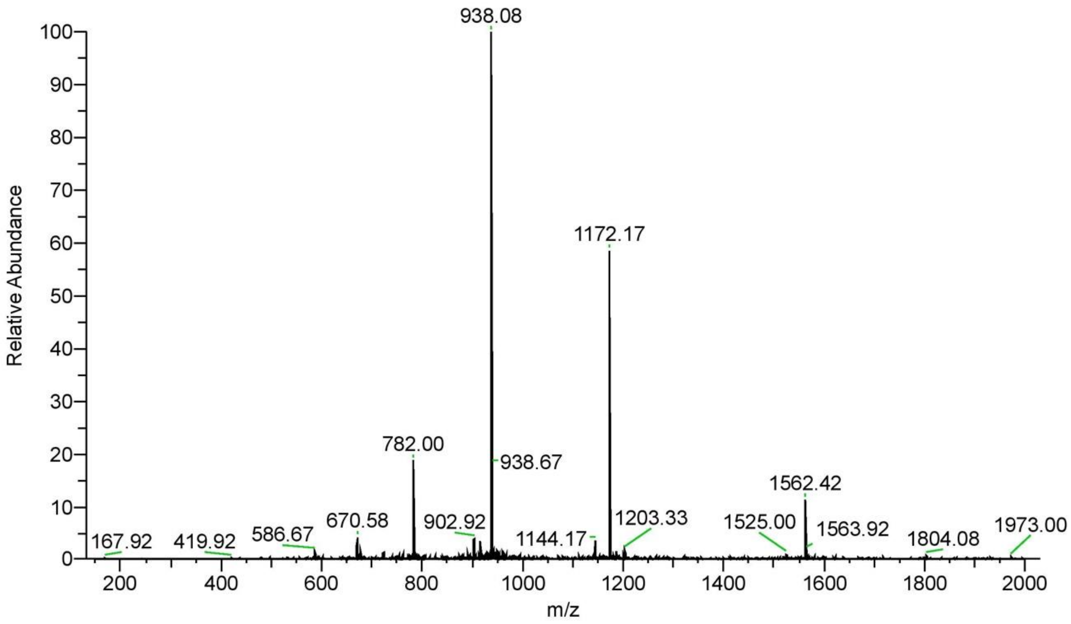

Figure S49. LCMS spectrum.

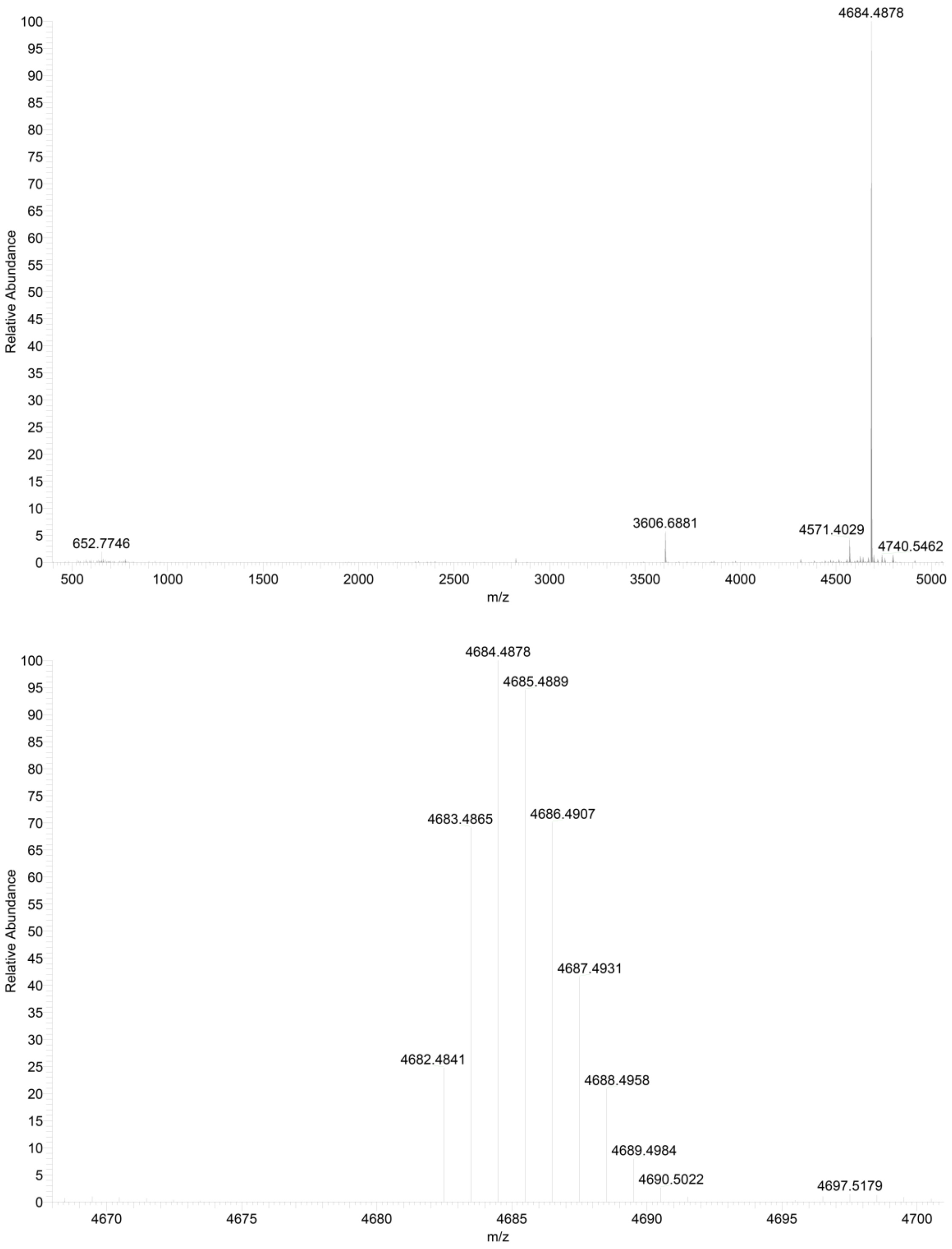

Figure S50. HRMS spectrum.

sr-**X8** ((LK)$_8$(*K*LL)$_4$(*K*KL)$_2$*K*LLL) was manually synthesized using TentaGel S RAM resin (393.4 mg, 0.09 mmol, 0.22 mmol·g^{-1}), the dendrimer was obtained as a white foamy solid after preparative RP-HPLC purification (74.6 mg, 14.1%). Analytical RP-HPLC: t_R = 1.47 min (100% A to 100% B in 3.5 min, λ= 214 nm). MS (ESI+): $C_{228}H_{438}N_{56}O_{38}$ calc./obs. 4569.41/4569.41 [M]$^+$.

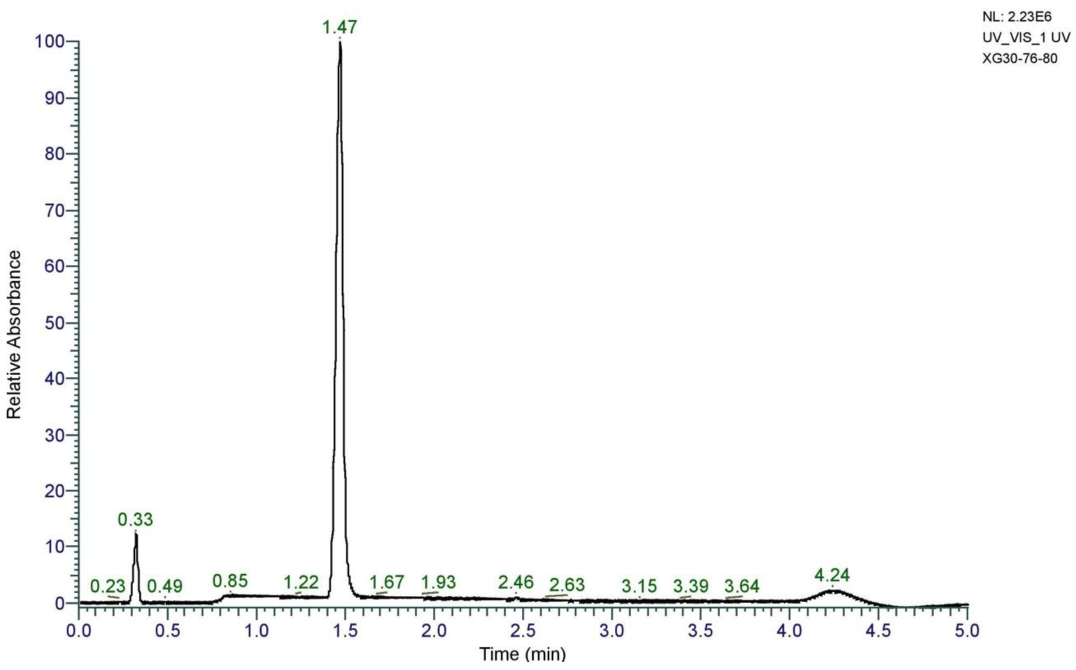

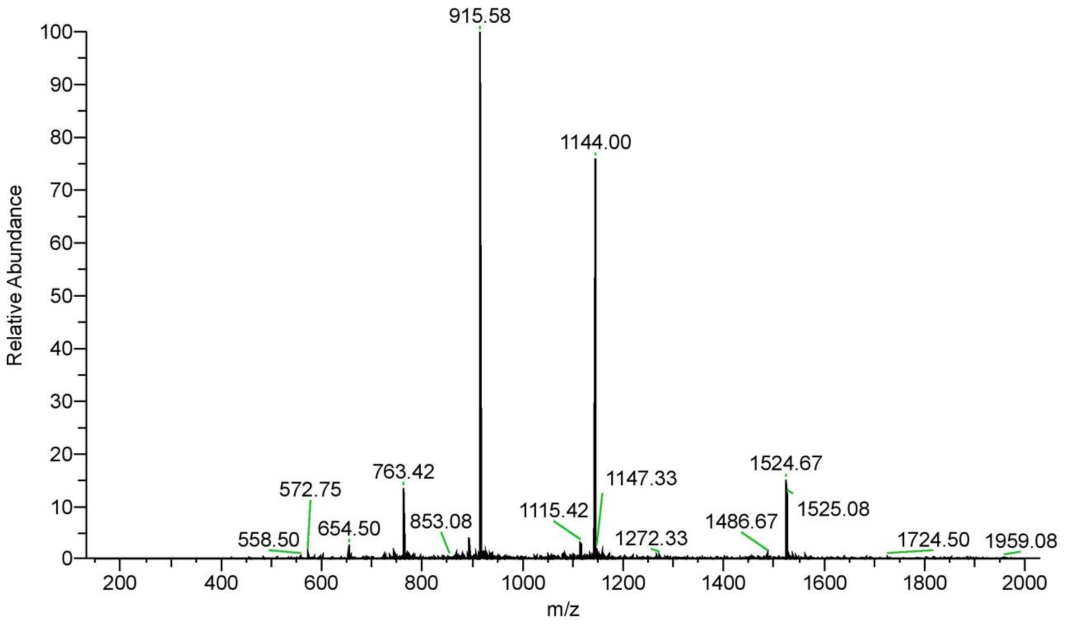

Figure S51. LCMS spectrum.

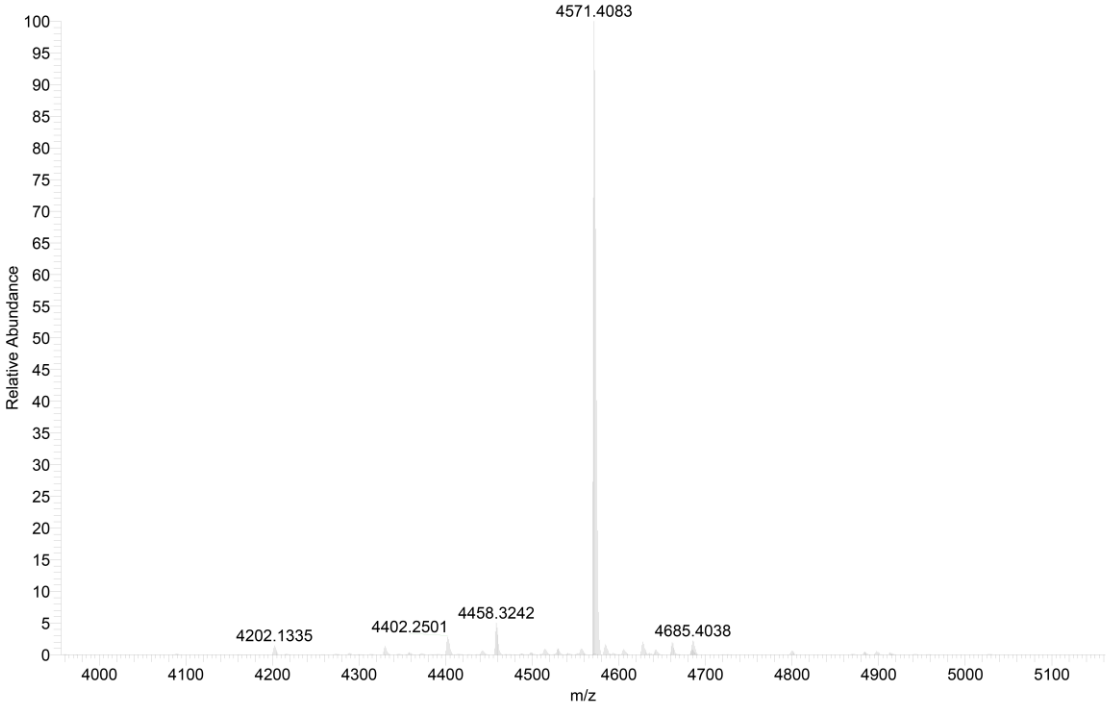

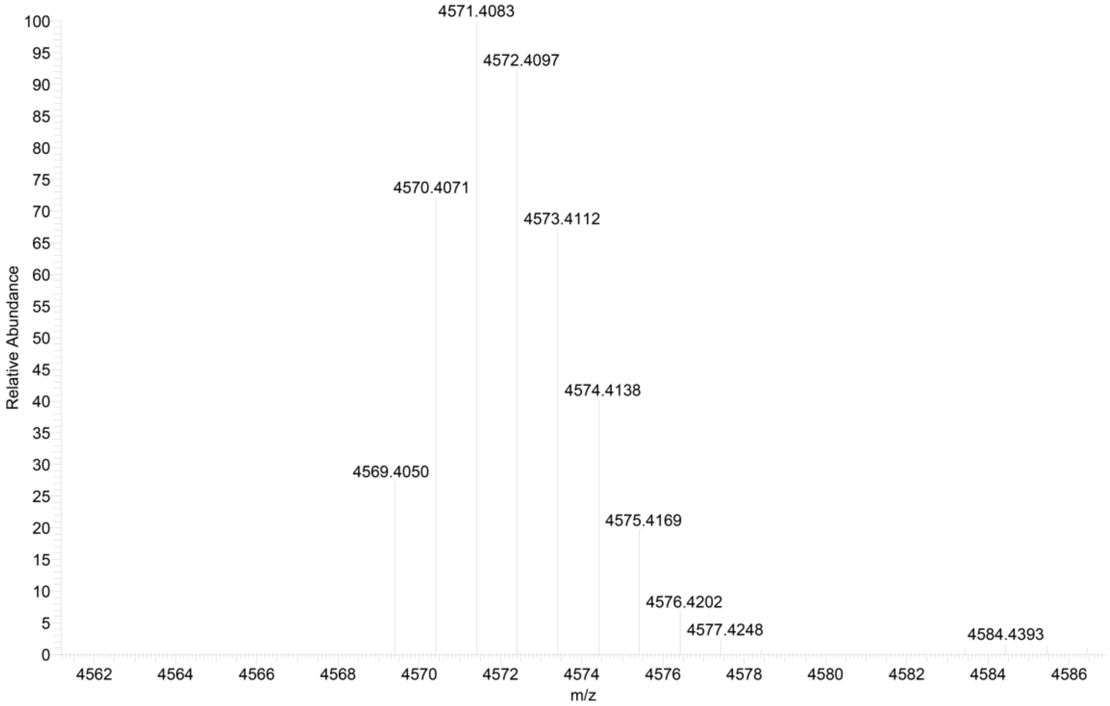

Figure S52. HRMS spectrum.

sr-**X9** ((*K*L)$_8$(*K*LLL)$_4$(*K*K)$_2$*K*KL) was manually synthesized using TentaGel S RAM resin (393.4 mg, 0.09 mmol, 0.22 mmol·g^{-1}), the dendrimer was obtained as a white foamy solid after preparative RP-HPLC purification (89.6 mg, 16.3%). Analytical RP-HPLC: t_R = 1.50 min (100% A to 100% B in 3.5 min, λ= 214 nm). MS (ESI+): $C_{234}H_{450}N_{58}O_{39}$ calc./obs. 4697.50/4697.49 $[M]^+$.

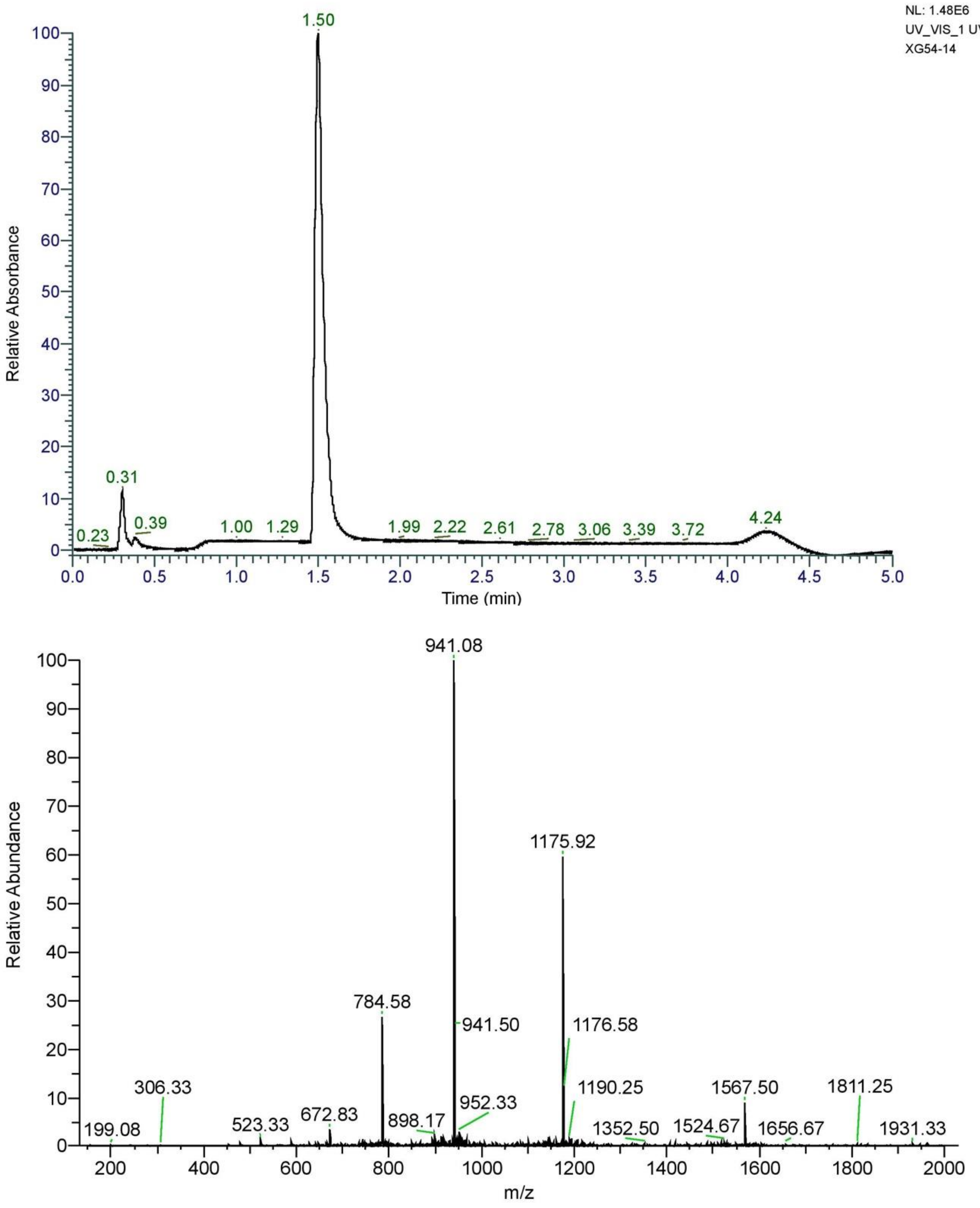

Figure S53. LCMS spectrum.

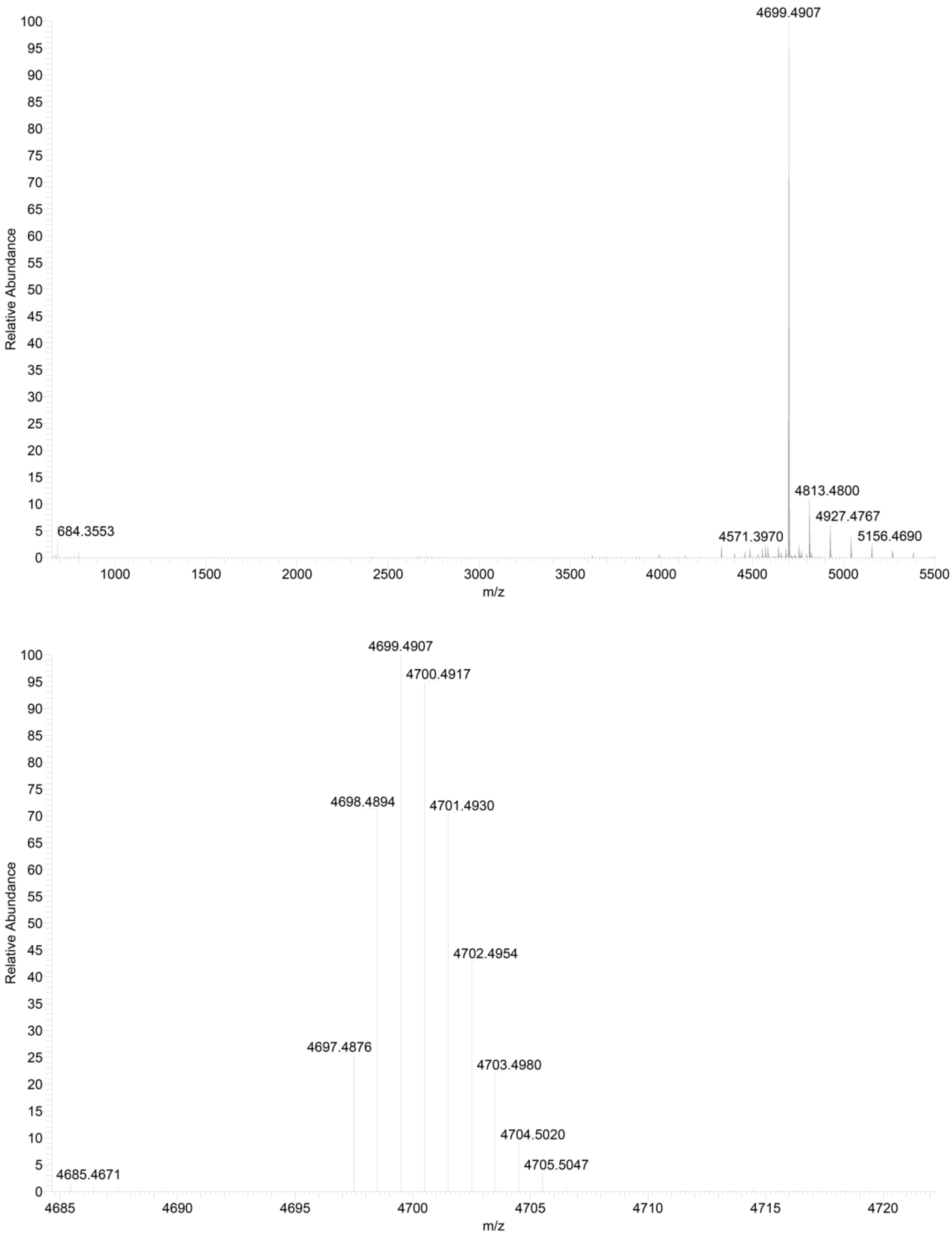

Figure S54. HRMS spectrum.

sr-**X10** ((*K*L)$_8$(*K*LL)$_4$(*K*KL)$_2$*K*LLK) was manually synthesized using TentaGel S RAM resin (393.4 mg, 0.09 mmol, 0.22 mmol·g^{-1}), the dendrimer was obtained as a white foamy solid after preparative RP-HPLC purification (133.4 mg, 24.7%). Analytical RP-HPLC: t_R = 1.44 min (100% A to 100% B in 3.5 min, λ= 214 nm). MS (ESI+): $C_{228}H_{439}N_{57}O_{38}$ calc./obs. 4584.42/4584.42 [M]$^+$.

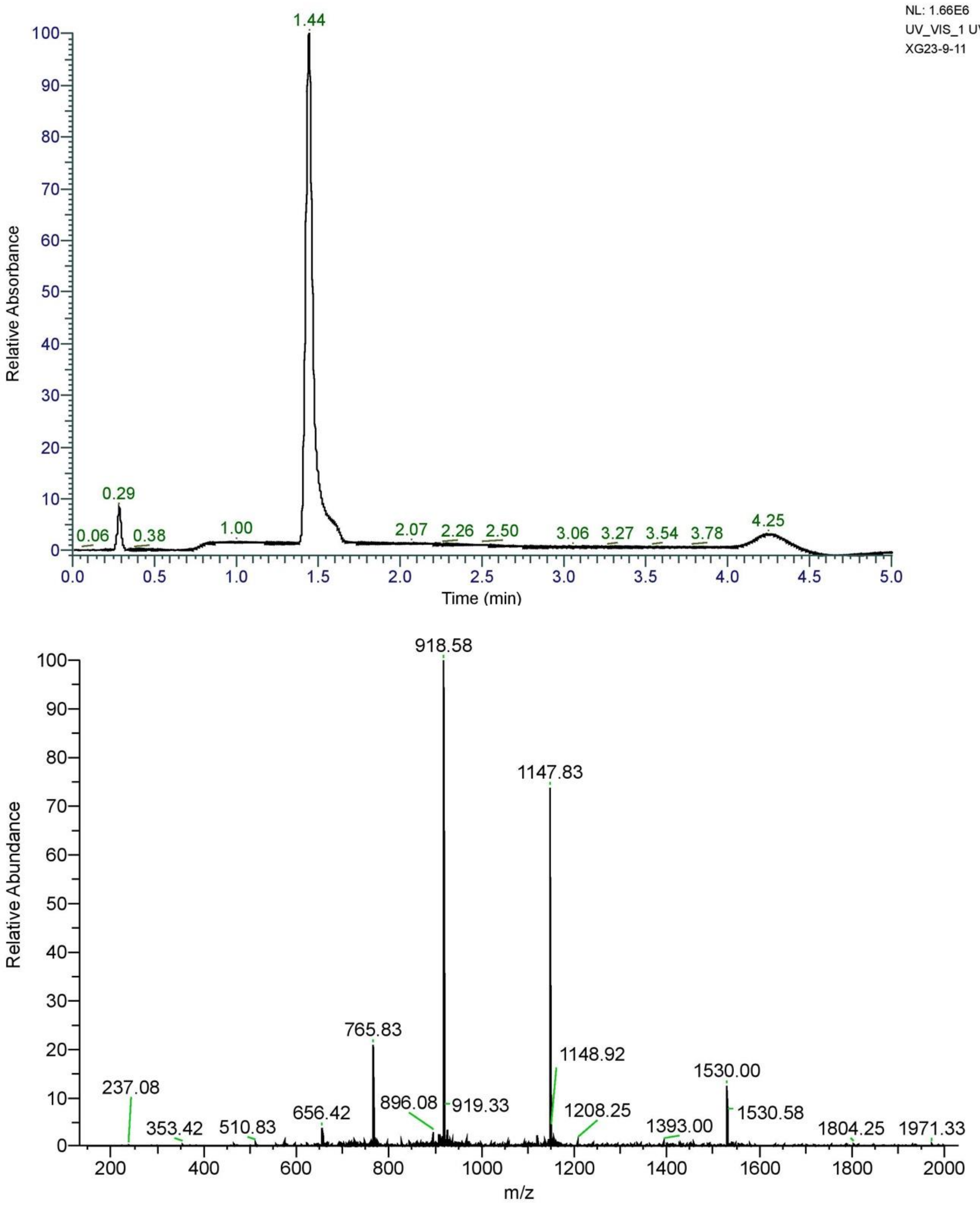

Figure S55. LCMS spectrum.

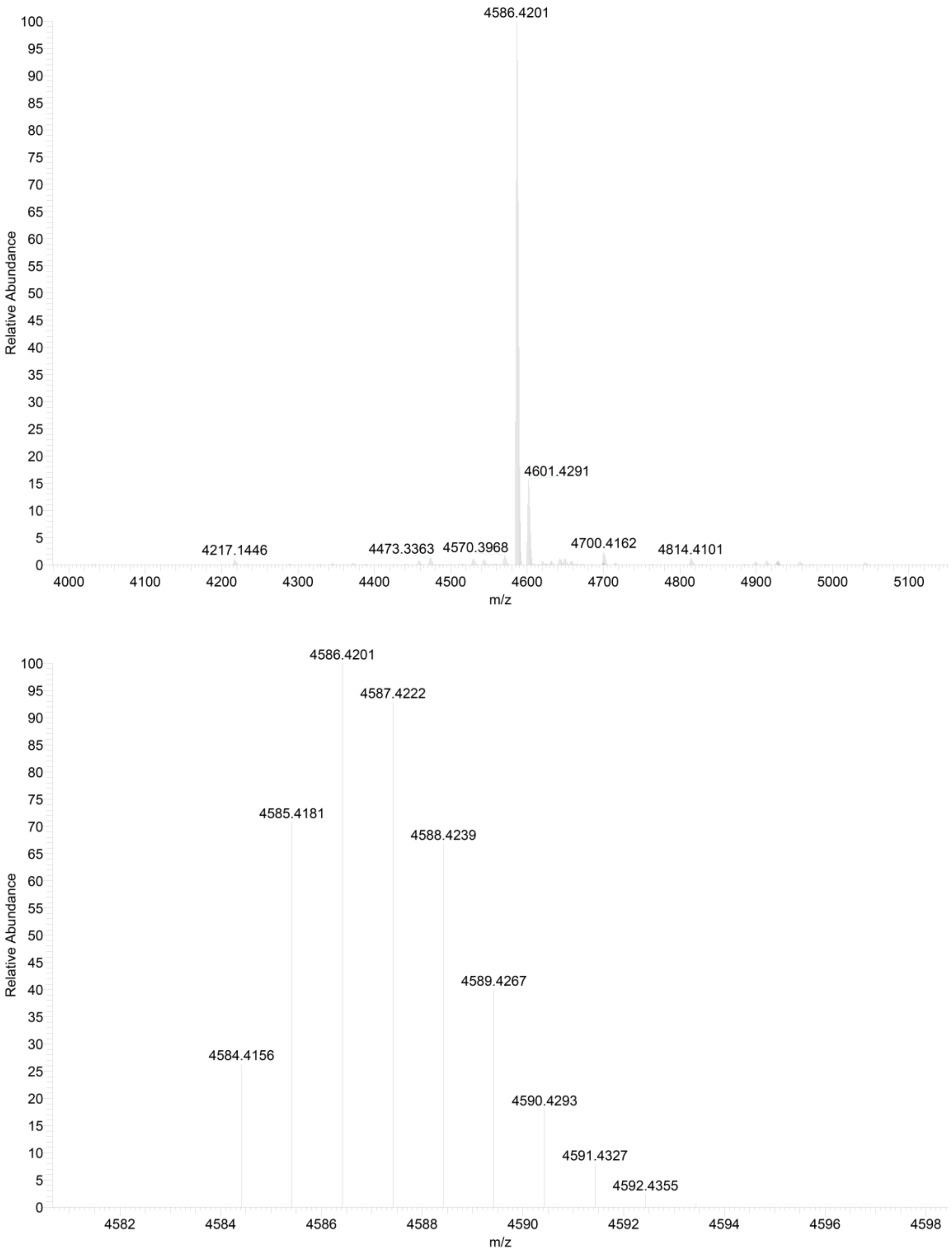

Figure S56. HRMS spectrum.

sr-**X11** ((LK)$_8$(*K*LL)$_4$(*K*KL)$_2$*K*KLL) was manually synthesized using TentaGel S RAM resin (393.4 mg, 0.09 mmol, 0.22 mmol·g^{-1}), the dendrimer was obtained as a white foamy solid after preparative RP-HPLC purification (167.5 mg, 31.0%). Analytical RP-HPLC: t_R = 1.44 min (100% A to 100% B in 3.5 min, λ= 214 nm). MS (ESI+): $C_{228}H_{439}N_{57}O_{38}$ calc./obs. 4584.42/4584.41 [M]$^+$.

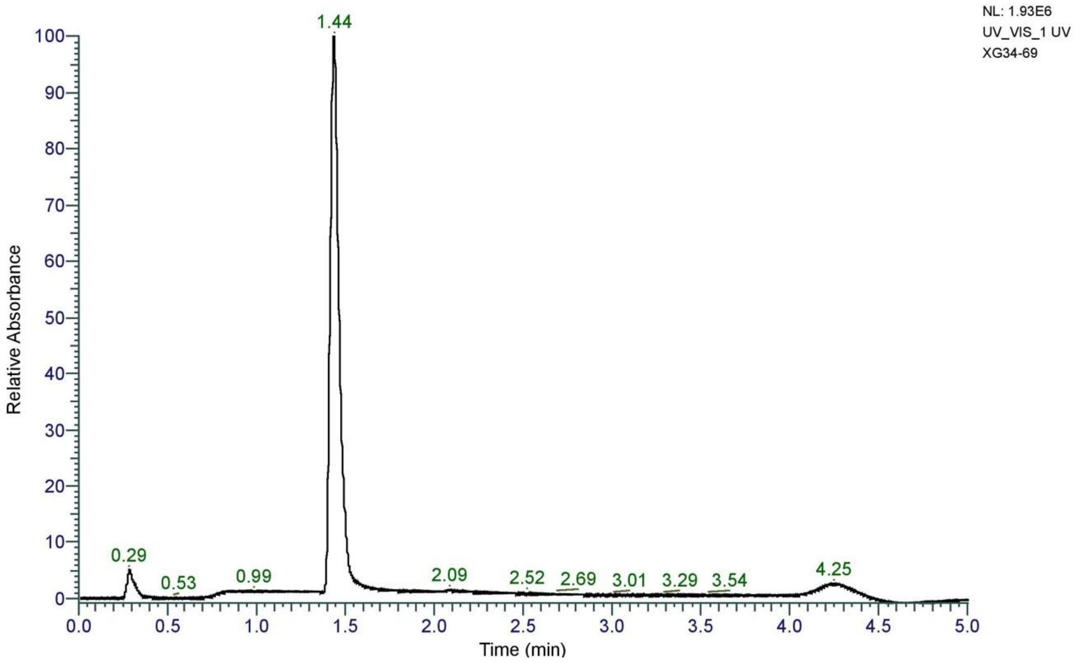

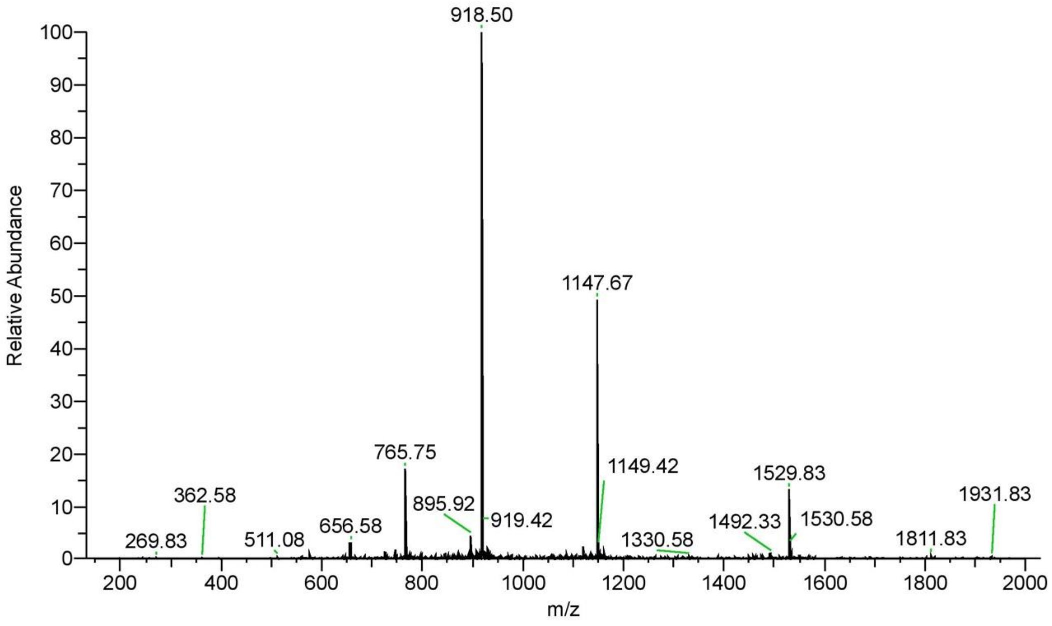

Figure S57. LCMS spectrum.

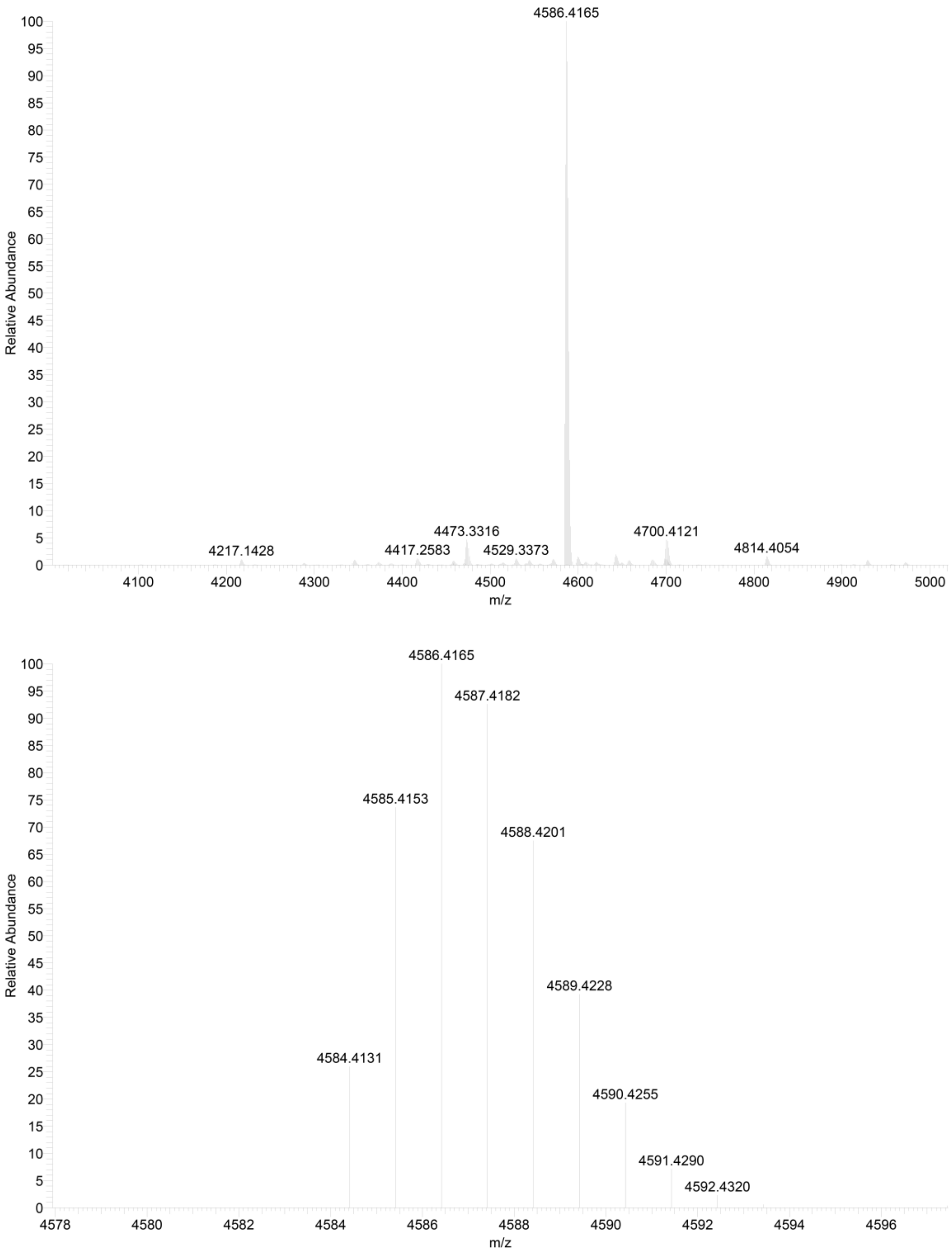

Figure S58. HRMS spectrum.

sr-**X12** ((LK)$_8$(*K*LL)$_4$(*K*KL)$_2$*K*LLK) was manually synthesized using TentaGel S RAM resin (393.4 mg, 0.09 mmol, 0.22 mmol·g^{-1}), the dendrimer was obtained as a white foamy solid after preparative RP-HPLC purification (113.9 mg, 21.1%). Analytical RP-HPLC: t_R = 1.43 min (100% A to 100% B in 3.5 min, λ= 214 nm). MS (ESI+): $C_{228}H_{439}N_{57}O_{38}$ calc./obs. 4584.42/4584.42 [M]$^+$.

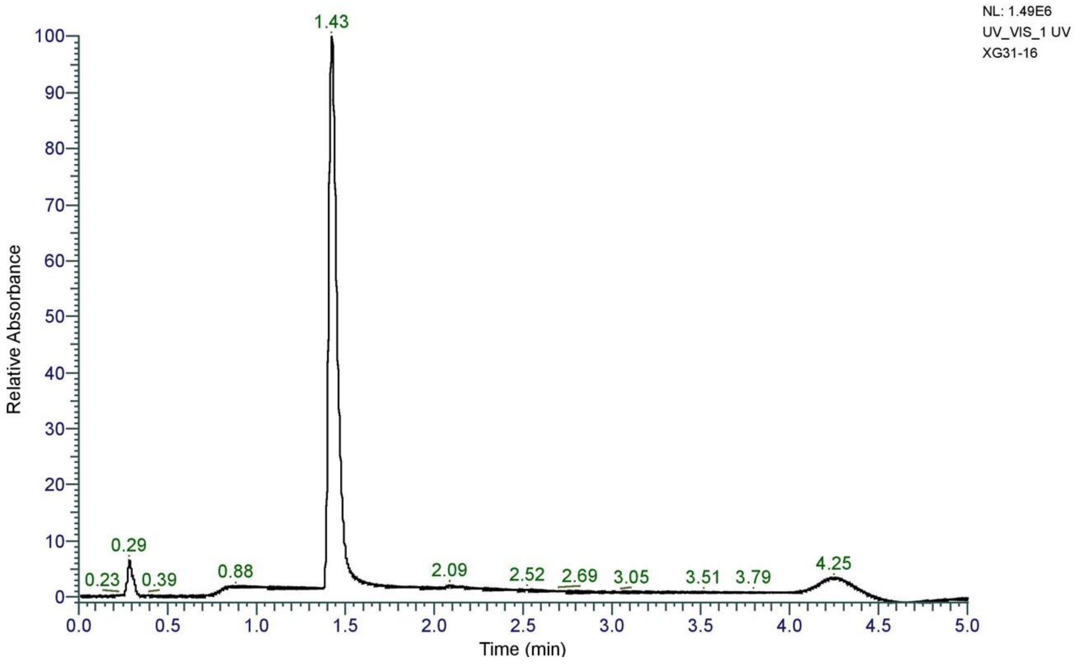

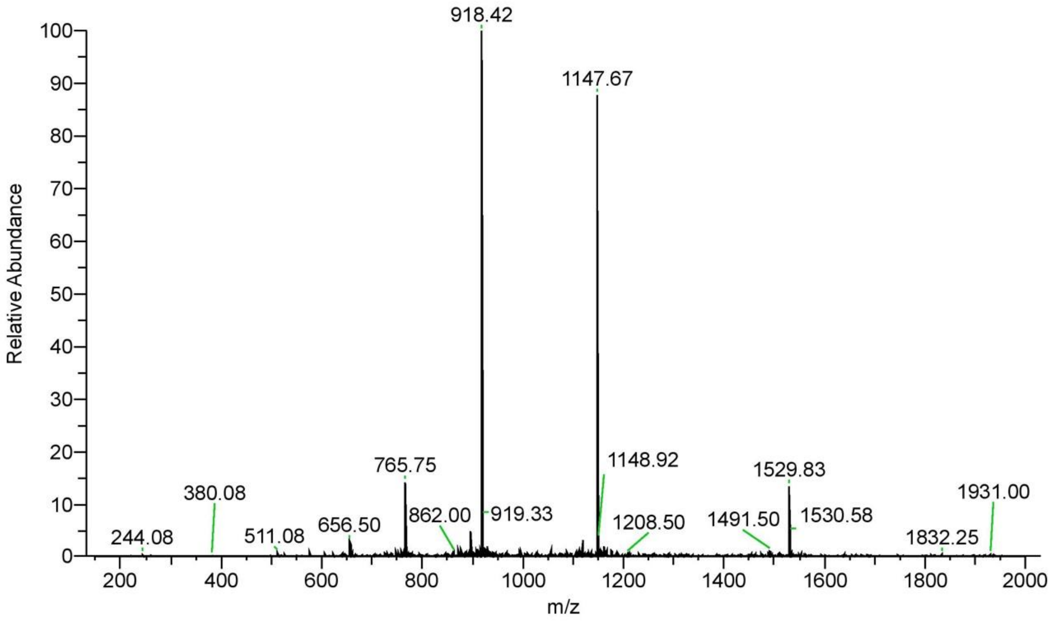

Figure S59. LCMS spectrum.

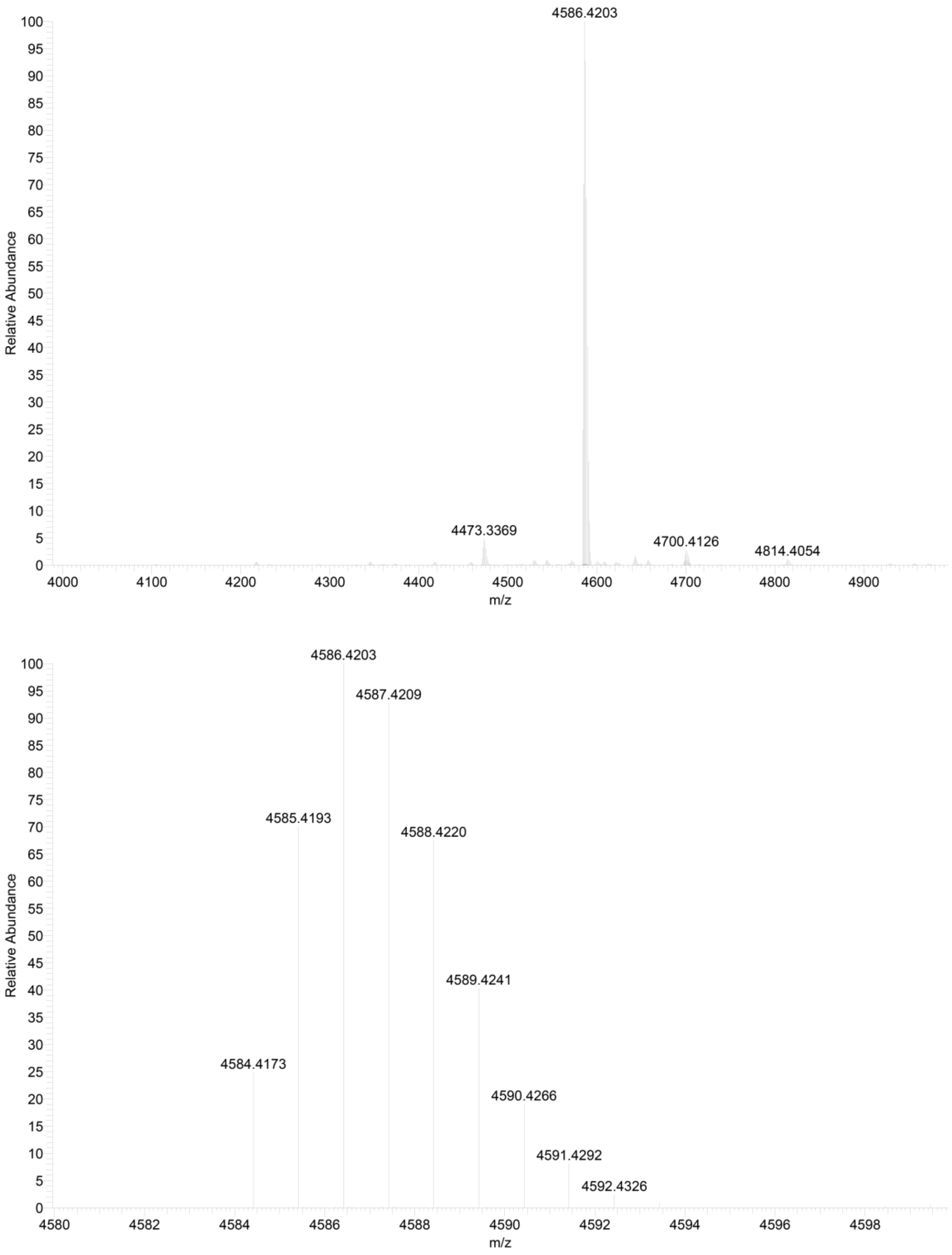

Figure S60. HRMS spectrum.

sr-**X13** ((*K*L)$_8$(*K*LL)$_4$(*K*LK)$_2$*K*LLK) was manually synthesized using TentaGel S RAM resin (393.4 mg, 0.09 mmol, 0.22 mmol·g^{-1}), the dendrimer was obtained as a white foamy solid after preparative RP-HPLC purification (118.8 mg, 22.0%). Analytical RP-HPLC: t_R = 1.46 min (100% A to 100% B in 3.5 min, λ= 214 nm). MS (ESI+): $C_{228}H_{439}N_{57}O_{38}$ calc./obs. 4584.42/4584.42 $[M]^+$.

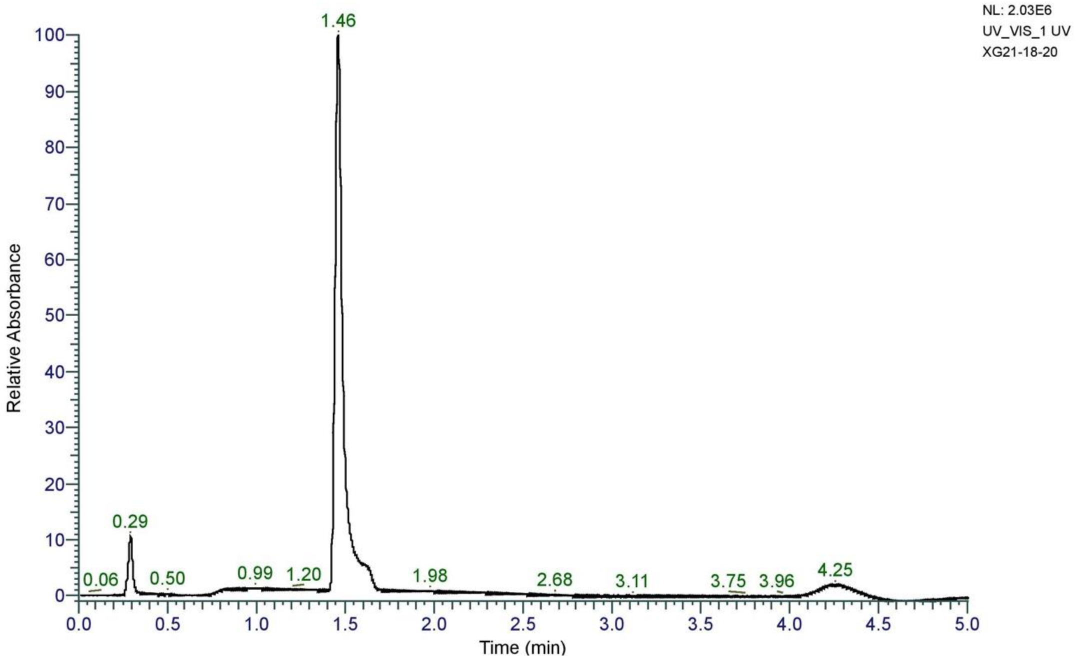

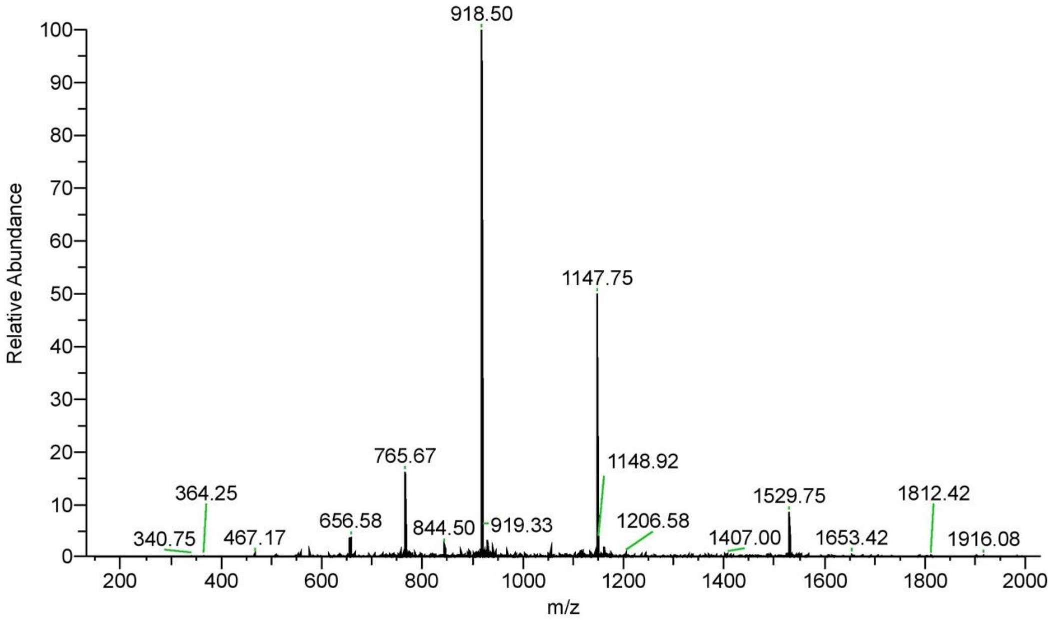

Figure S61. LCMS spectrum.

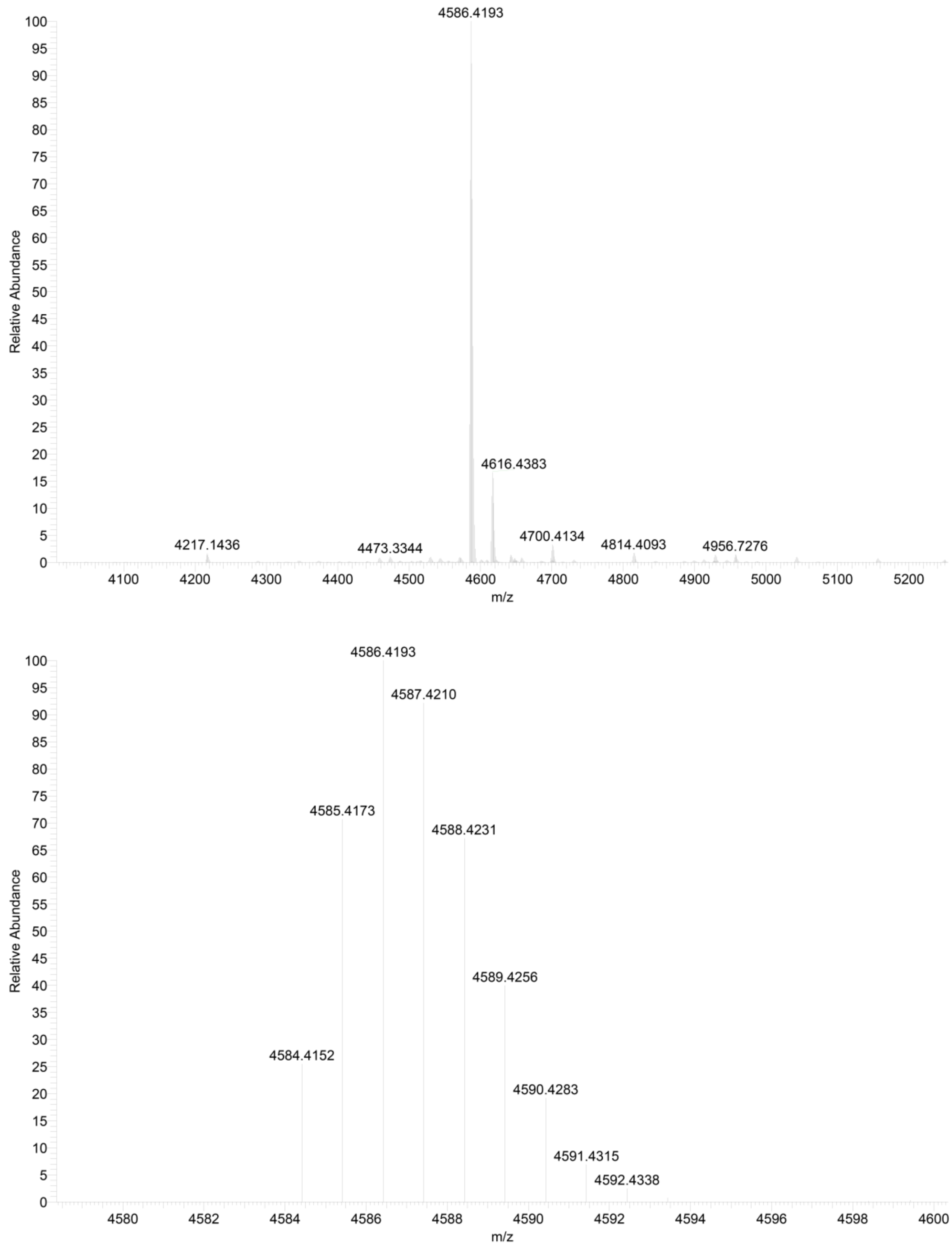

Figure S62. HRMS spectrum.

sr-**X14** ((*K*L)$_8$(*K*LL)$_4$(*K*KL)$_2$*K*KL) was synthesized by CEM Liberty Blue synthesizer using Rink Amide MBHA resin (320.0 mg, 0.08 mmol, 0.25 mmol·g^{-1}), the dendrimer was obtained as a white foamy solid after preparative RP-HPLC purification (106.2 mg, 20.0%). Analytical RP-HPLC: t_R = 1.47 min (100% A to 100% B in 3.5 min, λ= 214 nm). MS (ESI+): $C_{222}H_{428}N_{56}O_{37}$ calc./obs. 4471.33/4471.33 [M]$^+$.

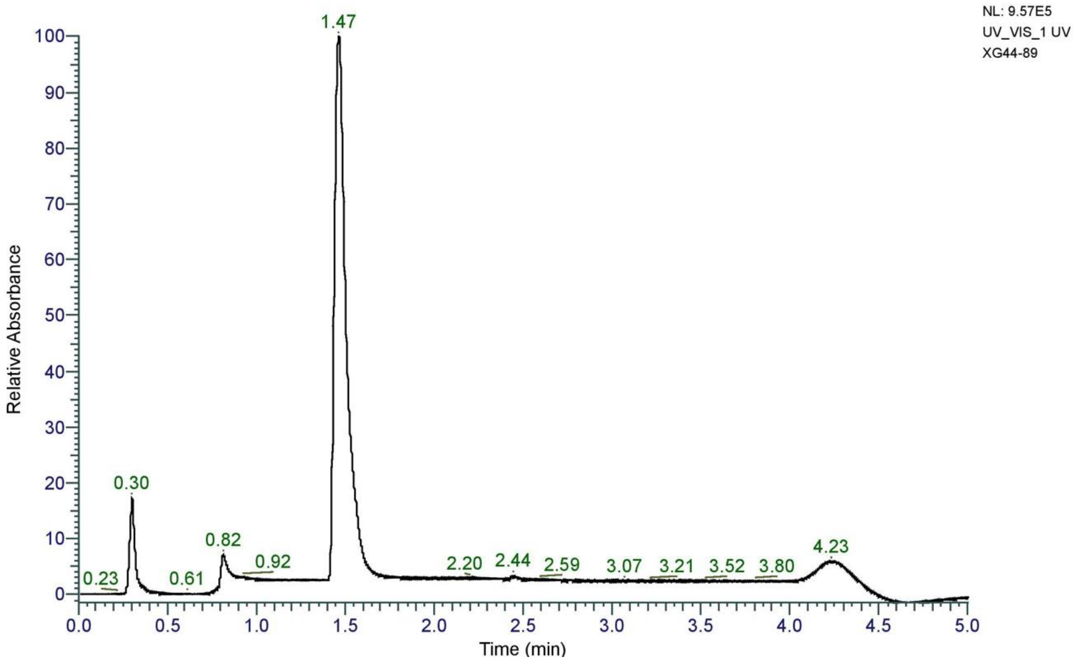

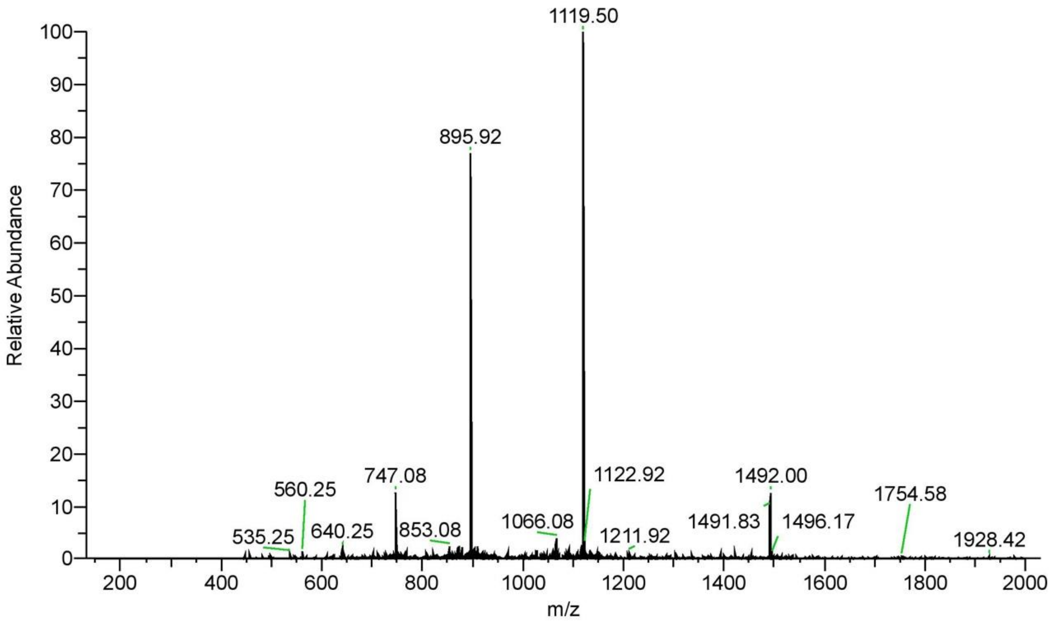

Figure S63. LCMS spectrum.

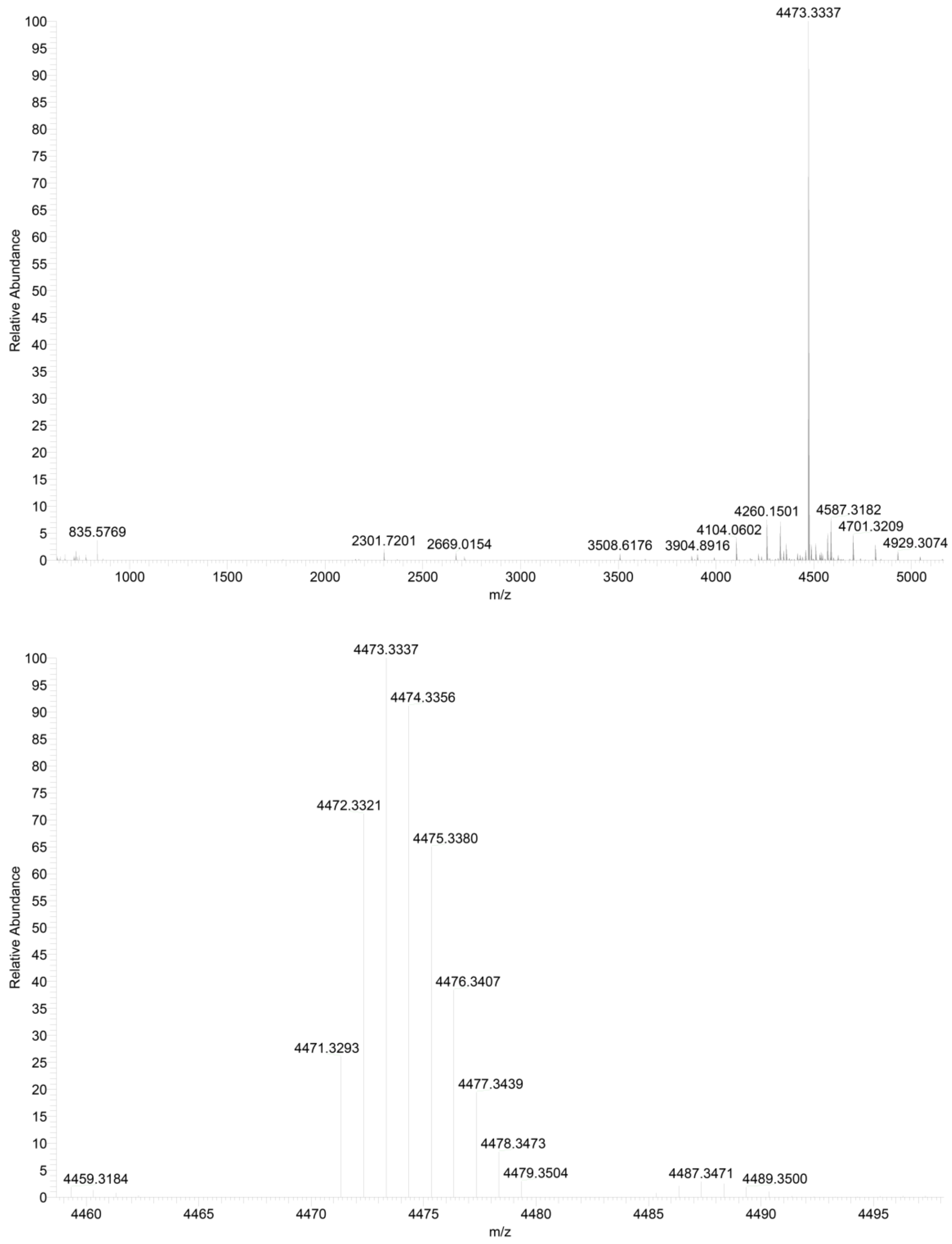

Figure S64. HRMS spectrum.

sr-**X15** ((*K*L)$_8$(*K*LL)$_4$(*K*KL)$_2$*K*K) was manually synthesized using TentaGel S RAM resin (393.4 mg, 0.09 mmol, 0.22 mmol·g^{-1}), the dendrimer was obtained as a white foamy solid after preparative RP-HPLC purification (98.4 mg, 18.9%). Analytical RP-HPLC: t_R = 1.46 min (100% A to 100% B in 3.5 min, λ= 214 nm). MS (ESI+): $C_{216}H_{417}N_{55}O_{36}$ calc./obs. 4358.25/4358.25 [M]$^+$.

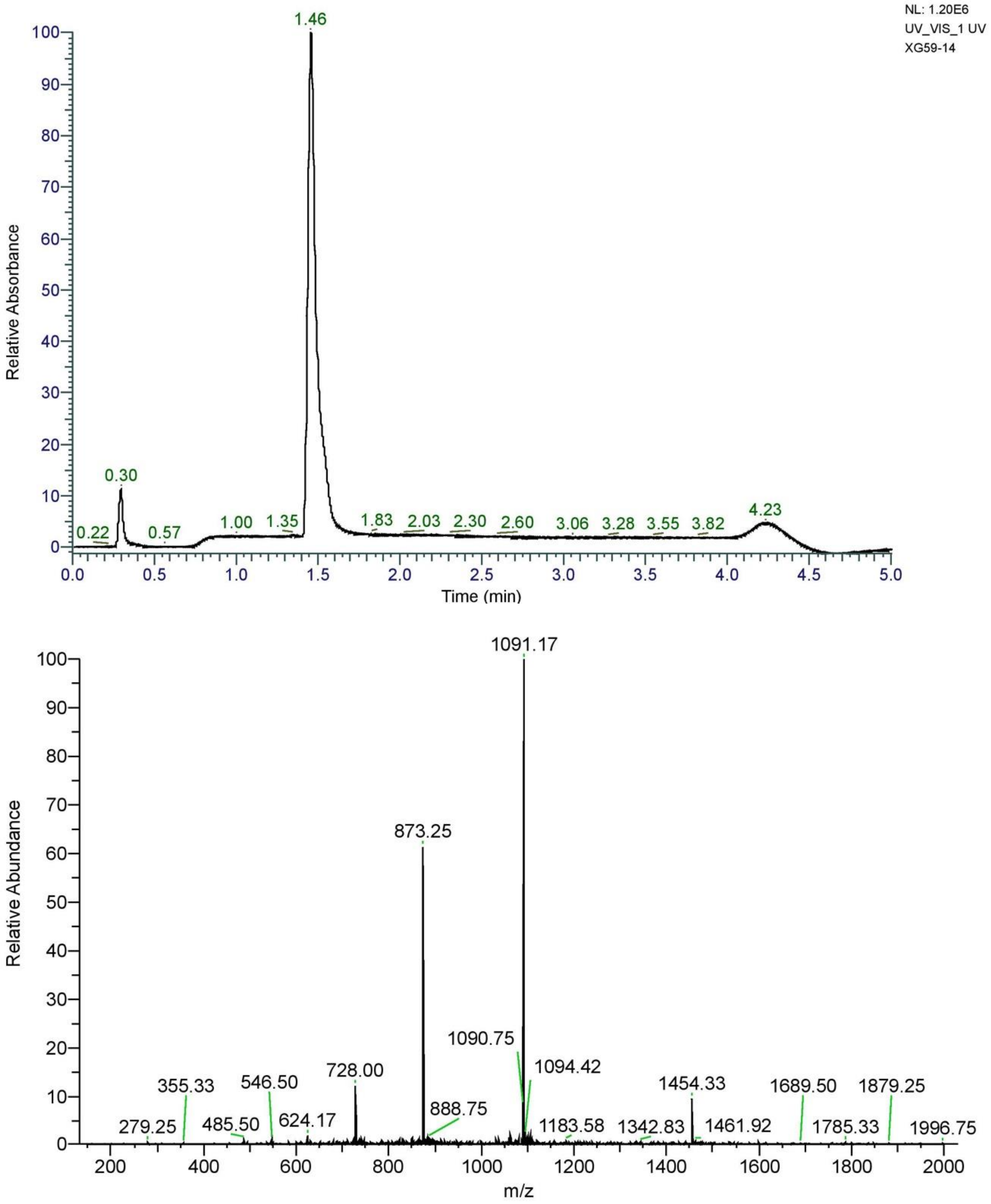

Figure S65. LCMS spectrum.

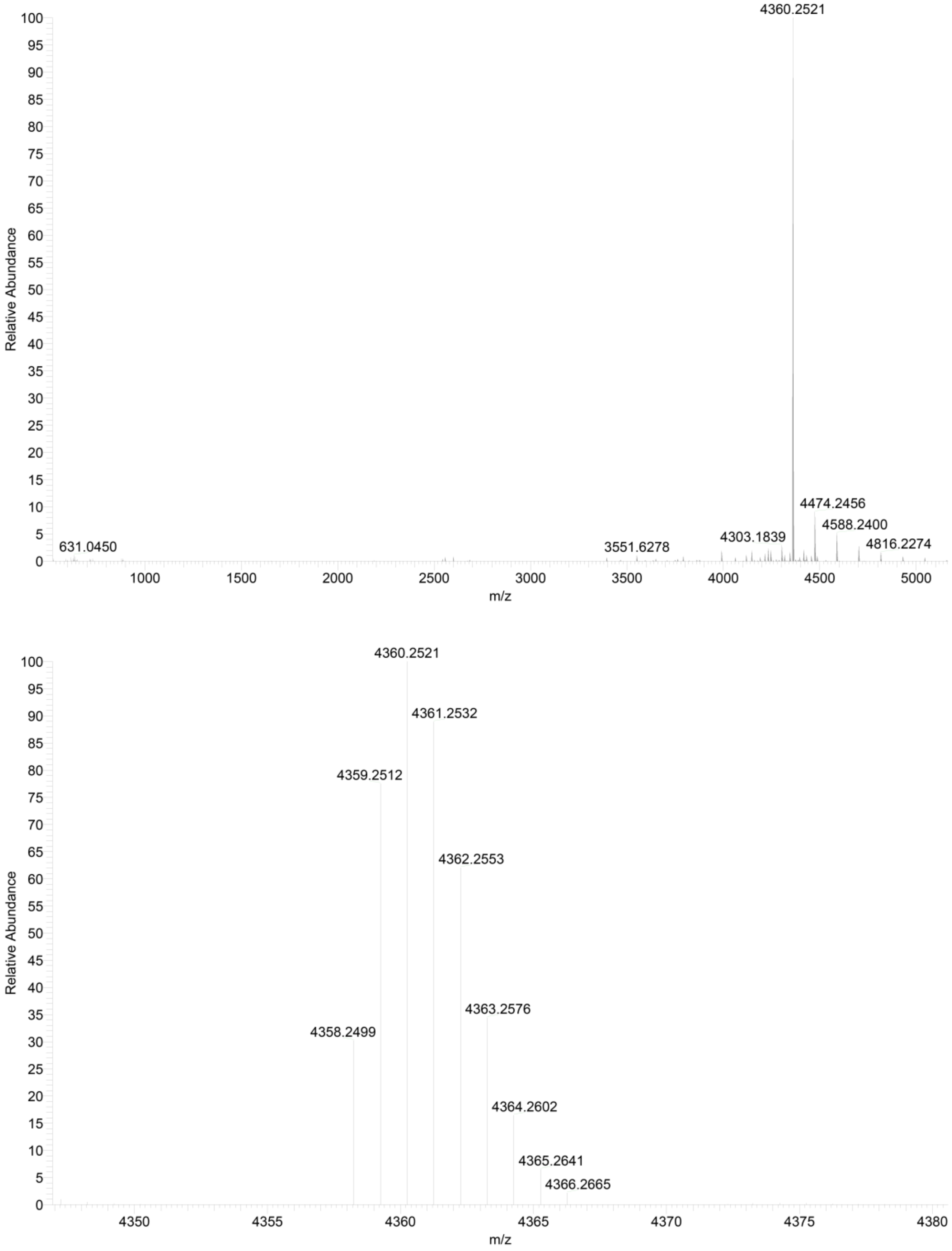

Figure S66. HRMS spectrum.

sr-**X16** ((*K*LL)$_8$(*K*K)$_4$(*K*LL)$_2$*K*KL) was manually synthesized using TentaGel S RAM resin (393.4 mg, 0.09 mmol, 0.22 mmol·g^{-1}), the dendrimer was obtained as a white foamy solid after preparative RP-HPLC purification (39.6 mg, 6.7%). Analytical RP-HPLC: t_R = 1.49 min (100% A to 100% B in 3.5 min, λ= 214 nm). MS (ESI+): $C_{246}H_{474}N_{62}O_{41}$ calc./obs. 4953.69/4953.70 $[M]^+$.

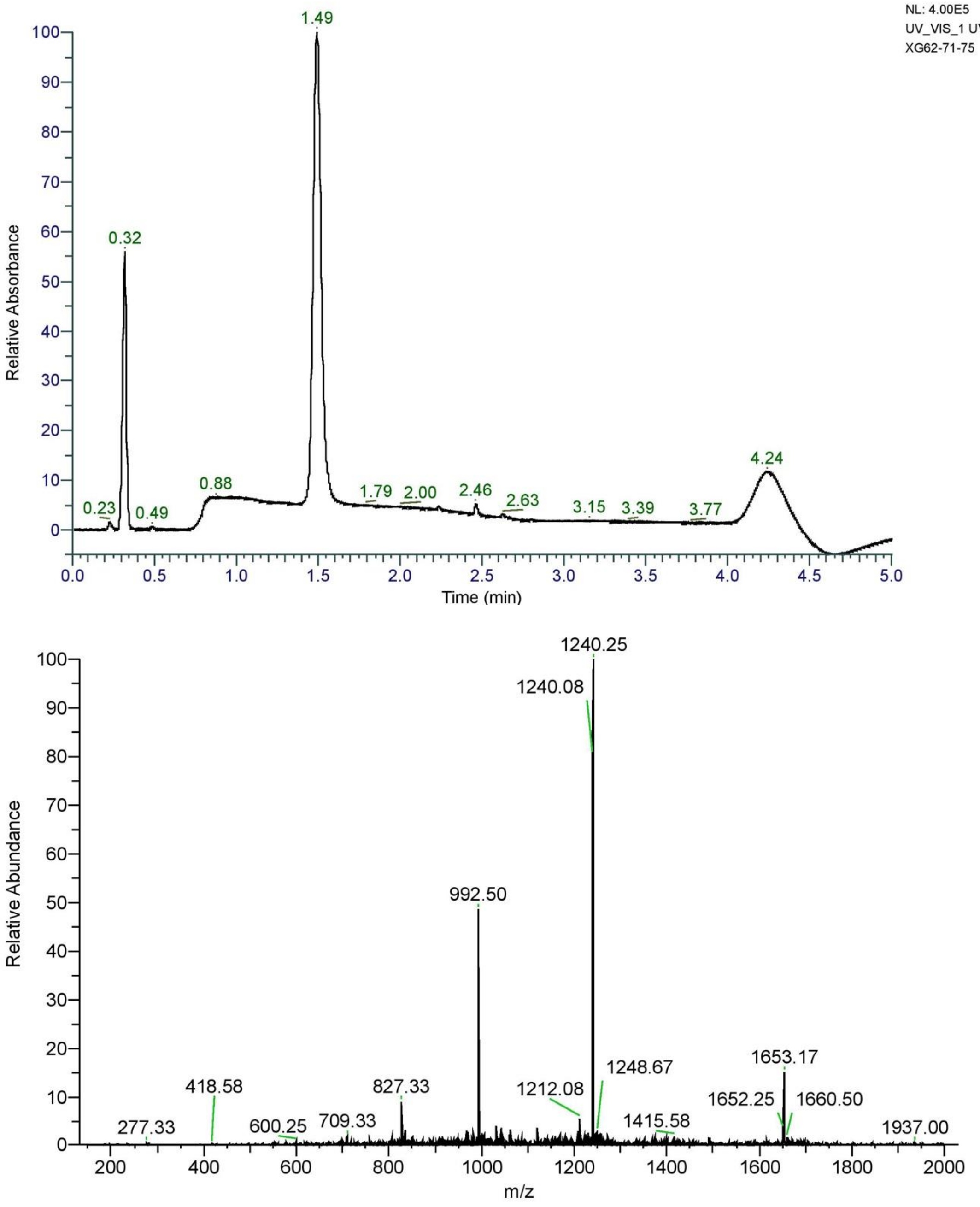

Figure S67. LCMS spectrum.

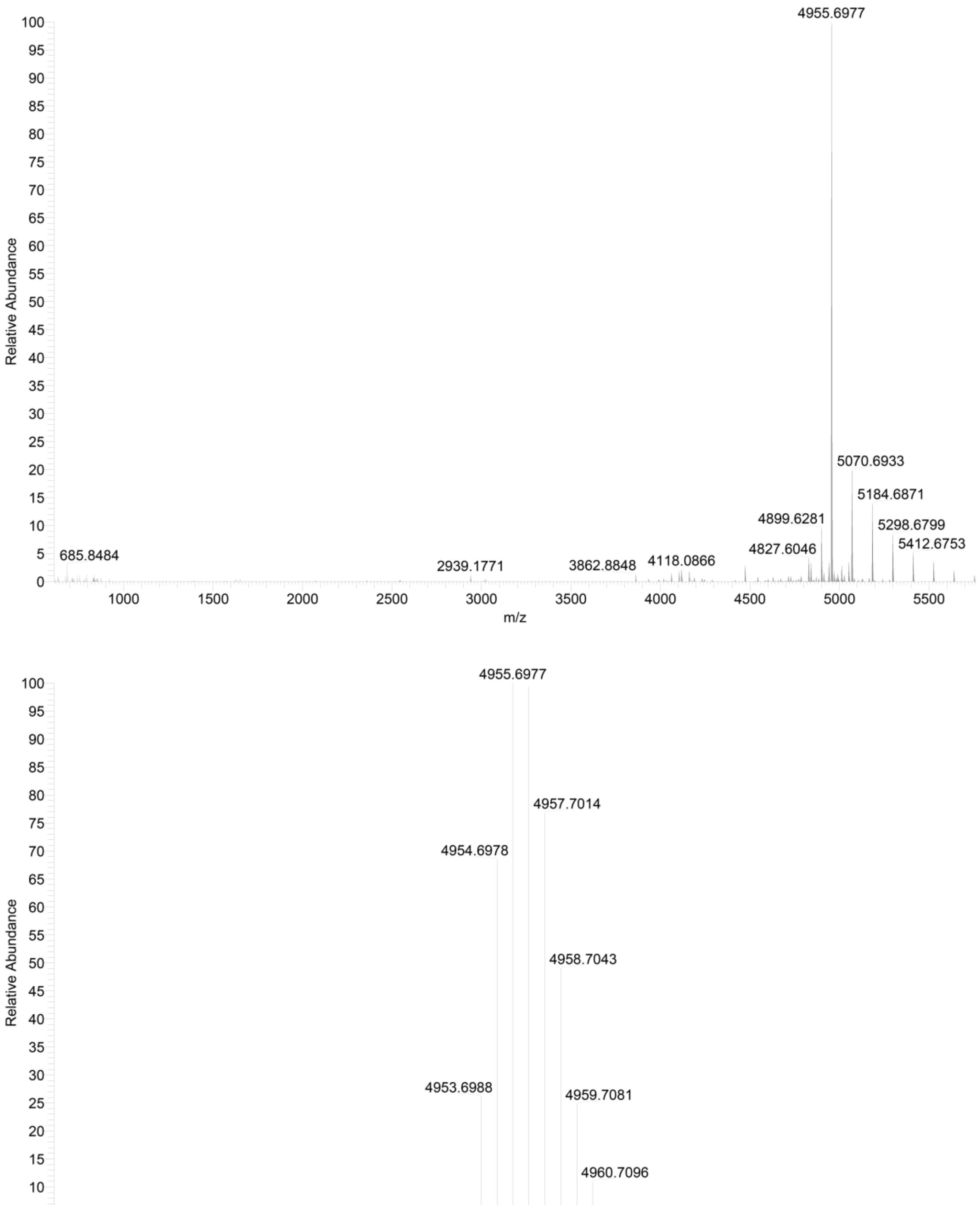

Figure S68. HRMS spectrum.

sr-**X17** ((*K*L)$_8$(*K*KL)$_4$(*K*LL)$_2$*K*LLL) was manually synthesized using TentaGel S RAM resin (393.4 mg, 0.09 mmol, 0.22 mmol·g^{-1}), the dendrimer was obtained as a white foamy solid after preparative RP-HPLC purification (131.5 mg, 23.9%). Analytical RP-HPLC: t_R = 1.43 min (100% A to 100% B in 3.5 min, λ= 214 nm). MS (ESI+): $C_{228}H_{440}N_{58}O_{38}$ calc./obs. 4599.43/4599.43 [M]$^+$.

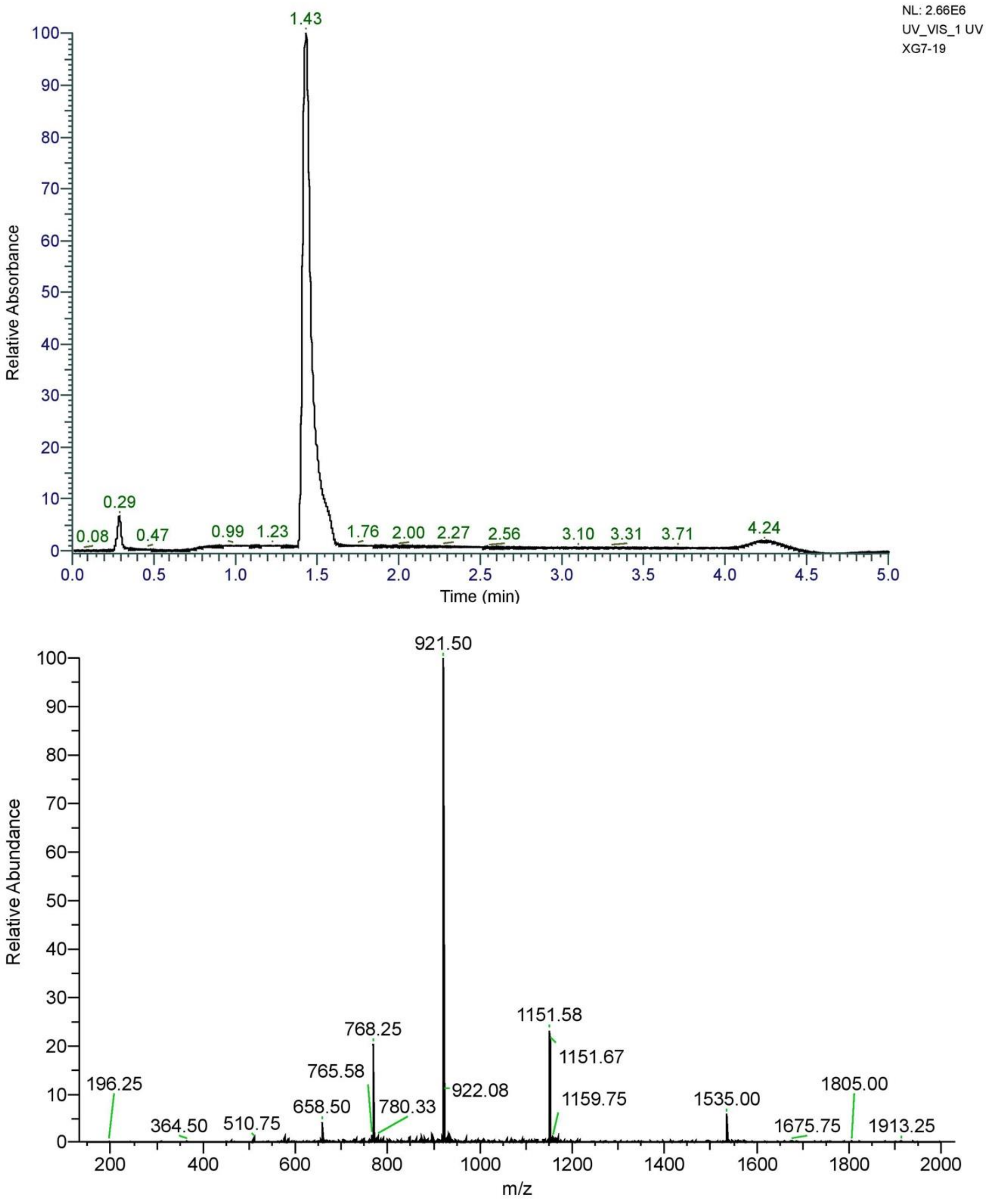

Figure S69. LCMS spectrum.

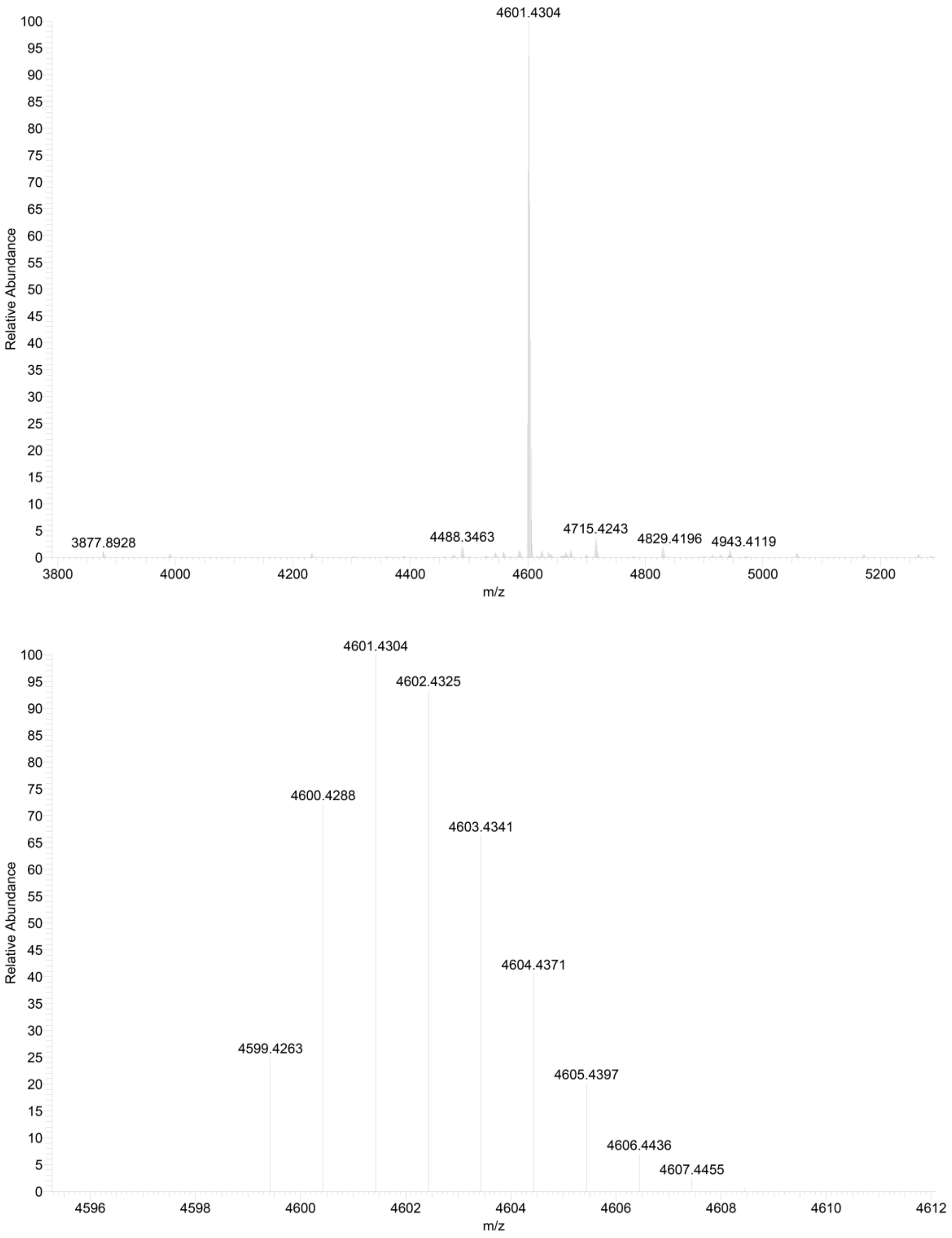

Figure S70. HRMS spectrum.

sr-**X18** ((*K*L)$_8$(*K*LK)$_4$(*K*LL)$_2$*K*LLL) was synthesized by CEM Liberty Blue synthesizer using Rink Amide MBHA resin (320.0 mg, 0.08 mmol, 0.25 mmol·g^{-1}), the dendrimer was obtained as a white foamy solid after preparative RP-HPLC purification (209.5 mg, 38.1%). Analytical RP-HPLC: t_R = 1.45 min (100% A to 100% B in 3.5 min, λ= 214 nm). MS (ESI+): $C_{228}H_{440}N_{58}O_{38}$ calc./obs. 4599.43/4599.43 [M]$^+$.

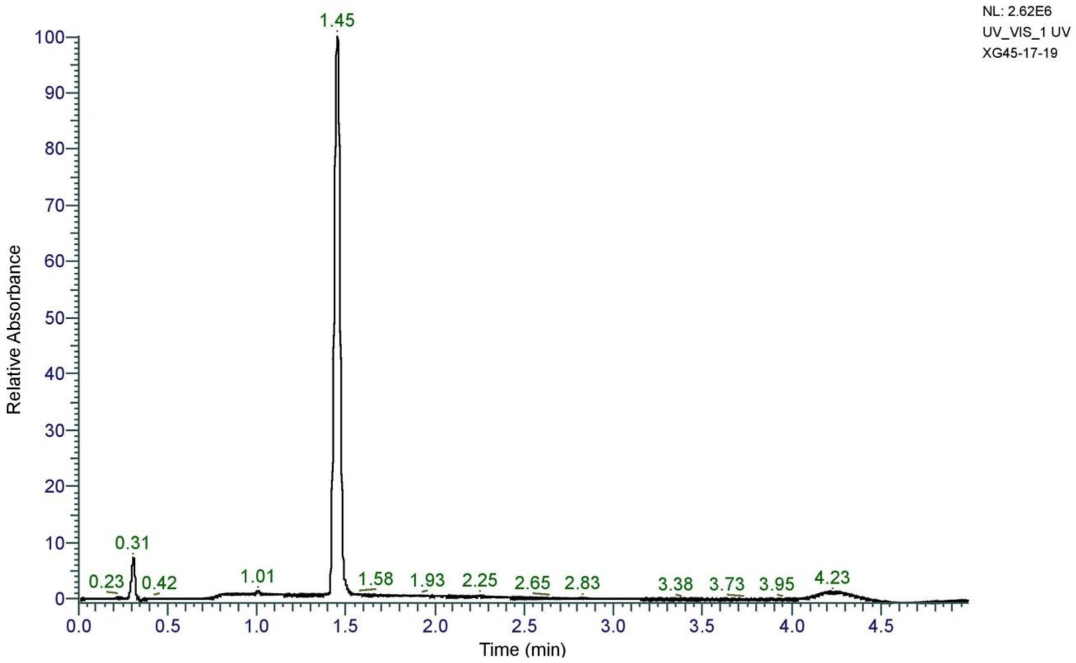

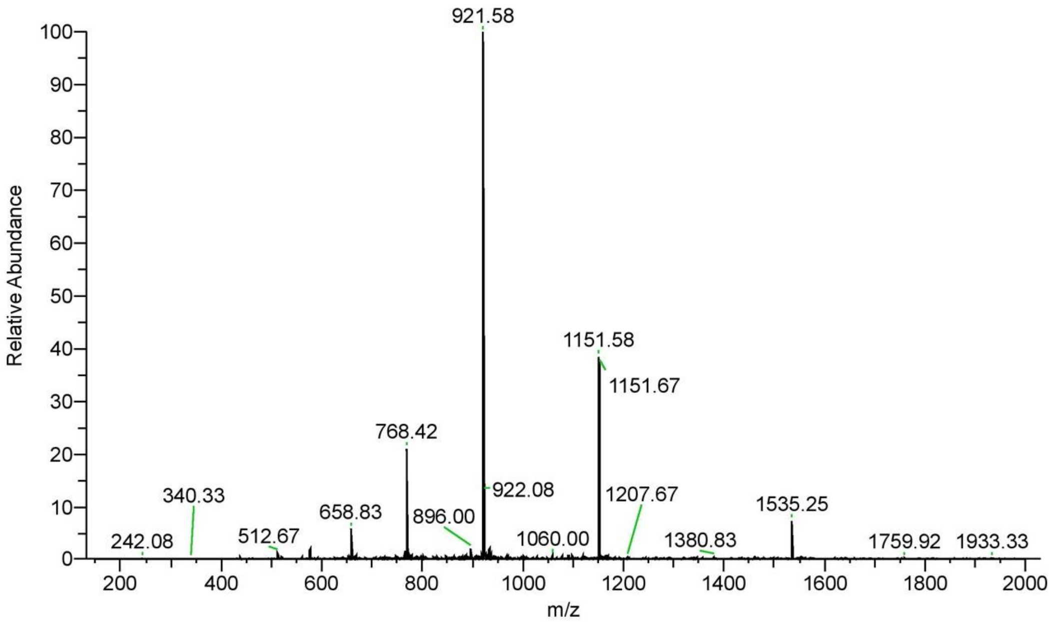

Figure S71. LCMS spectrum.

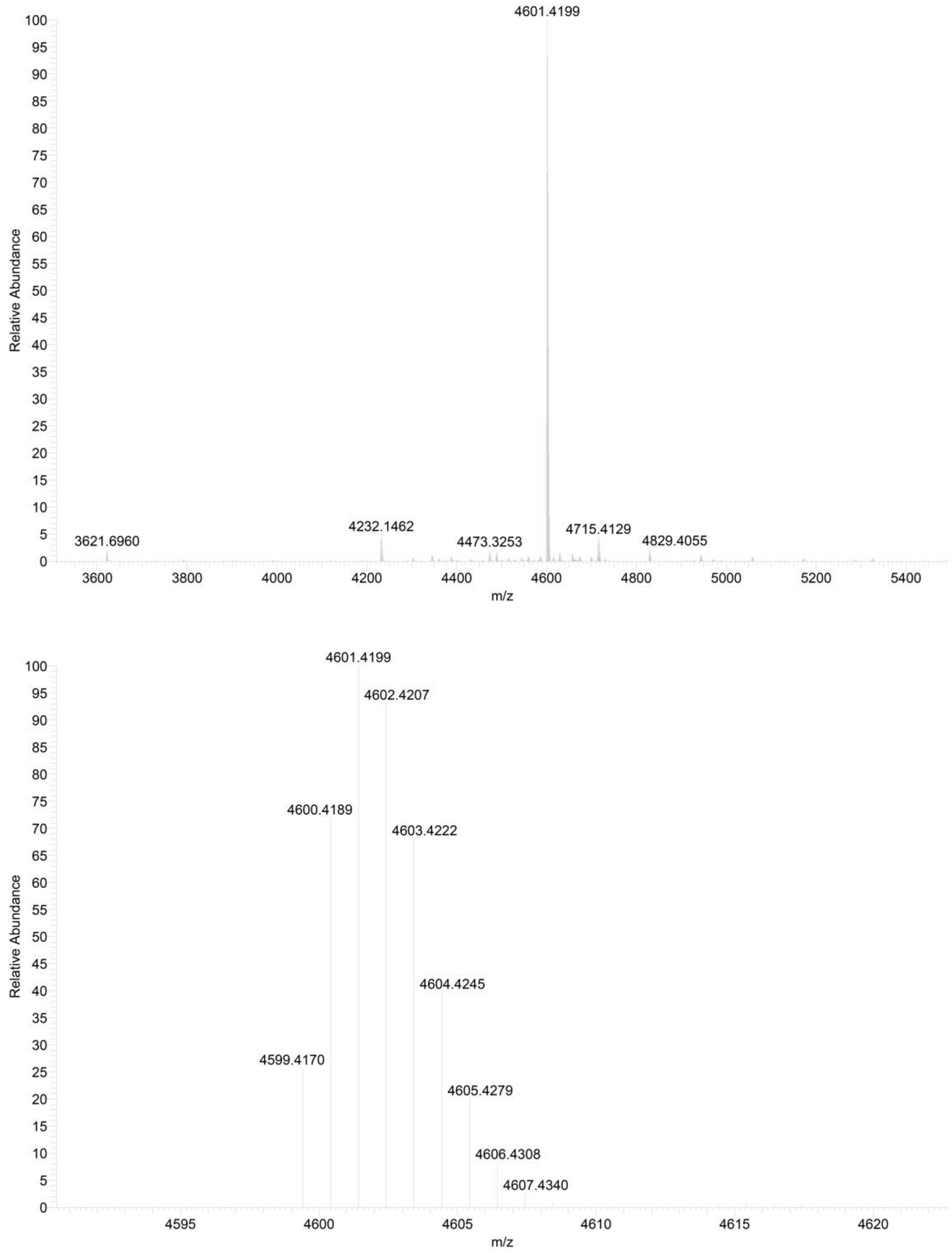

Figure S72. HRMS spectrum.

sr-**X19** ((*K*L)$_8$(*K*LL)$_4$(*K*KL)$_2$*K*KKL) was manually synthesized using TentaGel S RAM resin (393.4 mg, 0.09 mmol, 0.22 mmol·g^{-1}), the dendrimer was obtained as a white foamy solid after preparative RP-HPLC purification (141.0 mg, 25.6%). Analytical RP-HPLC: t_R = 1.43 min (100% A to 100% B in 3.5 min, λ= 214 nm). MS (ESI+): $C_{228}H_{440}N_{58}O_{38}$ calc./obs. 4599.43/4599.43 $[M]^+$.

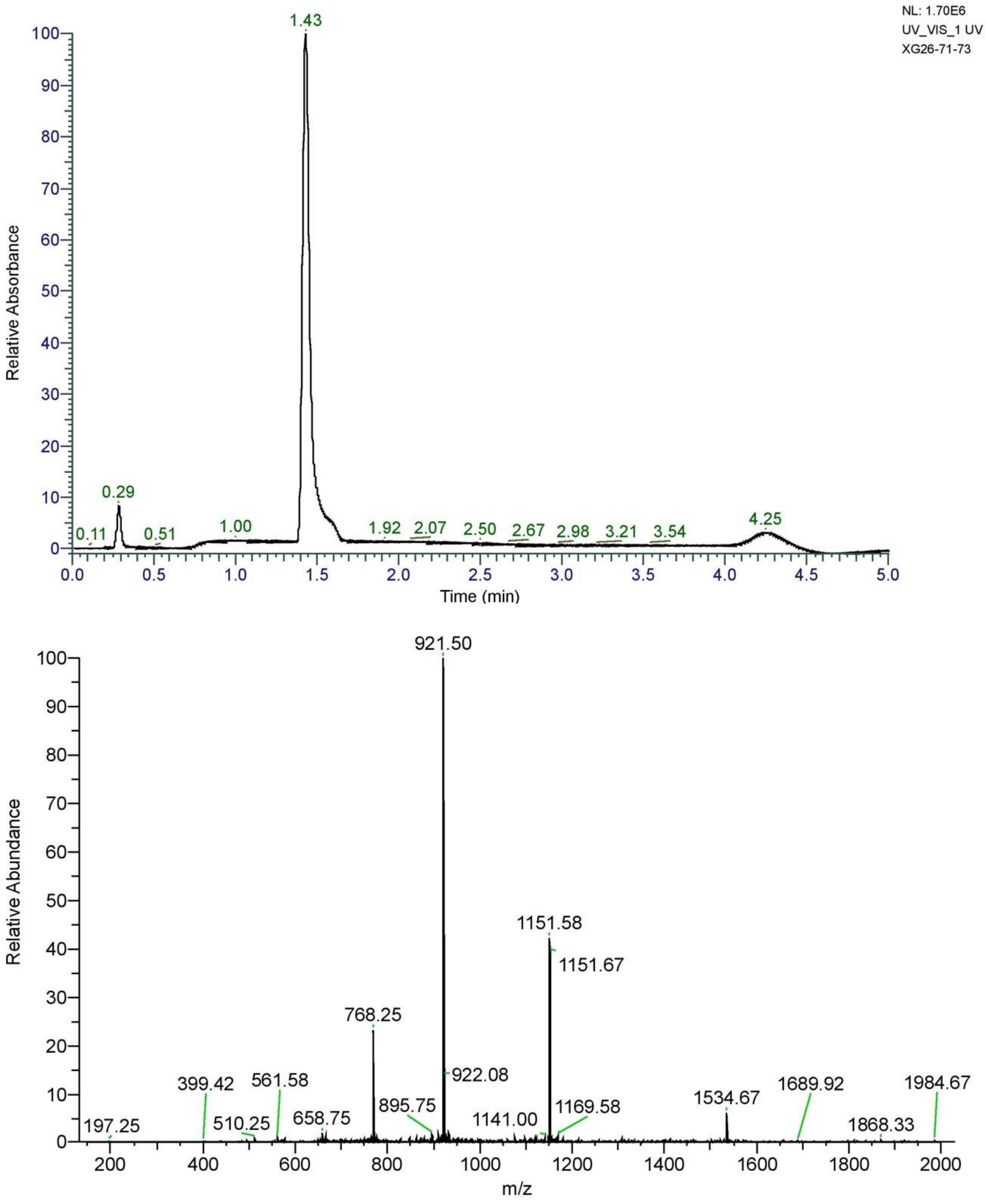

Figure S73. LCMS spectrum.

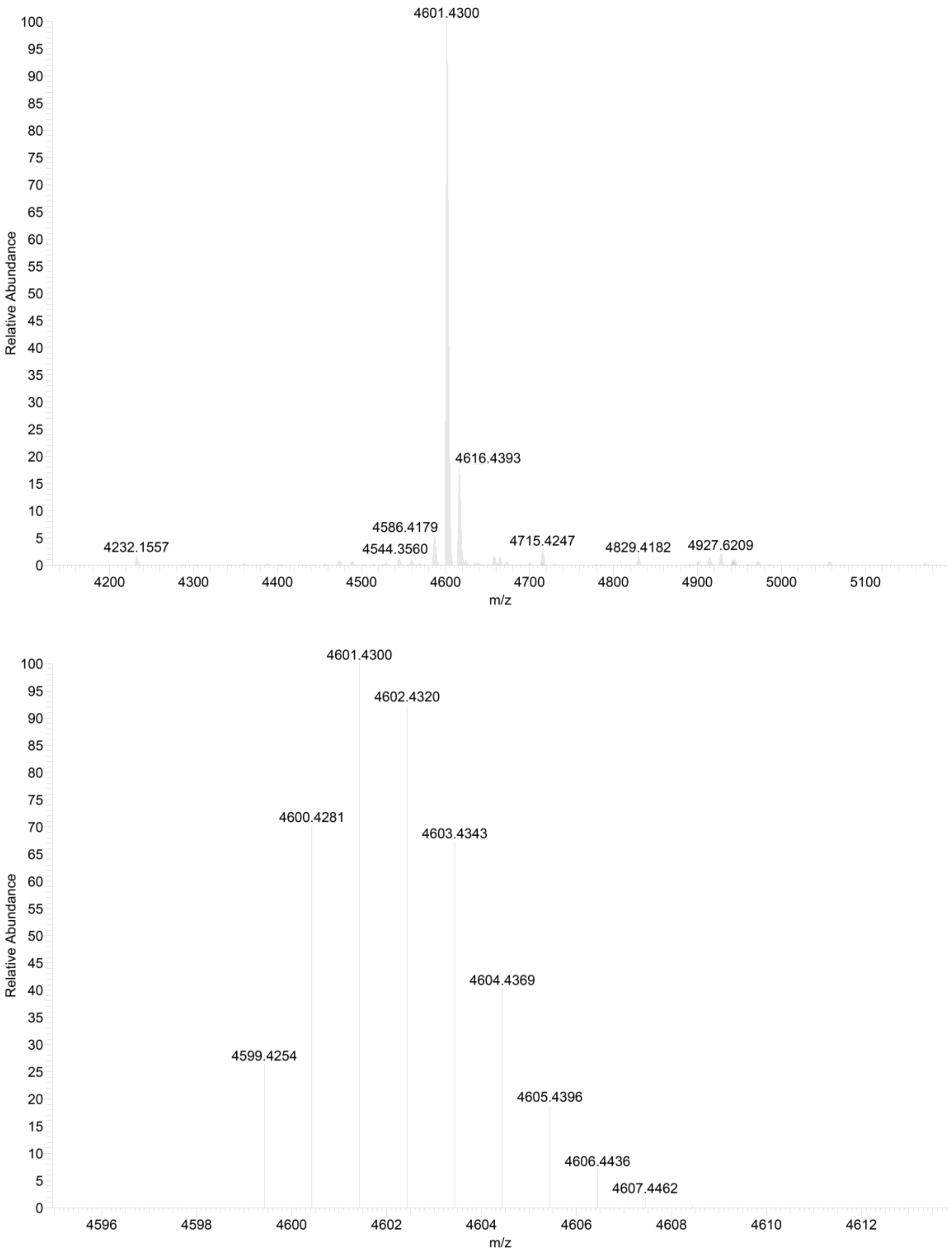

Figure S74. HRMS spectrum.

sr-**X20** ((LK)$_8$(*K*LK)$_4$(*K*LL)$_2$*K*LLL) was synthesized by CEM Liberty Blue synthesizer using Rink Amide MBHA resin (363.6 mg, 0.09 mmol, 0.25 mmol·g^{-1}), the dendrimer was obtained as a white foamy solid after preparative RP-HPLC purification (157.5 mg, 25.2%). Analytical RP-HPLC: t_R = 1.42 min (100% A to 100% B in 3.5 min, λ= 214 nm). MS (ESI+): $C_{228}H_{440}N_{58}O_{38}$ calc./obs. 4599.43/4599.42 [M]$^+$.

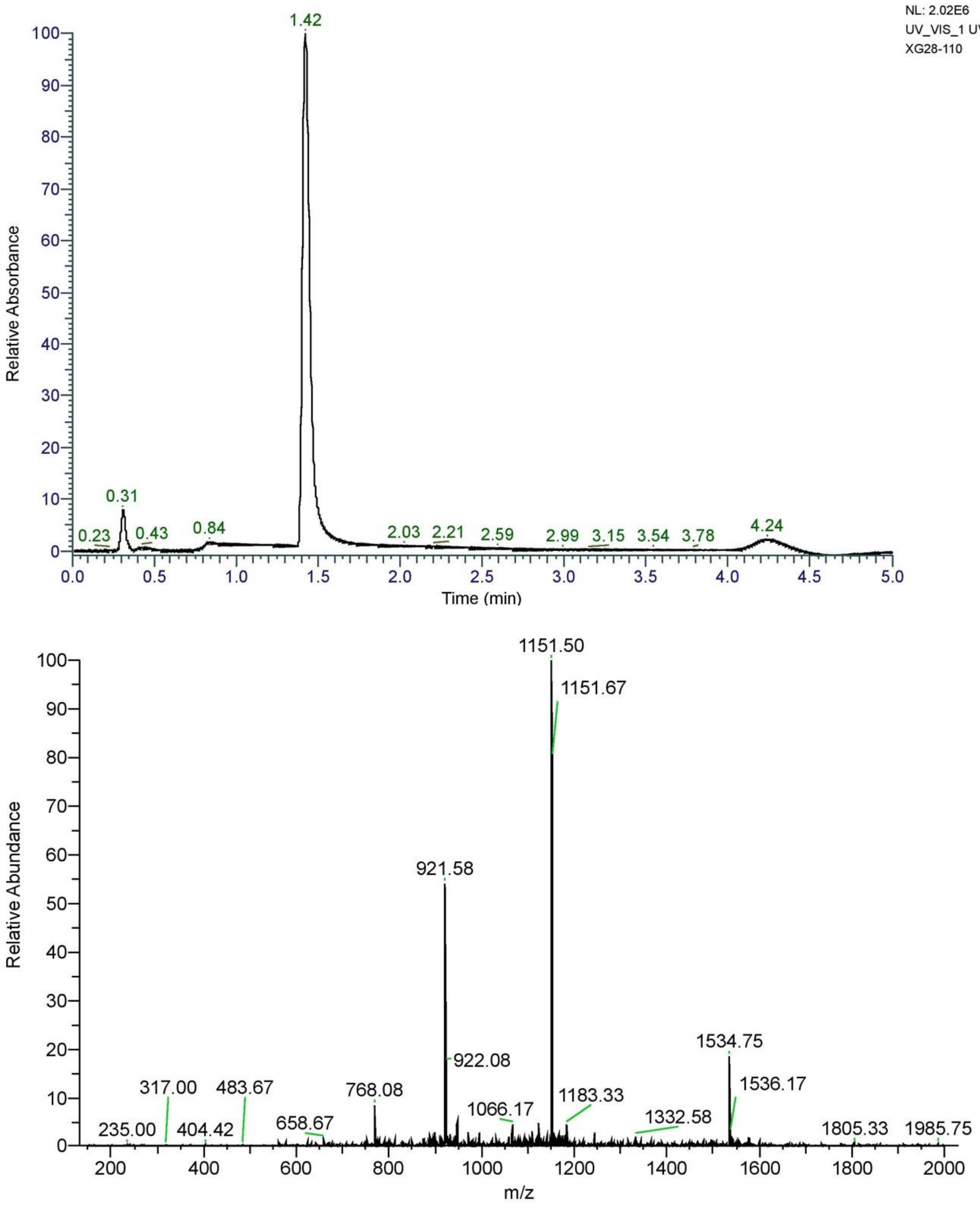

Figure S75. LCMS spectrum.

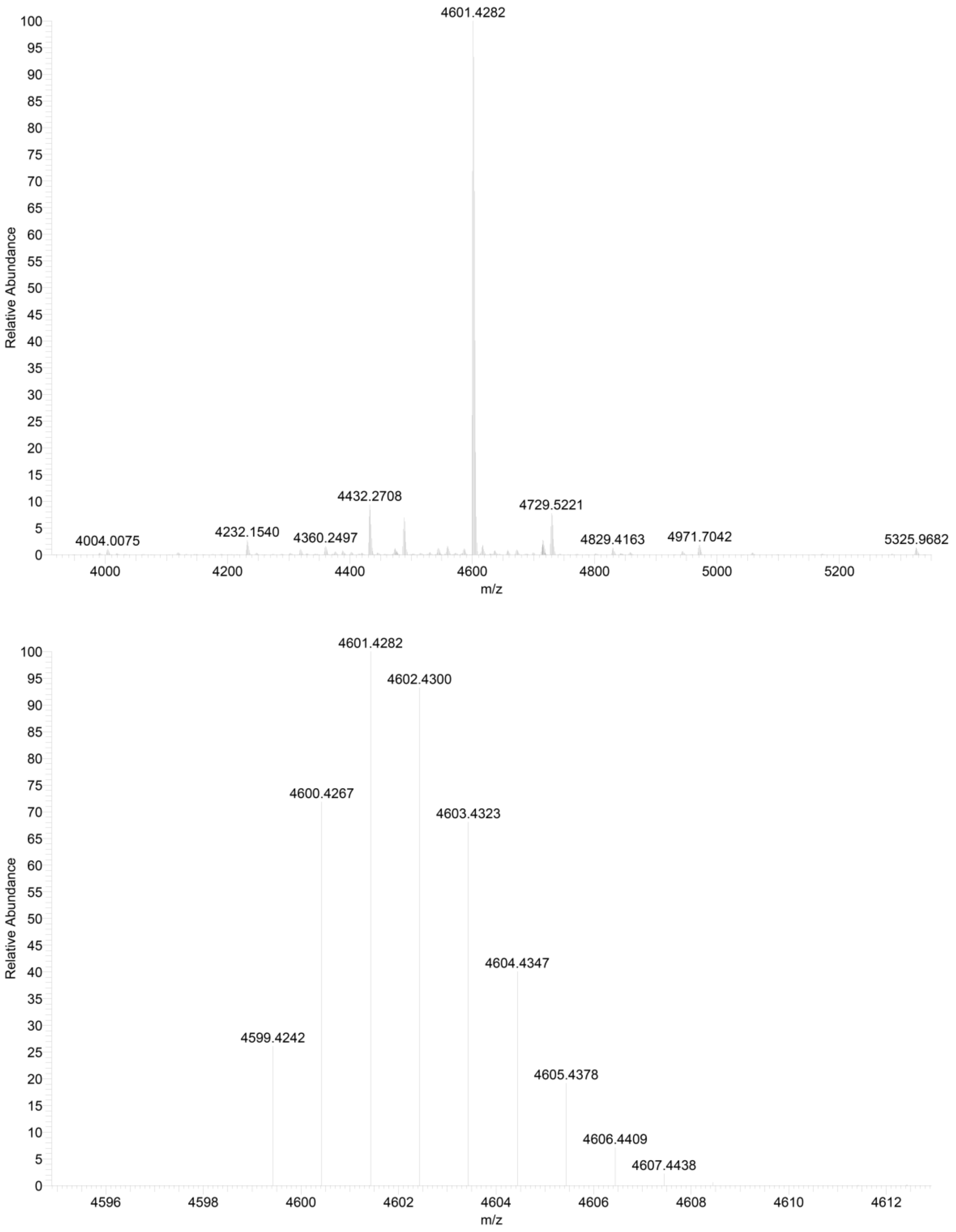

Figure S76. HRMS spectrum.

sr-**X21** ((*K*L)$_8$(*K*KLL)$_4$(*K*L)$_2$*K*KL) was manually synthesized using TentaGel S RAM resin (393.4 mg, 0.09 mmol, 0.22 mmol·g^{-1}), the dendrimer was obtained as a white foamy solid after preparative RP-HPLC purification (106.9 mg, 18.8%). Analytical RP-HPLC: t_R = 1.44 min (100% A to 100% B in 3.5 min, λ= 214 nm). MS (ESI+): $C_{234}H_{452}N_{60}O_{39}$ calc./obs. 4727.52/4727.53 [M]$^+$.

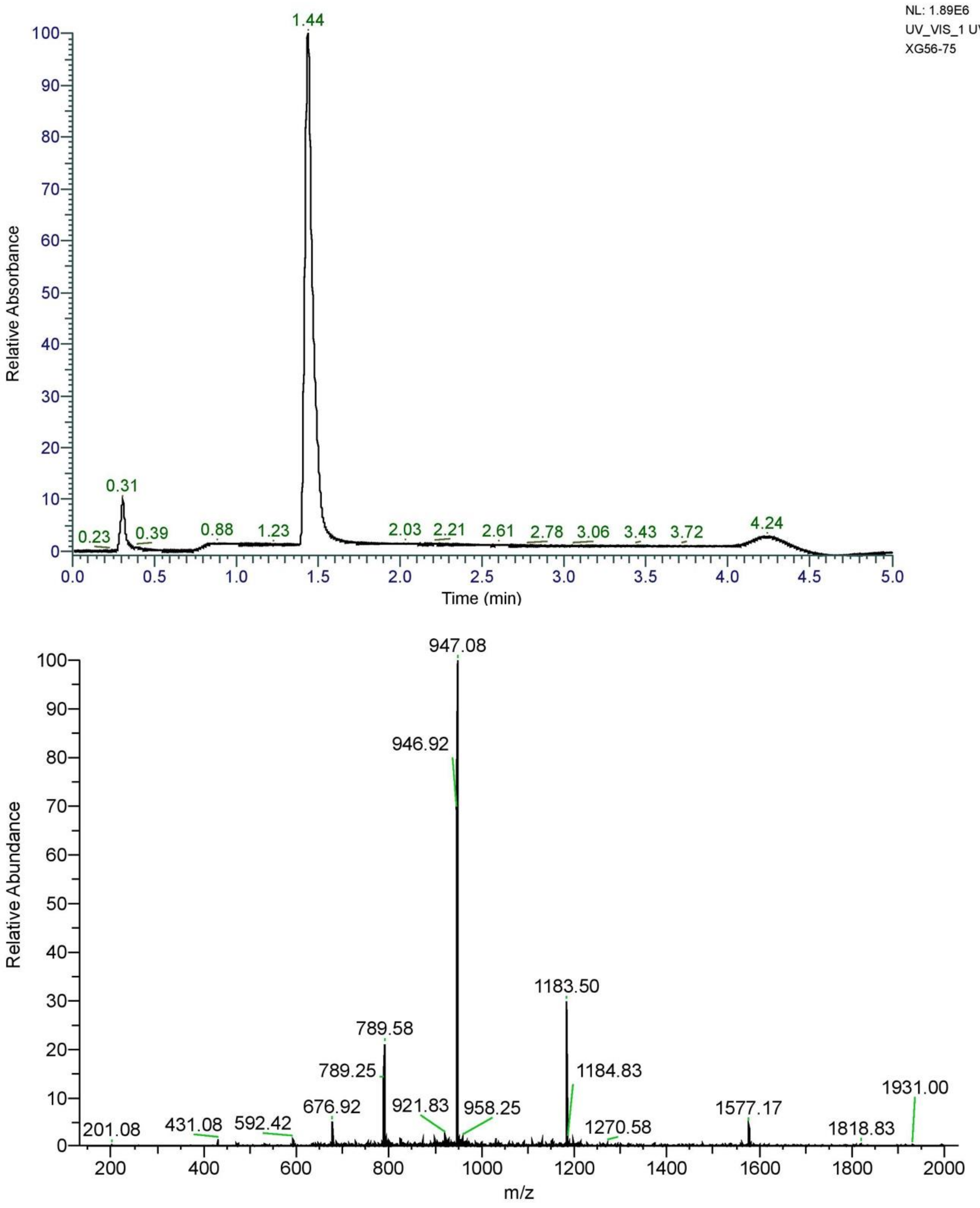

Figure S77. LCMS spectrum.

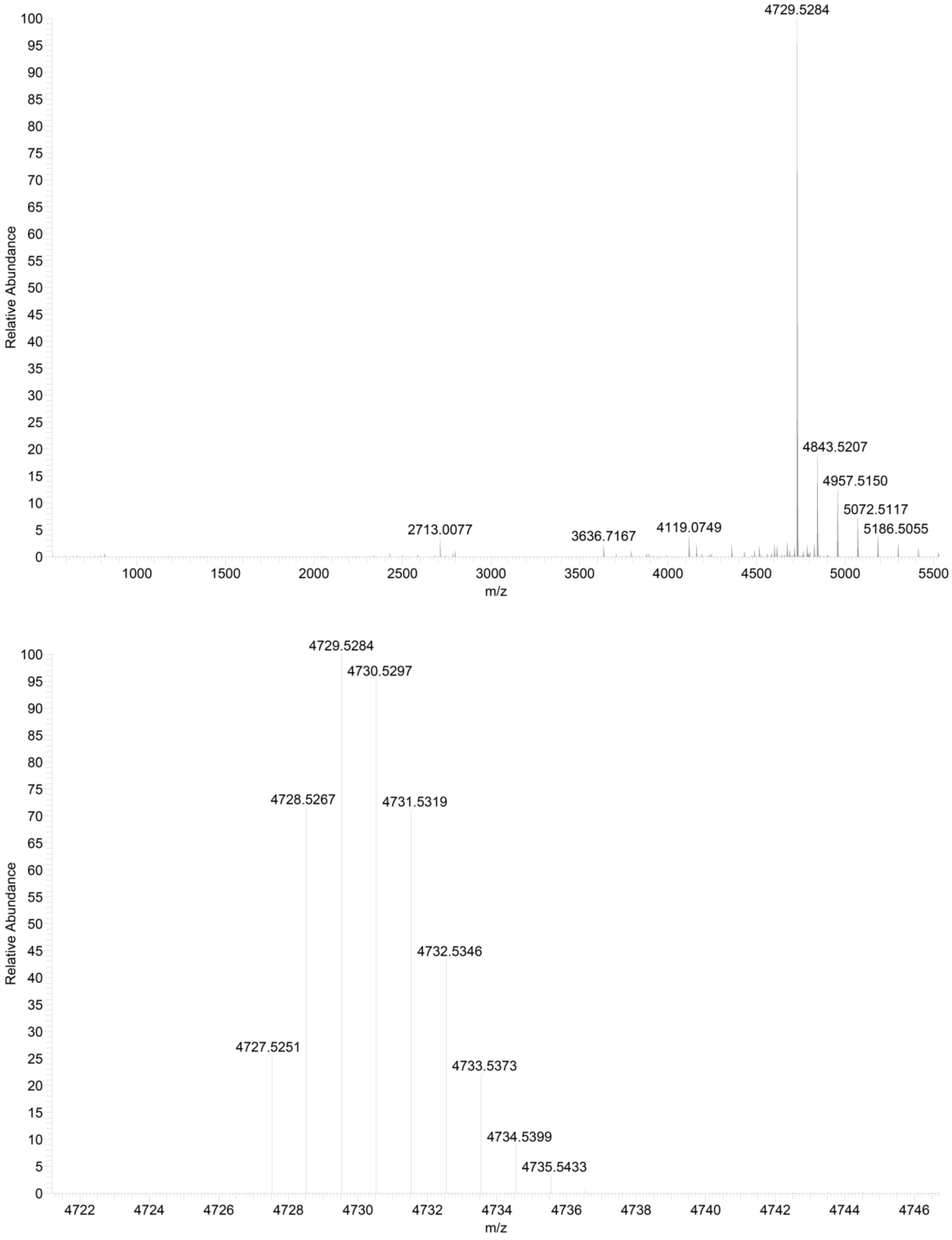

Figure S78. HRMS spectrum.

sr-**X22** ((*K*L)$_8$(*K*L)$_4$(*K*KLL)$_2$*K*LKK) was manually synthesized using TentaGel S RAM resin (393.4 mg, 0.09 mmol, 0.22 mmol·g^{-1}), the dendrimer was obtained as a white foamy solid after preparative RP-HPLC purification (97.8 mg, 18.4%). Analytical RP-HPLC: t_R = 1.41 min (100% A to 100% B in 3.5 min, λ= 214 nm). MS (ESI+): $C_{216}H_{418}N_{56}O_{36}$ calc./obs. 4373.26/4373.28 $[M]^+$.

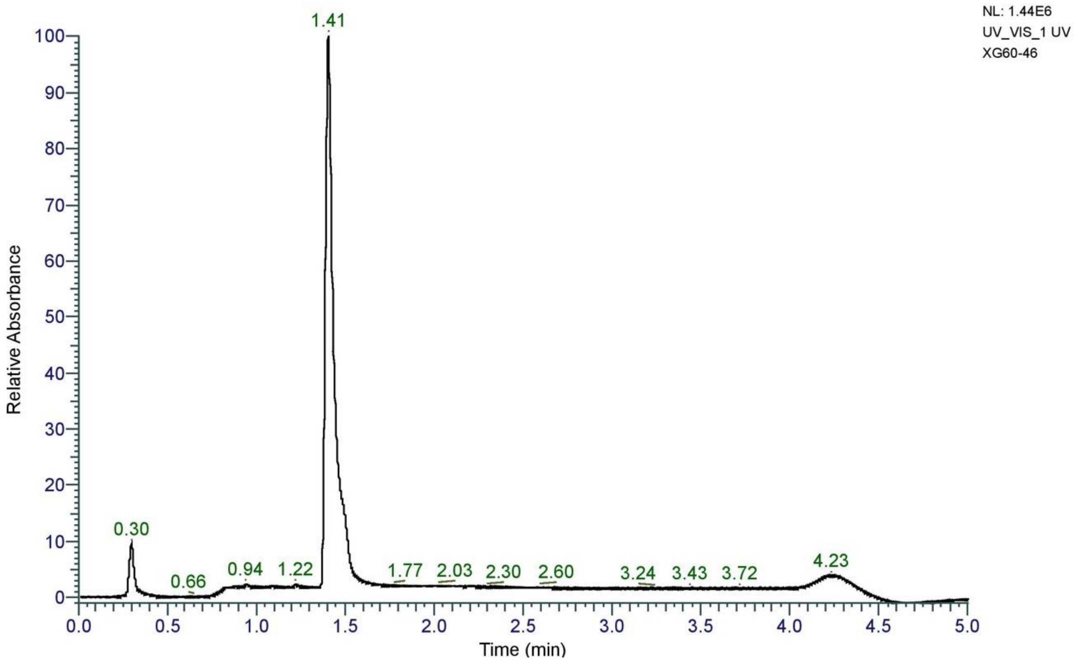

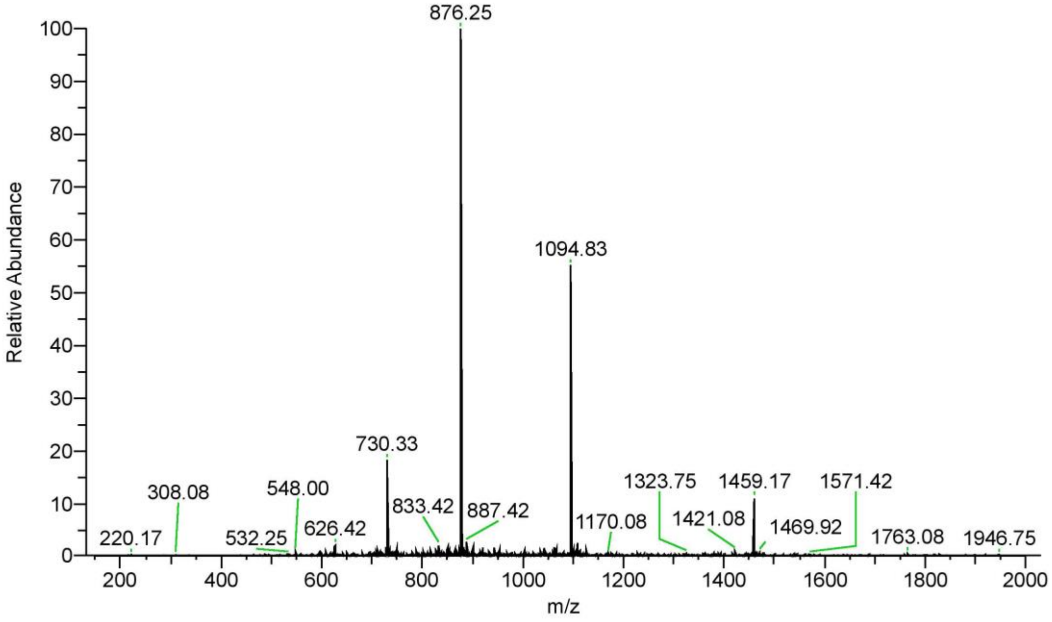

Figure S79. LCMS spectrum.

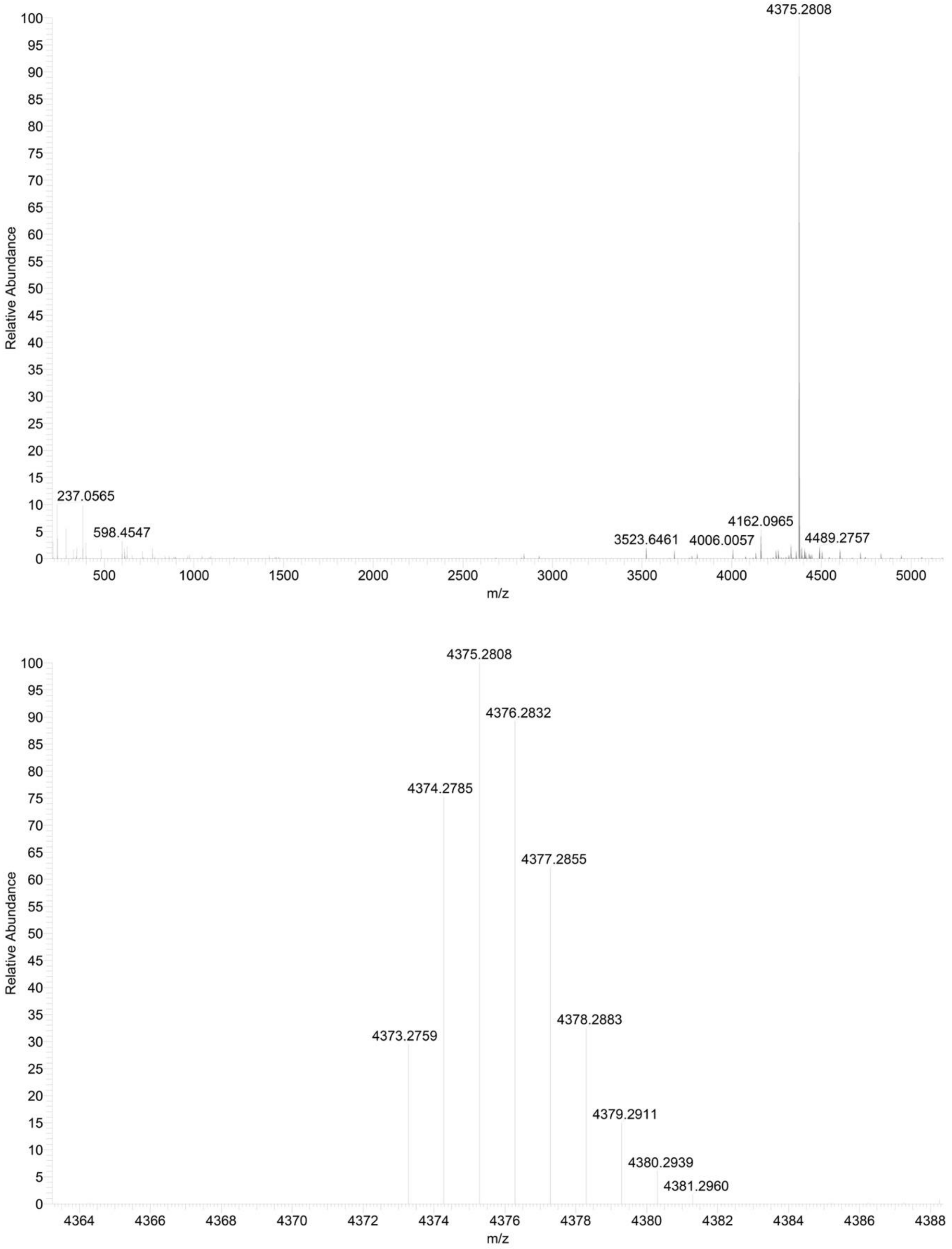

Figure S80. HRMS spectrum.

sr-**X23** ((*K*L)$_8$(*K*LK)$_4$(*K*LL)$_2$*K*LLK) was manually synthesized using TentaGel S RAM resin (393.4 mg, 0.09 mmol, 0.22 mmol·g^{-1}), the dendrimer was obtained as a white foamy solid after preparative RP-HPLC purification (80.1 mg, 14.3%). Analytical RP-HPLC: t_R = 1.40 min (100% A to 100% B in 3.5 min, λ= 214 nm). MS (ESI+): $C_{228}H_{441}N_{59}O_{38}$ calc./obs. 4614.44/4614.43 [M]$^+$.

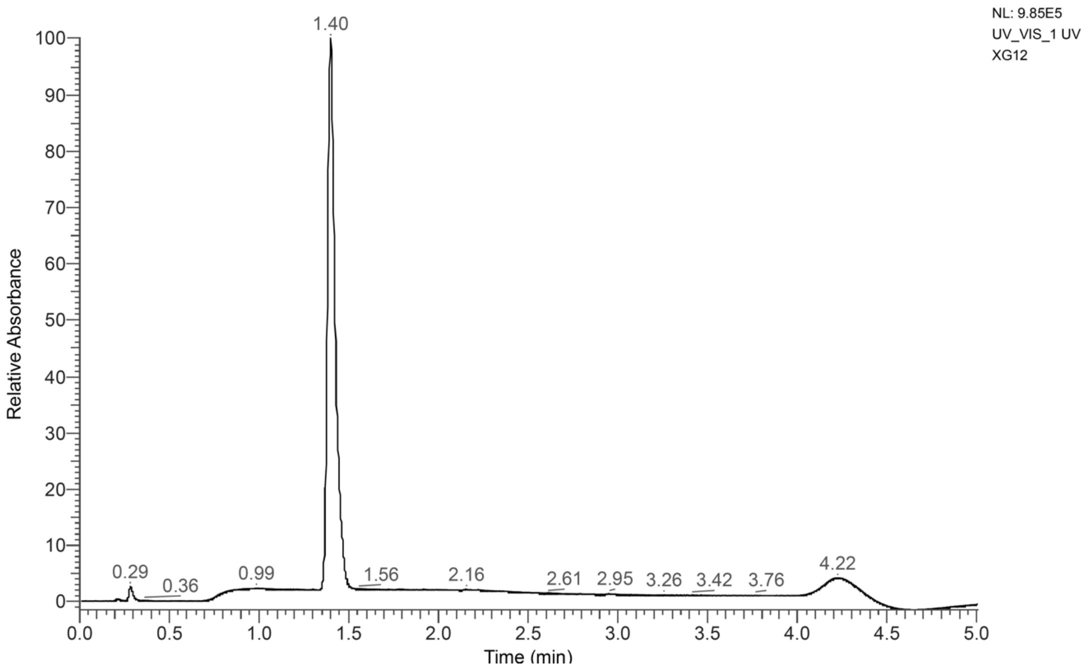

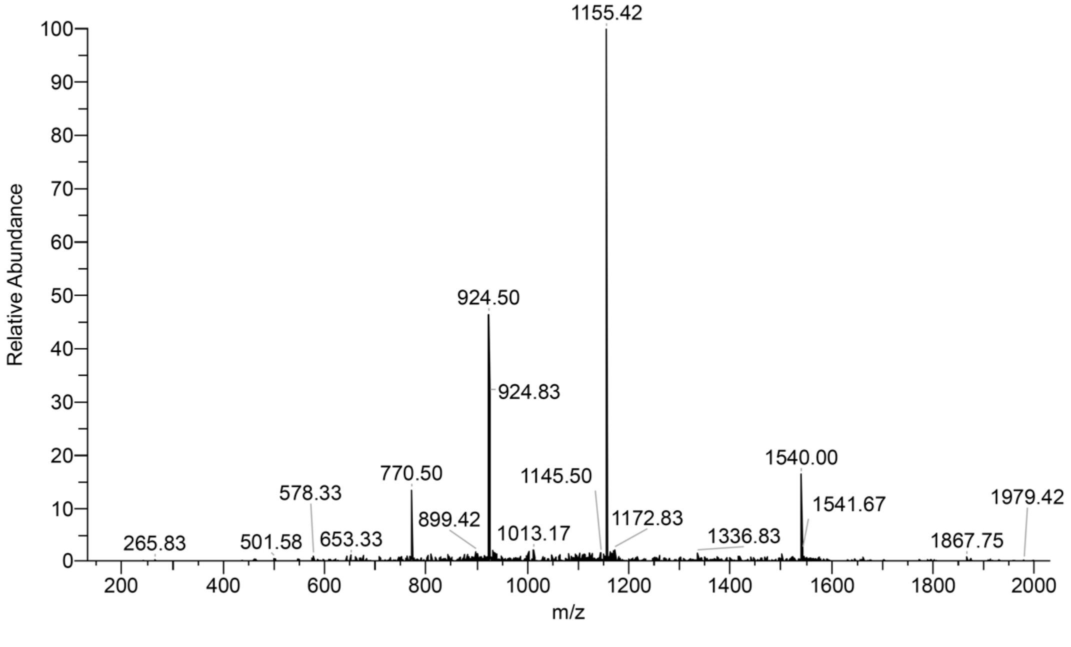

Figure S81. LCMS spectrum.

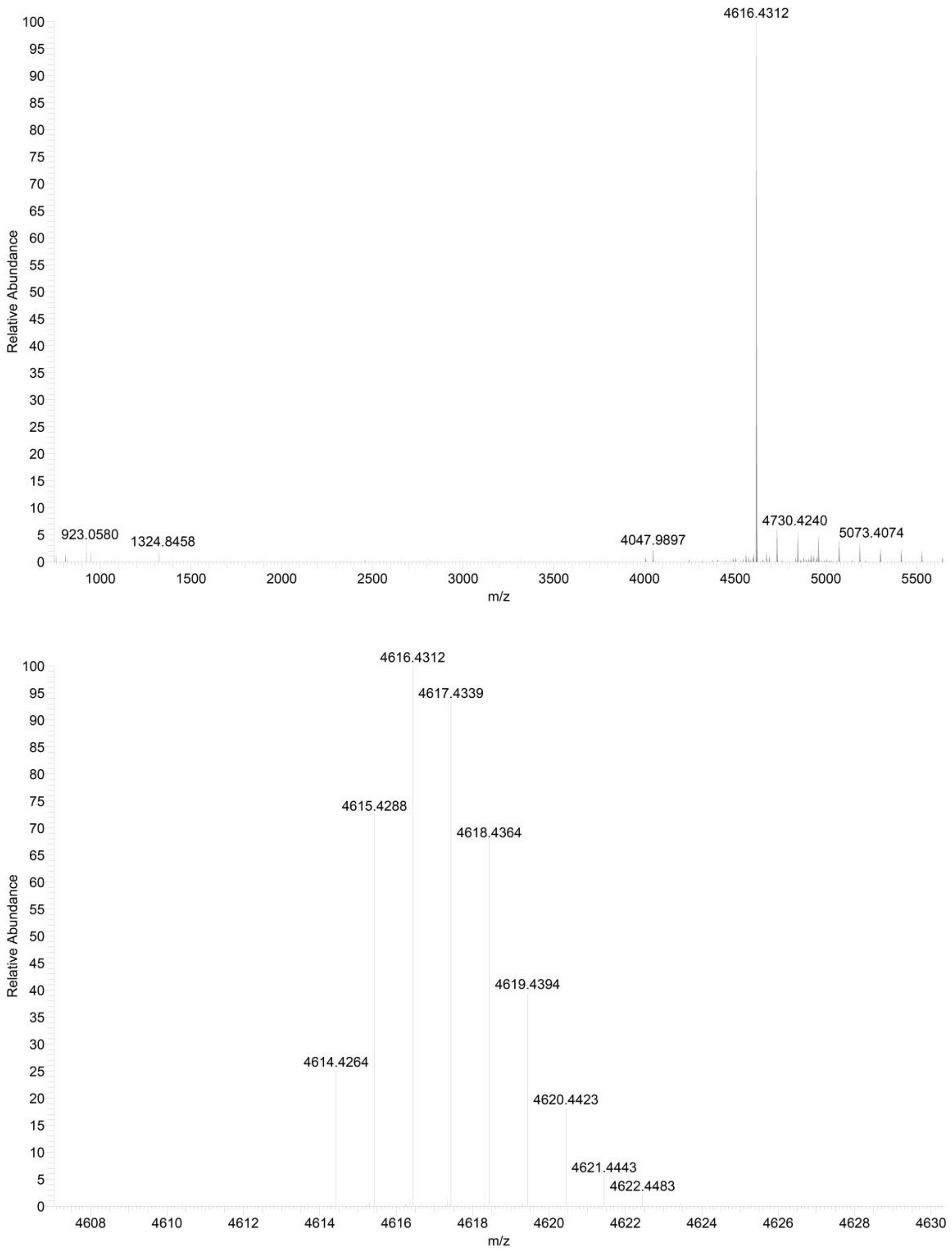

Figure S82. HRMS spectrum.

sr-**X24** ((*K*L)$_8$(*K*KL)$_4$(*K*LL)$_2$*K*LLK) was manually synthesized using TentaGel S RAM resin (393.4 mg, 0.09 mmol, 0.22 mmol·g^{-1}), the dendrimer was obtained as a white foamy solid after preparative RP-HPLC purification (116.9 mg, 19.3%). Analytical RP-HPLC: t_R = 1.38 min (100% A to 100% B in 3.5 min, λ= 214 nm). MS (ESI+): $C_{228}H_{441}N_{59}O_{38}$ calc./obs. 4614.44/4614.44 $[M]^+$.

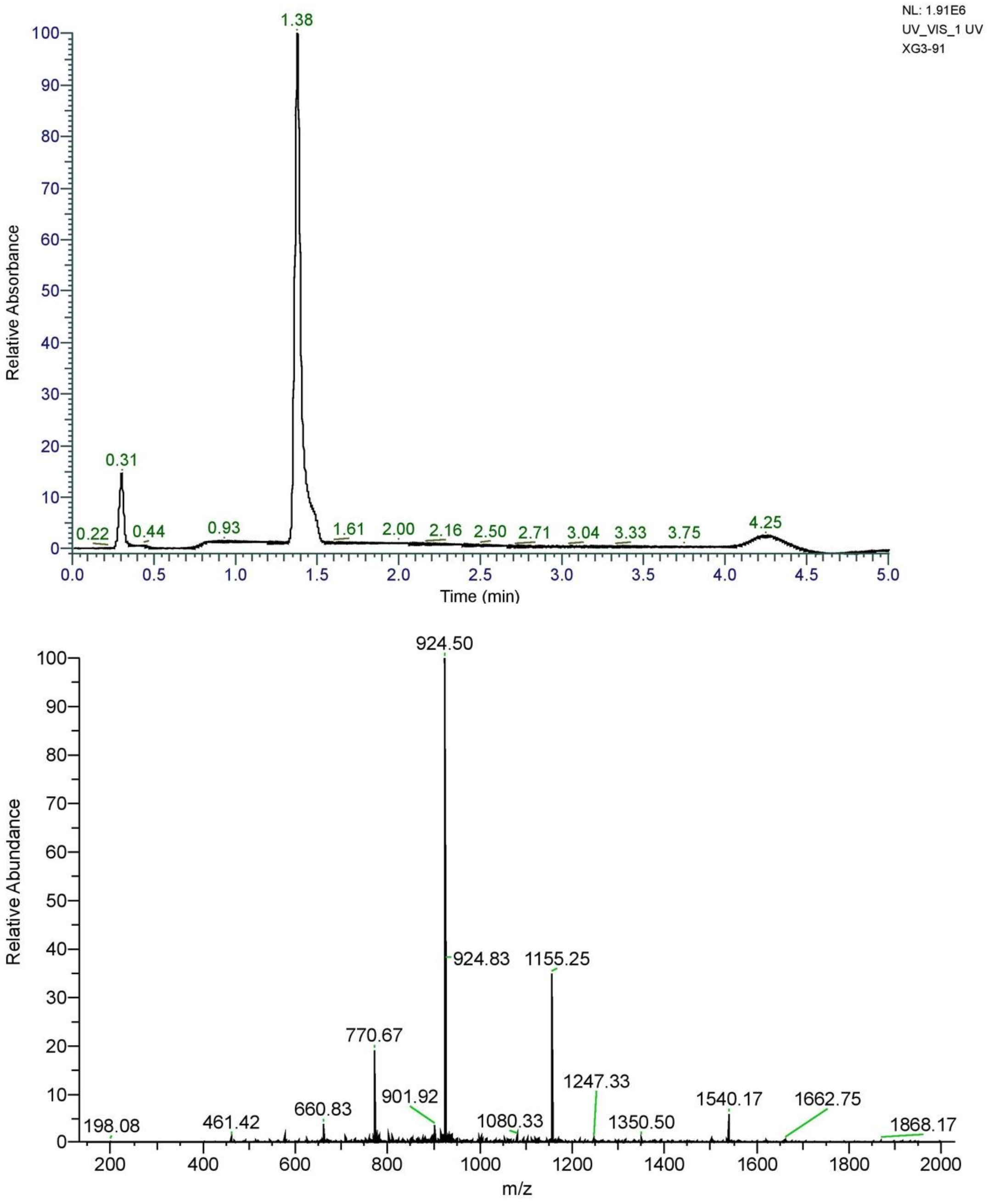

Figure S83. LCMS spectrum.

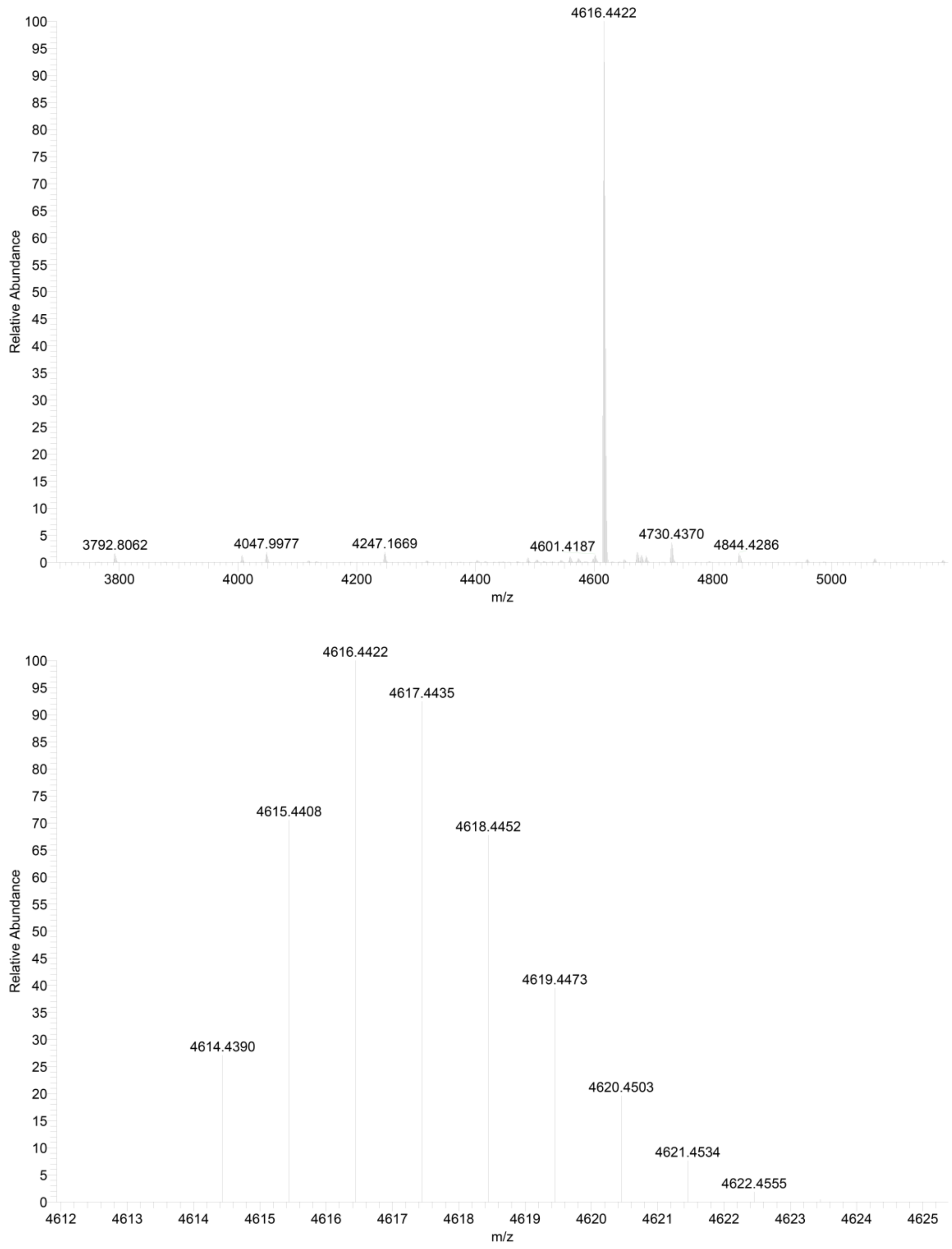

Figure S84. HRMS spectrum.

sr-**X25** ((*K*L)$_8$(*K*LK)$_4$(*K*LL)$_2$*K*LKL) was manually synthesized using TentaGel S RAM resin (393.4 mg, 0.09 mmol, 0.22 mmol·g^{-1}), the dendrimer was obtained as a white foamy solid after preparative RP-HPLC purification (133.3 mg, 23.8%). Analytical RP-HPLC: t_R = 1.38 min (100% A to 100% B in 3.5 min, λ= 214 nm). MS (ESI+): $C_{228}H_{441}N_{59}O_{38}$ calc./obs. 4614.44/4614.44 [M]$^+$.

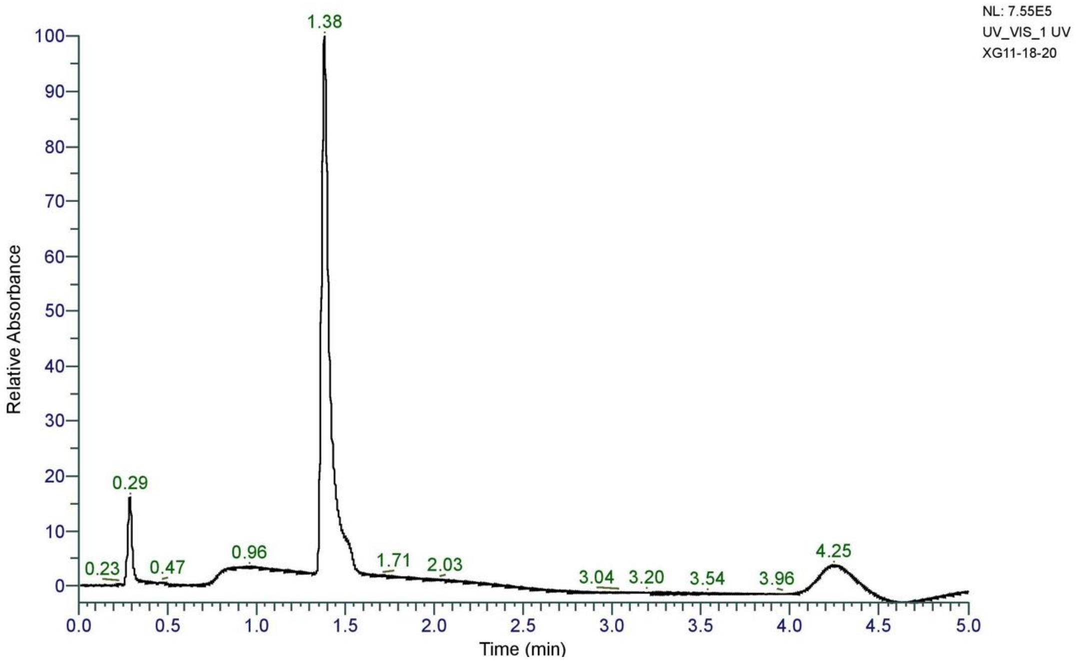

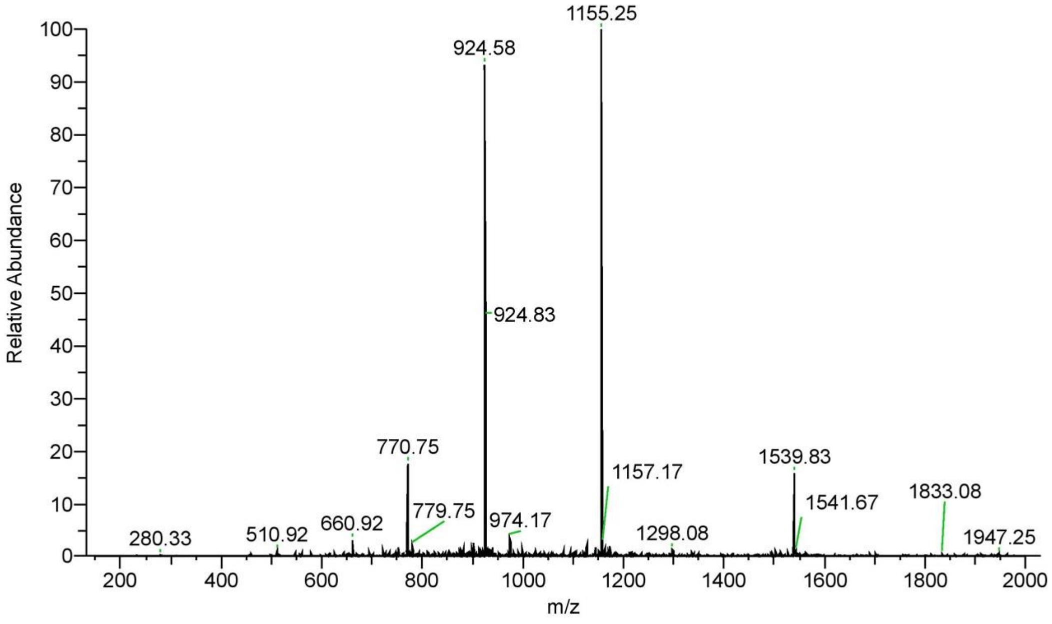

Figure S85. LCMS spectrum.

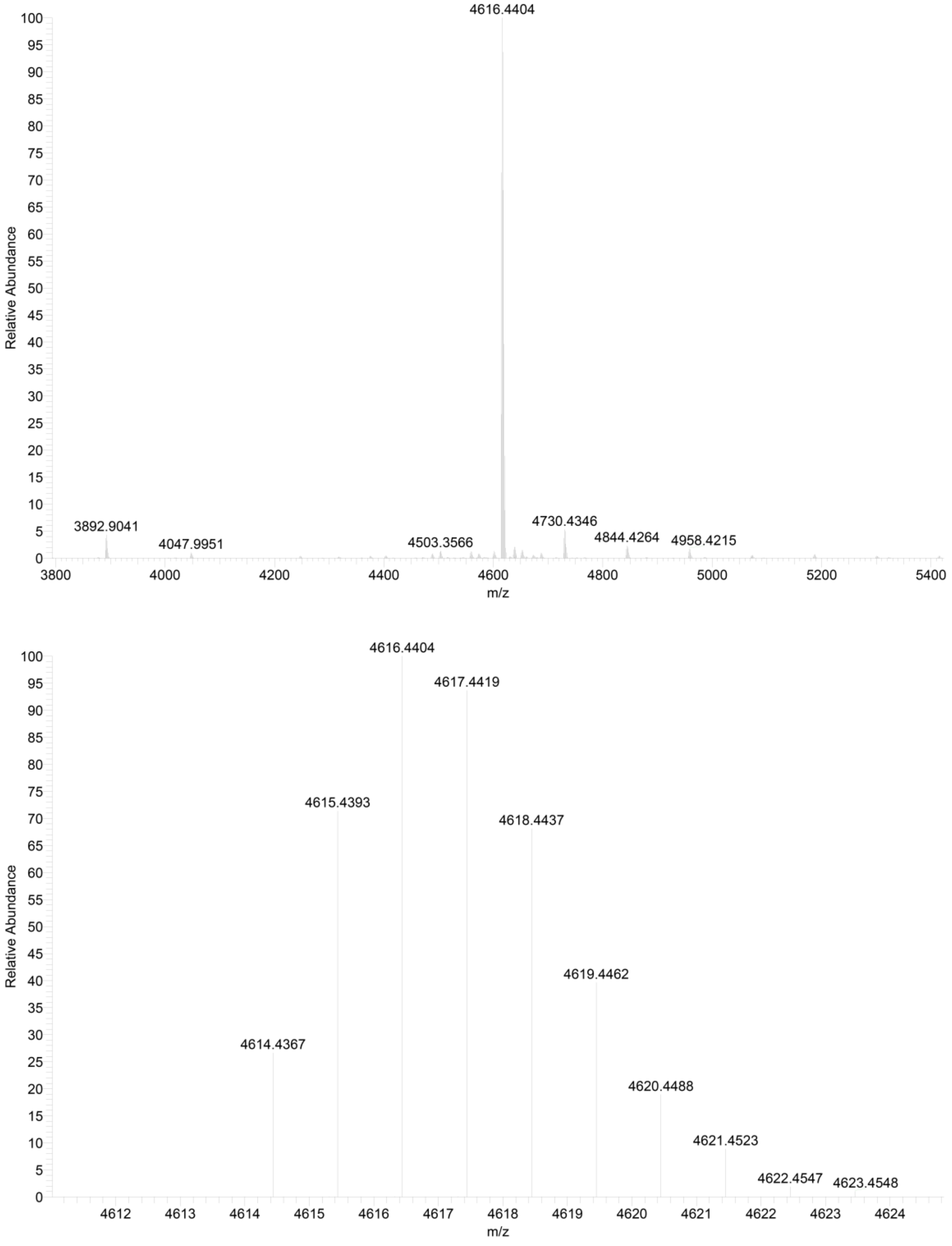

Figure S86. HRMS spectrum.

sr-**X26** ((*K*L)$_8$(*K*KL)$_4$(*K*LL)$_2$*K*LKL) was manually synthesized using TentaGel S RAM resin (332.0 mg, 0.07 mmol, 0.22 mmol·g^{-1}), the dendrimer was obtained as a white foamy solid after preparative RP-HPLC purification (79.4 mg, 15.5%). Analytical RP-HPLC: t_R = 1.40 min (100% A to 100% B in 3.5 min, λ= 214 nm). MS (ESI+): $C_{228}H_{441}N_{59}O_{38}$ calc./obs. 4614.44/4614.44 $[M]^+$.

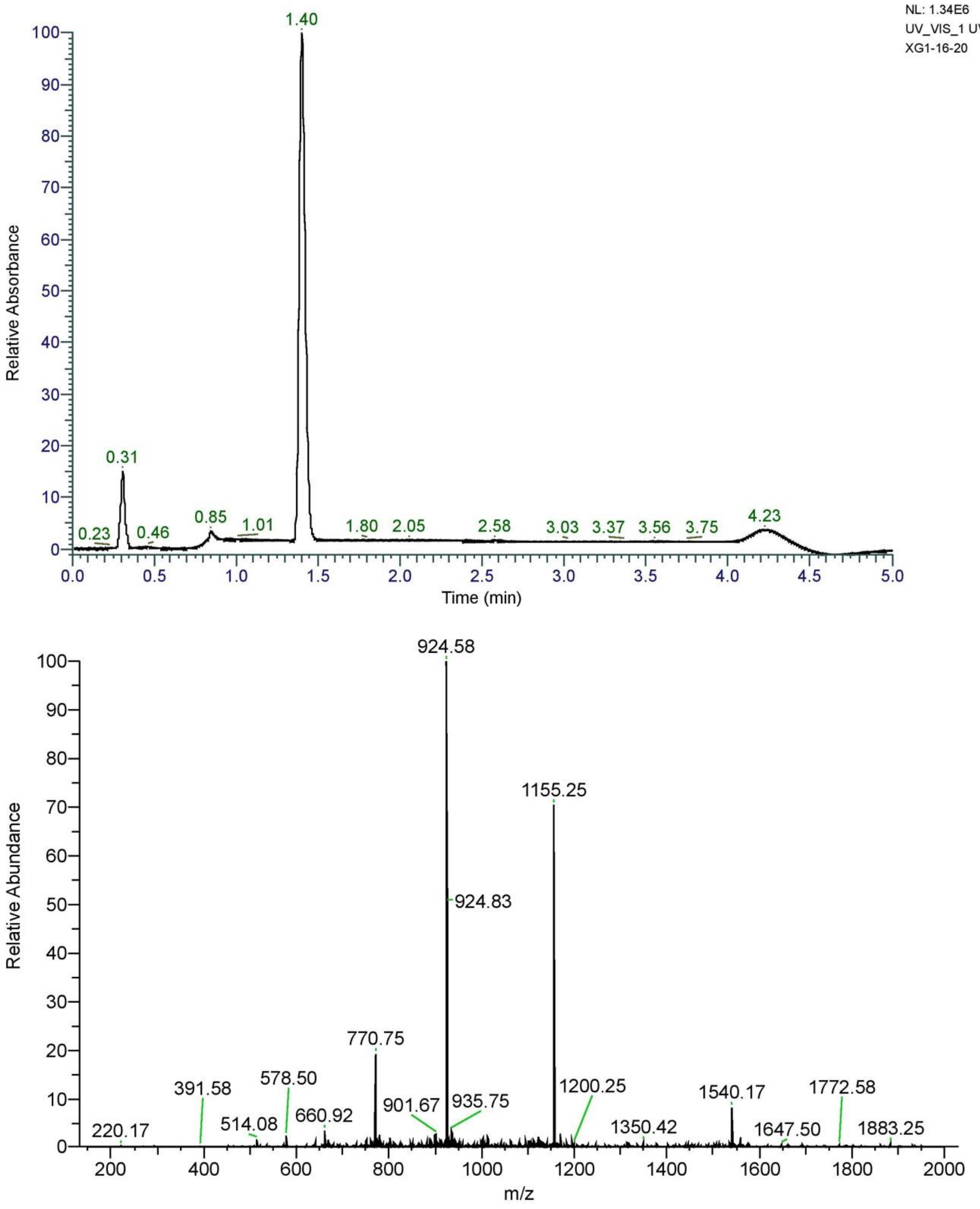

Figure S87. LCMS spectrum.

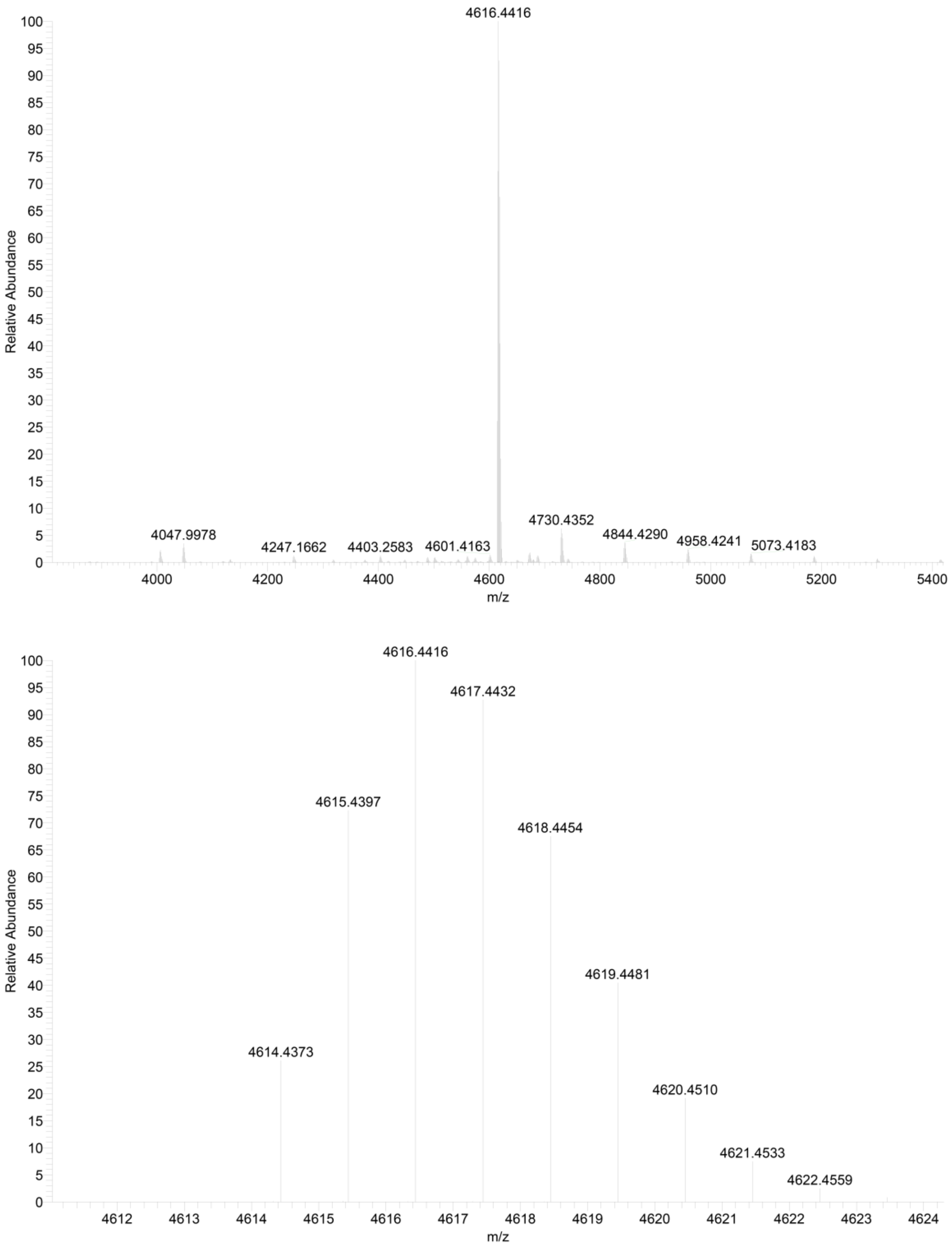

Figure S88. HRMS spectrum.

sr-**X27** ((*K*L)$_8$(*K*LK)$_4$(*K*LL)$_2$*K*KLL) was manually synthesized using TentaGel S RAM resin (393.4 mg, 0.09 mmol, 0.22 mmol·g^{-1}), the dendrimer was obtained as a white foamy solid after preparative RP-HPLC purification (151.1 mg, 27.0%). Analytical RP-HPLC: t_R = 1.40 min (100% A to 100% B in 3.5 min, λ= 214 nm). MS (ESI+): $C_{228}H_{441}N_{59}O_{38}$ calc./obs. 4614.44/4614.44 $[M]^+$.

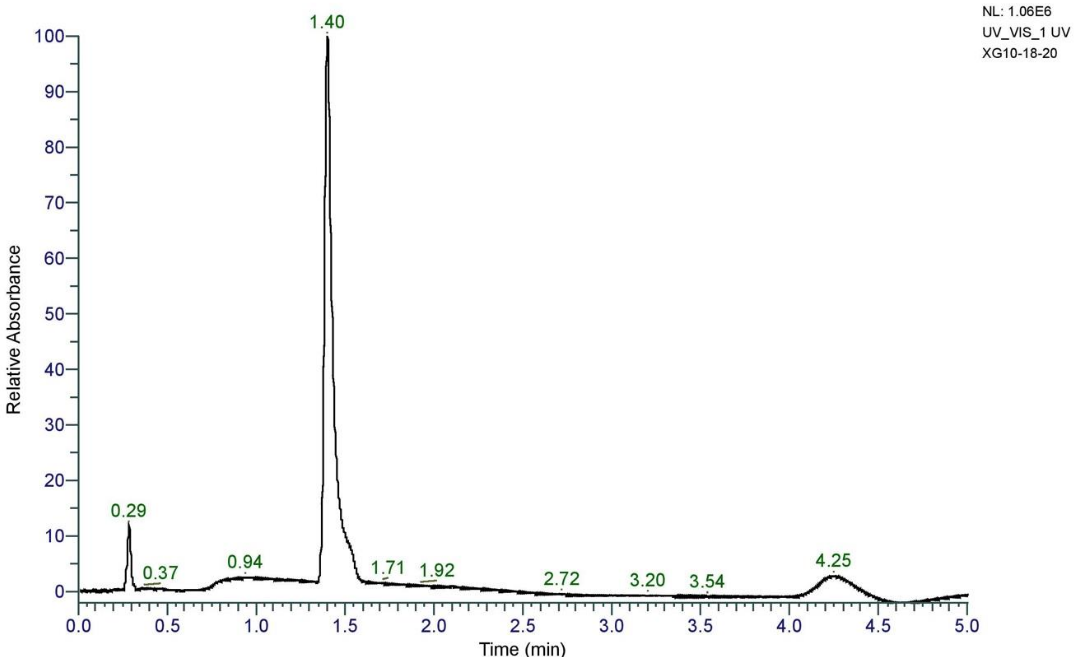

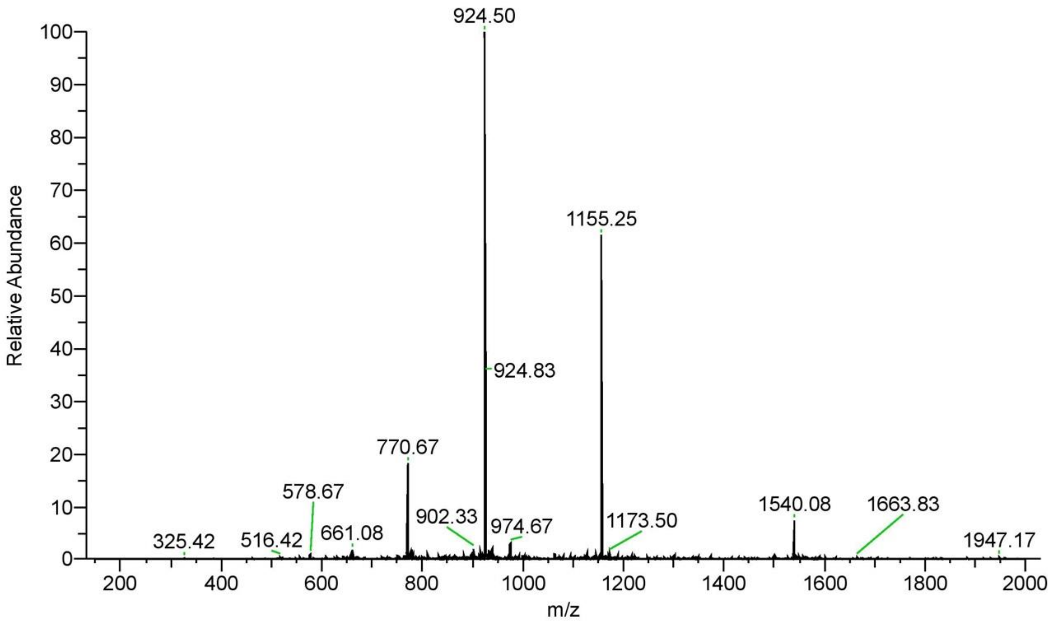

Figure S89. LCMS spectrum.

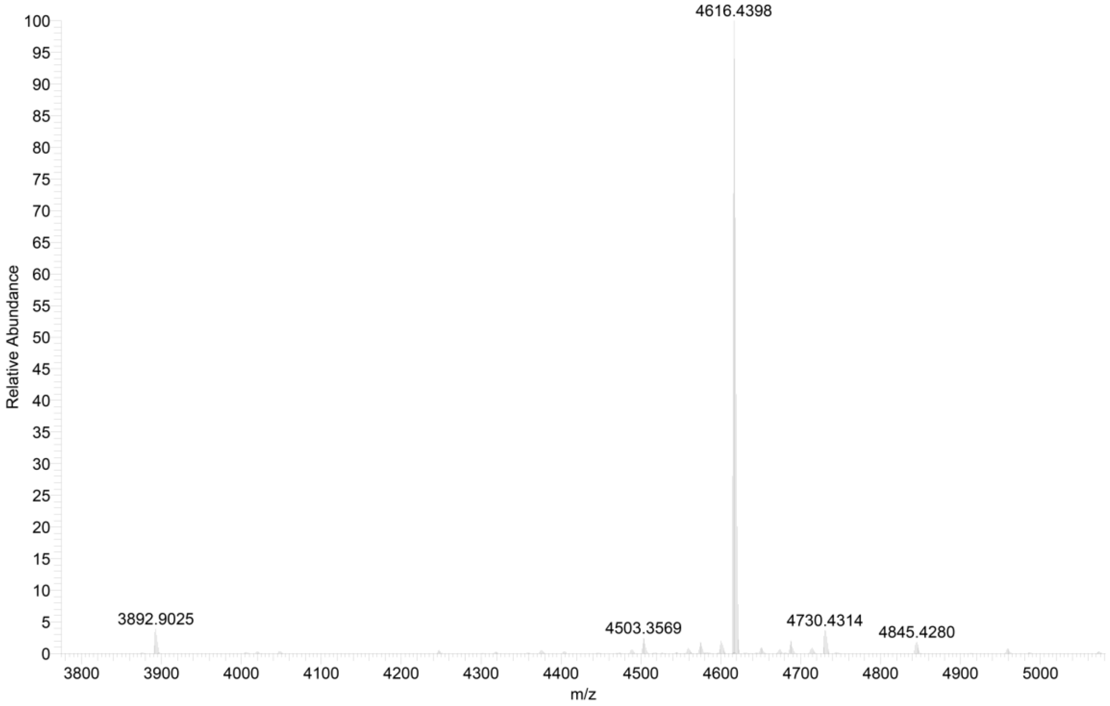

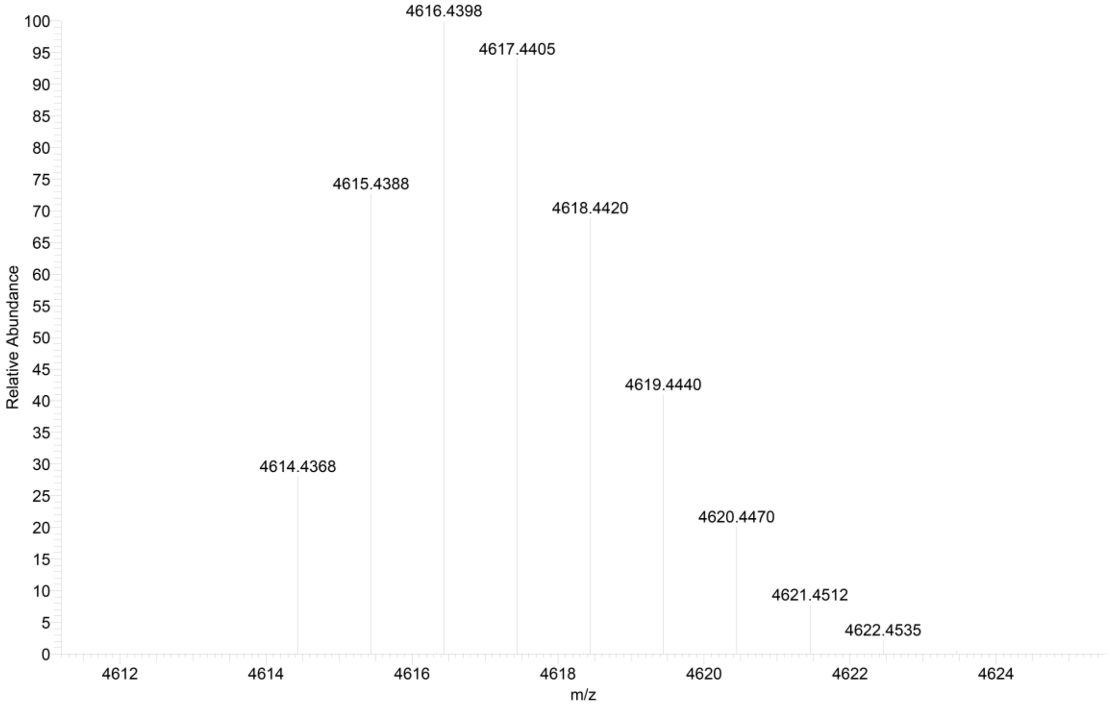

Figure S90. HRMS spectrum.

sr-**X28** ((LK)$_8$(*K*KL)$_4$(*K*LL)$_2$*K*LLK) was manually synthesized using TentaGel S RAM resin (393.4 mg, 0.09 mmol, 0.22 mmol·g^{-1}), the dendrimer was obtained as a white foamy solid after preparative RP-HPLC purification (87.5 mg, 15.6%). Analytical RP-HPLC: t_R = 1.35 min (100% A to 100% B in 3.5 min, λ= 214 nm). MS (ESI+): $C_{228}H_{441}N_{59}O_{38}$ calc./obs. 4614.44/4614.46 [M]$^+$.

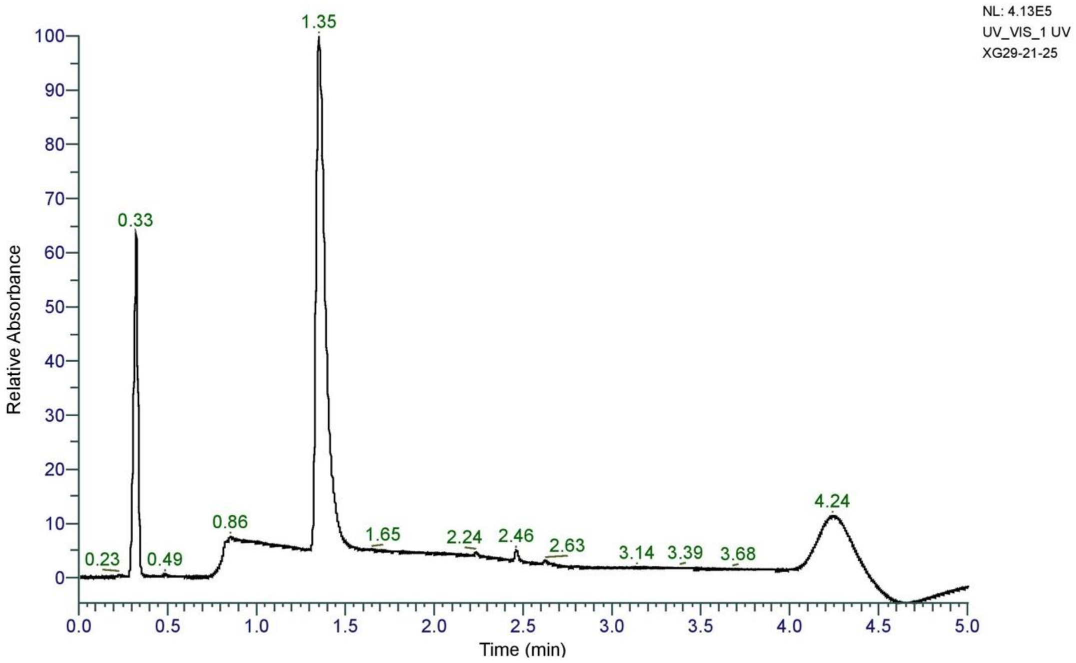

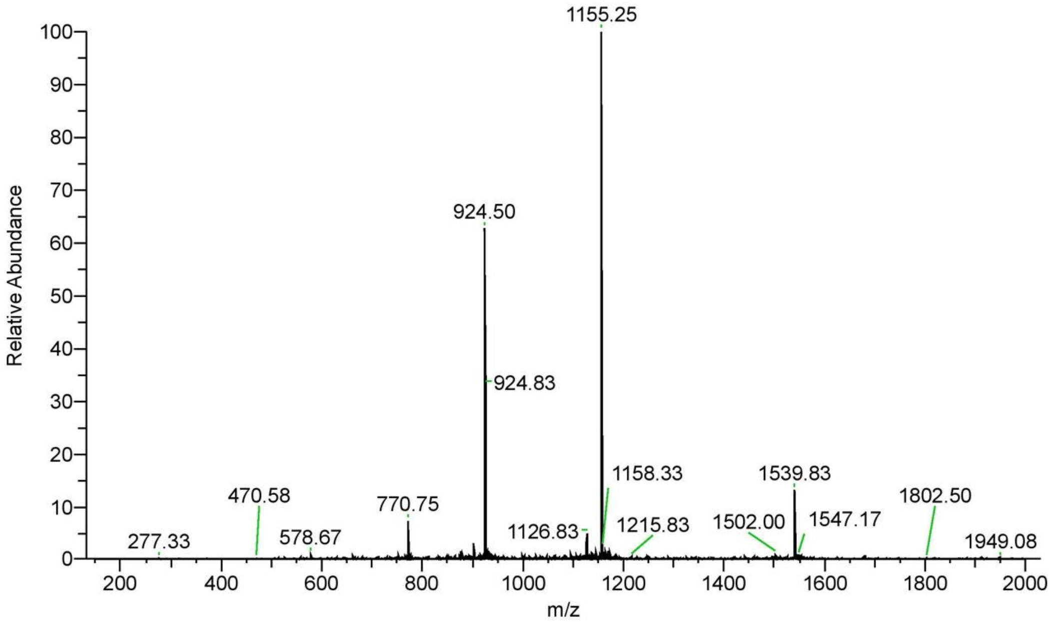

Figure S91. LCMS spectrum.

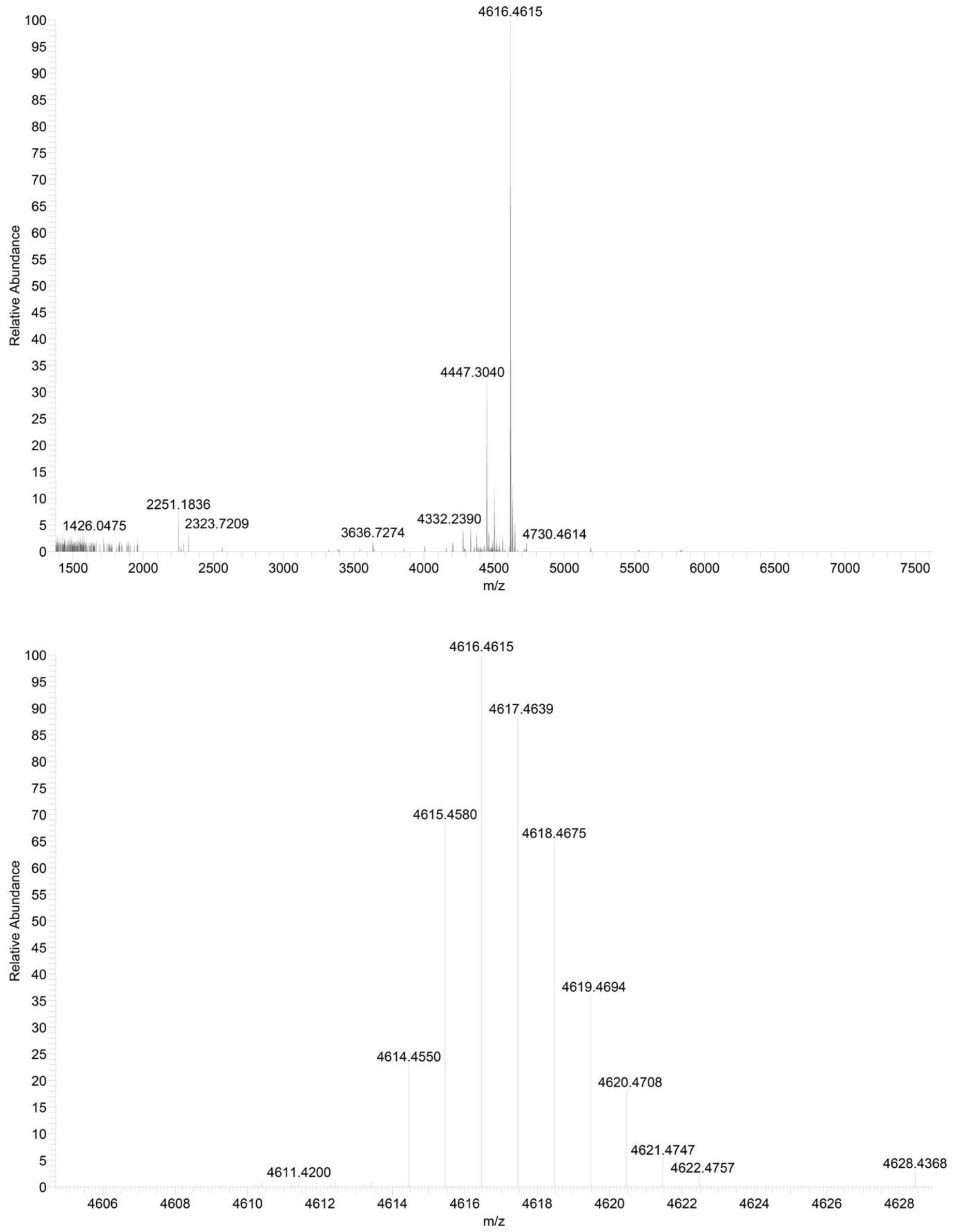

Figure S92. HRMS spectrum.

sr-**X29** ((*K*L)$_8$(*K*KL)$_4$(*K*LL)$_2$*K*KL) was manually synthesized using TentaGel S RAM resin (393.4 mg, 0.09 mmol, 0.22 mmol·g^{-1}), the dendrimer was obtained as a white foamy solid after preparative RP-HPLC purification (77.6 mg, 14.1%). Analytical RP-HPLC: t_R = 1.38 min (100% A to 100% B in 3.5 min, λ= 214 nm). MS (ESI+): $C_{222}H_{430}N_{58}O_{37}$ calc./obs. 4501.35/4501.35 $[M]^+$.

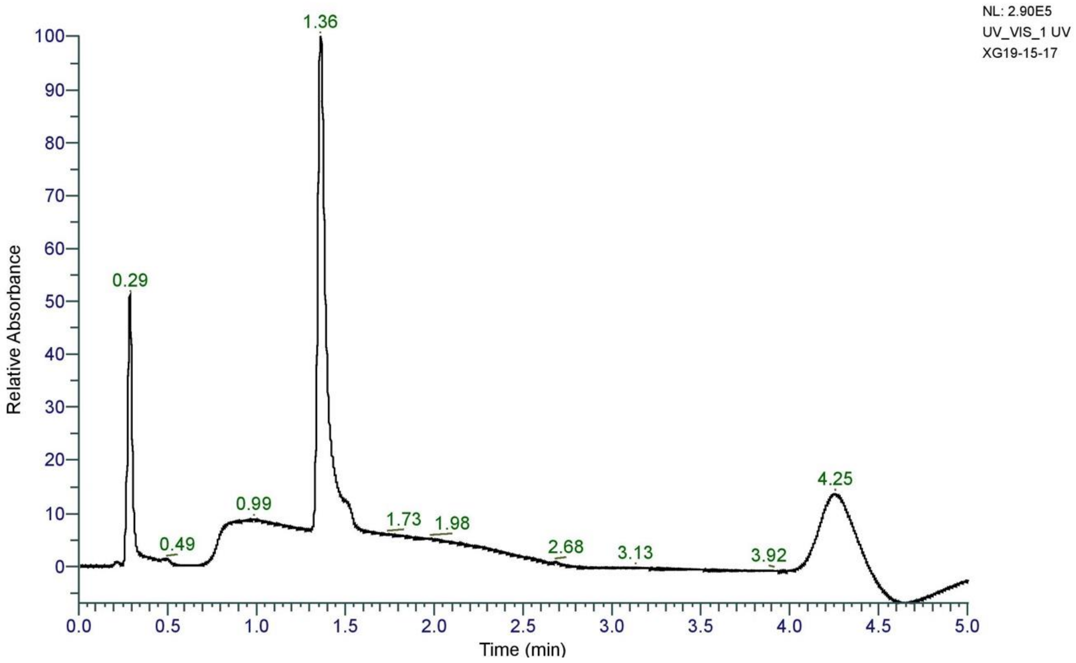

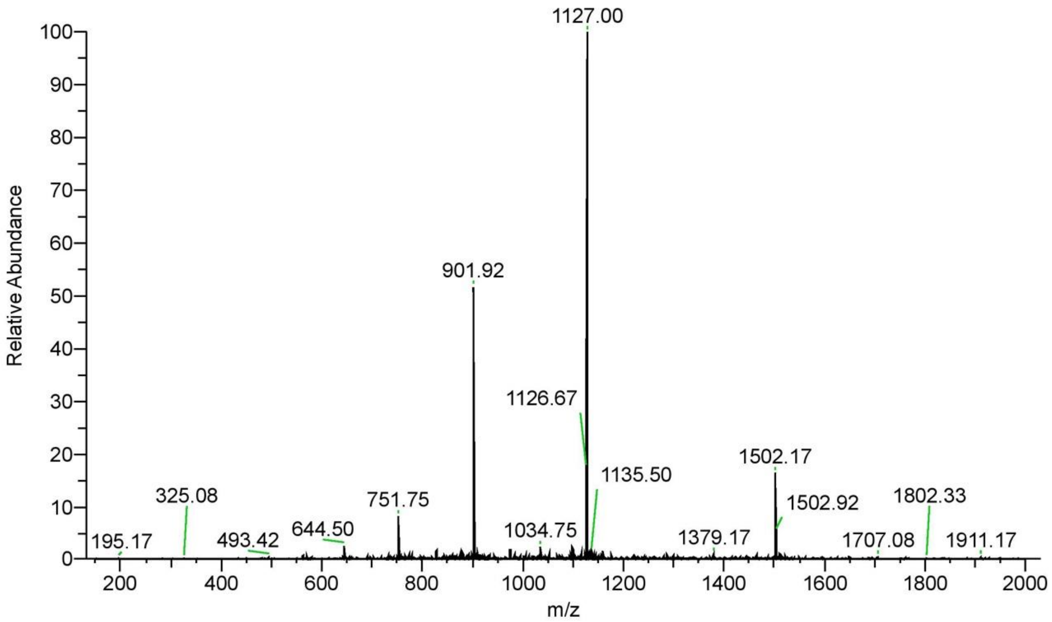

Figure S93. LCMS spectrum.

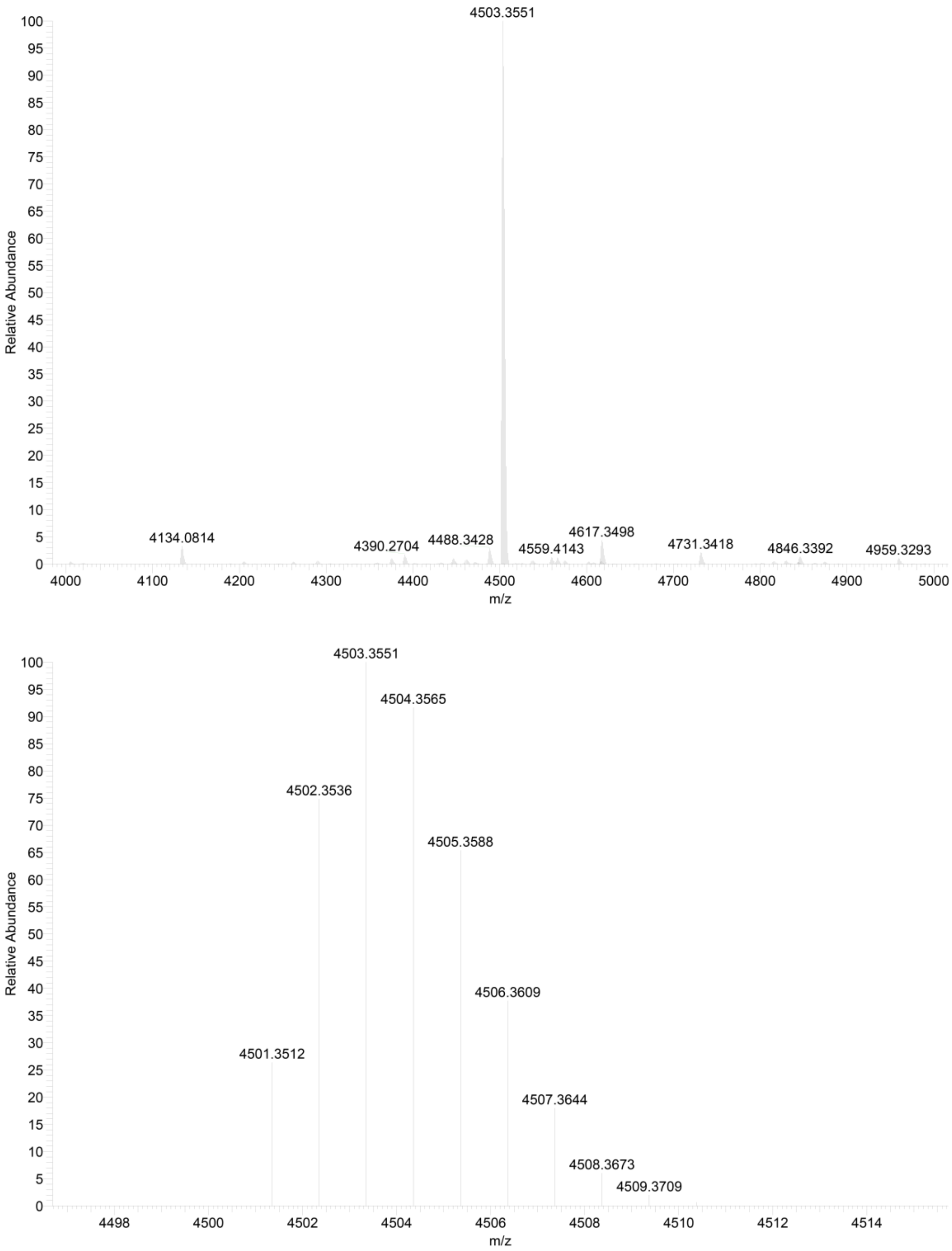

Figure S94. HRMS spectrum.

sr-**X30** ((*K*L)$_8$(*K*KLL)$_4$(*K*K)$_2$*K*LL) was manually synthesized using TentaGel S RAM resin (393.4 mg, 0.09 mmol, 0.22 mmol·g^{-1}), the dendrimer was obtained as a white foamy solid after preparative RP-HPLC purification (89.0 mg, 15.3%). Analytical RP-HPLC: t_R = 1.42 min (100% A to 100% B in 3.5 min, λ= 214 nm). MS (ESI+): $C_{234}H_{453}N_{61}O_{39}$ calc./obs. 4742.53/4742.52 [M]$^+$.

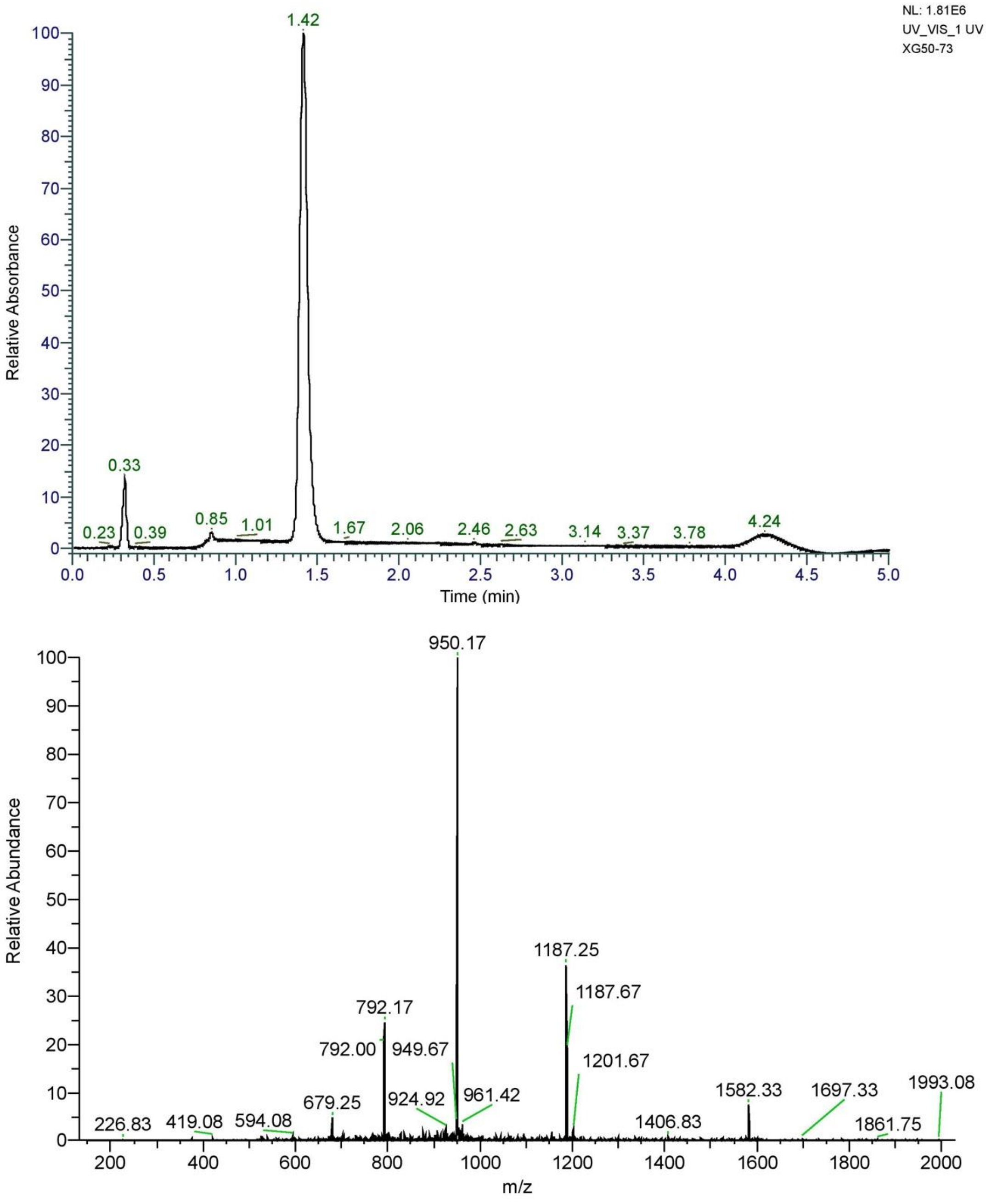

Figure S95. LCMS spectrum.

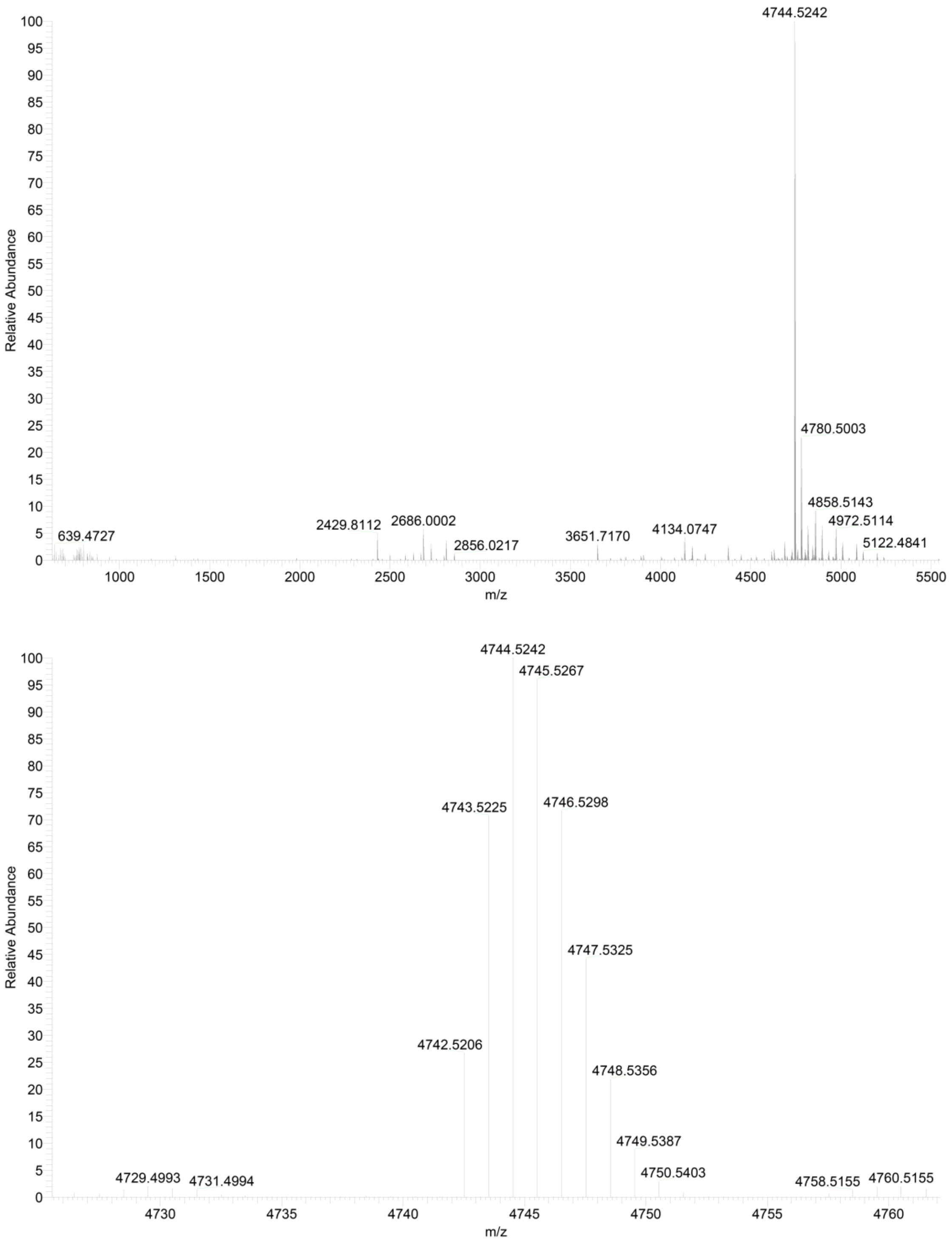

Figure S96. HRMS spectrum.

sr-**X31** ((LK)$_8$(*K*LK)$_4$(*K*LL)$_2$*K*LKL) was synthesized by CEM Liberty Blue synthesizer using Rink Amide MBHA resin (363.6 mg, 0.09 mmol, 0.25 mmol·g^{-1}), the dendrimer was obtained as a white foamy solid after preparative RP-HPLC purification (51.6 mg, 8.1%). Analytical RP-HPLC: t_R = 1.35 min (100% A to 100% B in 3.5 min, λ= 214 nm). MS (ESI+): $C_{228}H_{441}N_{59}O_{38}$ calc./obs. 4614.44/4614.44 [M]$^+$.

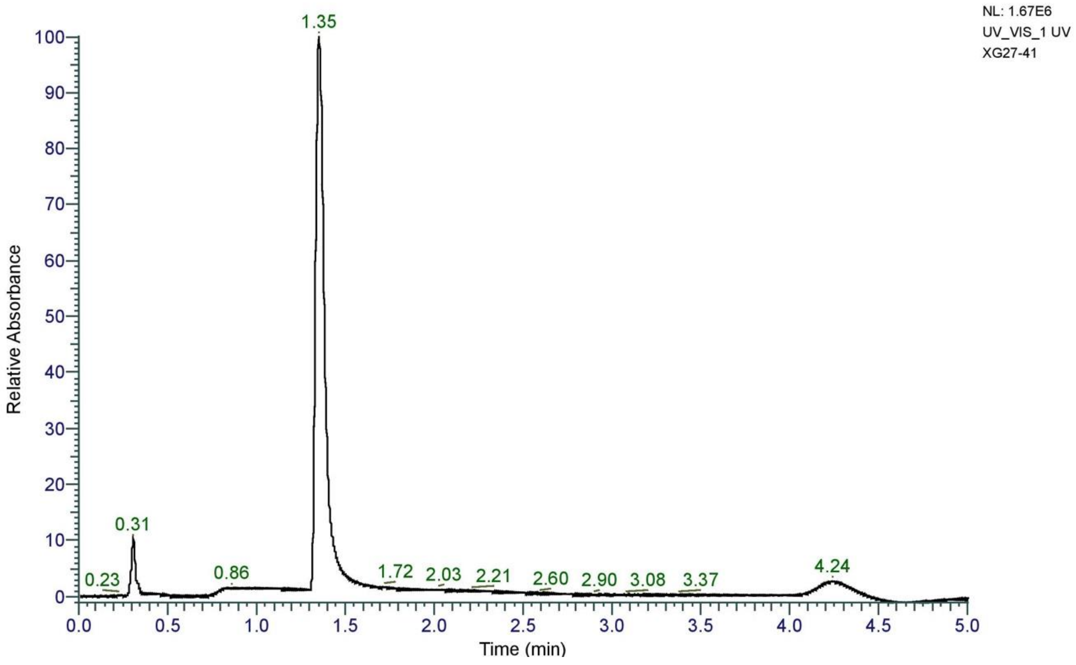

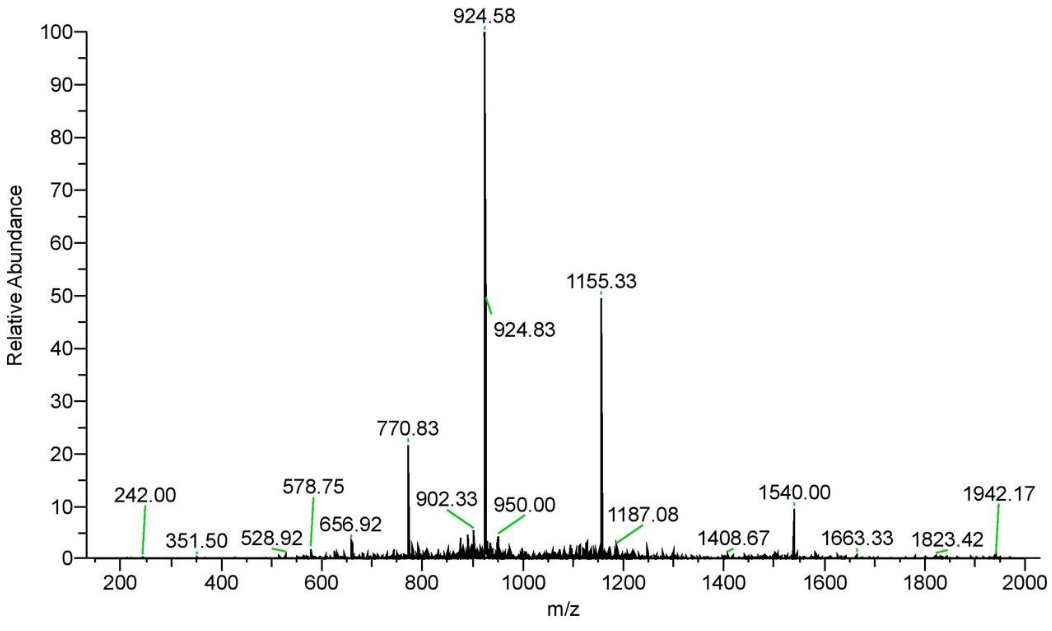

Figure S97. LCMS spectrum.

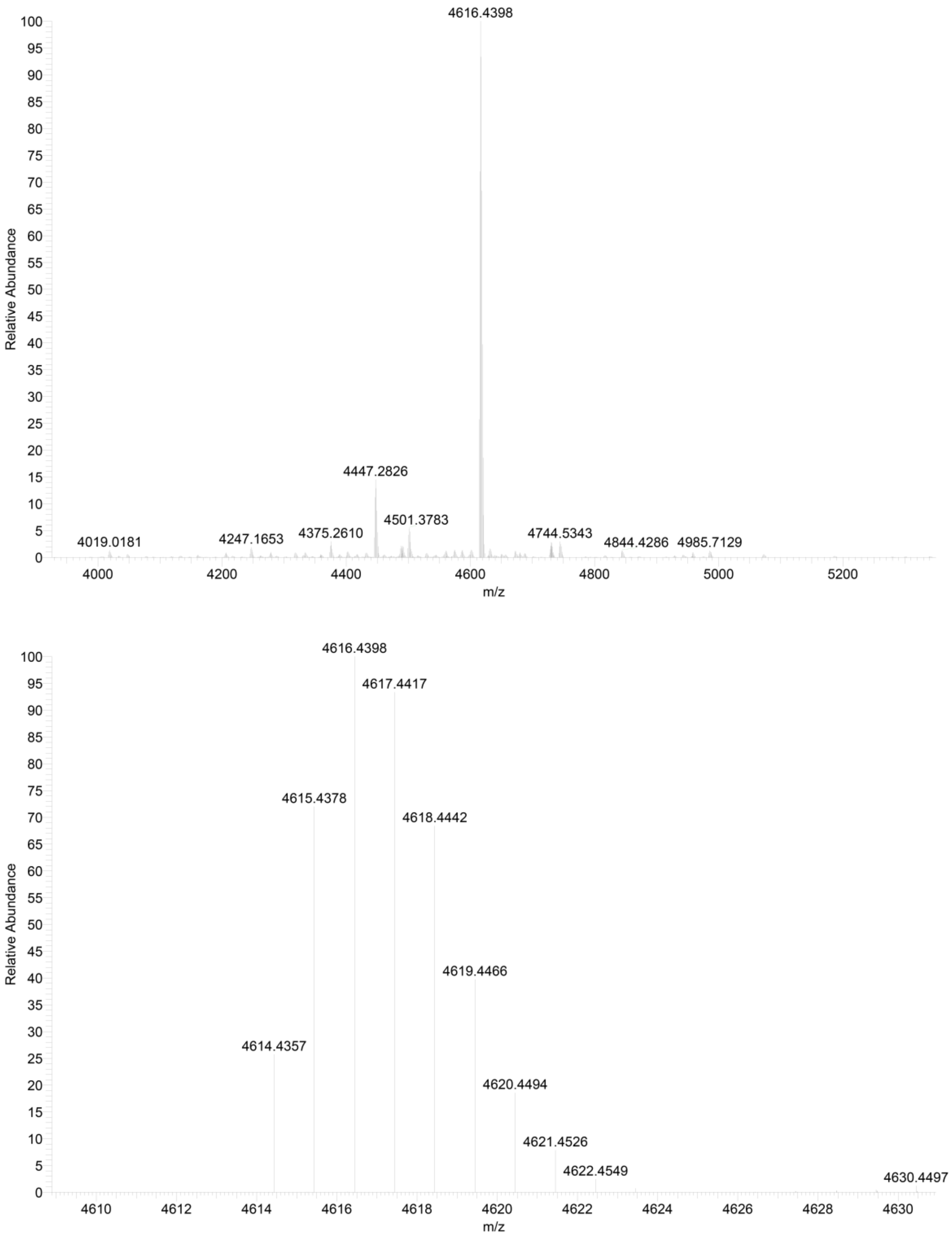

Figure S98. HRMS spectrum.

sr-**X32** ((*K*L)$_8$(*K*KL)$_4$(*K*LL)$_2$*K*K) was manually synthesized using TentaGel S RAM resin (393.4 mg, 0.09 mmol, 0.22 mmol·g^{-1}), the dendrimer was obtained as a white foamy solid after preparative RP-HPLC purification (59.9 mg, 11.0%). Analytical RP-HPLC: t_R = 1.38 min (100% A to 100% B in 3.5 min, λ= 214 nm). MS (ESI+): $C_{216}H_{419}N_{57}O_{36}$ calc./obs. 4388.27/4388.26 $[M]^+$.

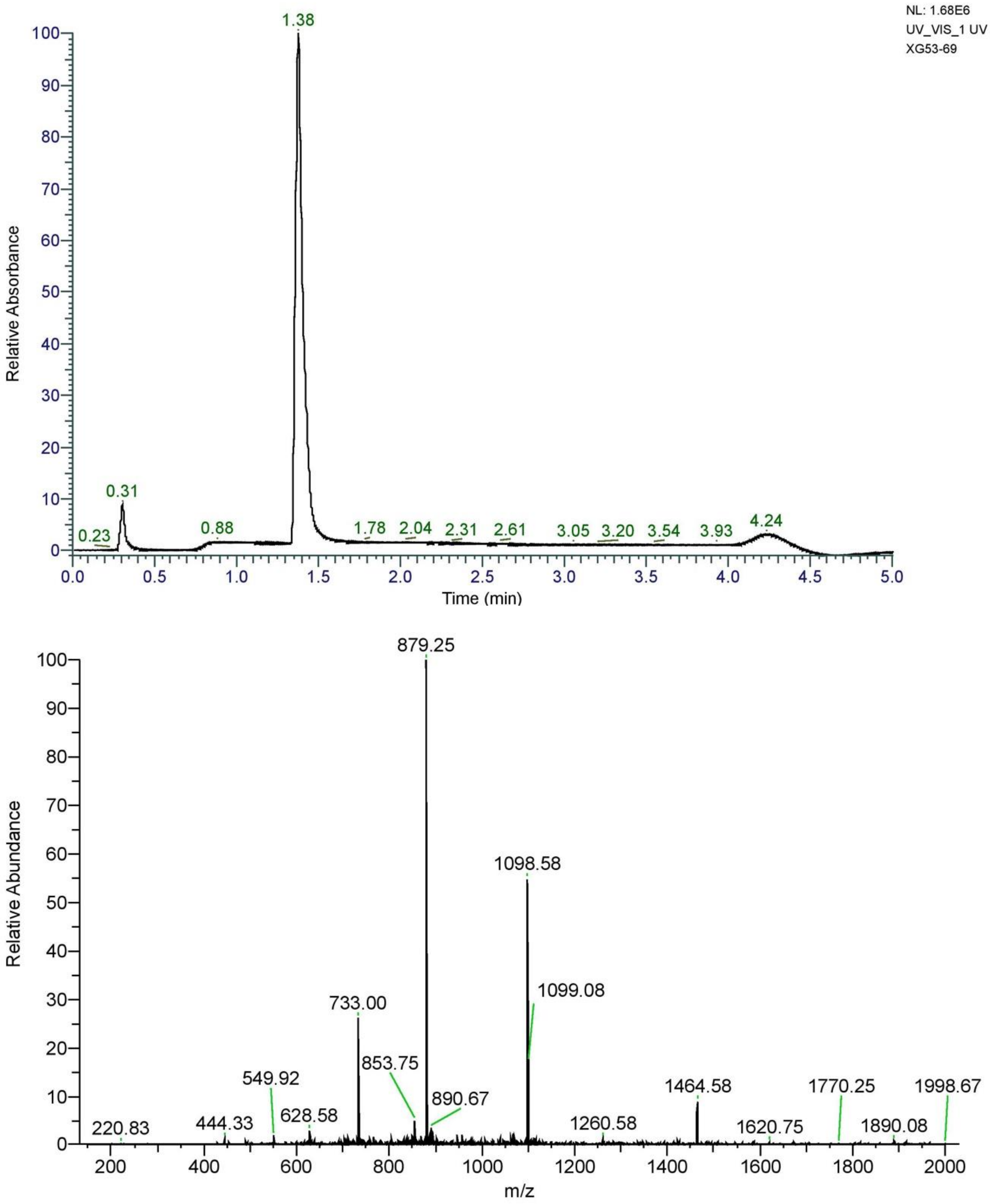

Figure S99. LCMS spectrum.

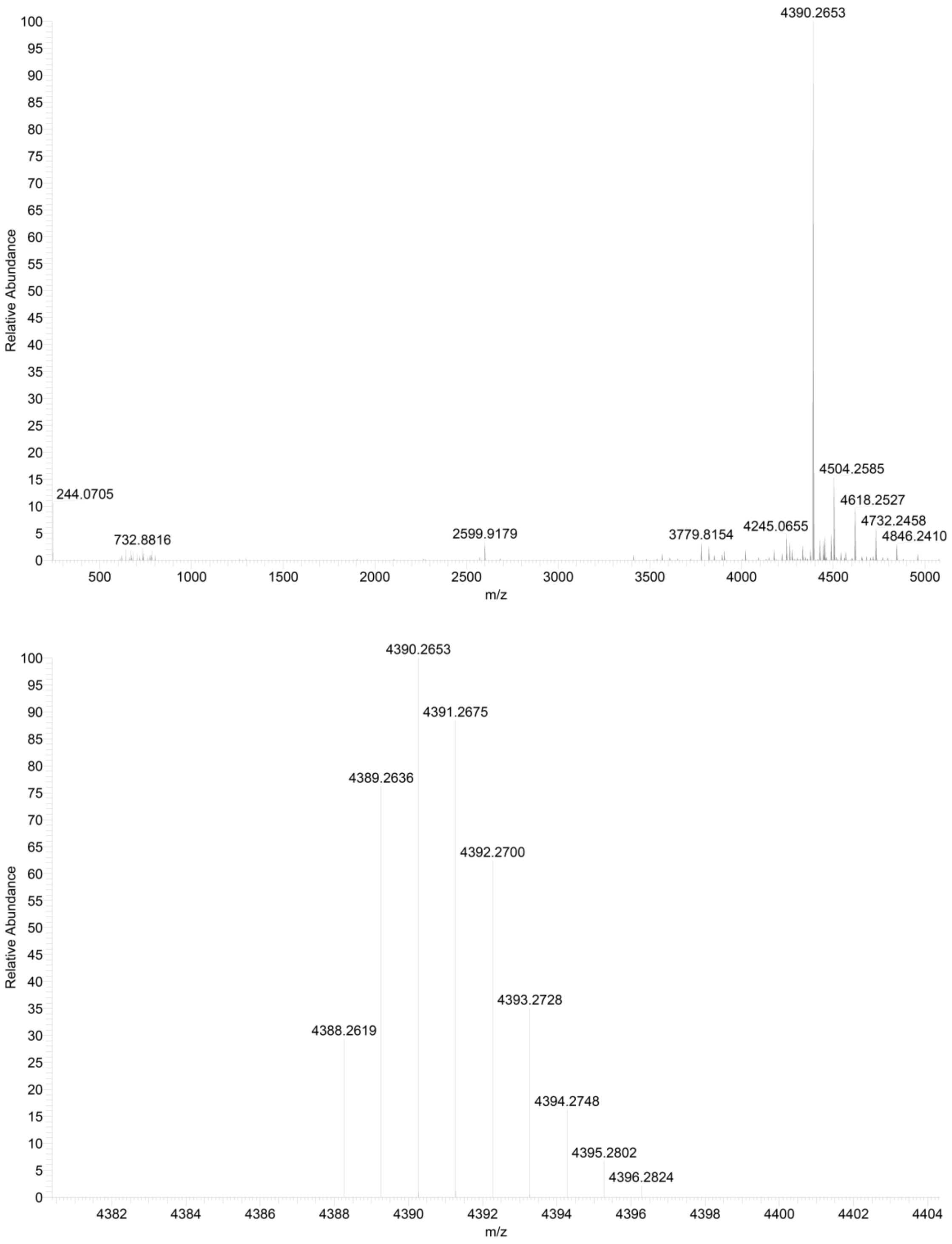

Figure S100. HRMS spectrum.

sr-**X33** ((*K*L)$_8$(*K*KL)$_4$(*K*KL)$_2$*K*LLL) was manually synthesized using TentaGel S RAM resin (387.3 mg, 0.09 mmol, 0.22 mmol·g^{-1}), the dendrimer was obtained as a white foamy solid after preparative RP-HPLC purification (103.8 mg, 17.1%). Analytical RP-HPLC: t_R = 1.37 min (100% A to 100% B in 3.5 min, λ= 214 nm). MS (ESI+): $C_{228}H_{442}N_{60}O_{38}$ calc./obs. 4629.45/4629.45 $[M]^+$.

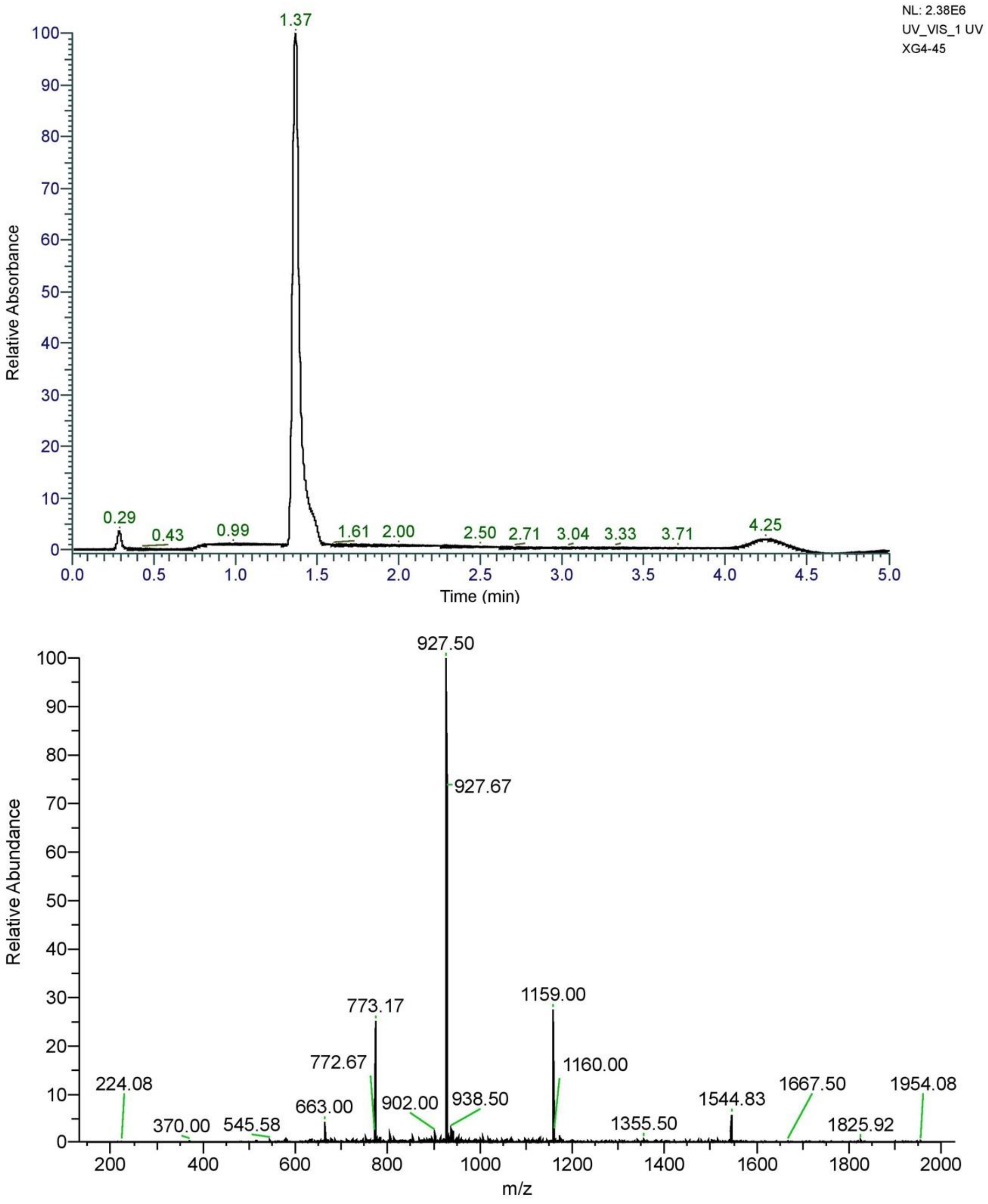

Figure S101. LCMS spectrum.

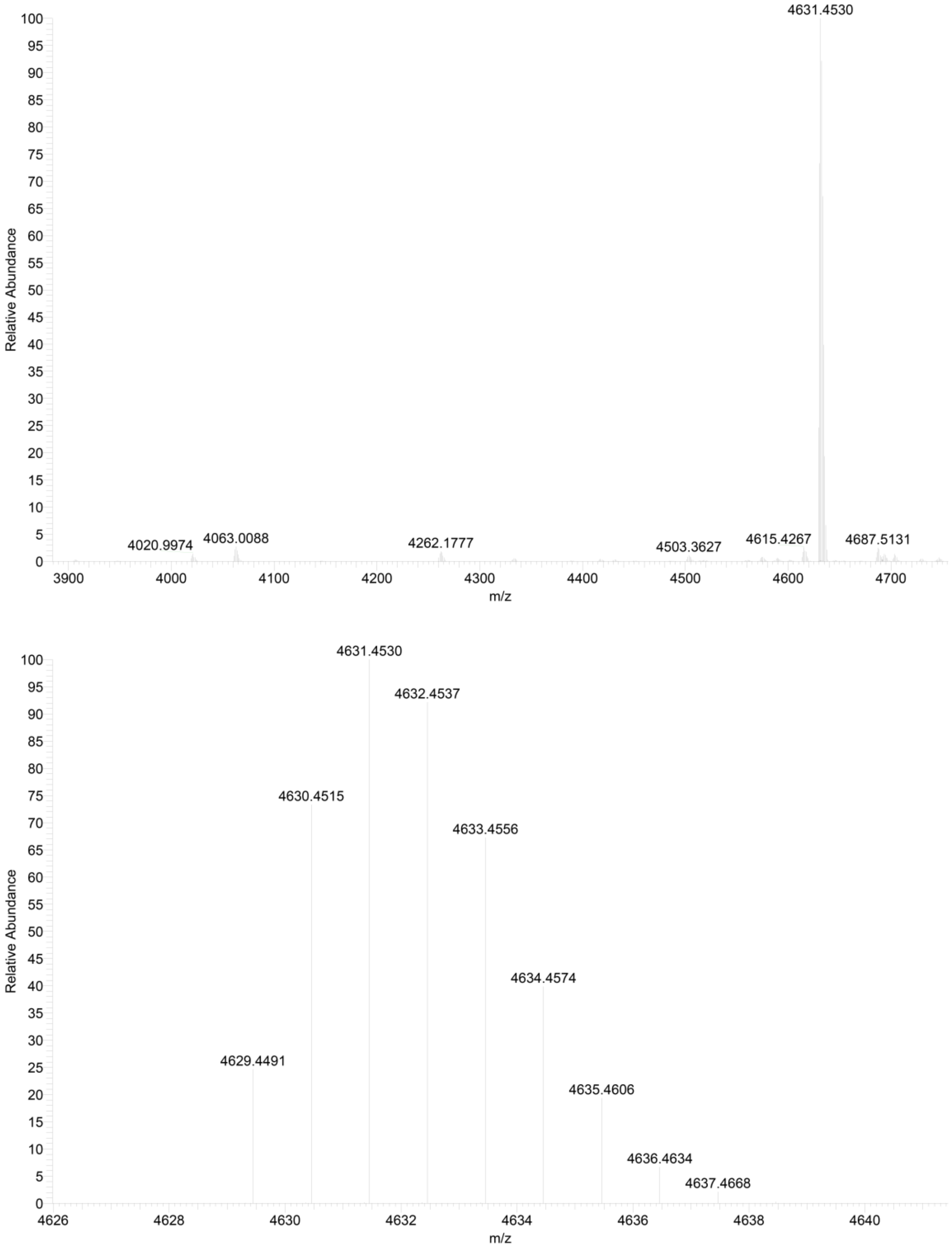

Figure S102. HRMS spectrum.

sr-**X34** ((*K*L)$_8$(*K*KL)$_4$(*K*LL)$_2$*K*LKK) was manually synthesized using TentaGel S RAM resin (393.4 mg, 0.09 mmol, 0.22 mmol·g^{-1}), the dendrimer was obtained as a white foamy solid after preparative RP-HPLC purification (101.7 mg, 17.8%). Analytical RP-HPLC: t_R = 1.36 min (100% A to 100% B in 3.5 min, λ= 214 nm). MS (ESI+): $C_{228}H_{442}N_{60}O_{38}$ calc./obs. 4629.45/4629.45 $[M]^+$.

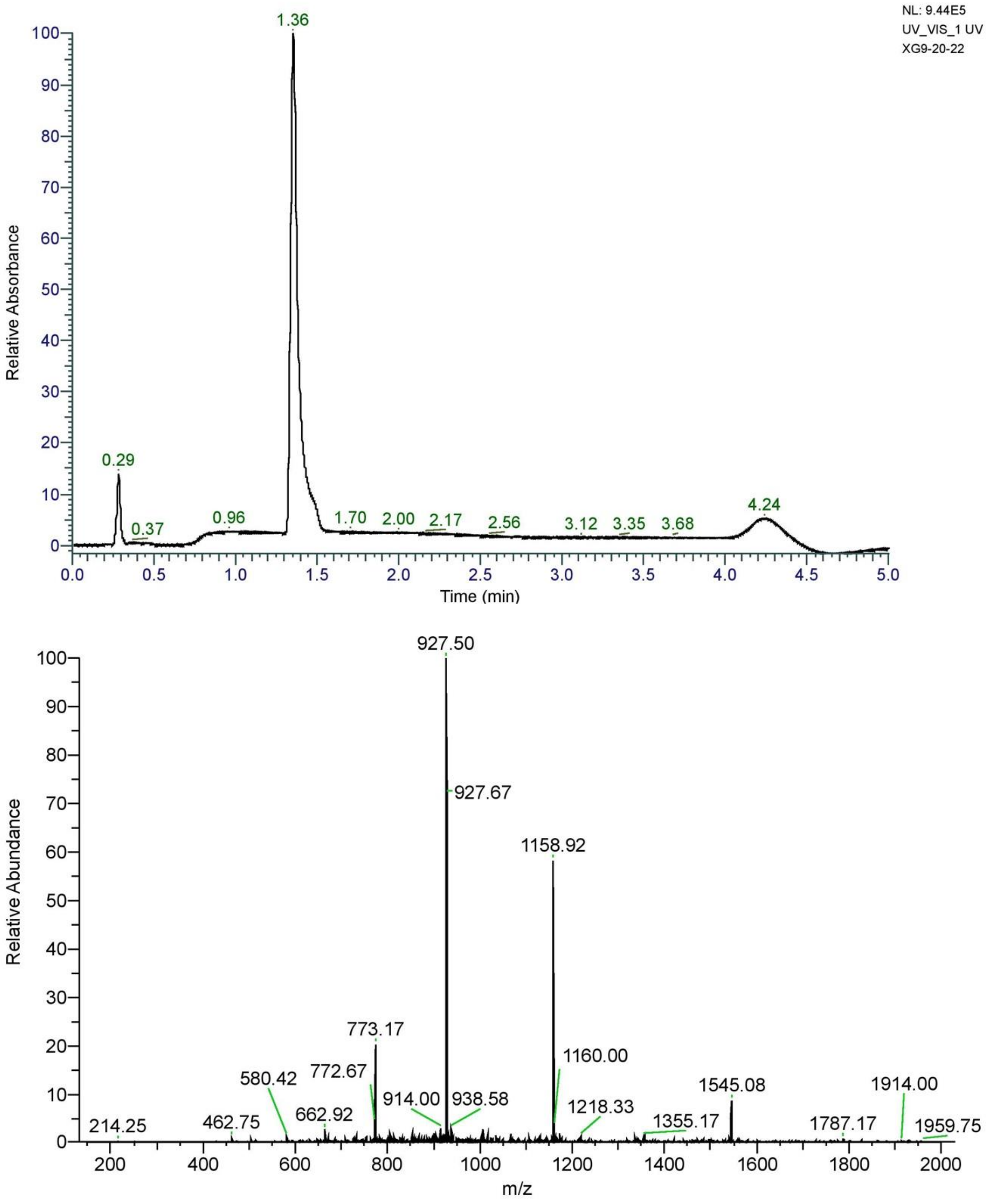

Figure S103. LCMS spectrum.

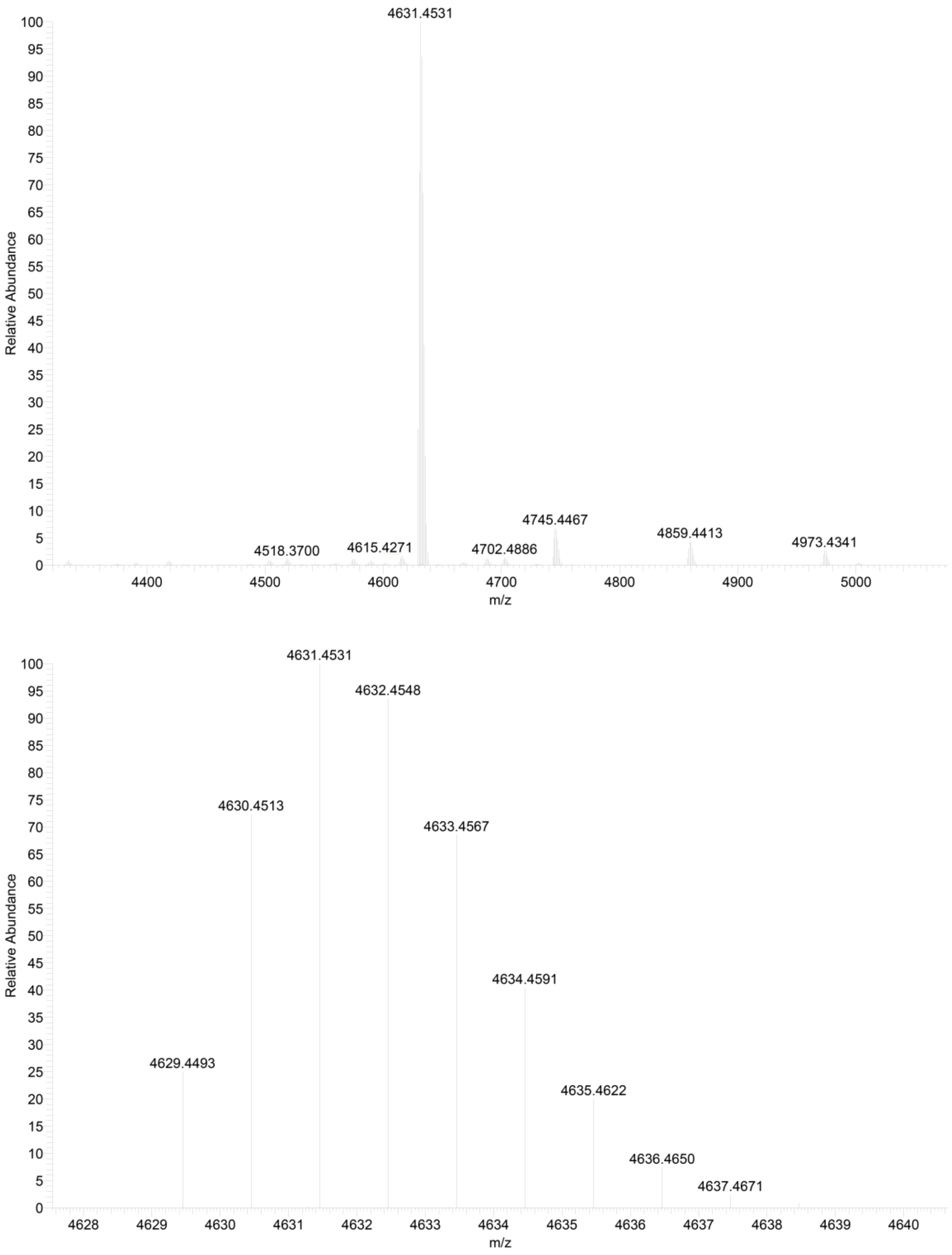

Figure S104. HRMS spectrum.

sr-**X35** ((*K*L)$_8$(*K*LK)$_4$(*K*LK)$_2$*K*LLL) was manually synthesized using TentaGel S RAM resin (393.4 mg, 0.09 mmol, 0.22 mmol·g^{-1}), the dendrimer was obtained as a white foamy solid after preparative RP-HPLC purification (34.4 mg, 6.0%). Analytical RP-HPLC: t_R = 1.36 min (100% A to 100% B in 3.5 min, λ= 214 nm). MS (ESI+): $C_{228}H_{442}N_{60}O_{38}$ calc./obs. 4629.45/4629.45 [M]$^+$.

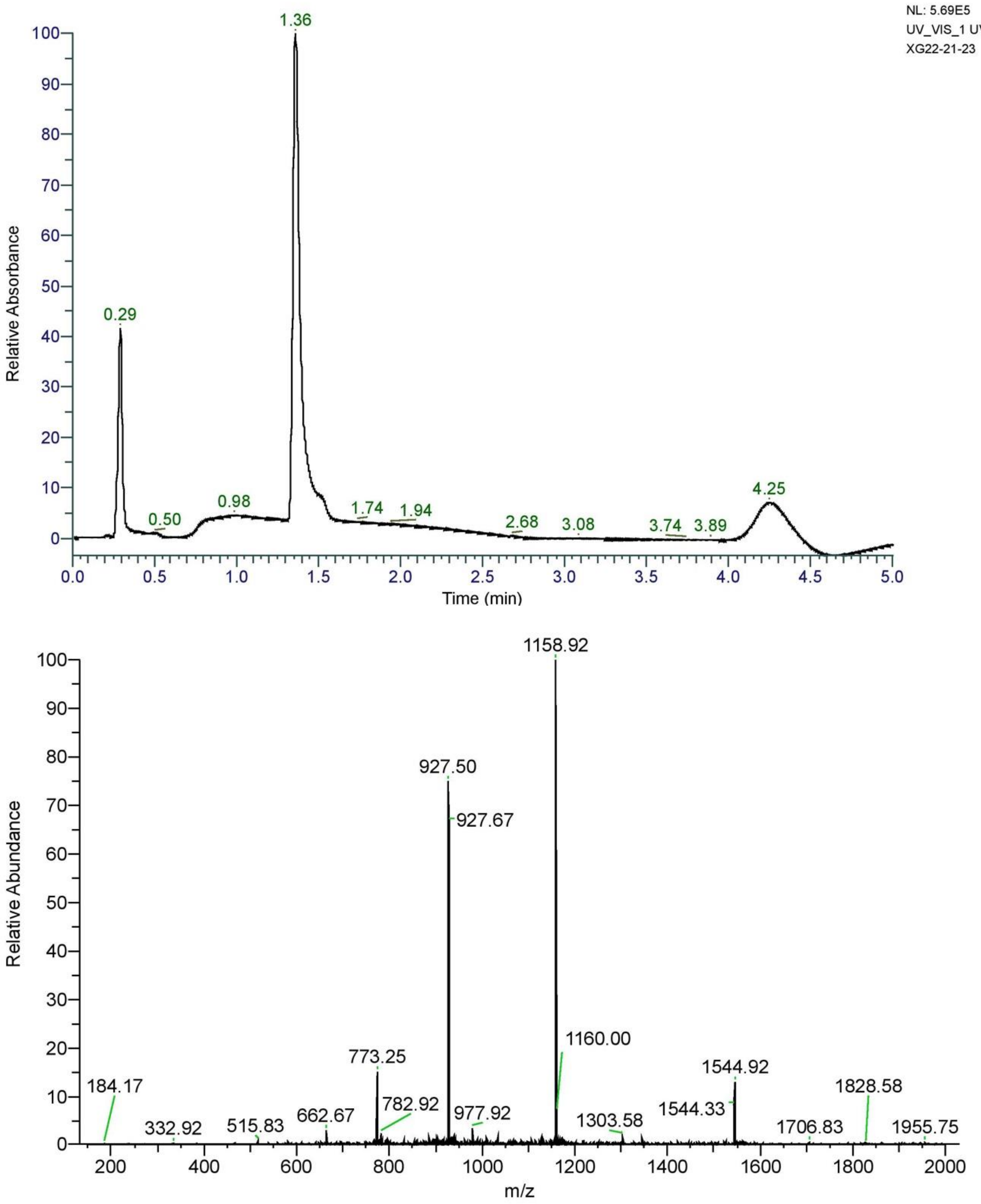

Figure S105. LCMS spectrum.

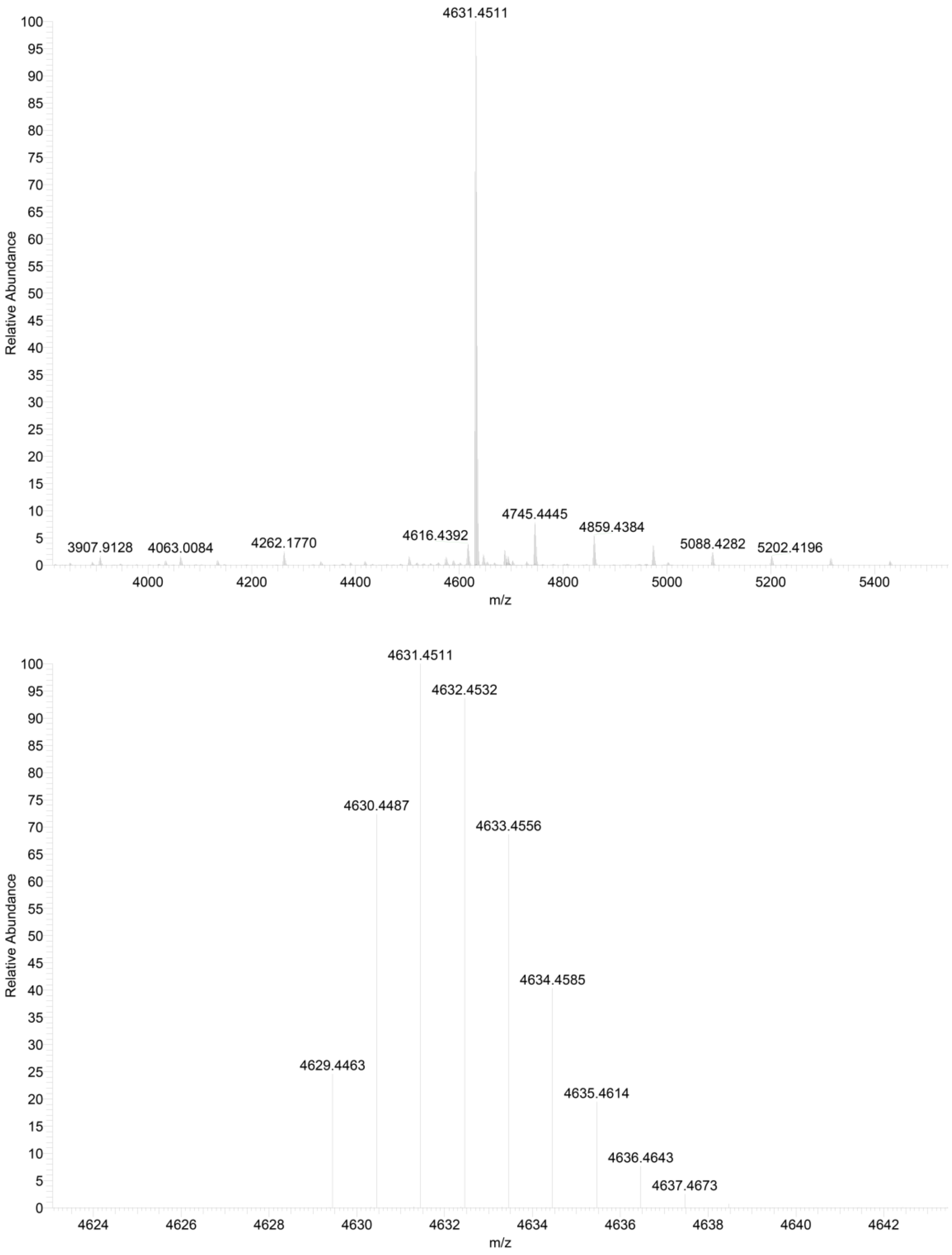

Figure S106. HRMS spectrum.

sr-**X36** ((*K*L)8(*K*KL)4(*K*LL)2*K*KLK) was manually synthesized using TentaGel S RAM resin (382.9 mg, 0.08 mmol, 0.22 mmol·g^{-1}), the dendrimer was obtained as a white foamy solid after preparative RP-HPLC purification (81.5 mg, 13.6%). Analytical RP-HPLC: t_R = 1.37 min (100% A to 100% B in 3.5 min, λ= 214 nm). MS (ESI+): $C_{228}H_{442}N_{60}O_{38}$ calc./obs. 4629.45/4629.45 $[M]^+$.

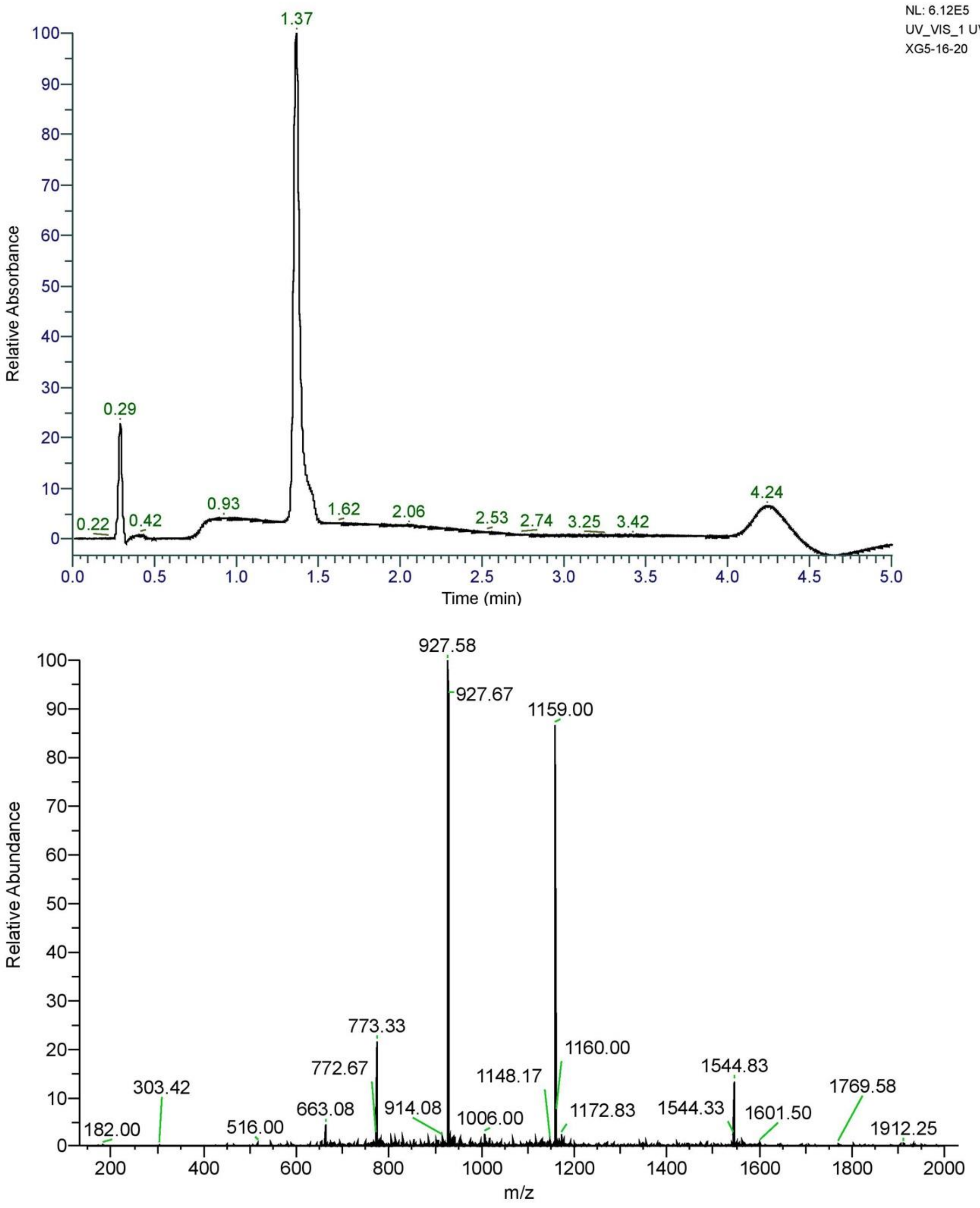

Figure S107. LCMS spectrum.

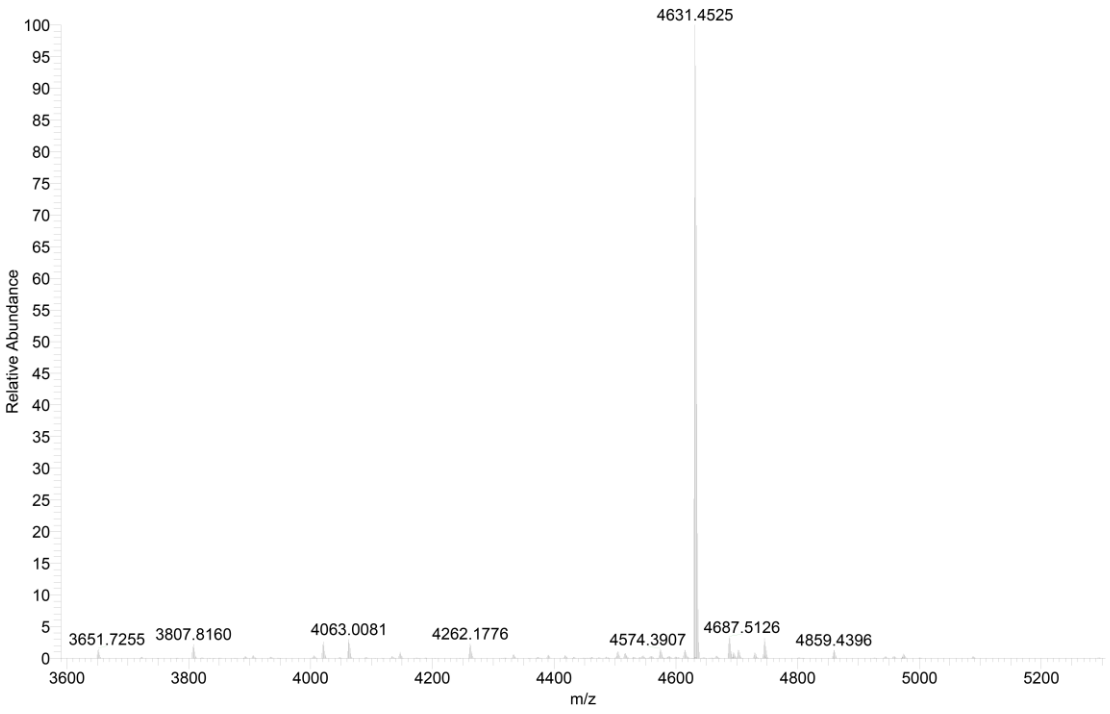

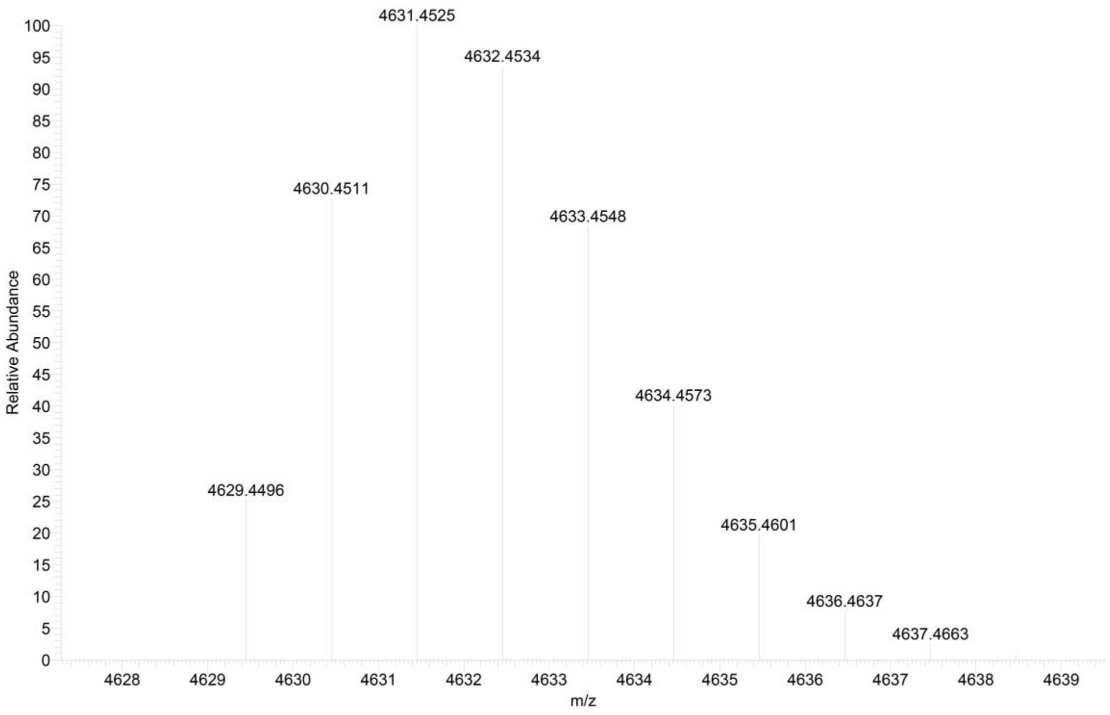

Figure S108. HRMS spectrum.

sr-**X37** ((*K*L)$_8$(*K*KL)$_4$(*K*LL)$_2$*K*KKL) was manually synthesized using TentaGel S RAM resin (345.5 mg, 0.08 mmol, 0.22 mmol·g^{-1}), the dendrimer was obtained as a white foamy solid after preparative RP-HPLC purification (93.0 mg, 17.1%). Analytical RP-HPLC: t_R = 1.35 min (100% A to 100% B in 3.5 min, λ= 214 nm). MS (ESI+): $C_{228}H_{442}N_{60}O_{38}$ calc./obs. 4629.45/4629.45 $[M]^+$.

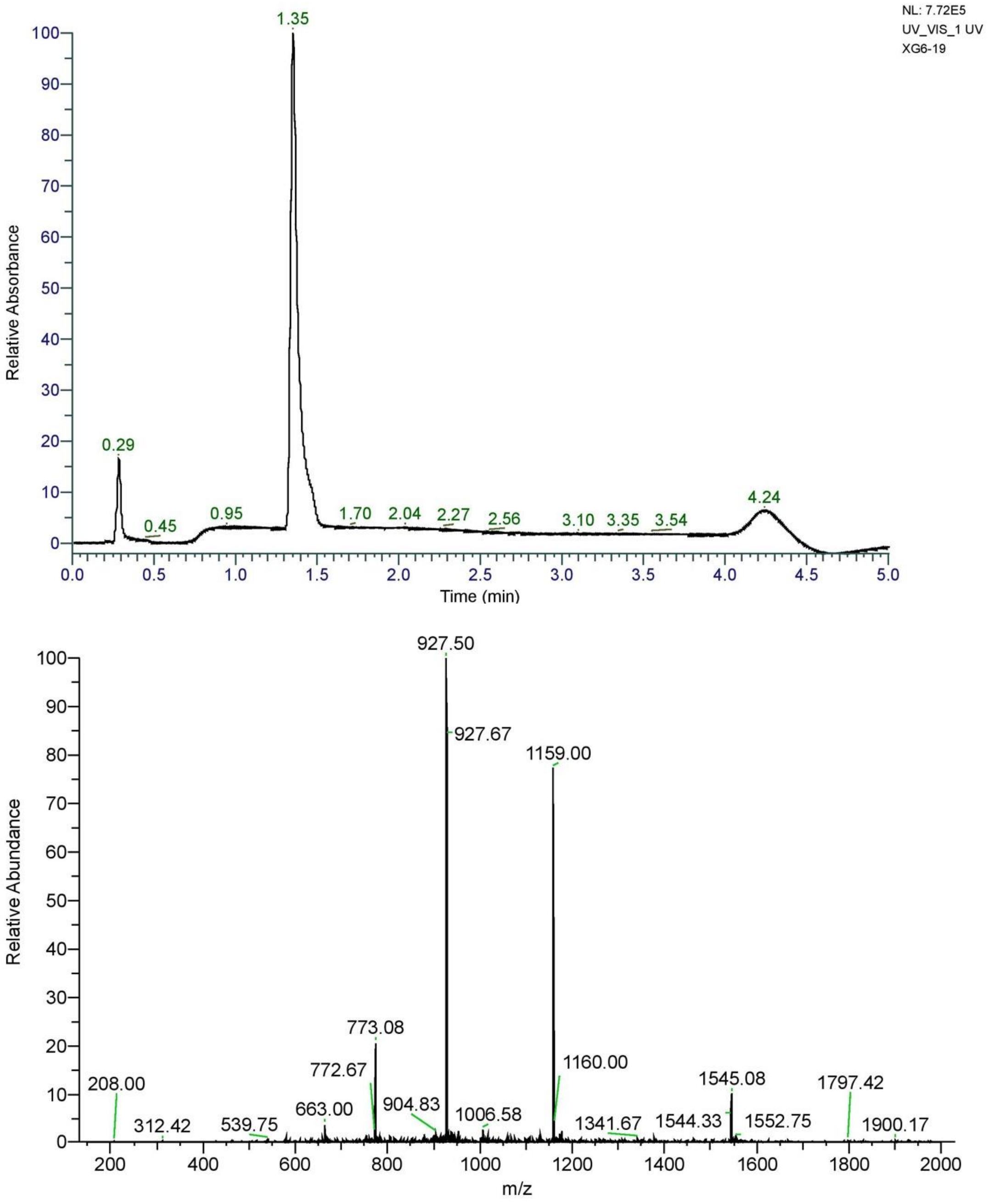

Figure S109. LCMS spectrum.

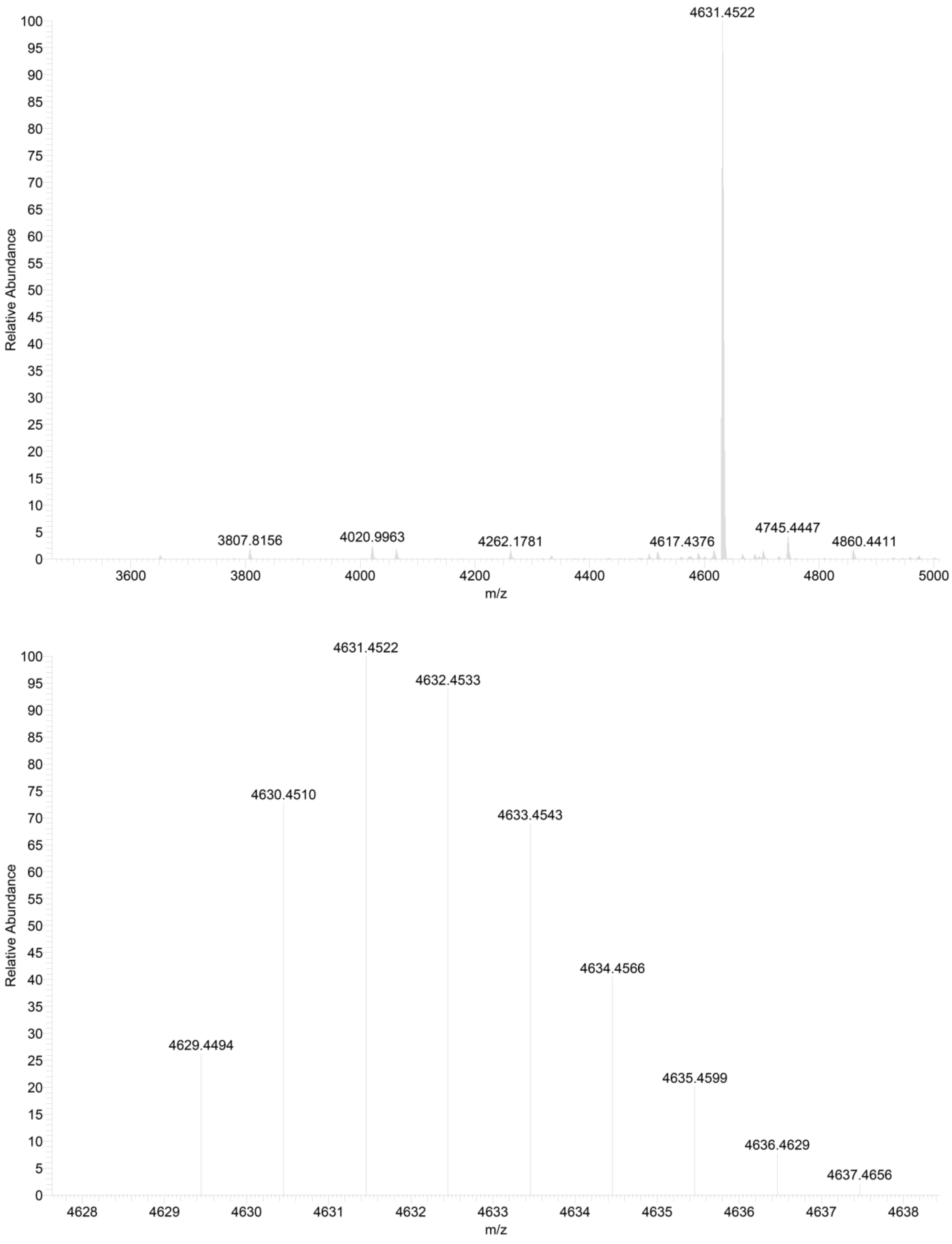

Figure S110. HRMS spectrum.

sr-**X38** ((*K*L)$_8$(*K*KL)$_4$(*K*LK)$_2$*K*LLL) was manually synthesized using TentaGel S RAM resin (393.4 mg, 0.09 mmol, 0.22 mmol·g^{-1}), the dendrimer was obtained as a white foamy solid after preparative RP-HPLC purification (139.0 mg, 24.3%). Analytical RP-HPLC: t_R = 1.36 min (100% A to 100% B in 3.5 min, λ= 214 nm). MS (ESI+): $C_{228}H_{442}N_{60}O_{38}$ calc./obs. 4629.45/4629.45 [M]$^+$.

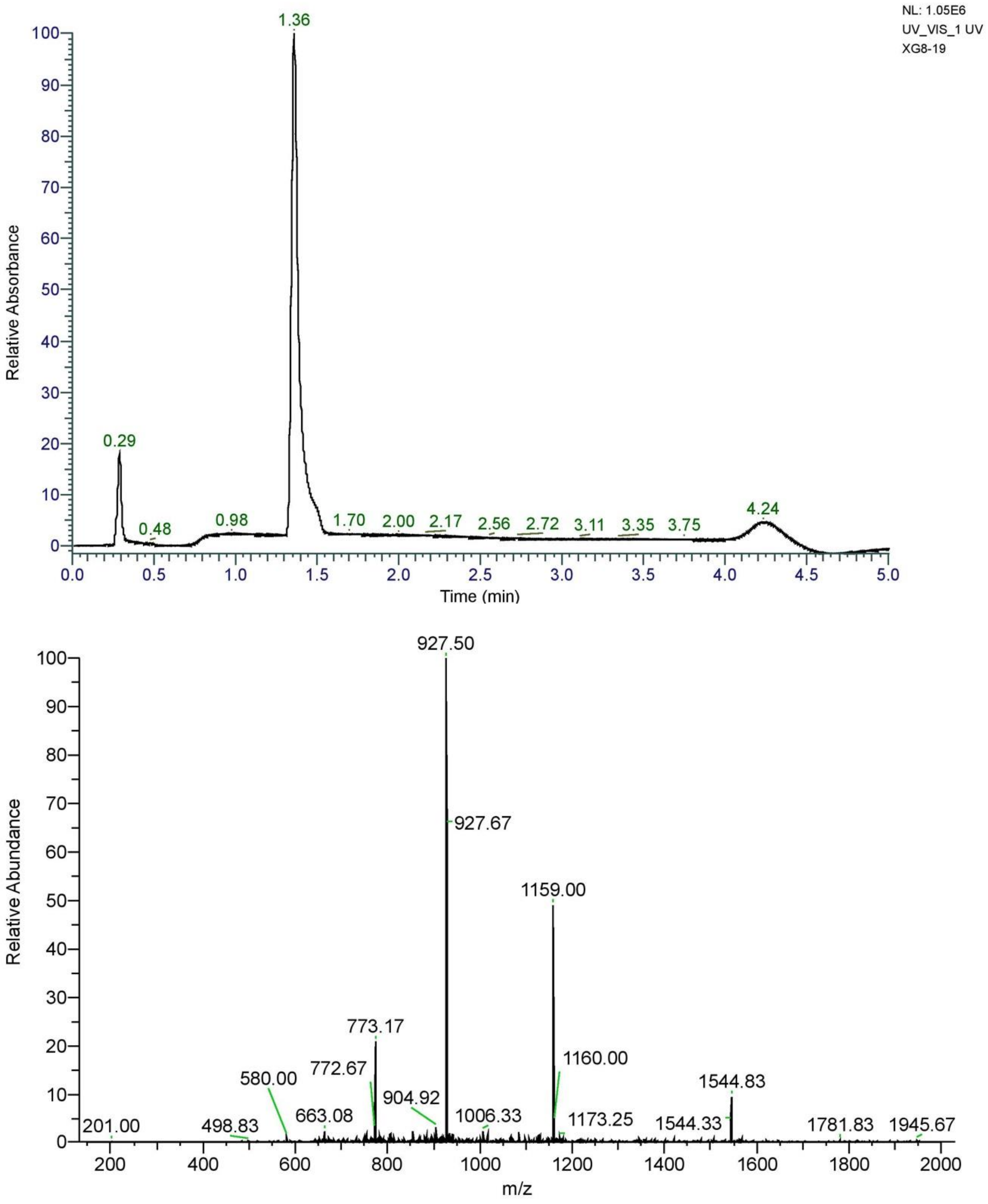

Figure S111. LCMS spectrum.

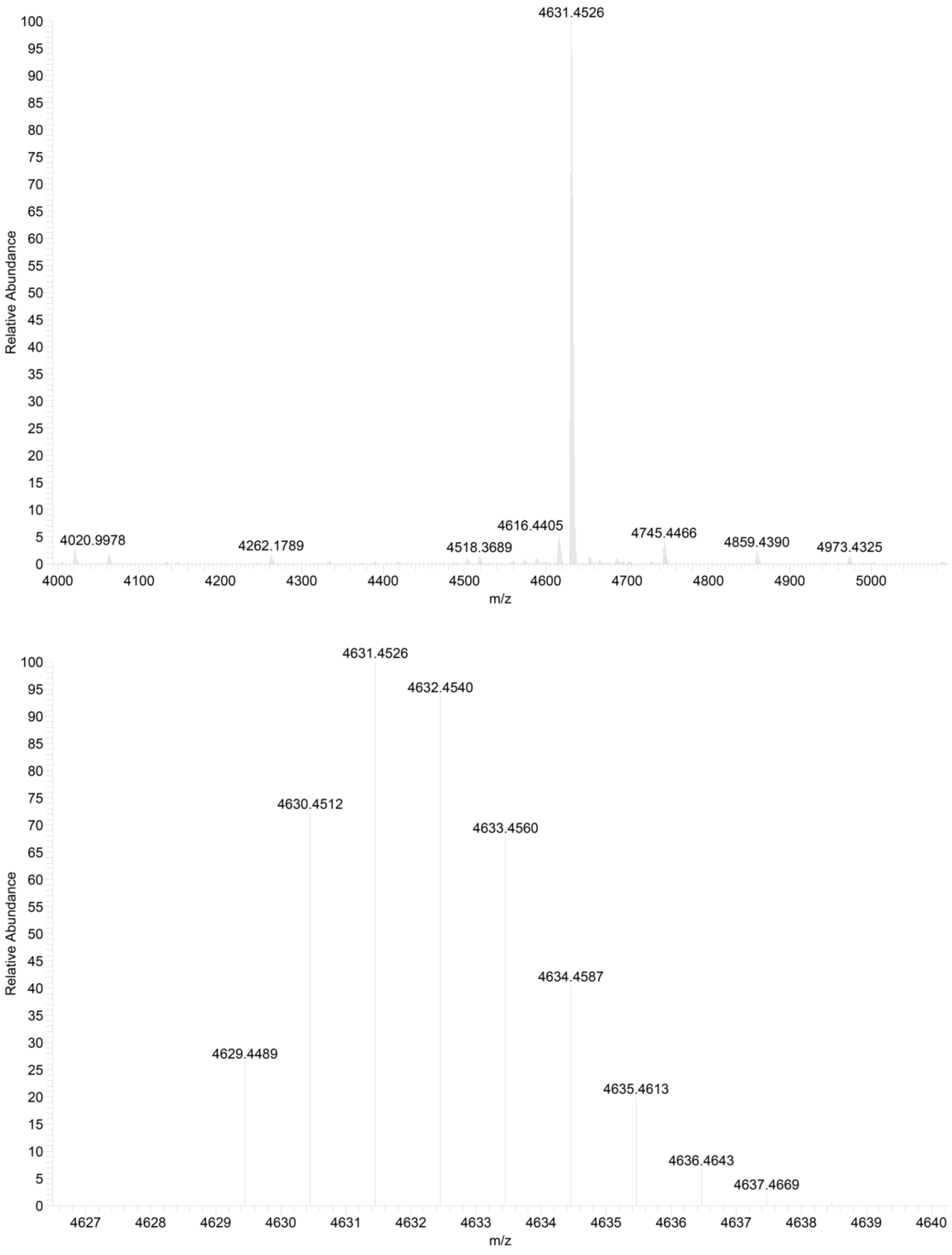

Figure S112. HRMS spectrum.

sr-**X39** ((*K*L)$_8$(*K*LK)$_4$(*K*LL)$_2$*K*LKK) was manually synthesized using TentaGel S RAM resin (393.4 mg, 0.09 mmol, 0.22 mmol·g^{-1}), the dendrimer was obtained as a white foamy solid after preparative RP-HPLC purification (73.0 mg, 12.8%). Analytical RP-HPLC: t_R = 1.34 min (100% A to 100% B in 3.5 min, λ= 214 nm). MS (ESI+): $C_{228}H_{442}N_{60}O_{38}$ calc./obs. 4629.45/4629.45 $[M]^+$.

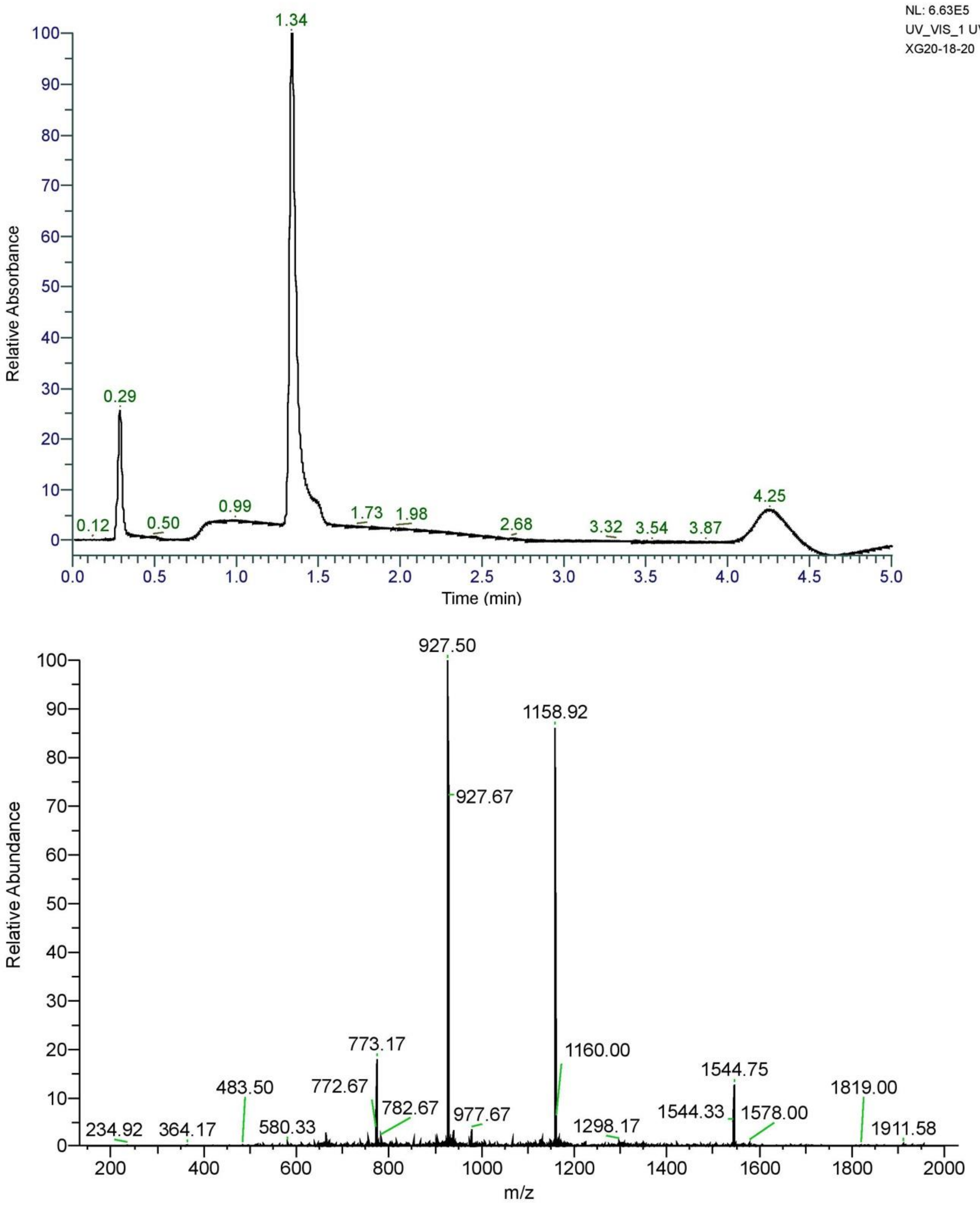

Figure S113. LCMS spectrum.

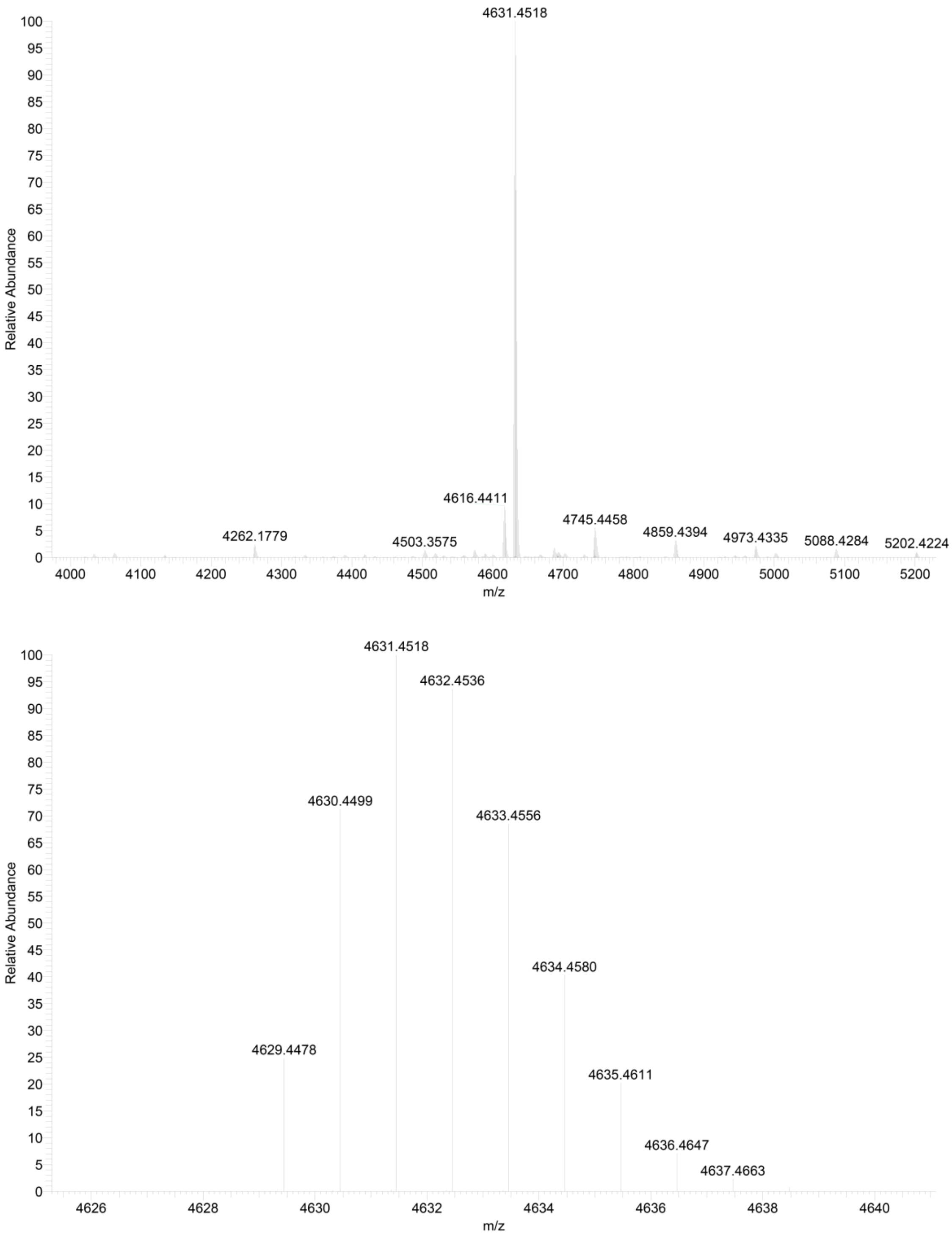

Figure S114. HRMS spectrum.

sr-**X40** ((LK)$_8$(*K*LK)$_4$(*K*LL)$_2$*K*LKK) was manually synthesized using TentaGel S RAM resin (393.4 mg, 0.09 mmol, 0.22 mmol·g^{-1}), the dendrimer was obtained as a white foamy solid after preparative RP-HPLC purification (104.5 mg, 18.3%). Analytical RP-HPLC: t_R = 1.32 min (100% A to 100% B in 3.5 min, λ= 214 nm). MS (ESI+): $C_{228}H_{442}N_{60}O_{38}$ calc./obs. 4629.45/4629.45 $[M]^+$.

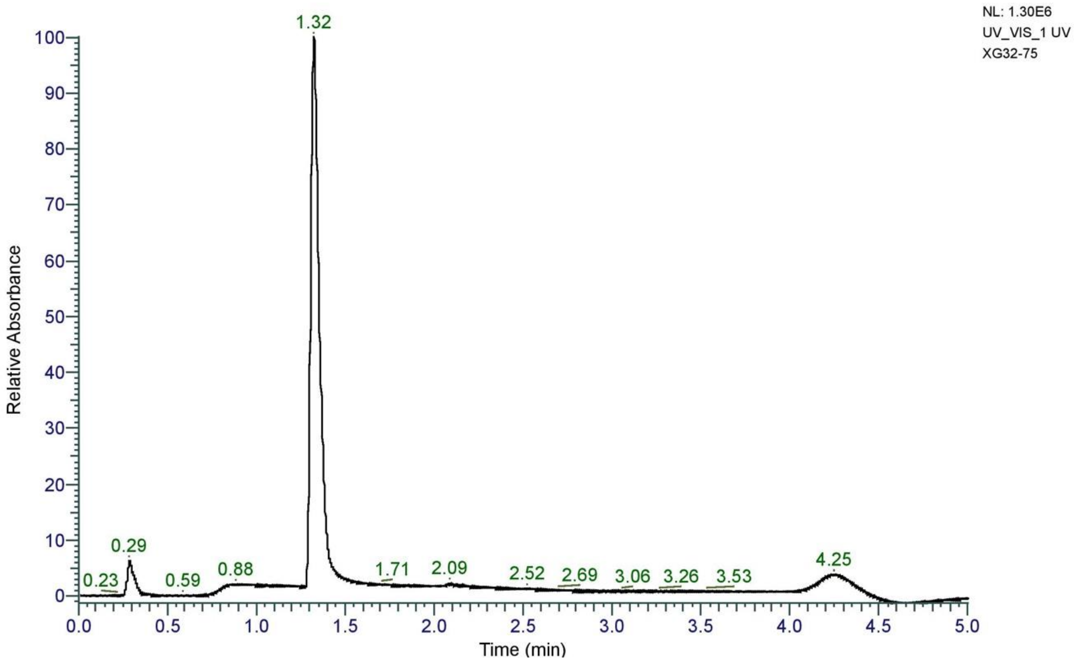

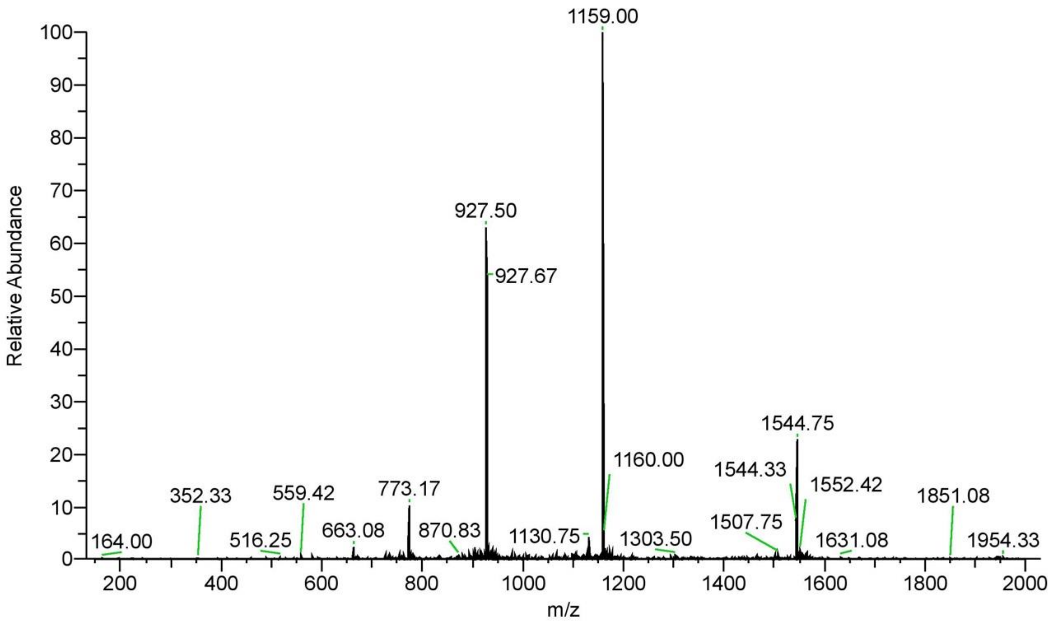

Figure S115. LCMS spectrum.

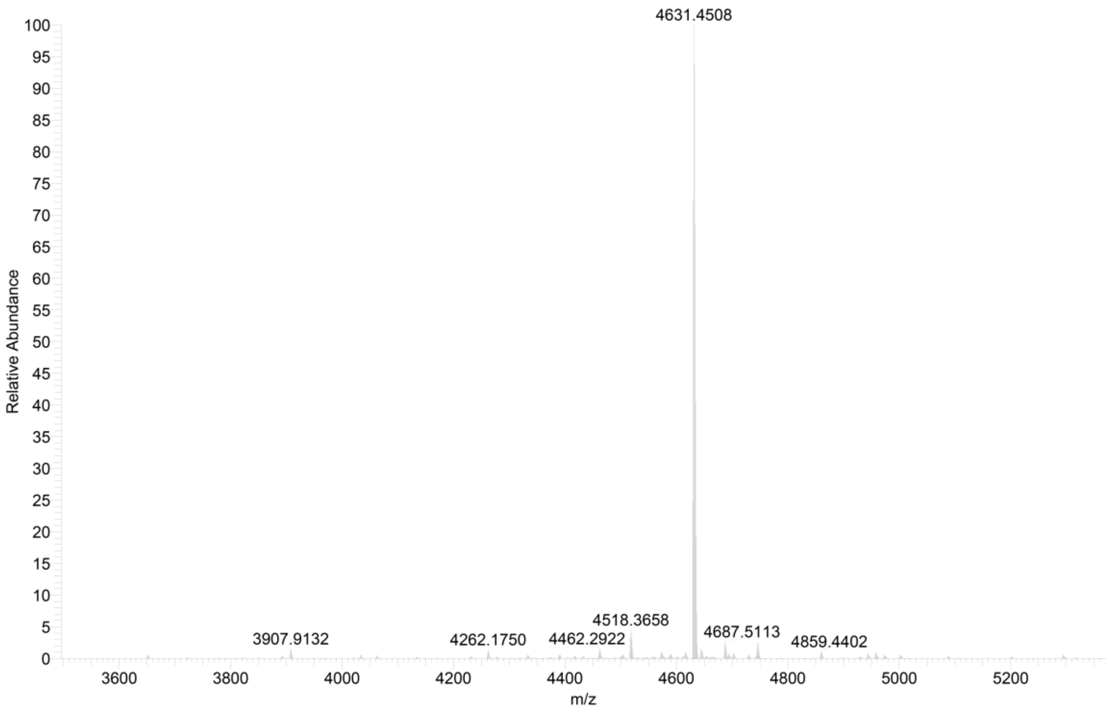

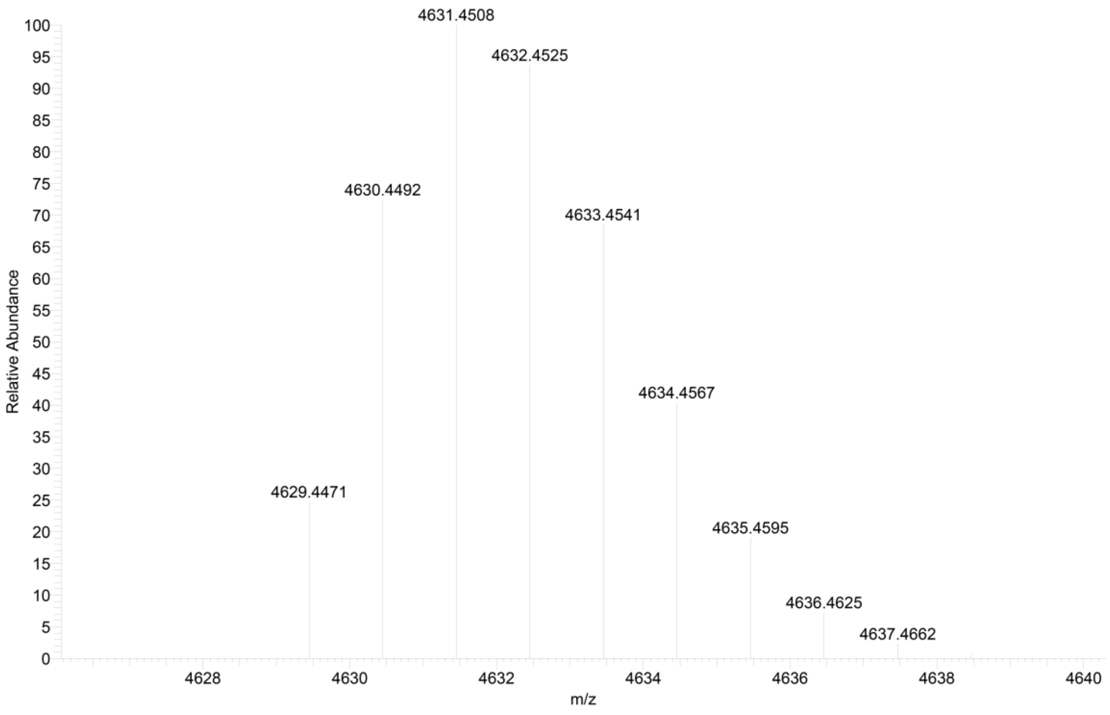

Figure S116. HRMS spectrum.

sr-**X41** ((*K*L)$_8$(*K*LK)$_4$(*K*LL)$_2$*K*KLK) was manually synthesized using TentaGel S RAM resin (393.4 mg, 0.09 mmol, 0.22 mmol·g^{-1}), the dendrimer was obtained as a white foamy solid after preparative RP-HPLC purification (80.5 mg, 14.1%). Analytical RP-HPLC: t_R = 1.35 min (100% A to 100% B in 3.5 min, λ= 214 nm). MS (ESI+): $C_{228}H_{442}N_{60}O_{38}$ calc./obs. 4629.45/4629.45 $[M]^+$.

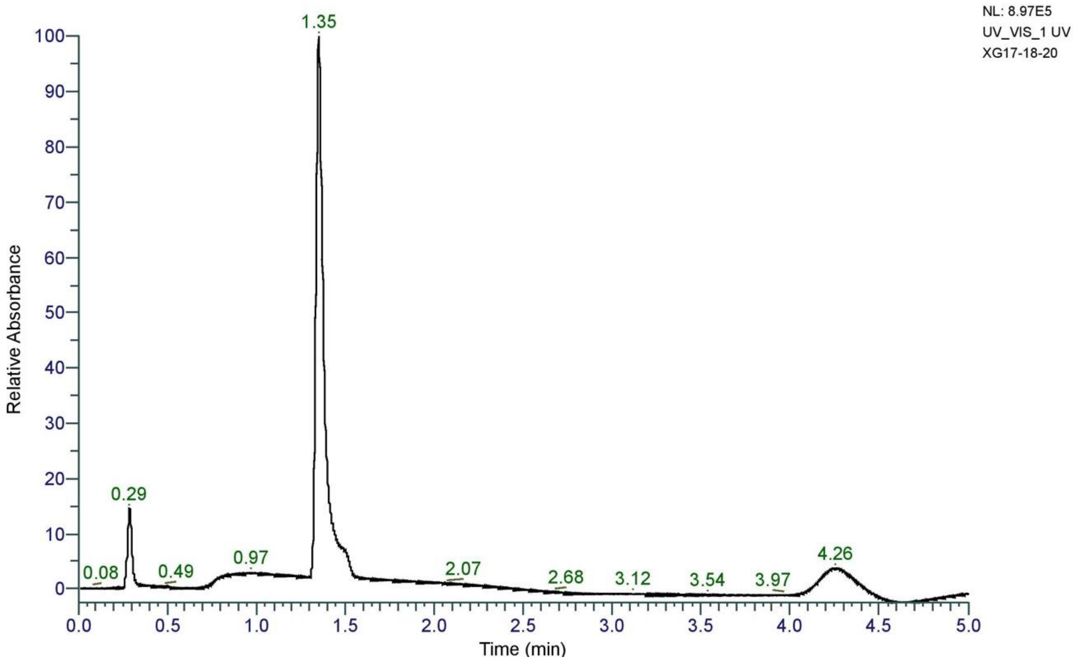

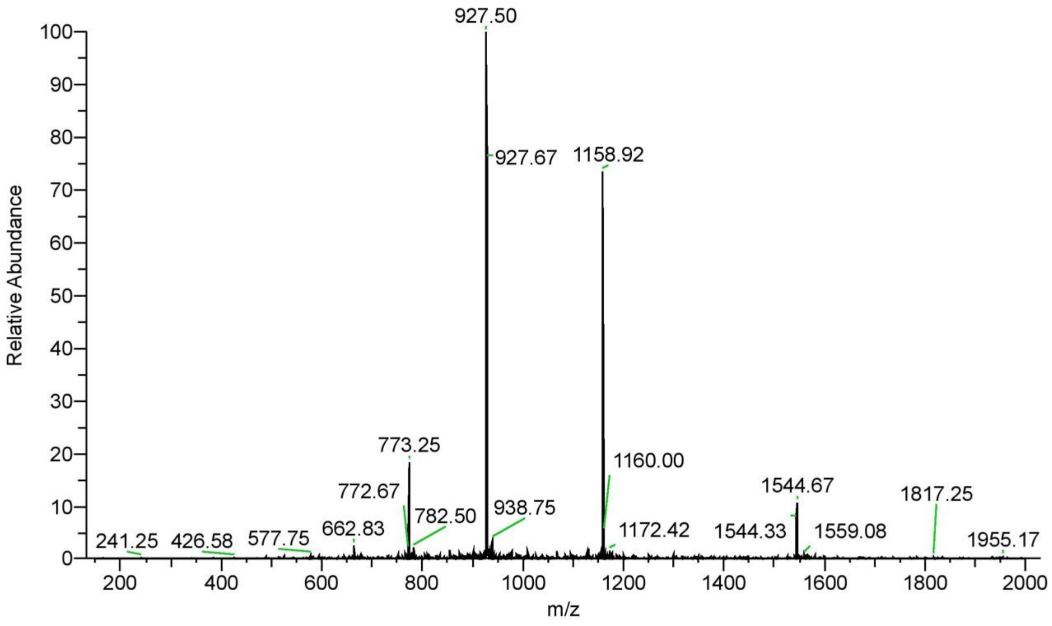

Figure S117. LCMS spectrum.

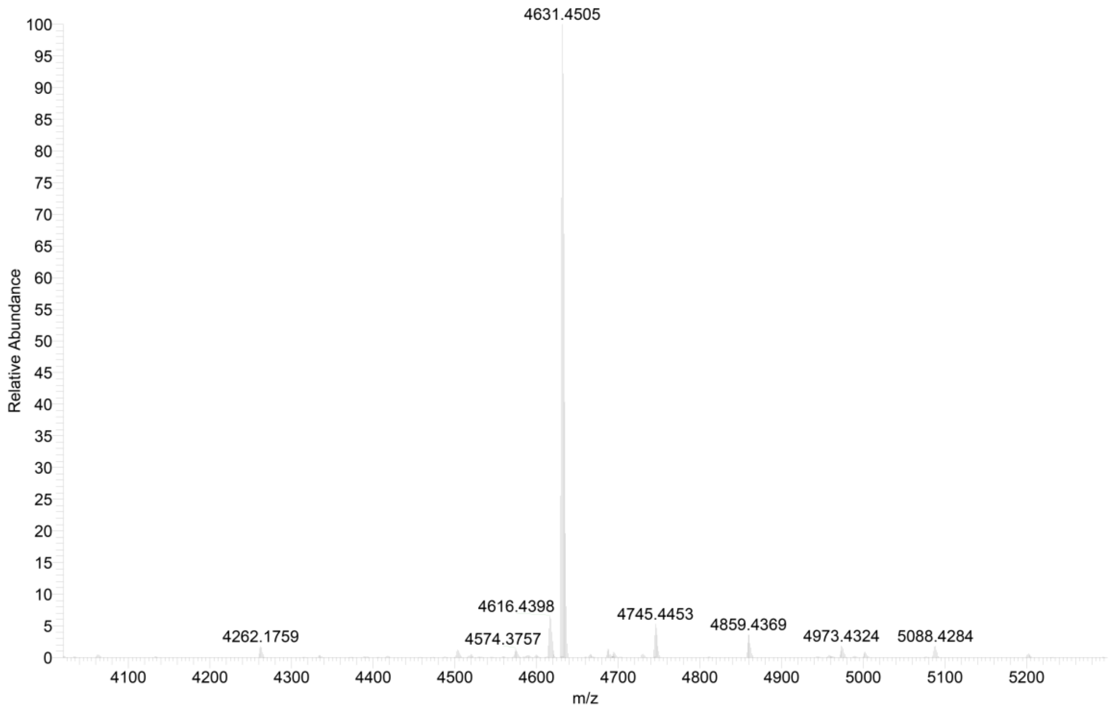

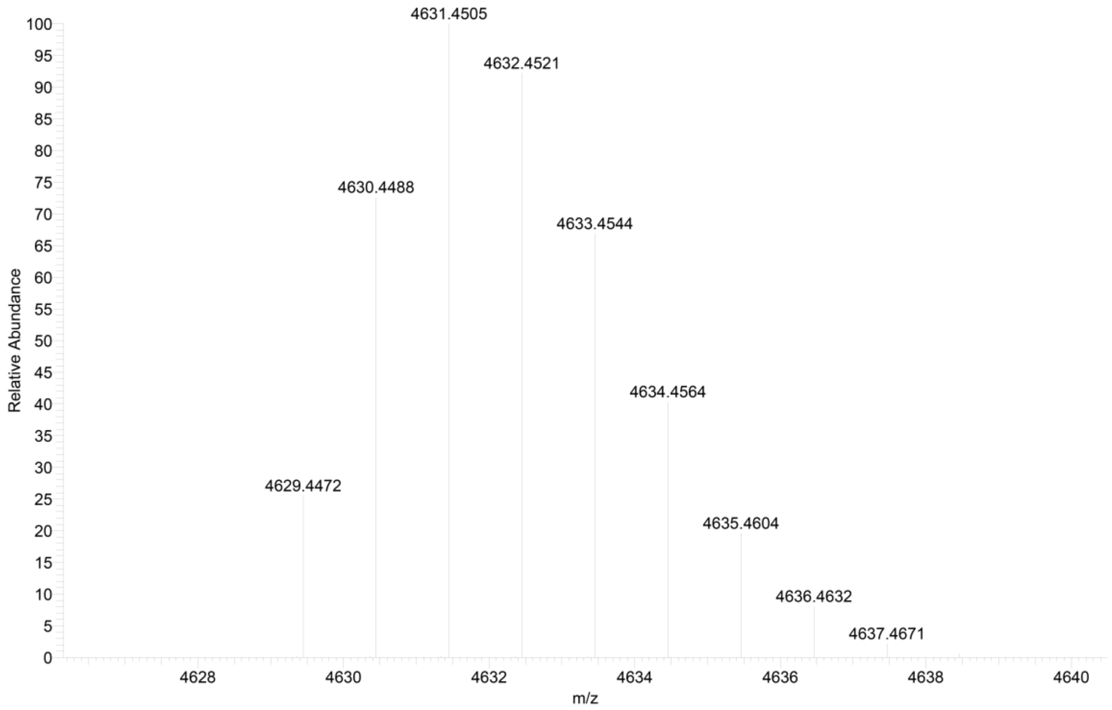

Figure S118. HRMS spectrum.

sr-**X42** $((LK)_8(\mathit{K}KL)_4(\mathit{K}LL)_2\mathit{K}KKL)$ was manually synthesized using TentaGel S RAM resin (393.4 mg, 0.09 mmol, 0.22 mmol·g^{-1}), the dendrimer was obtained as a white foamy solid after preparative RP-HPLC purification (30.4 mg, 5.3%). Analytical RP-HPLC: t_R = 1.32 min (100% A to 100% B in 3.5 min, λ= 214 nm). MS (ESI+): $C_{228}H_{442}N_{60}O_{38}$ calc./obs. 4629.45/4629.45 $[M]^+$.

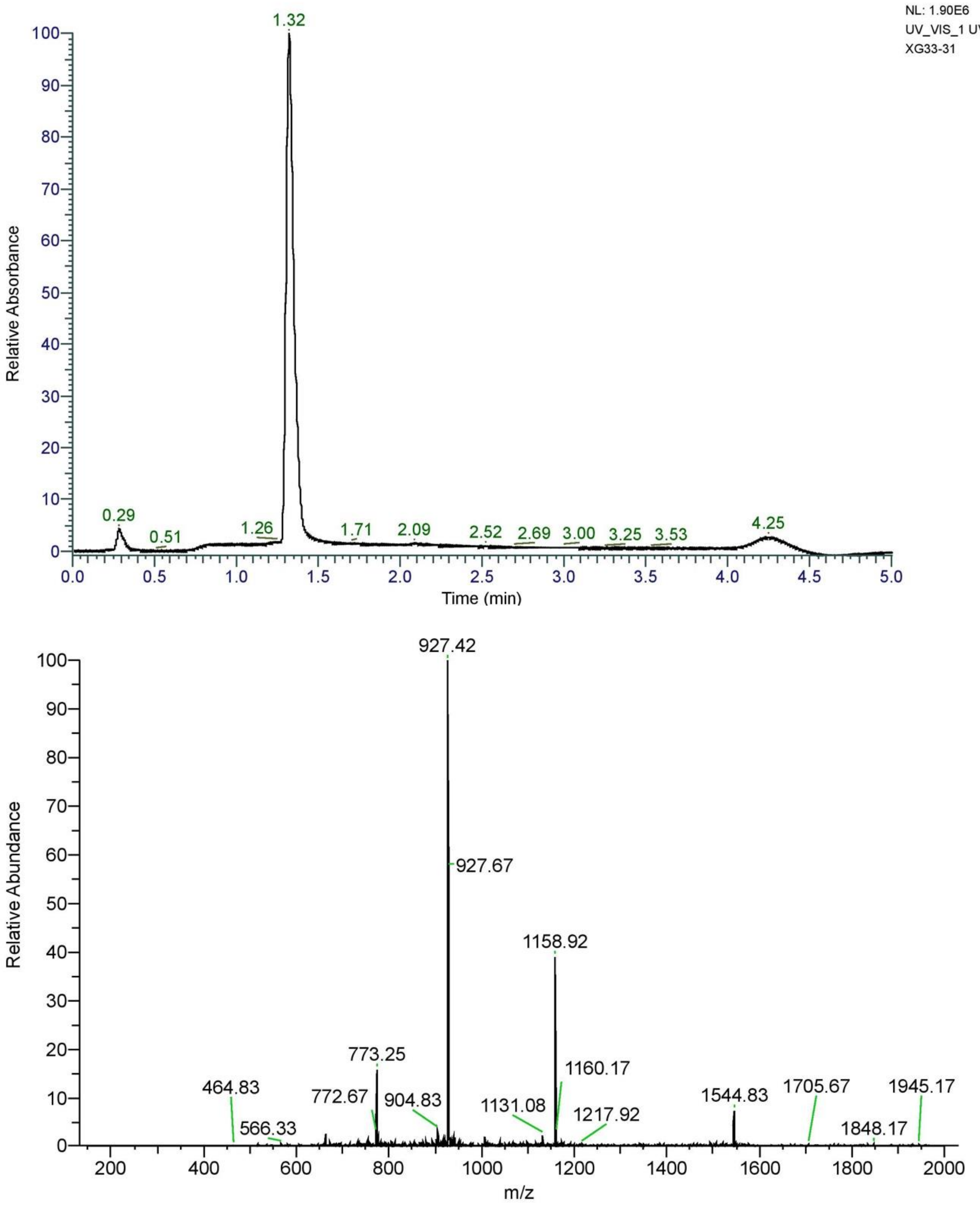

Figure S119. LCMS spectrum.

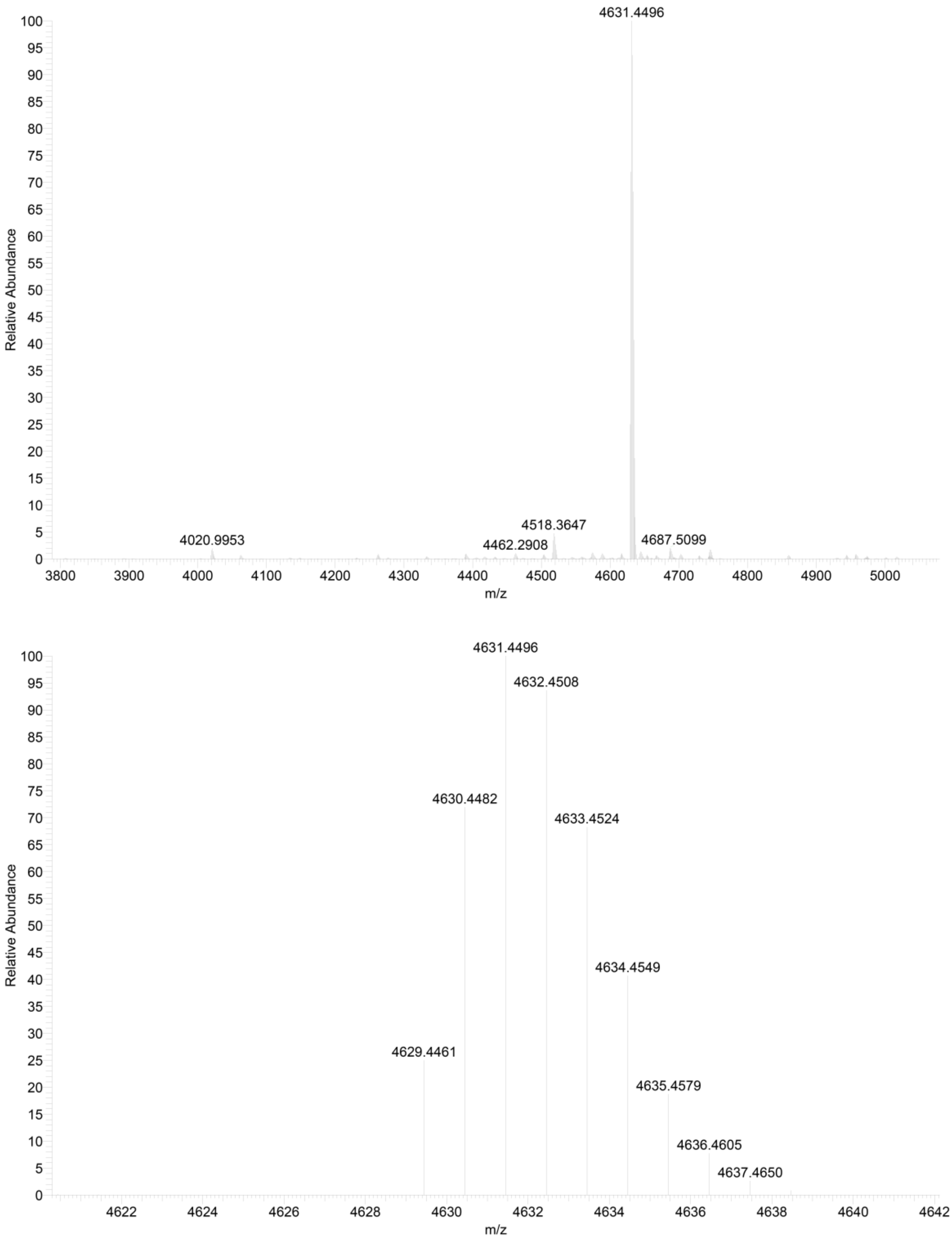

Figure S120. HRMS spectrum.

sr-**X43** ((*K*L)$_8$(*K*KLL)$_4$(*K*K)$_2$*K*KLL) was manually synthesized using TentaGel S RAM resin (393.4 mg, 0.09 mmol, 0.22 mmol·g^{-1}), the dendrimer was obtained as a white foamy solid after preparative RP-HPLC purification (118.5 mg, 19.8%). Analytical RP-HPLC: t_R = 1.40 min (100% A to 100% B in 3.5 min, λ= 214 nm). MS (ESI+): $C_{240}H_{465}N_{63}O_{40}$ calc./obs. 4870.63/4870.63 $[M]^+$.

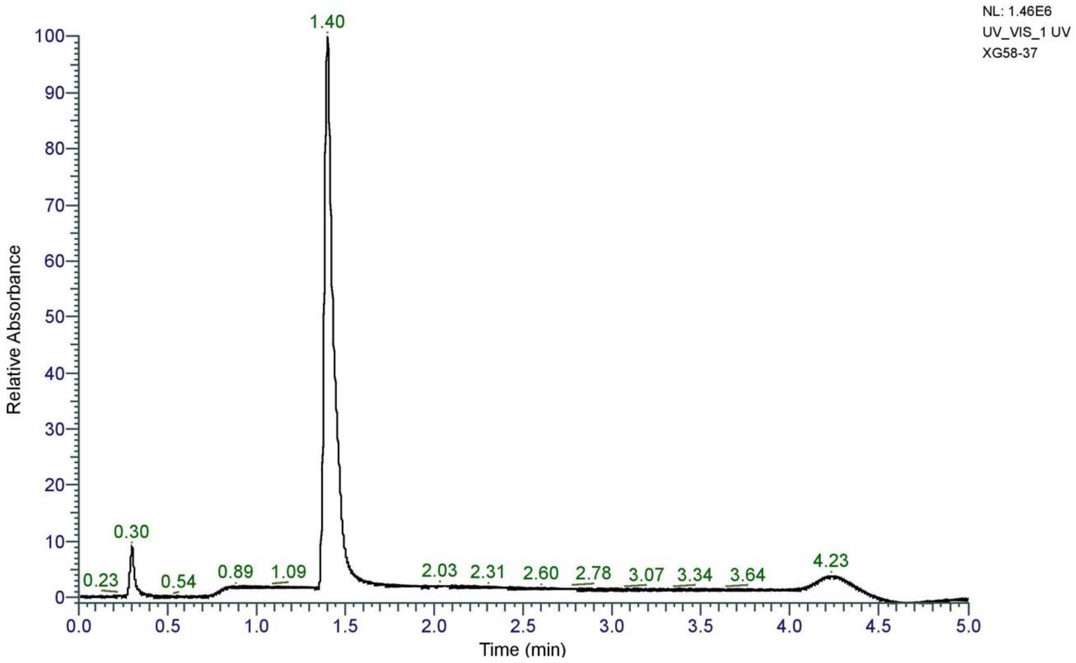

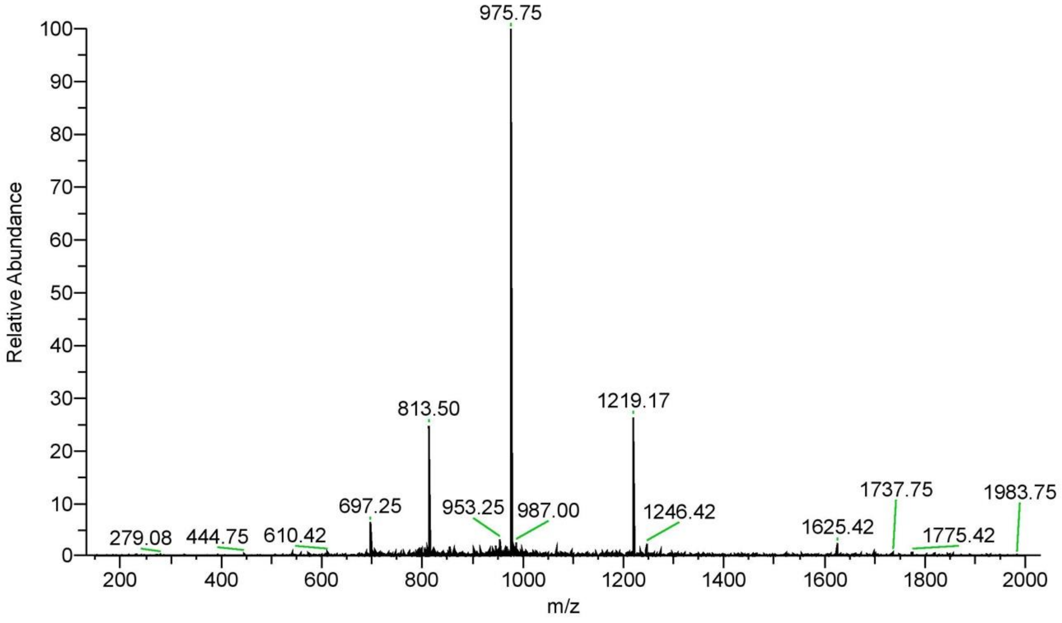

Figure S121. LCMS spectrum.

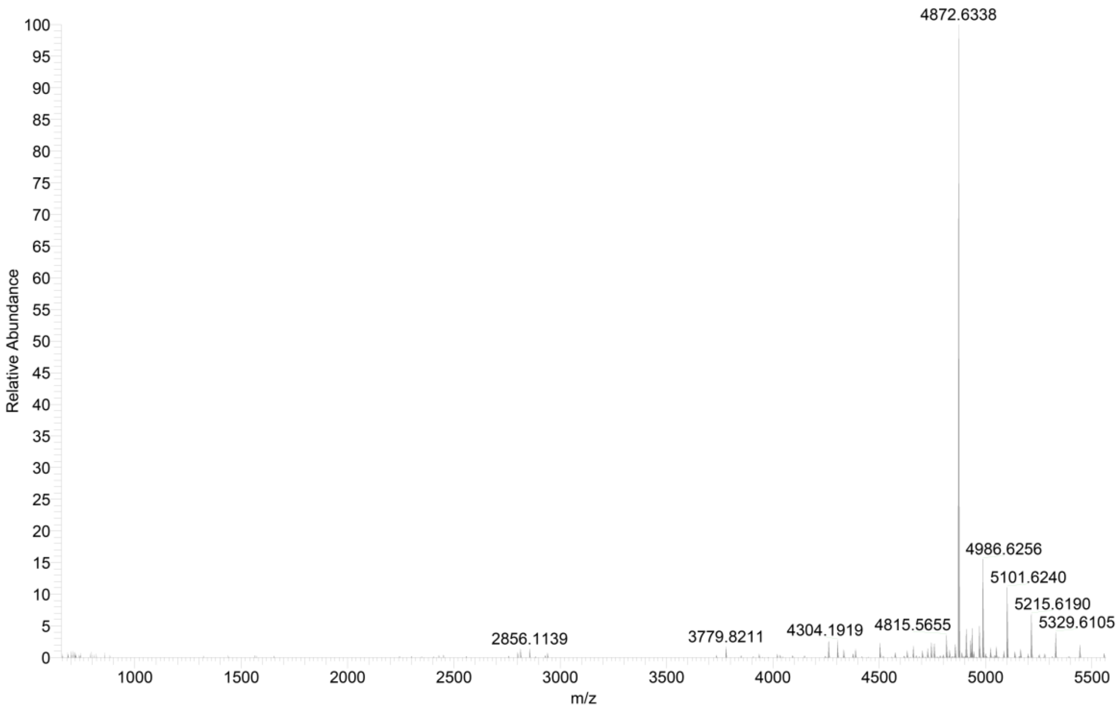

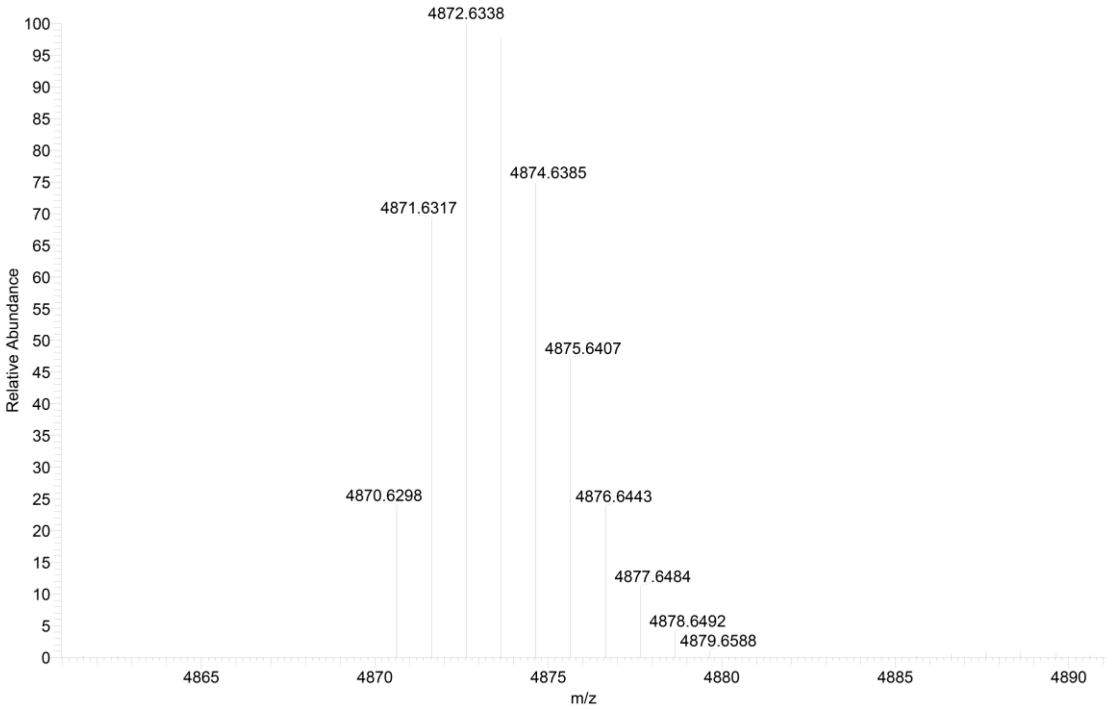

Figure S122. HRMS spectrum.

sr-**X44** ((*K*L)$_8$(*K*KL)$_4$(*K*LL)$_2$*K*KK) was manually synthesized using TentaGel S RAM resin (393.4 mg, 0.09 mmol, 0.22 mmol·g^{-1}), the dendrimer was obtained as a white foamy solid after preparative RP-HPLC purification (118.1 mg, 21.0%). Analytical RP-HPLC: t_R = 1.36 min (100% A to 100% B in 3.5 min, λ= 214 nm). MS (ESI+): $C_{222}H_{431}N_{59}O_{37}$ calc./obs. 4516.37/4516.37 [M]$^+$.

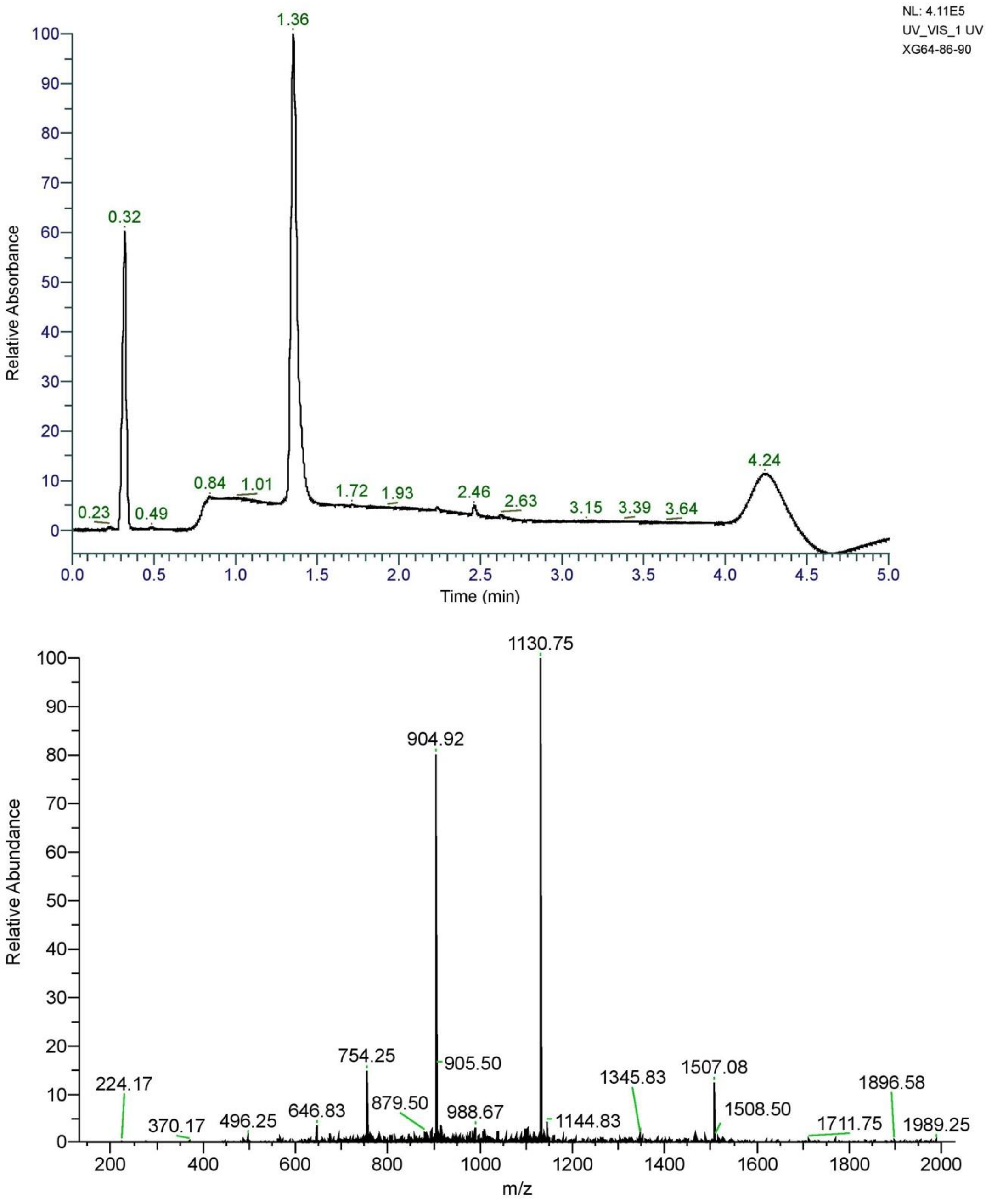

Figure S123. LCMS spectrum.

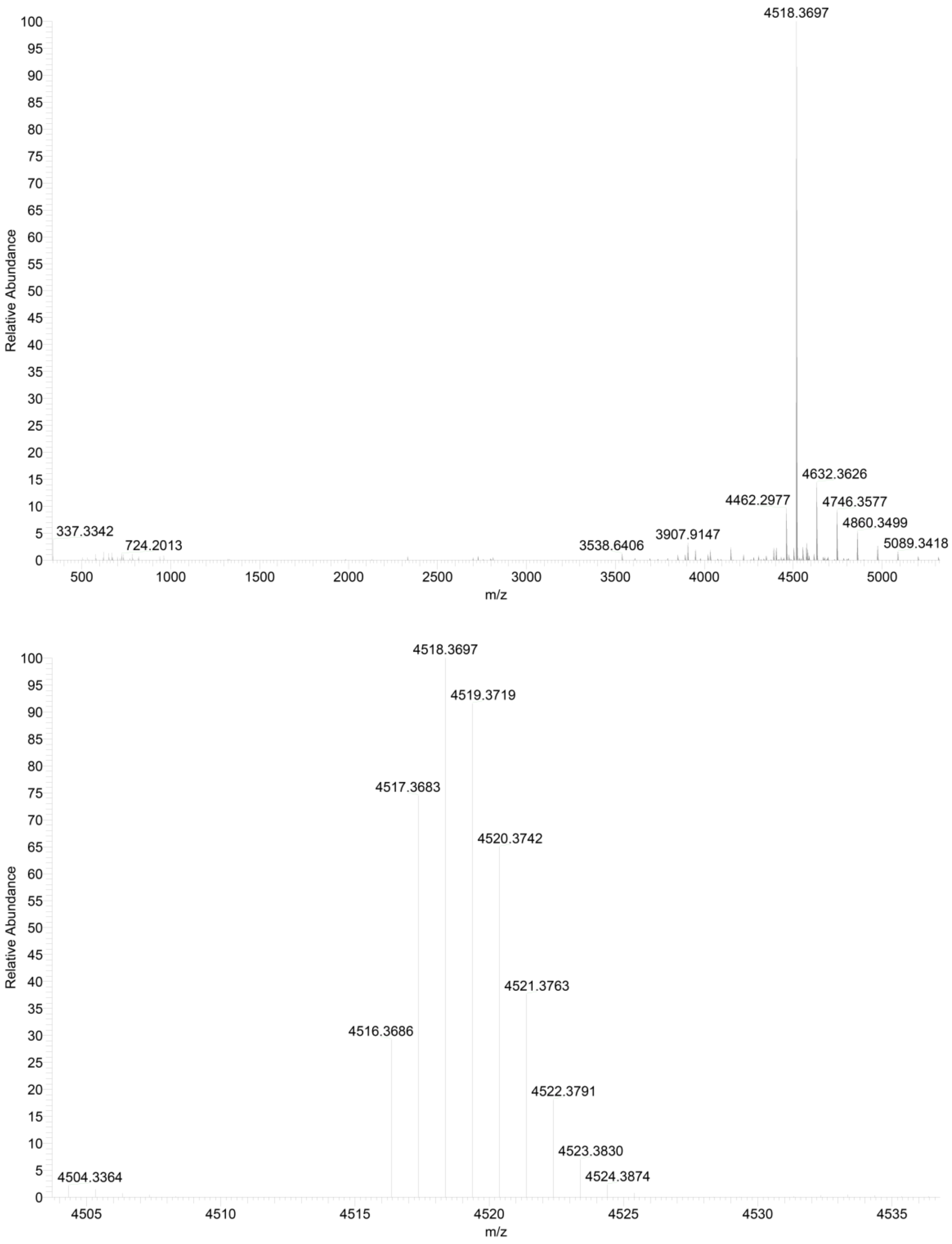

Figure S124. HRMS spectrum.

sr-**X45** ((*K*L)$_8$(*K*LK)$_4$(*K*KL)$_2$*K*LL) was manually synthesized using TentaGel S RAM resin (393.4 mg, 0.09 mmol, 0.22 mmol·g^{-1}), the dendrimer was obtained as a white foamy solid after preparative RP-HPLC purification (97.0 mg, 17.3%). Analytical RP-HPLC: t_R = 1.34 min (100% A to 100% B in 3.5 min, λ= 214 nm). MS (ESI+): $C_{222}H_{431}N_{59}O_{37}$ calc./obs. 4516.37/4516.35 [M]$^+$.

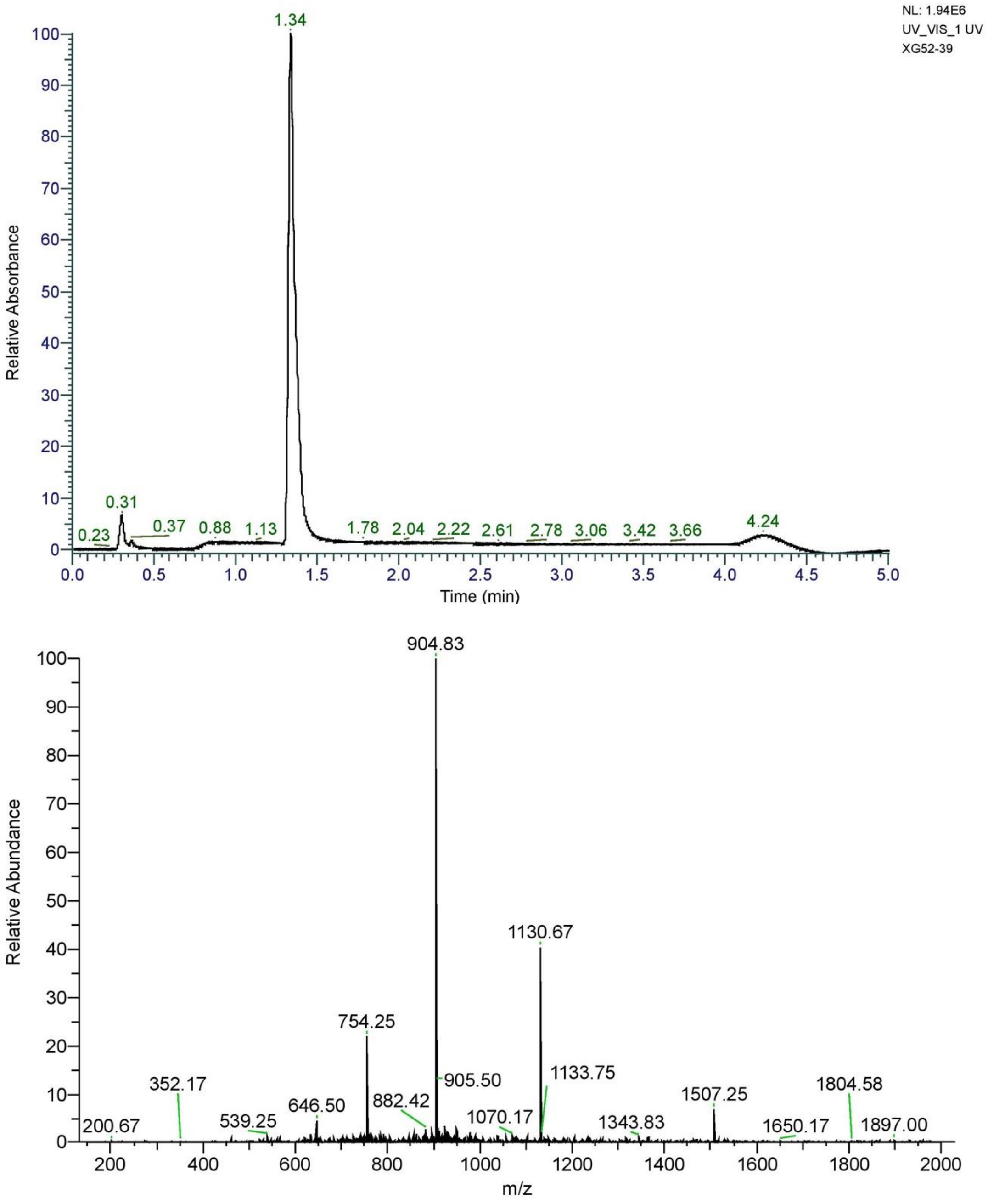

Figure S125. LCMS spectrum.

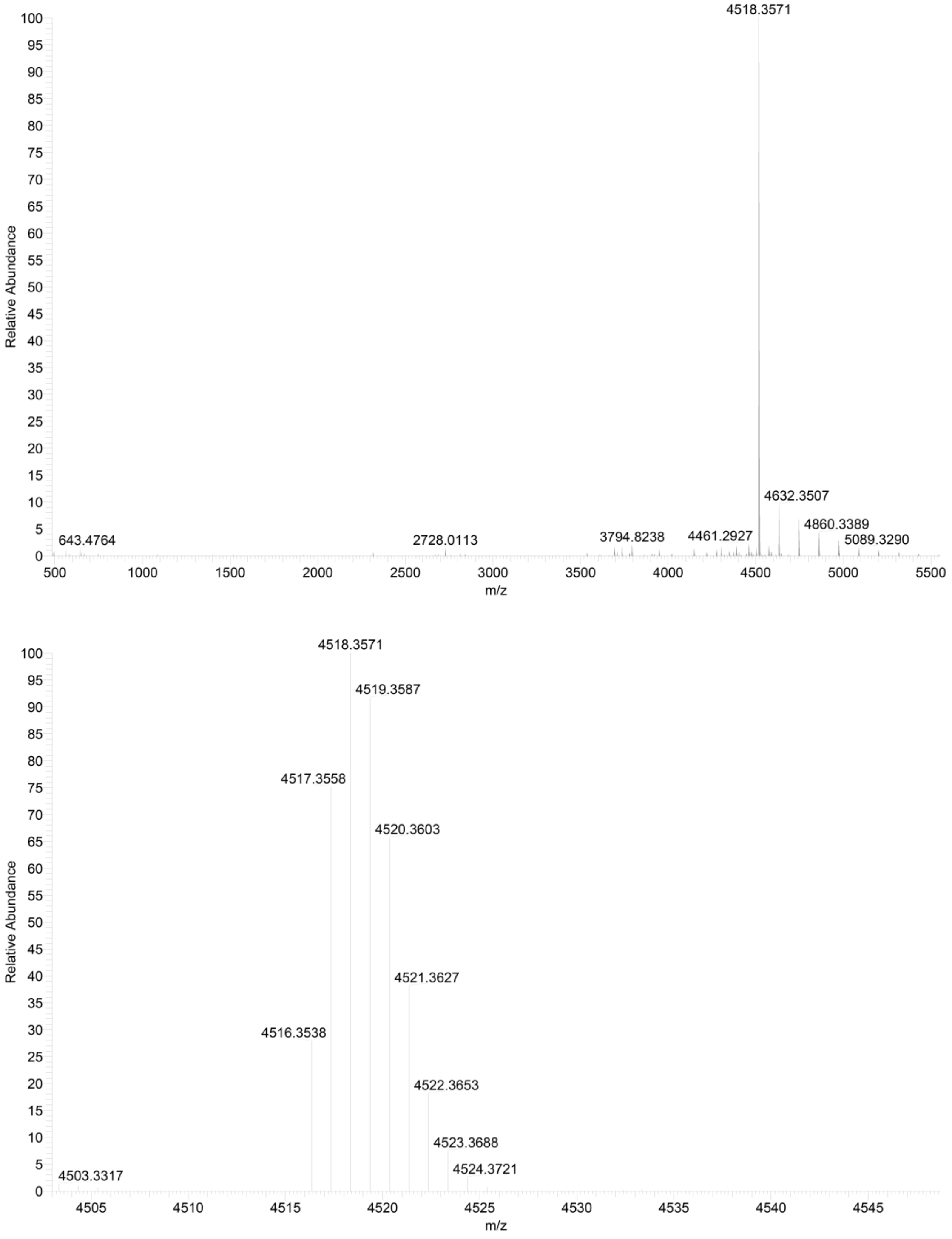

Figure S126. HRMS spectrum.

sr-**X46** ((*K*)$_8$(*K*LKL)$_4$(*K*LL)$_2$*K*KLL) was manually synthesized using TentaGel S RAM resin (393.4 mg, 0.09 mmol, 0.22 mmol·g^{-1}), the dendrimer was obtained as a white foamy solid after preparative RP-HPLC purification (185.6 mg, 35.4%). Analytical RP-HPLC: t_R = 1.38 min (100% A to 100% B in 3.5 min, λ= 214 nm). MS (ESI+): $C_{204}H_{397}N_{55}O_{34}$ calc./obs. 4162.10/4162.12 [M]$^+$.

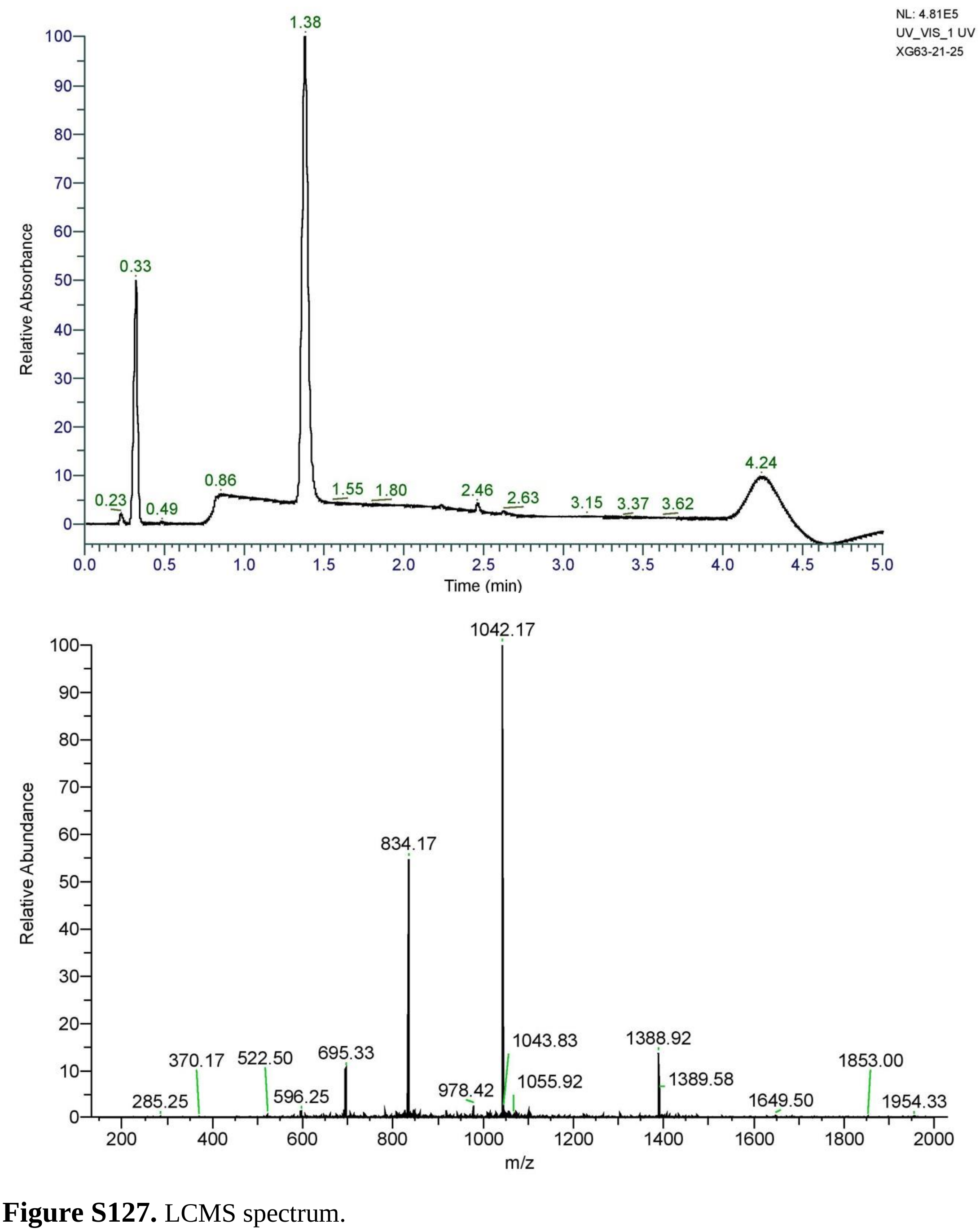

Figure S127. LCMS spectrum.

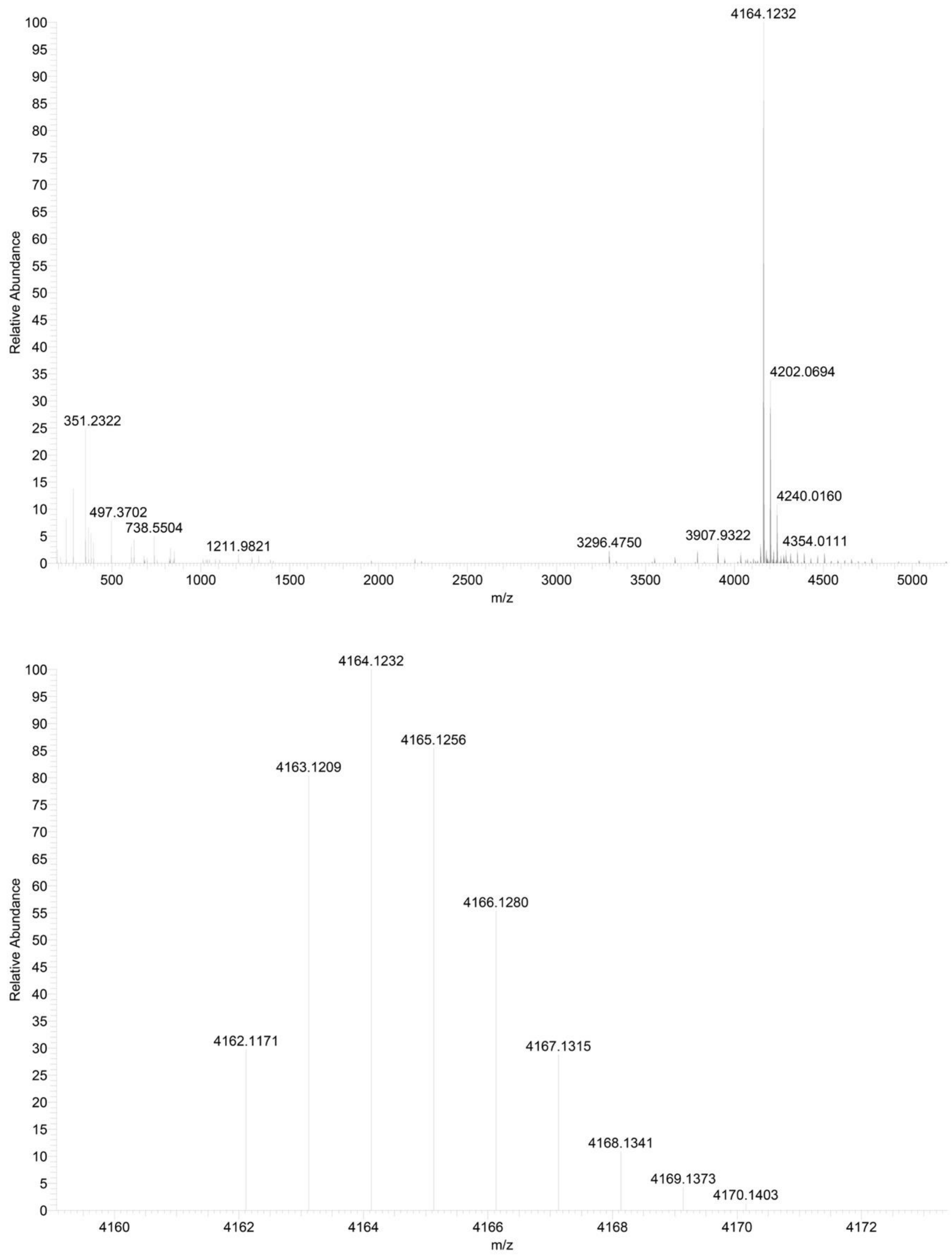

Figure S128. HRMS spectrum.

sr-**X47** ((*K*L)$_8$(*K*K)$_4$(*K*LLL)$_2$*K*LKK) was manually synthesized using TentaGel S RAM resin (393.4 mg, 0.09 mmol, 0.22 mmol·g^{-1}), the dendrimer was obtained as a white foamy solid after preparative RP-HPLC purification (115.5 mg, 20.9%). Analytical RP-HPLC: t_R = 1.35 min (100% A to 100% B in 3.5 min, λ= 214 nm). MS (ESI+): $C_{216}H_{420}N_{58}O_{36}$ calc./obs. 4403.28/4403.28 $[M]^+$.

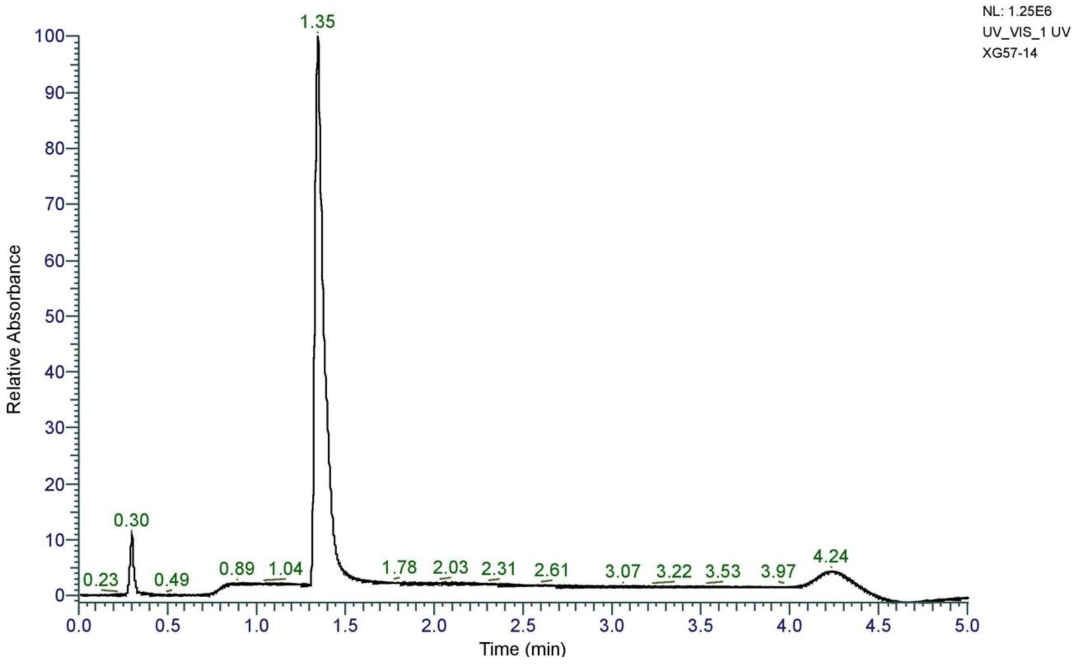

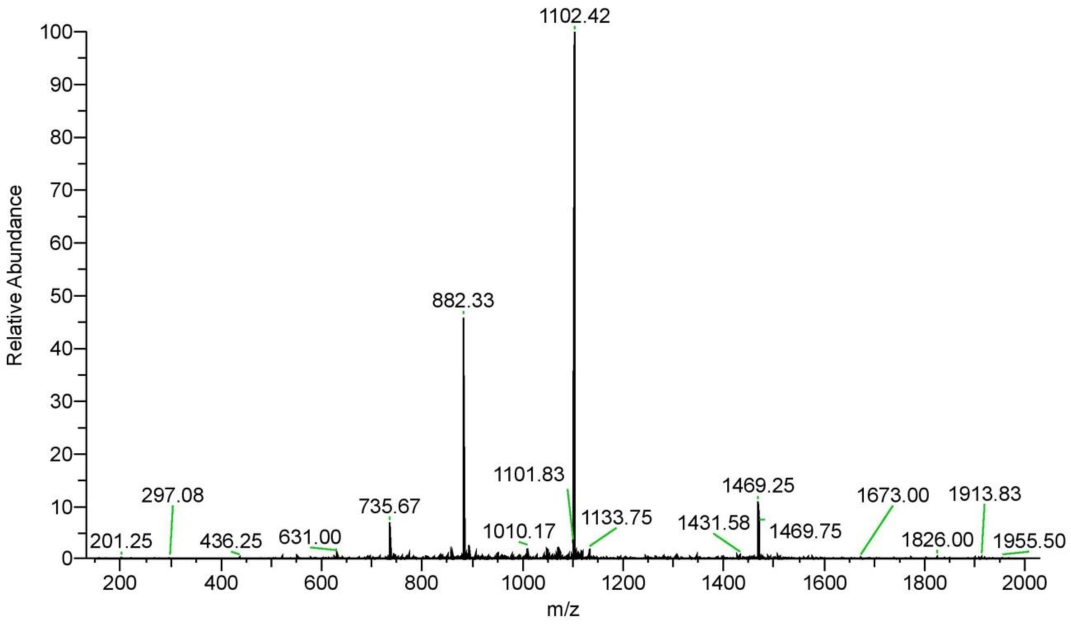

Figure S129. LCMS spectrum.

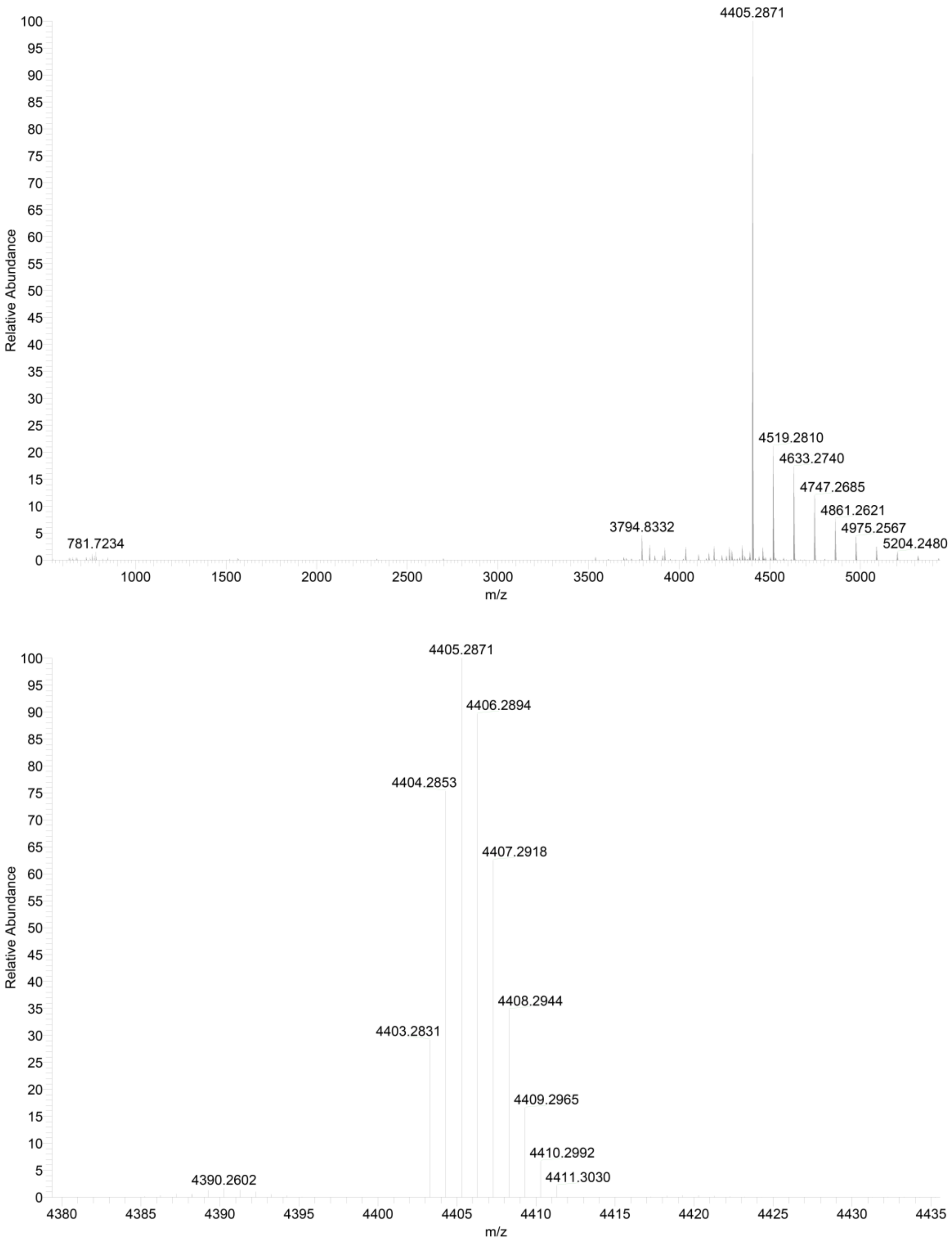

Figure S130. HRMS spectrum.

sr-**X48** ((*K*L)$_8$(*K*KL)$_4$(*K*LK)$_2$*K*LKL) was manually synthesized using TentaGel S RAM resin (393.4 mg, 0.09 mmol, 0.22 mmol·g^{-1}), the dendrimer was obtained as a white foamy solid after preparative RP-HPLC purification (69.0 mg, 11.9%). Analytical RP-HPLC: t_R = 1.37 min (100% A to 100% B in 3.5 min, λ= 214 nm). MS (ESI+): $C_{228}H_{443}N_{61}O_{38}$ calc./obs. 4644.46/4644.46 [M]$^+$.

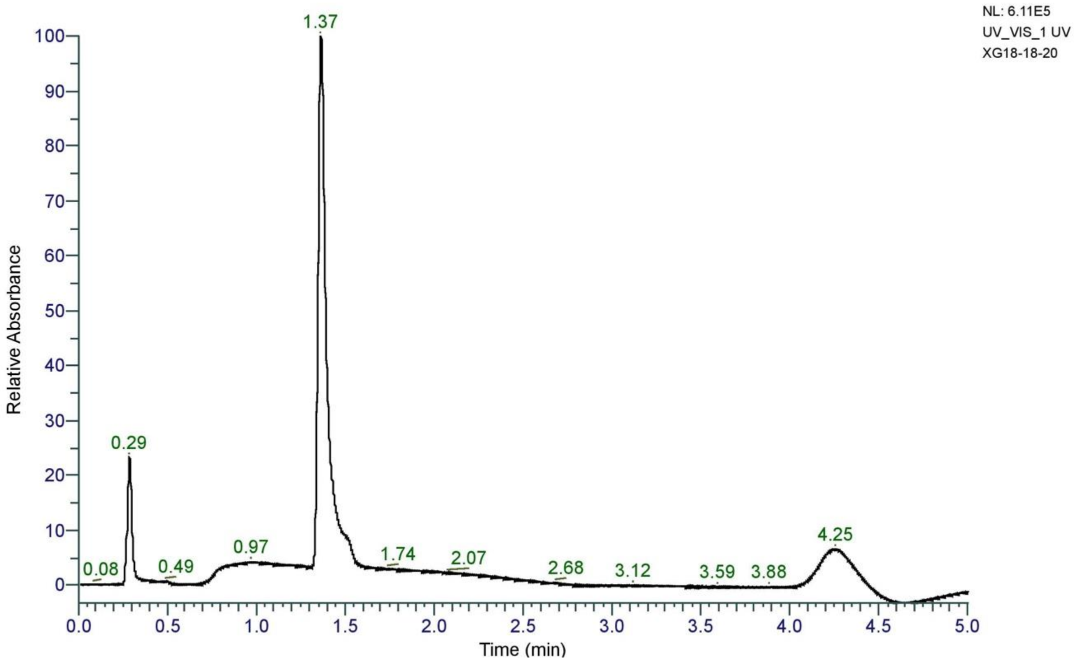

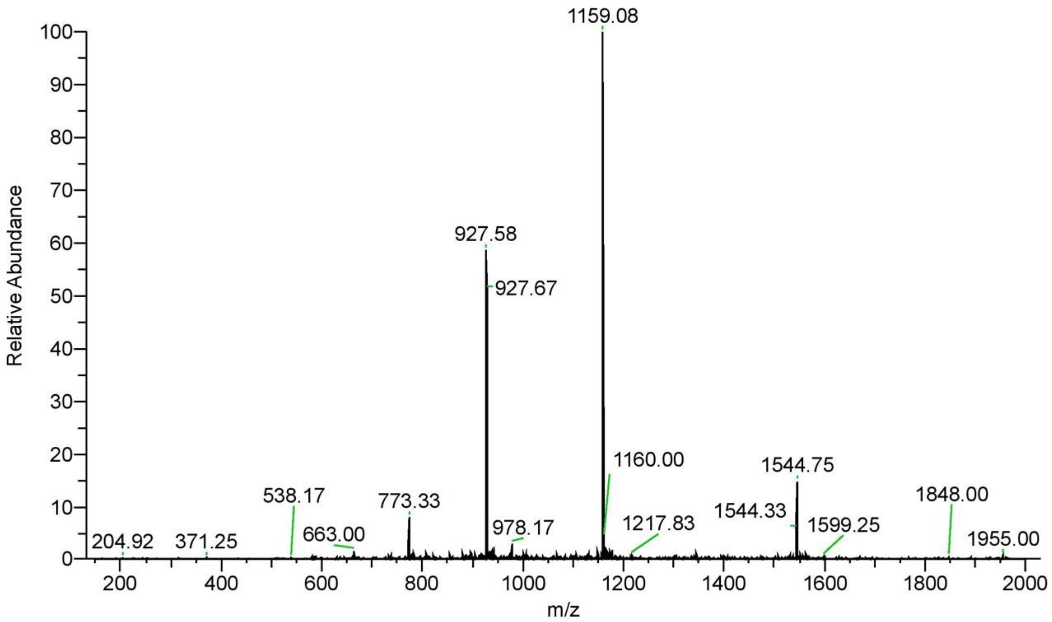

Figure S131. LCMS spectrum.

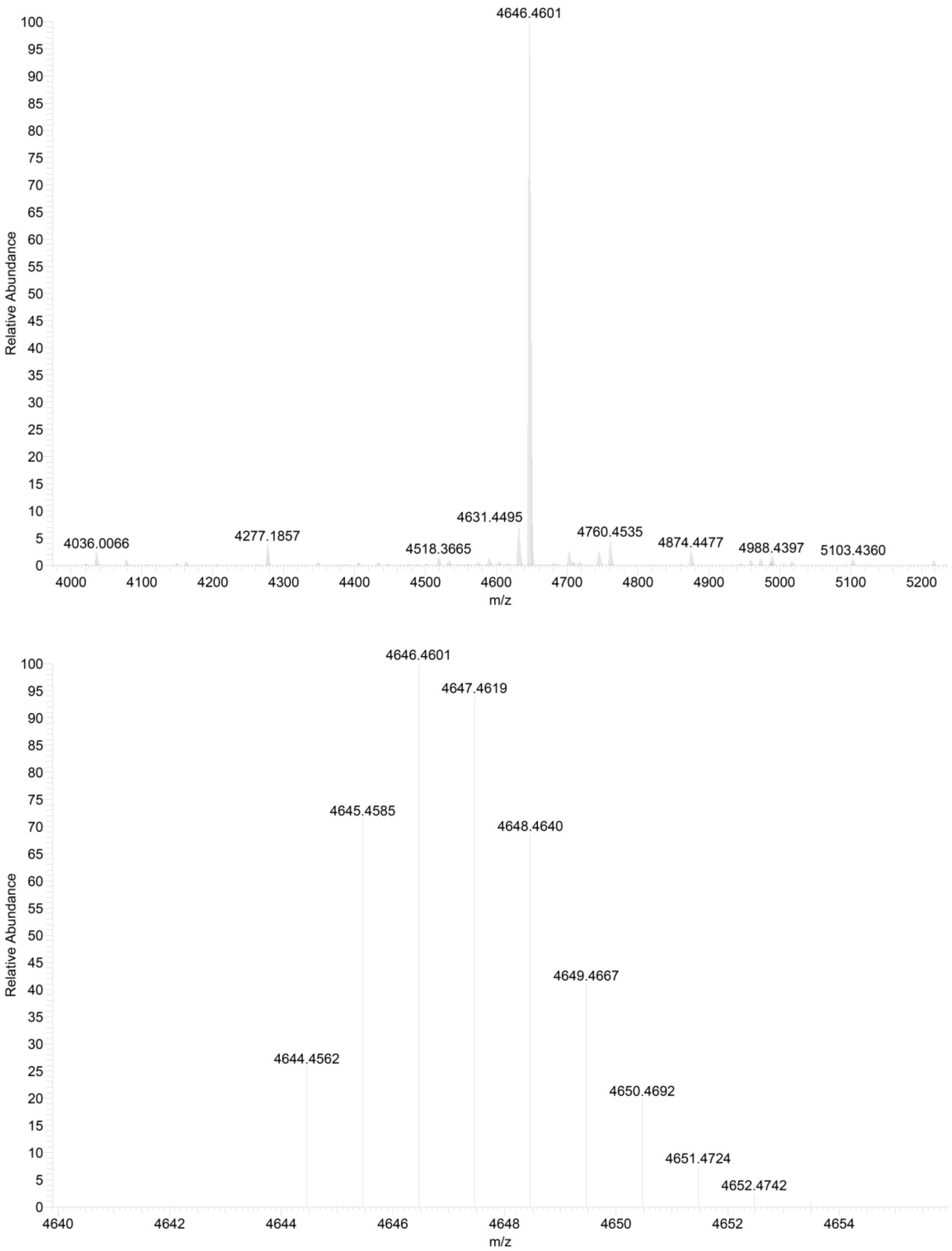

Figure S132. HRMS spectrum.

sr-**X49** ((*K*L)$_8$(*K*KL)$_4$(*K*KL)$_2$*K*LLK) was manually synthesized using TentaGel S RAM resin (393.4 mg, 0.09 mmol, 0.22 mmol·g^{-1}), the dendrimer was obtained as a white foamy solid after preparative RP-HPLC purification (129.1 mg, 22.2%). Analytical RP-HPLC: t_R = 1.35 min (100% A to 100% B in 3.5 min, λ= 214 nm MS (ESI+): $C_{228}H_{443}N_{61}O_{38}$ calc./obs. 4644.46/4644.46 [M]$^+$.

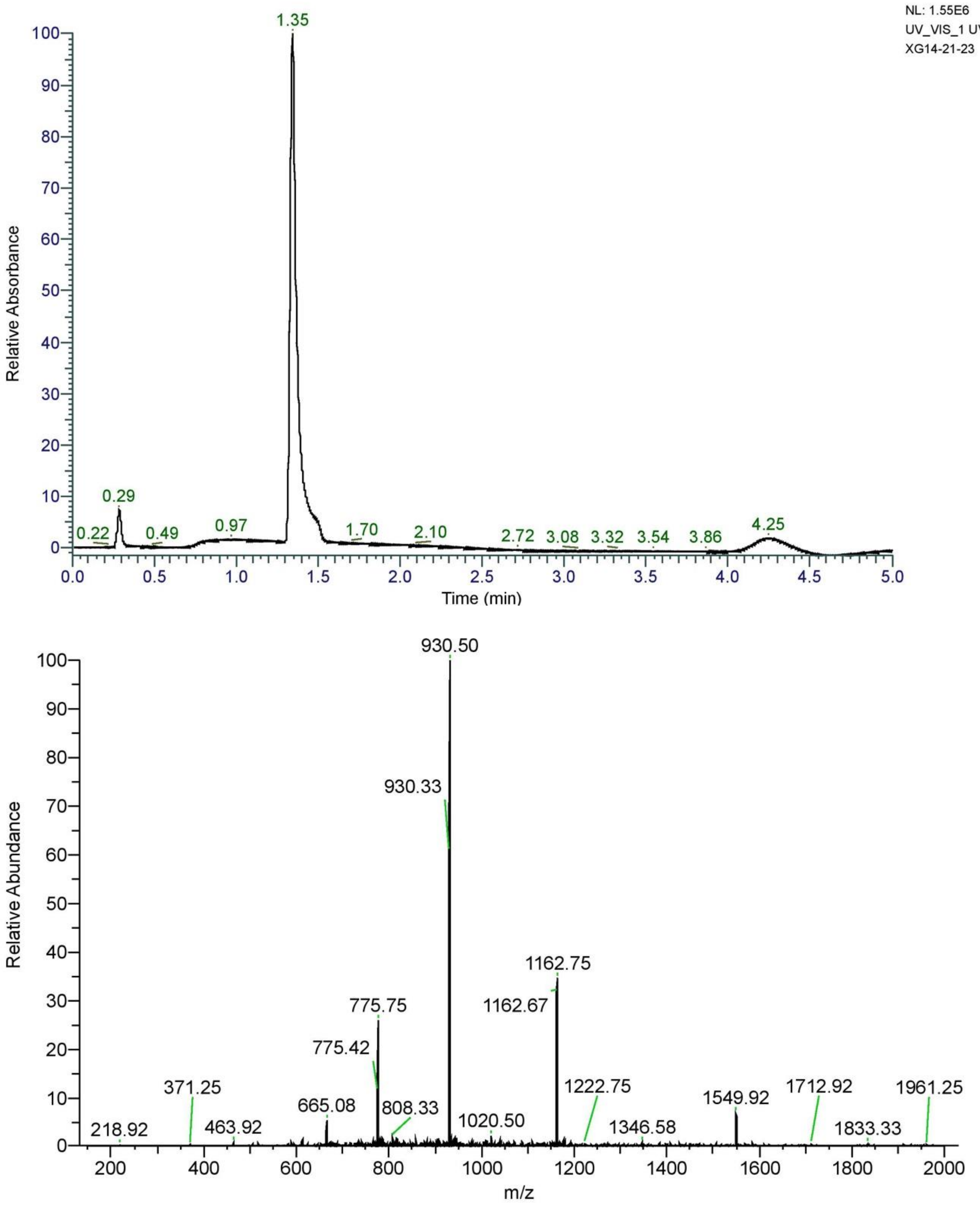

Figure S133. LCMS spectrum.

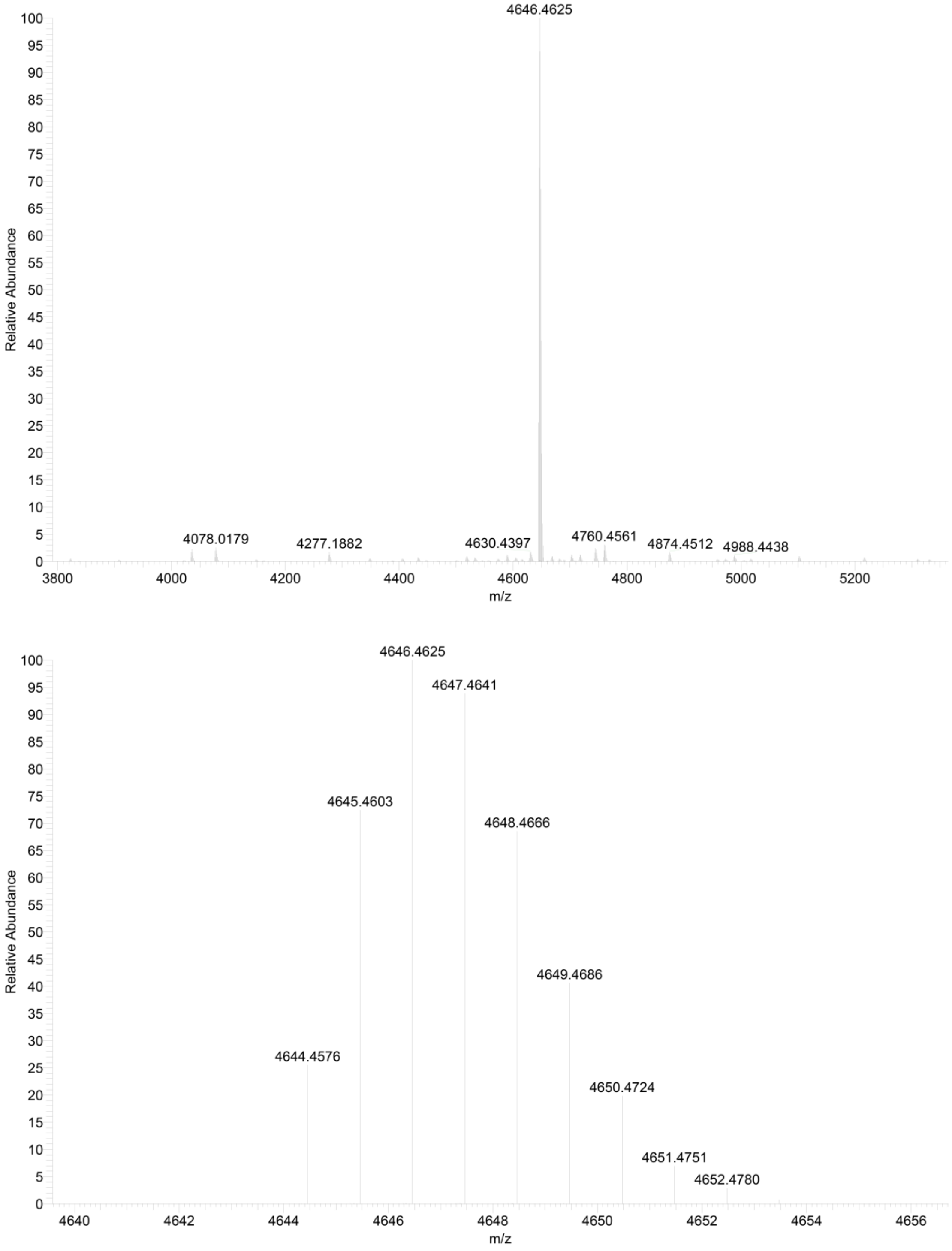

Figure S134. HRMS spectrum.

sr-**X50** ((*K*L)$_8$(*K*KL)$_4$(*K*KL)$_2$*K*LKL) was manually synthesized using TentaGel S RAM resin (393.4 mg, 0.09 mmol, 0.22 mmol·g^{-1}), the dendrimer was obtained as a white foamy solid after preparative RP-HPLC purification (86.1 mg, 14.8%). Analytical RP-HPLC: t_R = 1.34 min (100% A to 100% B in 3.5 min, λ= 214 nm). MS (ESI+): $C_{228}H_{443}N_{61}O_{38}$ calc./obs. 4644.46/4644.46 $[M]^+$.

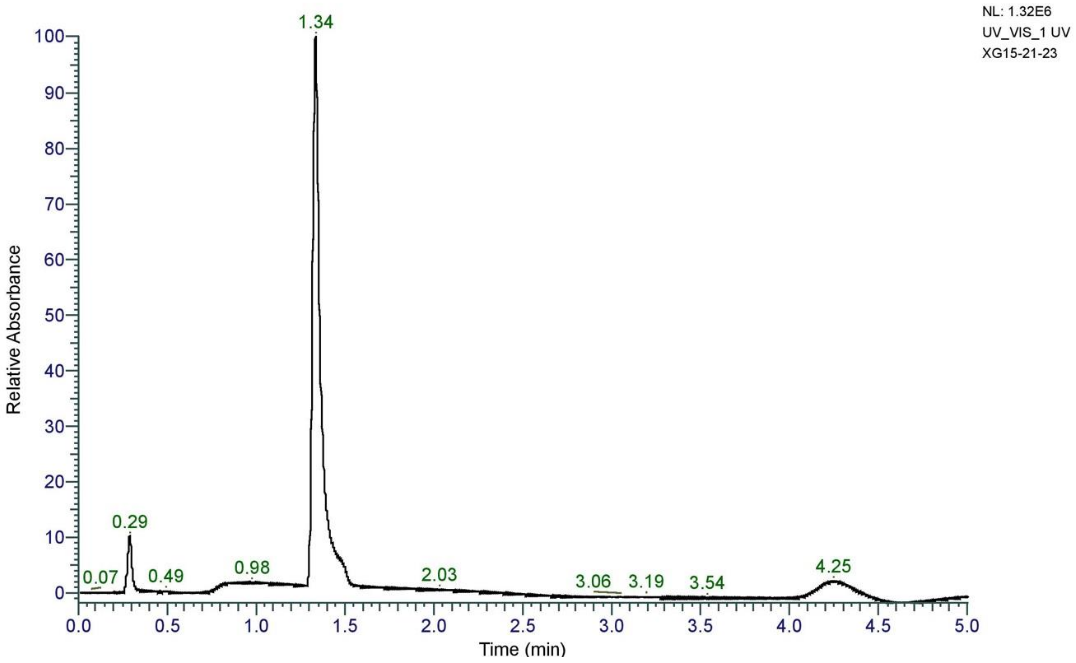

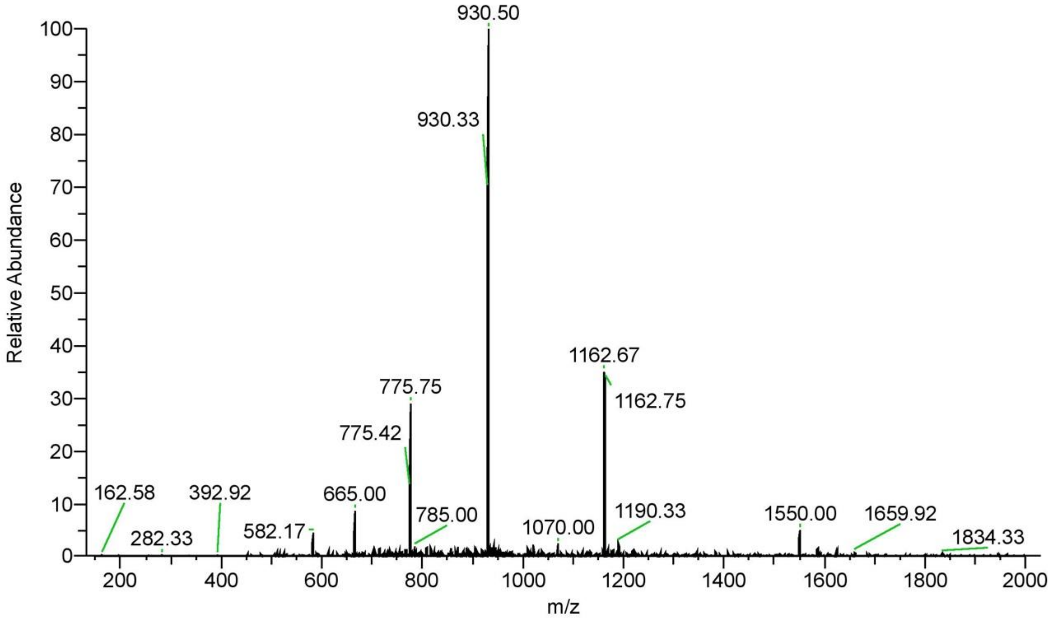

Figure S135. LCMS spectrum.

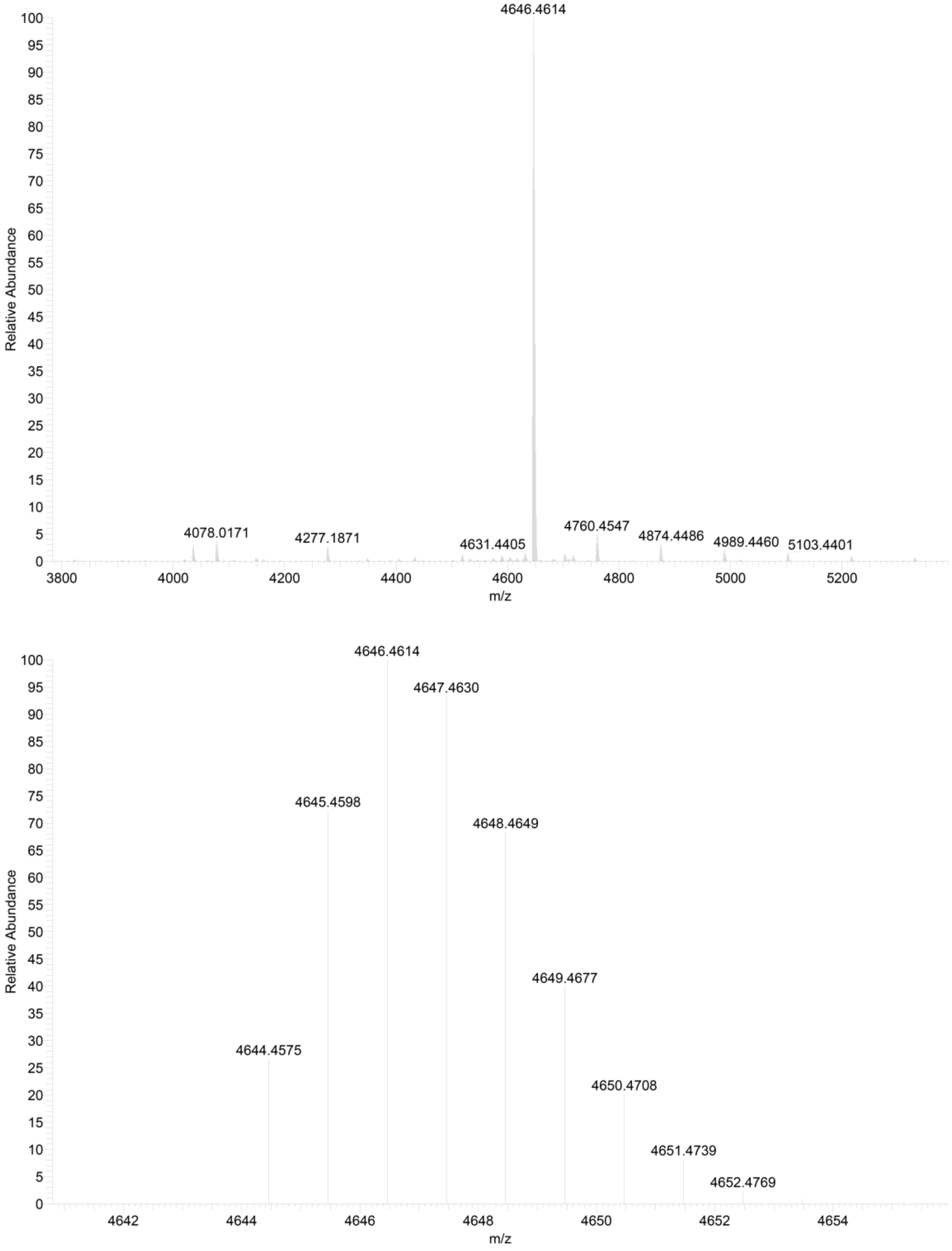

Figure S136. HRMS spectrum.

sr-**X51** ((*K*L)$_8$(*K*KL)$_4$(*K*LK)$_2$*K*LLK) was manually synthesized using TentaGel S RAM resin (393.4 mg, 0.09 mmol, 0.22 mmol·g^{-1}), the dendrimer was obtained as a white foamy solid after preparative RP-HPLC purification (98.2 mg, 16.9%). Analytical RP-HPLC: t_R = 1.32 min (100% A to 100% B in 3.5 min, λ= 214 nm). MS (ESI+): $C_{228}H_{443}N_{61}O_{38}$ calc./obs. 4644.46/4644.46 [M]$^+$.

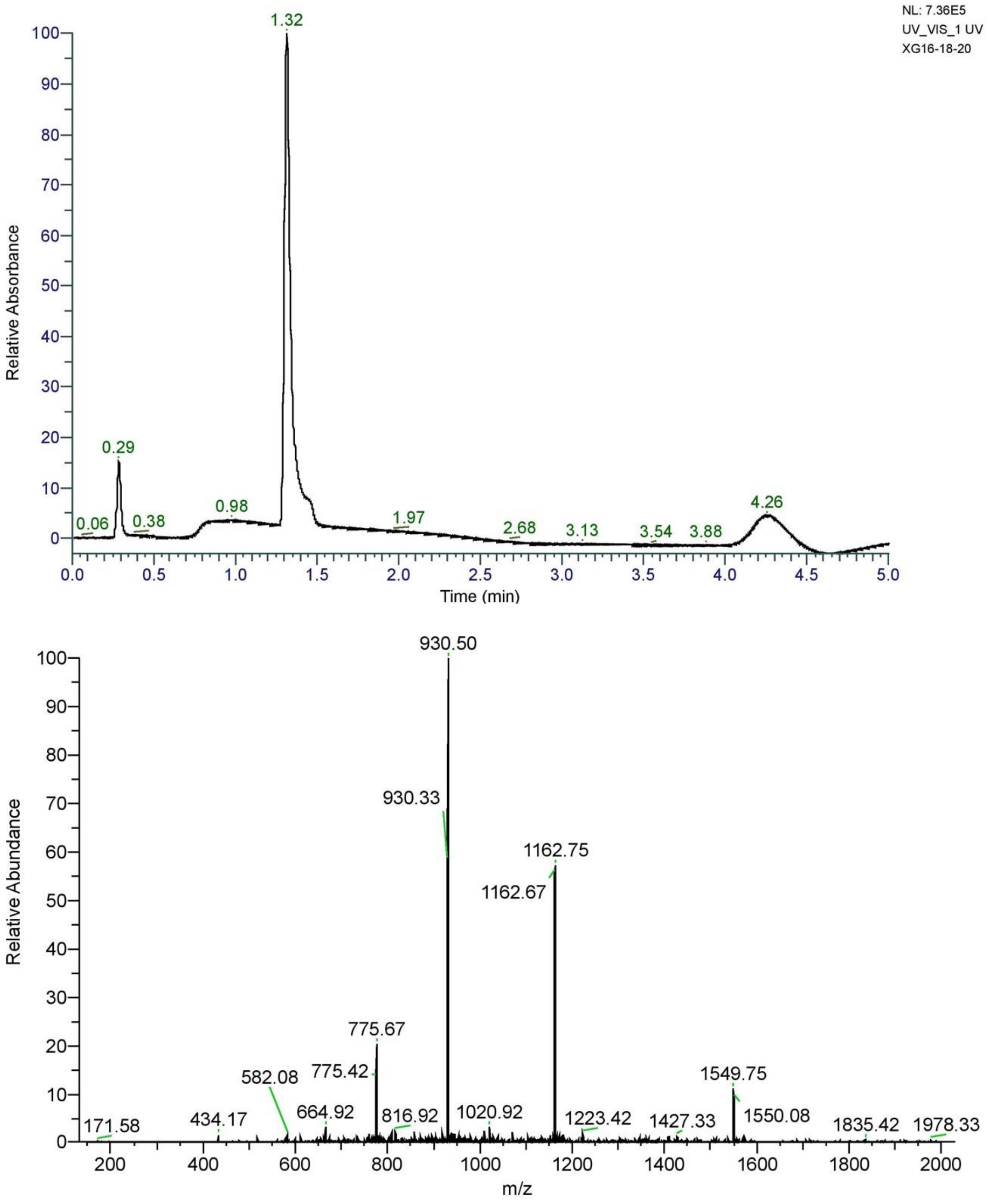

Figure S137. LCMS spectrum.

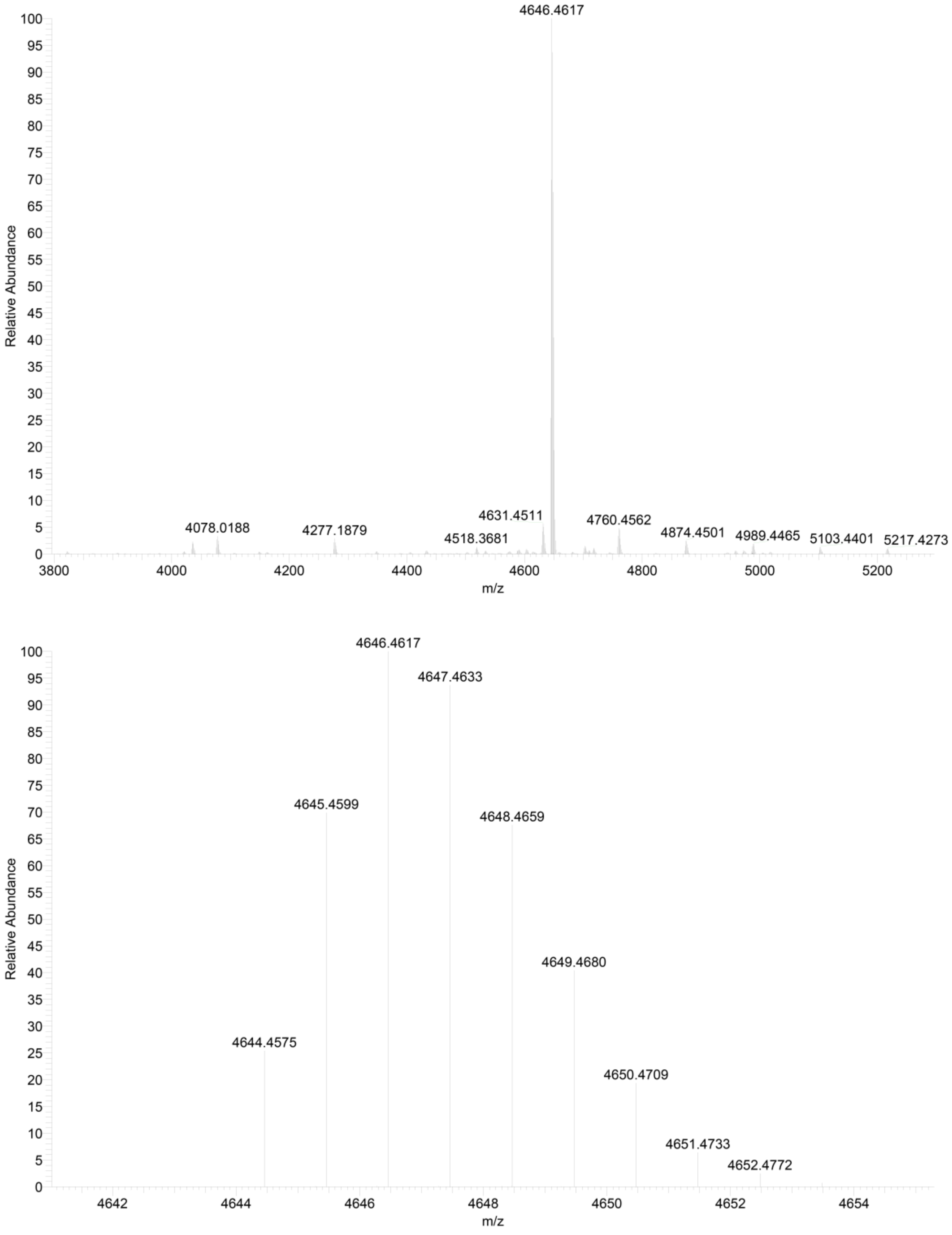

Figure S138. HRMS spectrum.

sr-**X52** ((*K*L)$_8$(*K*KL)$_4$(*K*KL)$_2$*K*KLL) was manually synthesized using TentaGel S RAM resin (393.4 mg, 0.09 mmol, 0.22 mmol·g^{-1}), the dendrimer was obtained as a white foamy solid after preparative RP-HPLC purification (122.0 mg, 21.0%). Analytical RP-HPLC: t_R = 1.35 min (100% A to 100% B in 3.5 min, λ= 214 nm). MS (ESI+): $C_{228}H_{443}N_{61}O_{38}$ calc./obs. 4644.46/4644.48 [M]$^+$.

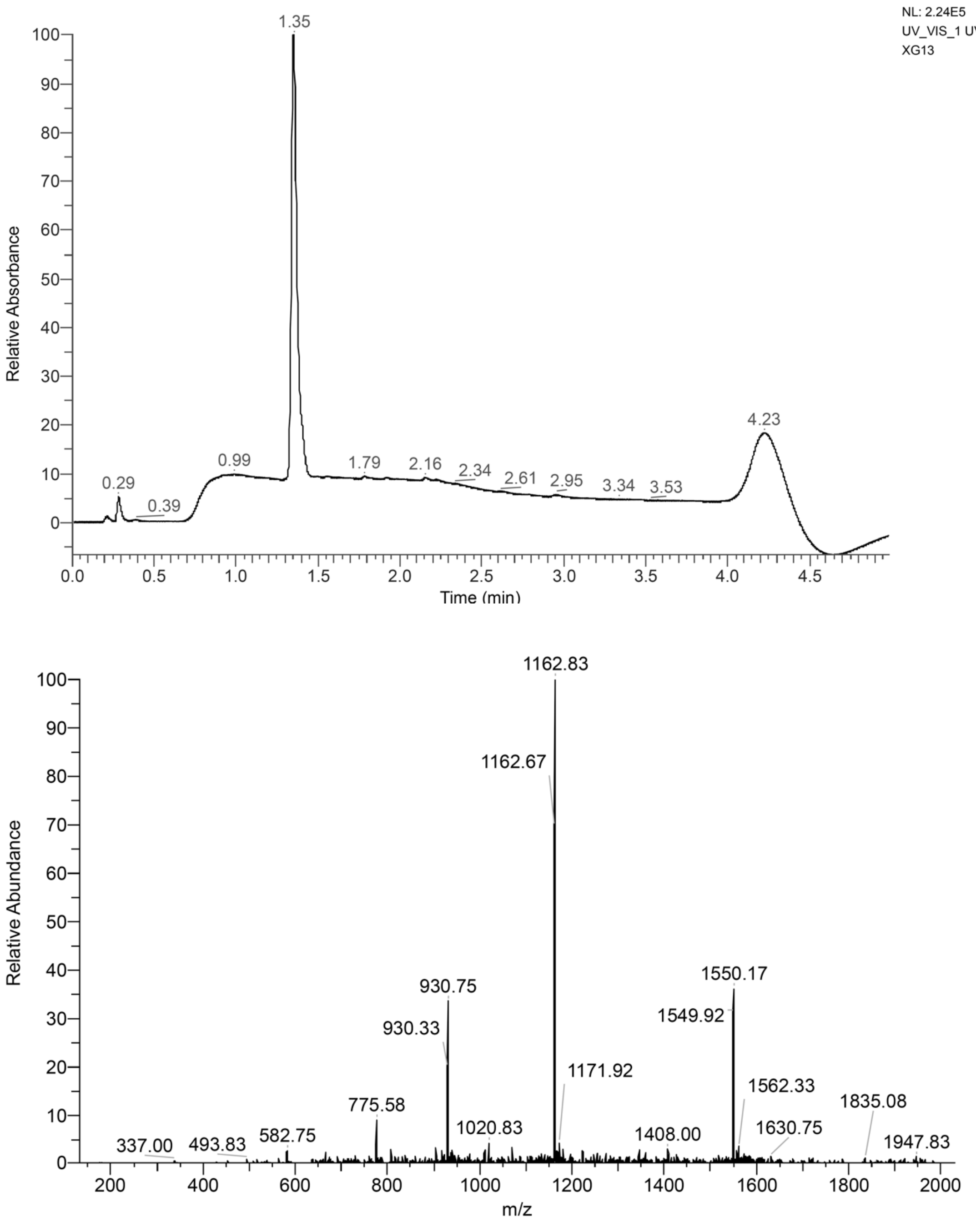

Figure S139. LCMS spectrum.

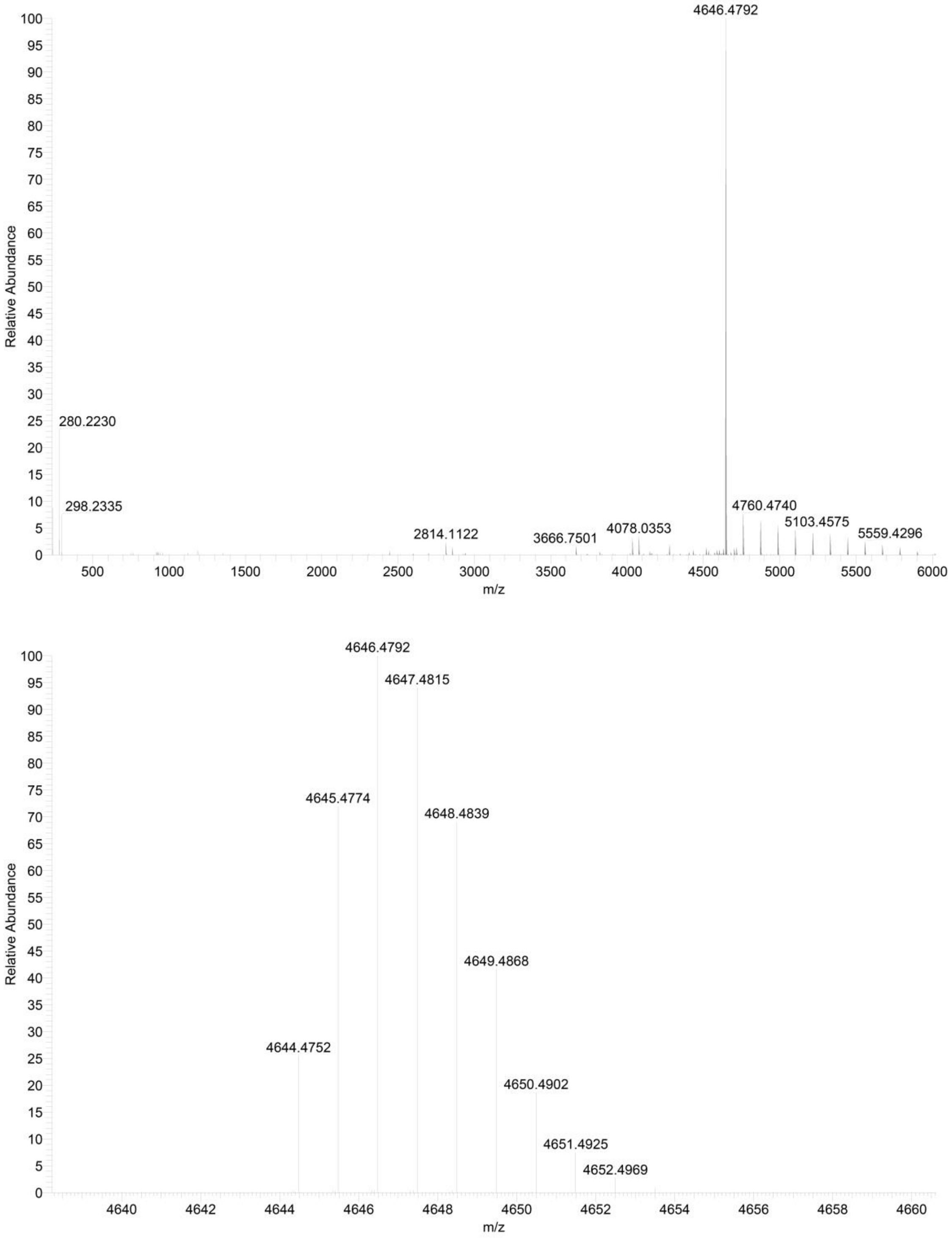

Figure S140. HRMS spectrum.

sr-**X53** ((LK)$_8$(*K*LK)$_4$(*K*KL)$_2$*K*LLK) was manually synthesized using TentaGel S RAM resin (393.4 mg, 0.09 mmol, 0.22 mmol·g^{-1}), the dendrimer was obtained as a white foamy solid after preparative RP-HPLC purification (181.0 mg, 31.1%). Analytical RP-HPLC: t_R = 1.28 min (100% A to 100% B in 3.5 min, λ= 214 nm). MS (ESI+): $C_{228}H_{443}N_{61}O_{38}$ calc./obs. 4644.46/4644.48 $[M]^+$.

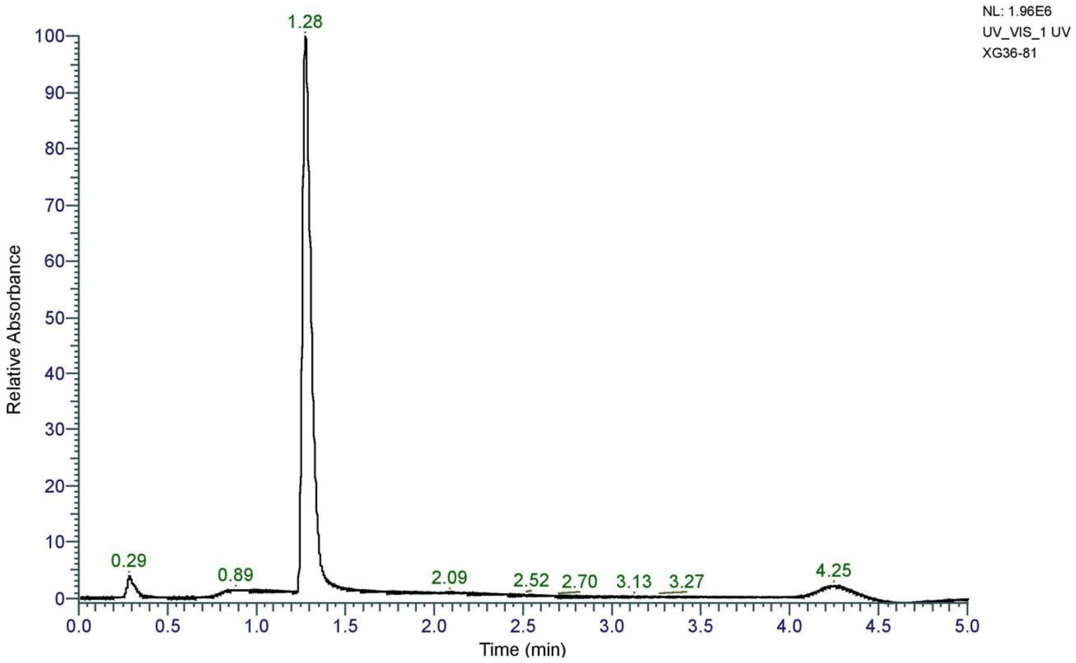

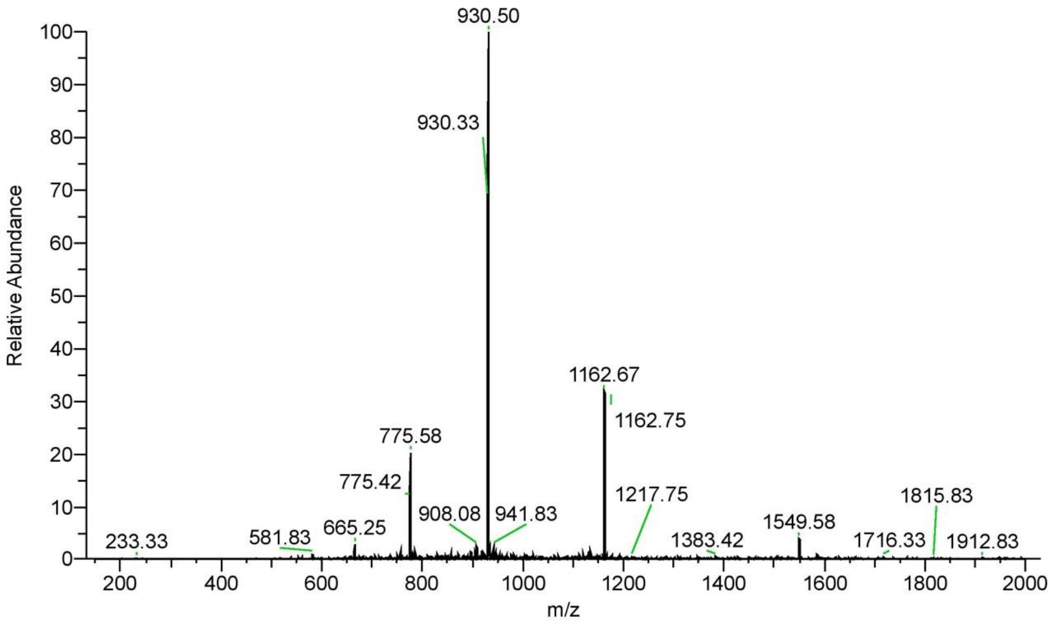

Figure S141. LCMS spectrum.

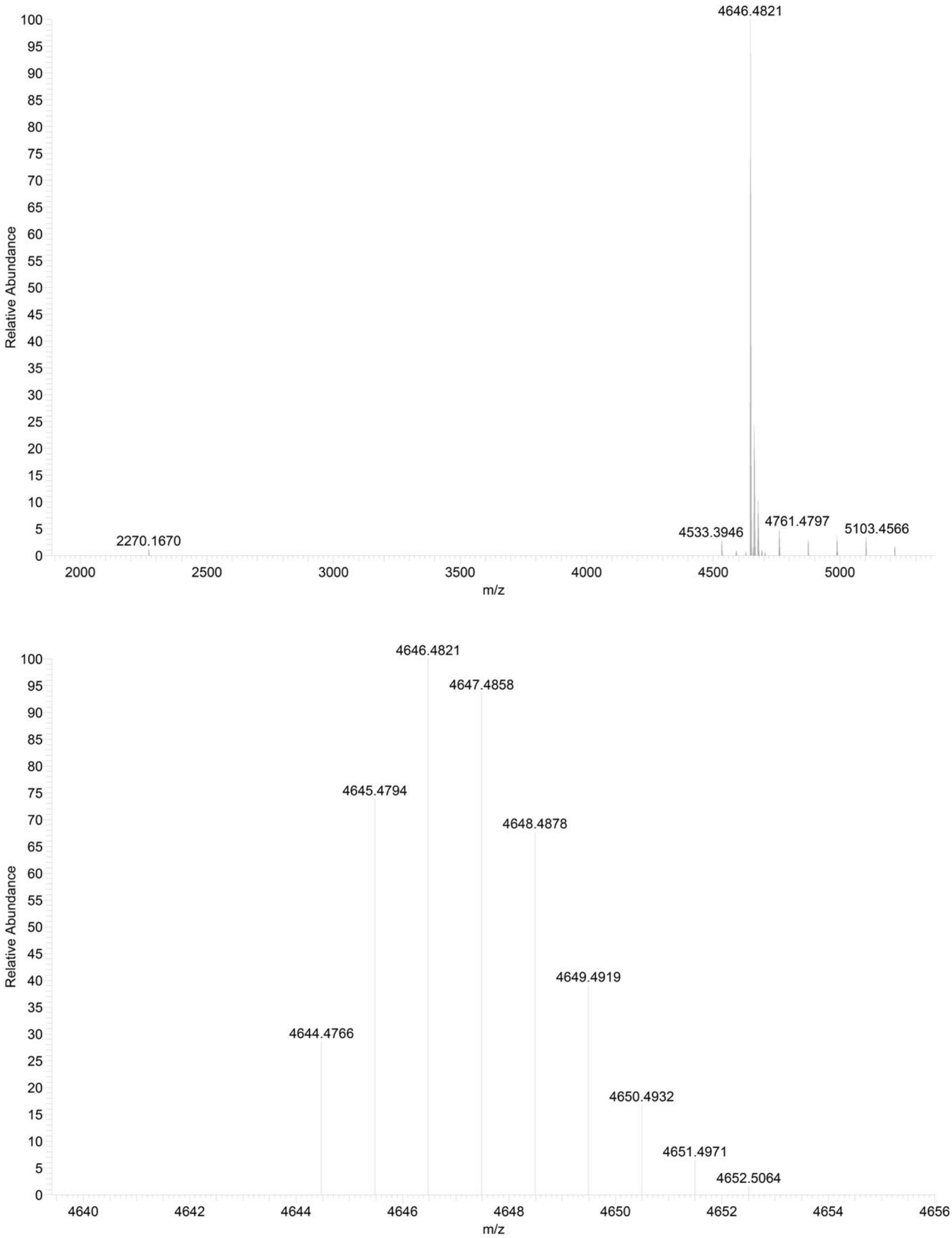

Figure S142. HRMS spectrum.

sr-**X54** ((KL)$_8$(KKL)$_4$(KKL)$_2$$K$KL) was synthesized by CEM Liberty Blue synthesizer using Rink Amide MBHA resin (320.0 mg, 0.08 mmol, 0.25 mmol·g^{-1}), the dendrimer was obtained as a white foamy solid after preparative RP-HPLC purification (78.8 mg, 13.8%). Analytical RP-HPLC: t_R = 1.33 min (100% A to 100% B in 3.5 min, λ= 214 nm). MS (ESI+): $C_{222}H_{432}N_{60}O_{37}$ calc./obs. 4531.38/4531.39 [M]$^+$.

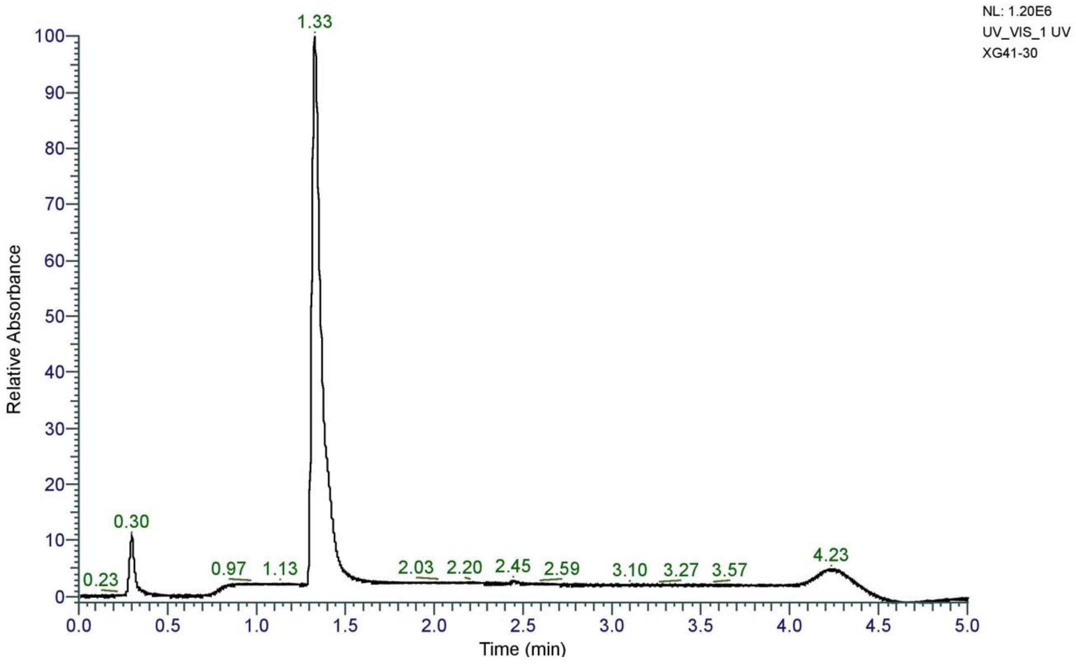

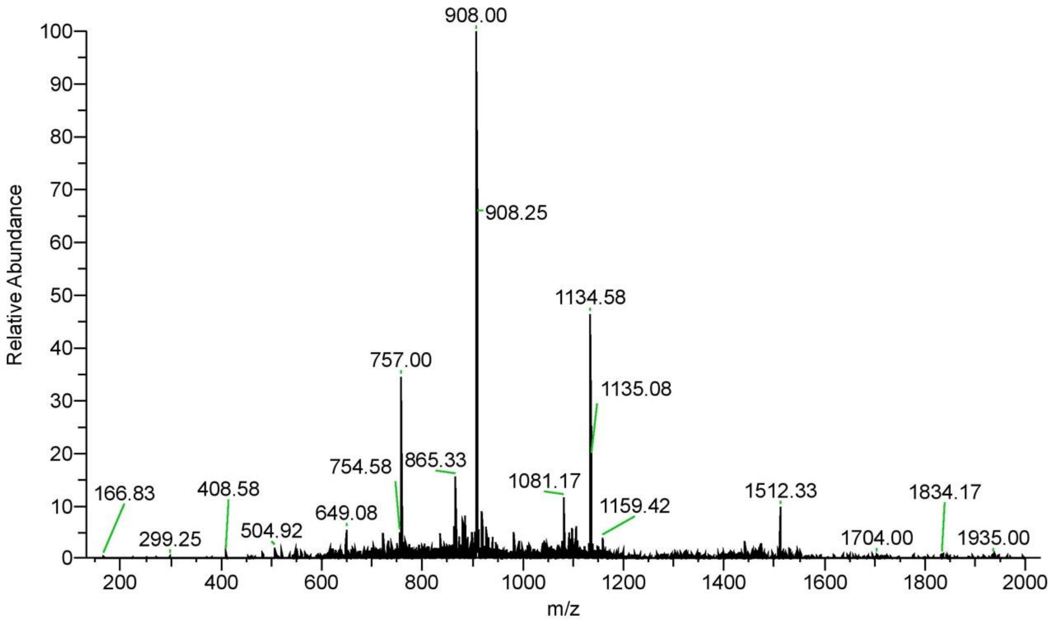

Figure S143. LCMS spectrum.

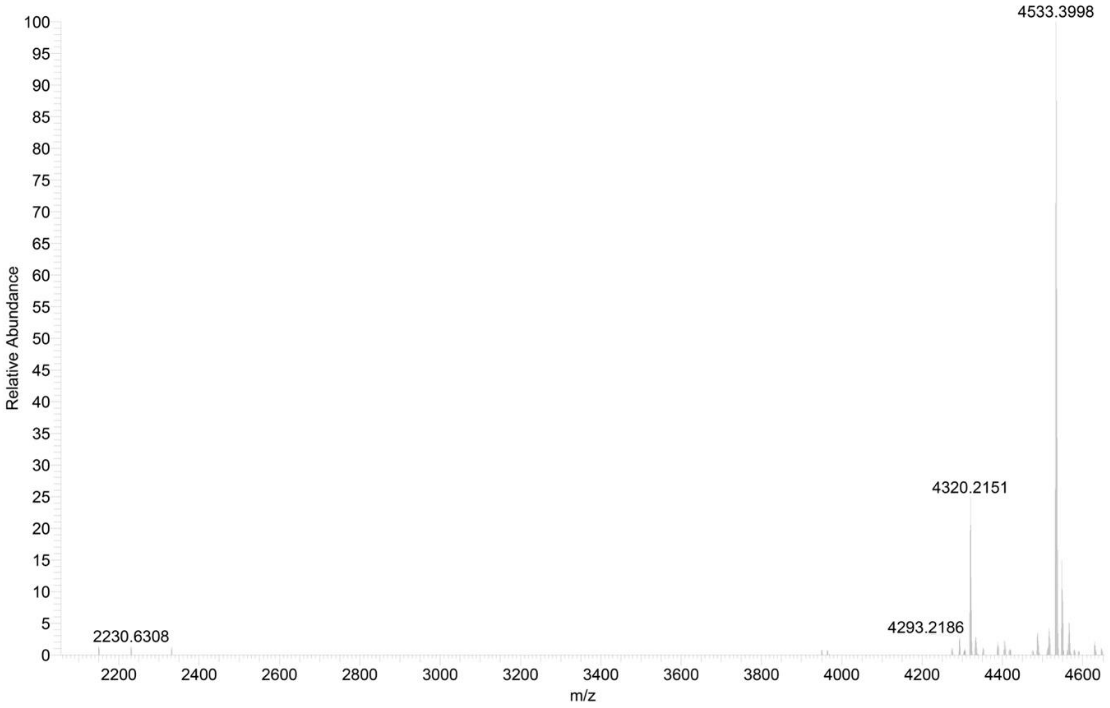

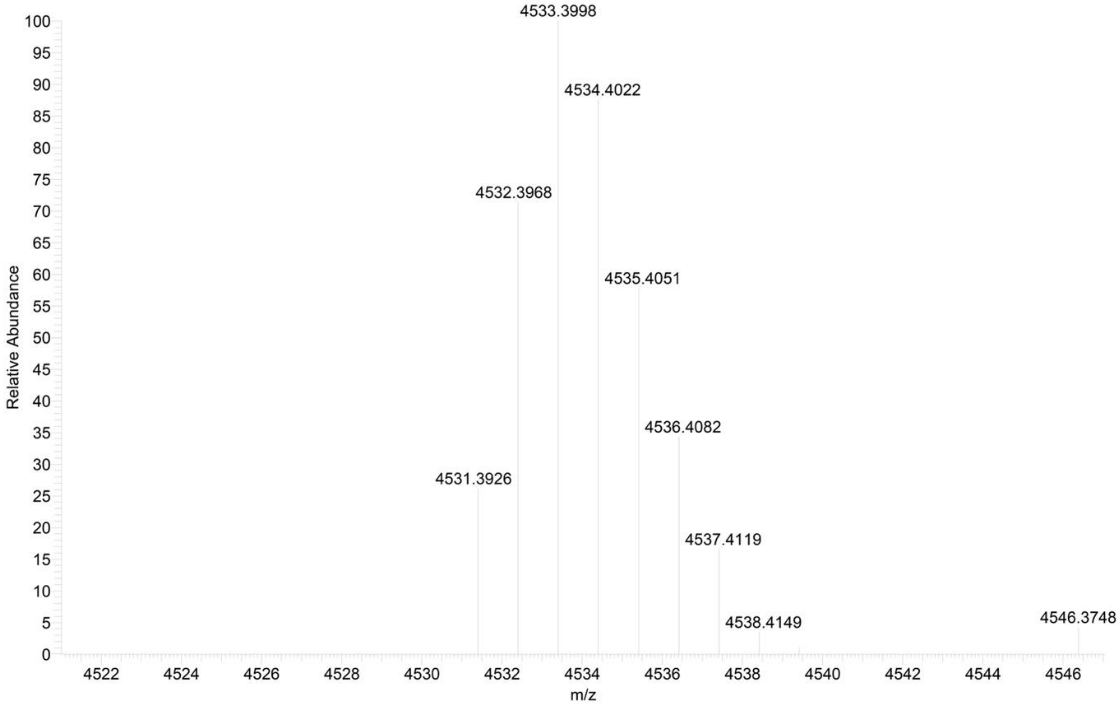

Figure S144. HRMS spectrum.

sr-**X55** ((LK)$_8$(*K*KK)$_4$(*K*LL)$_2$*K*LLL) was manually synthesized using TentaGel S RAM resin (393.4 mg, 0.09 mmol, 0.22 mmol·g^{-1}), the dendrimer was obtained as a white foamy solid after preparative RP-HPLC purification (156.7 mg, 26.5%). Analytical RP-HPLC: t_R = 1.31 min (100% A to 100% B in 3.5 min, λ= 214 nm). MS (ESI+): $C_{228}H_{444}N_{62}O_{38}$ calc./obs. 4659.47/4659.47 $[M]^+$.

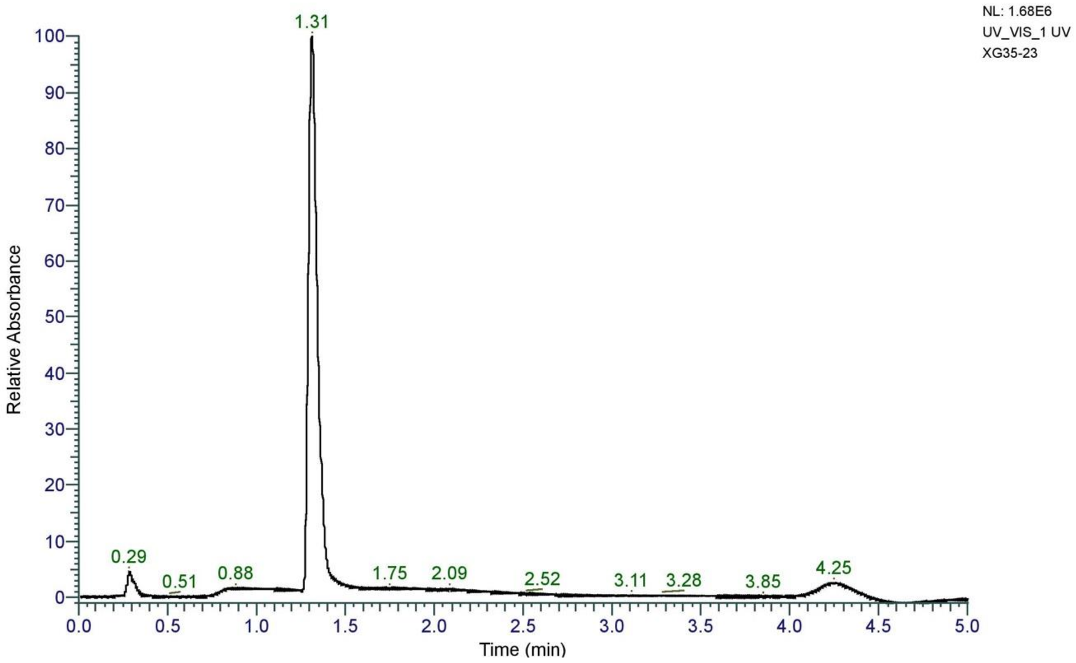

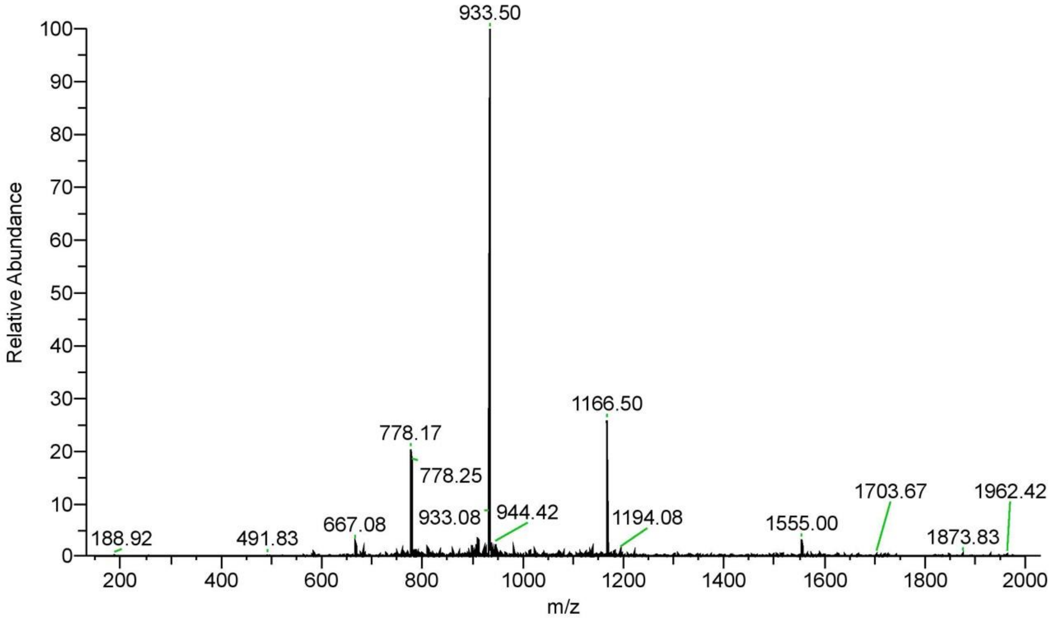

Figure S145. LCMS spectrum.

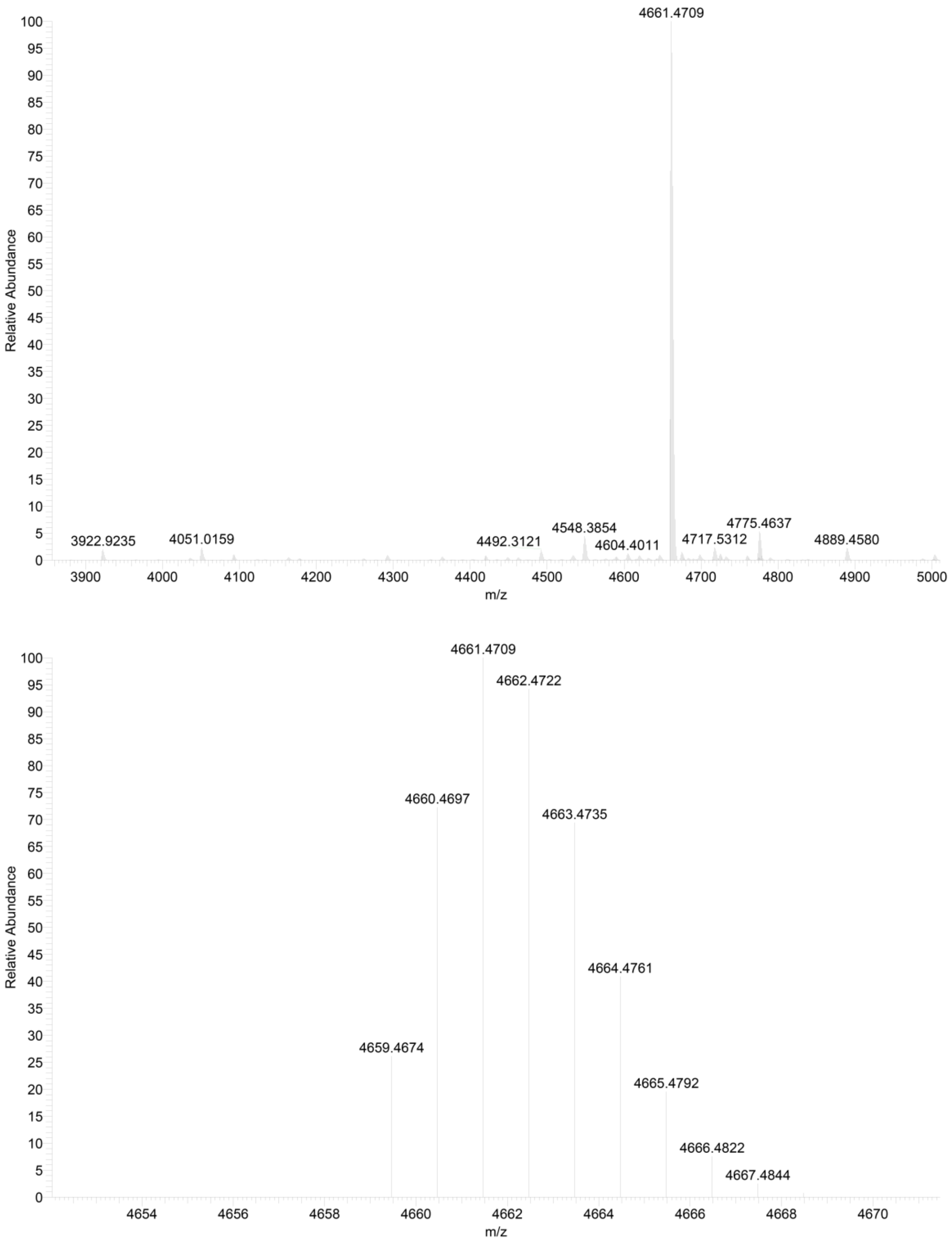

Figure S146. HRMS spectrum.

sr-**X56** ((*K*L)$_8$(*K*LK)$_4$(*K*KK)$_2$*K*LLL) was manually synthesized using TentaGel S RAM resin (393.4 mg, 0.09 mmol, 0.22 mmol·g^{-1}), the dendrimer was obtained as a white foamy solid after preparative RP-HPLC purification (109.0 mg, 18.4%). Analytical RP-HPLC: t_R = 1.33 min (100% A to 100% B in 3.5 min, λ= 214 nm). MS (ESI+): $C_{228}H_{444}N_{62}O_{38}$ calc./obs. 4659.47/4659.46 [M]$^+$.

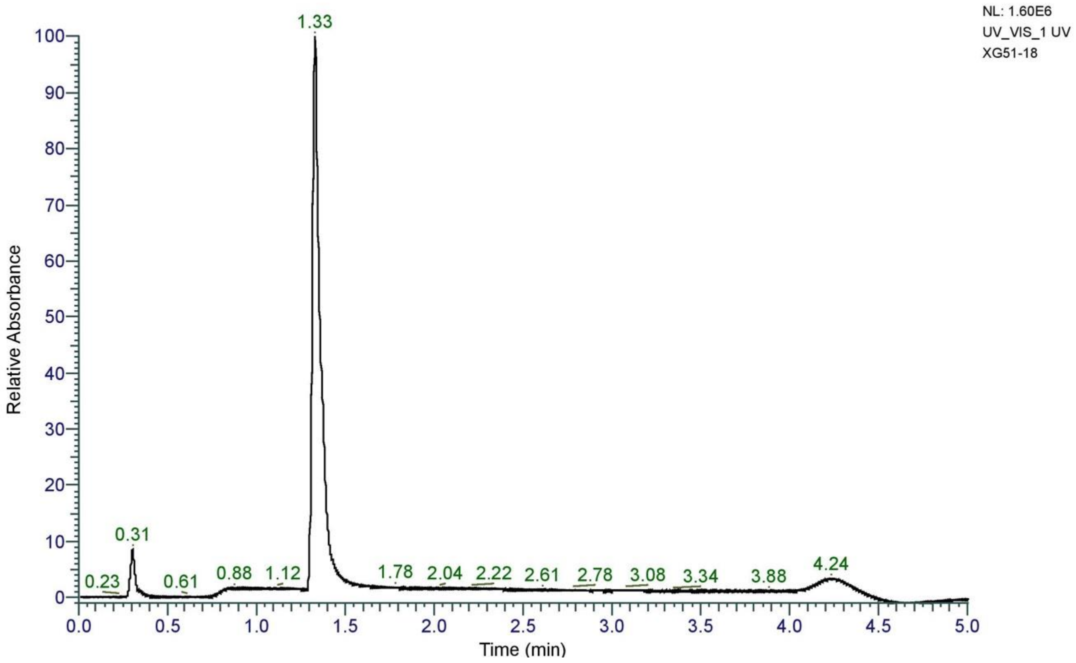

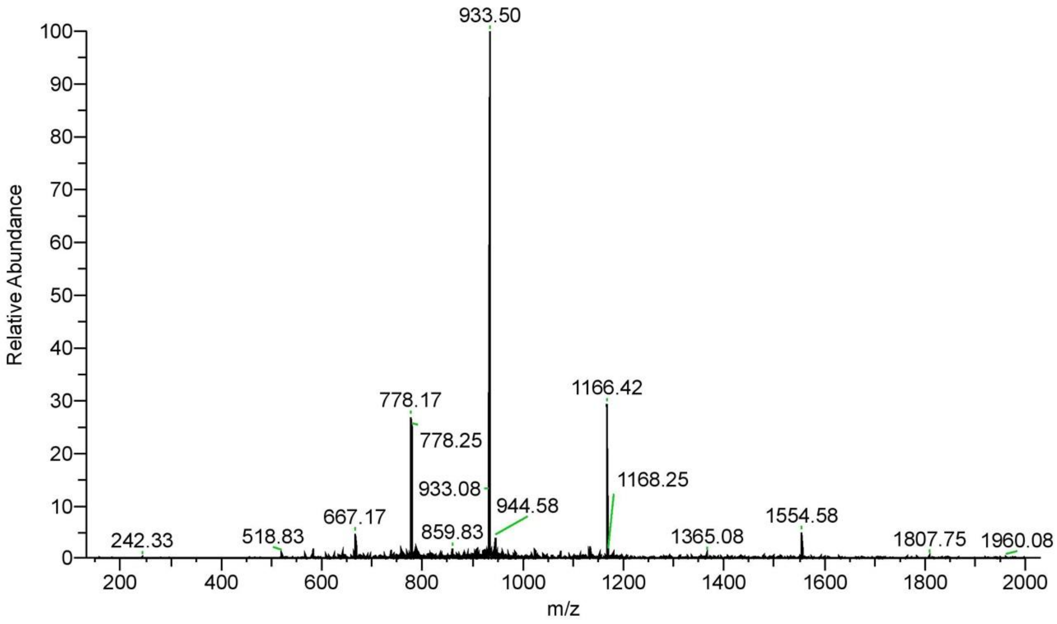

Figure S147. LCMS spectrum.

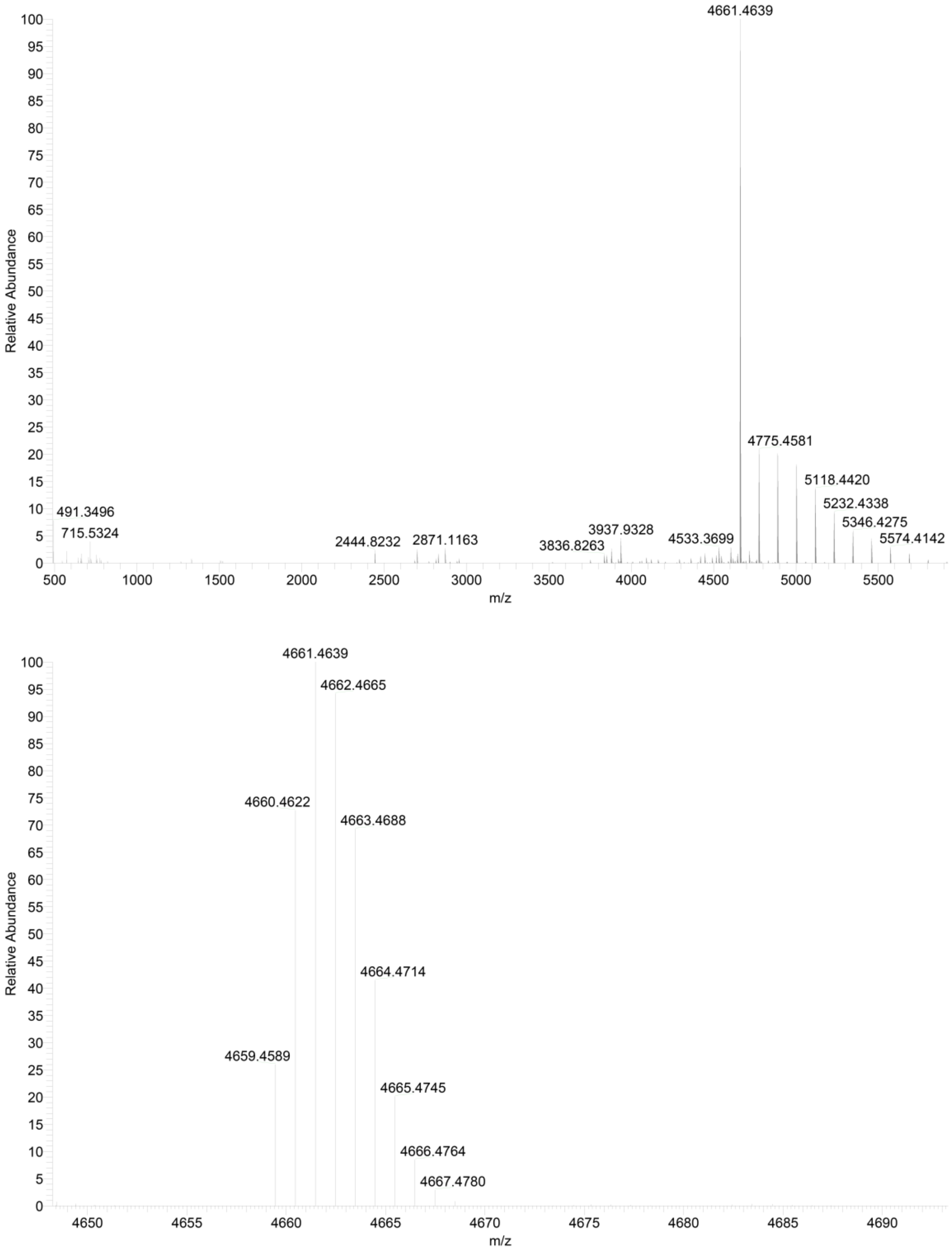

Figure S148. HRMS spectrum.

sr-**X57** ((*K*L)$_8$(*K*KK)$_4$(*K*LL)$_2$*K*KLL) was synthesized by CEM Liberty Blue synthesizer using Rink Amide MBHA resin (320.0 mg, 0.08 mmol, 0.25 mmol·g^{-1}), the dendrimer was obtained as a white foamy solid after preparative RP-HPLC purification (108.7 mg, 18.1%). Analytical RP-HPLC: t_R = 1.30 min (100% A to 100% B in 3.5 min, λ= 214 nm). MS (ESI+): $C_{228}H_{445}N_{63}O_{38}$ calc./obs. 4674.48/4674.50 [M]$^+$.

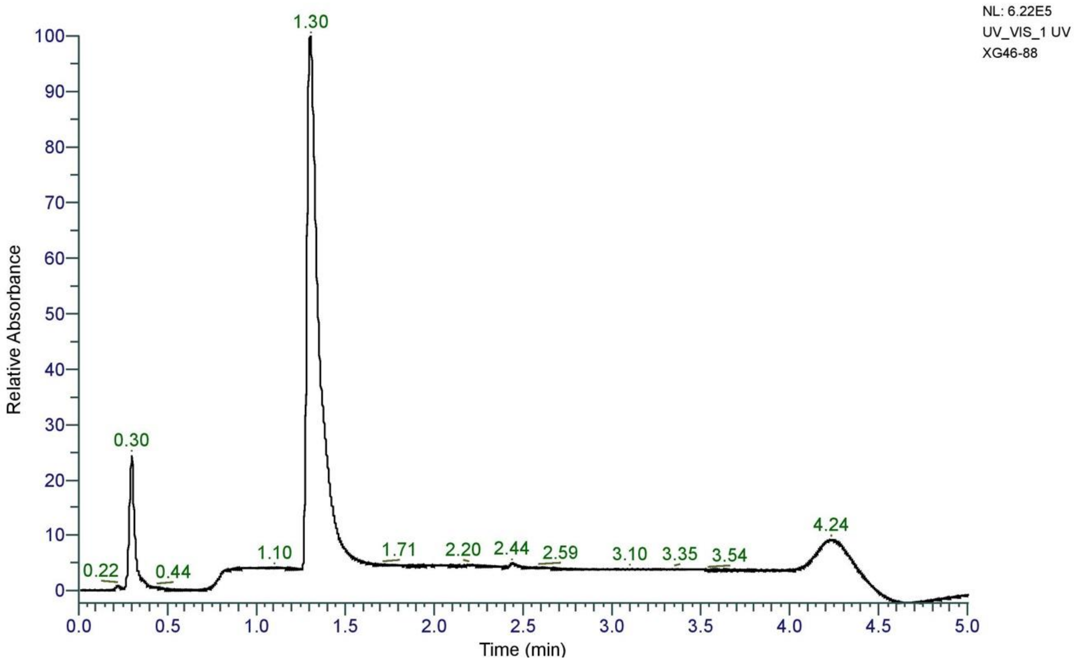

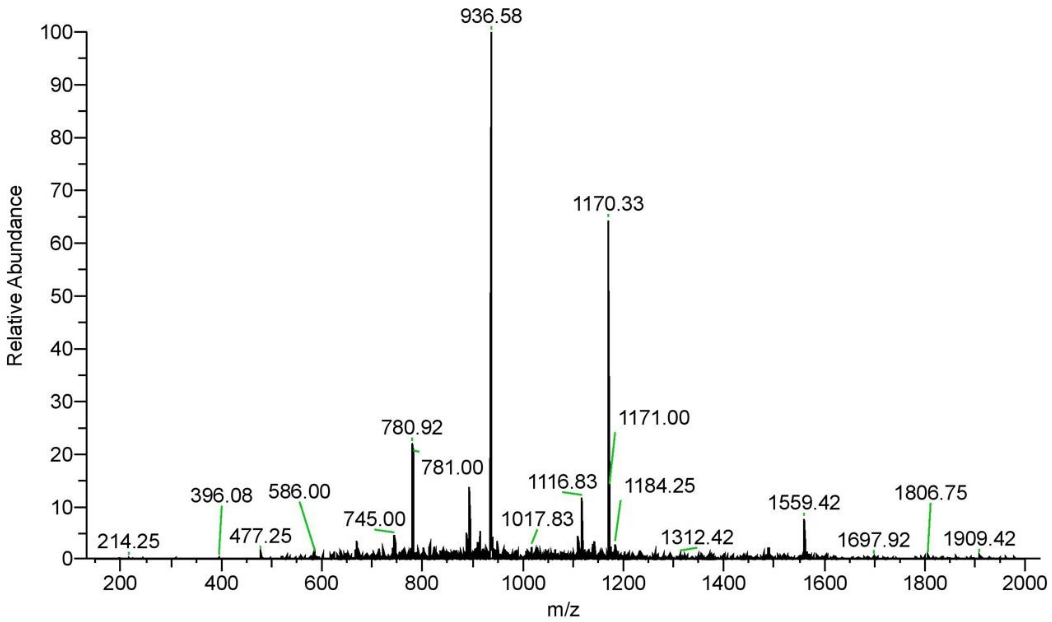

Figure S149. LCMS spectrum.

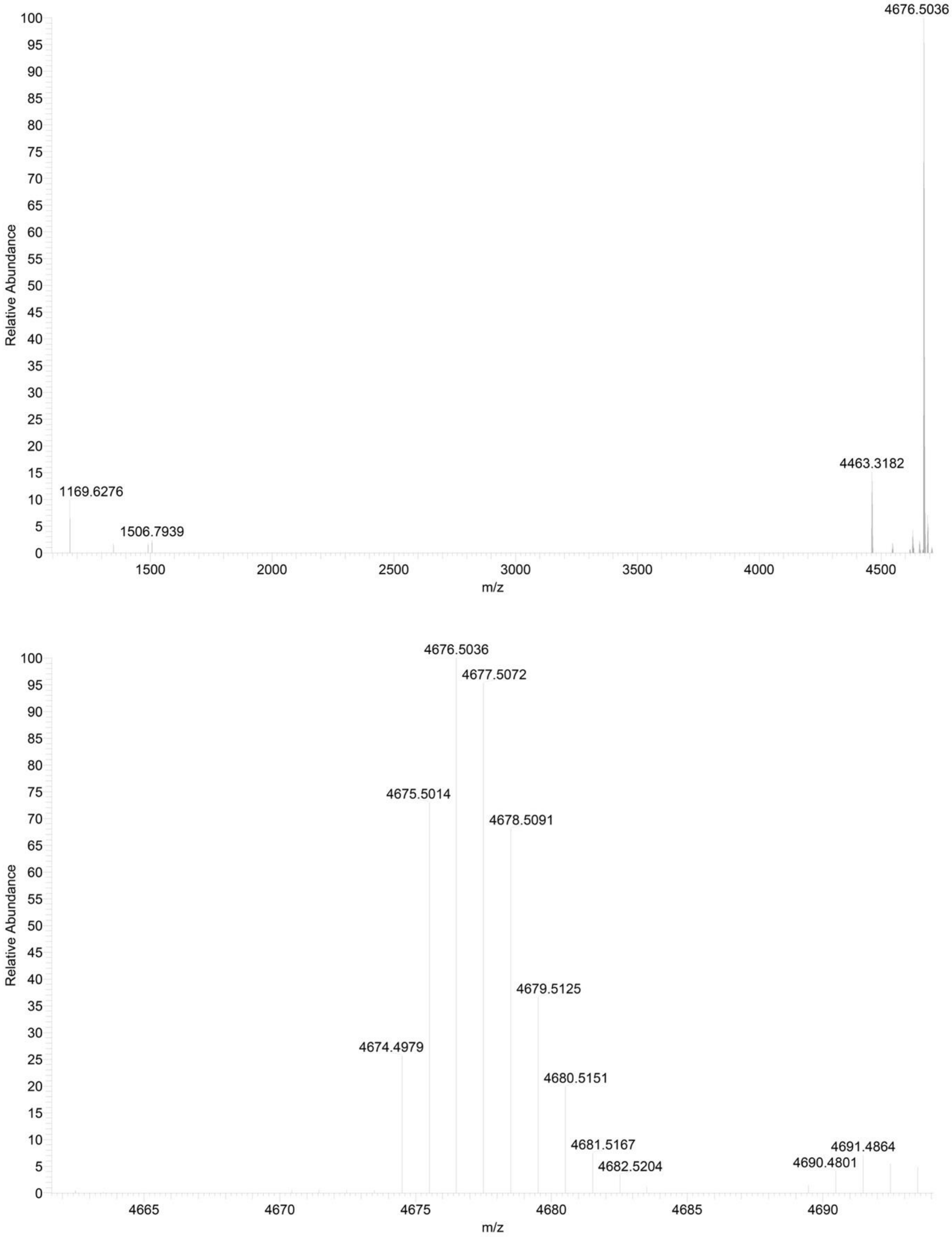

Figure S150. HRMS spectrum.

sr-**X58** ((*K*L)$_8$(*K*KK)$_4$(*K*LL)$_2$*K*LLL) was manually synthesized using TentaGel S RAM resin (393.4 mg, 0.09 mmol, 0.22 mmol·g^{-1}), the dendrimer was obtained as a white foamy solid after preparative RP-HPLC purification (96.5 mg, 16.3%). Analytical RP-HPLC: t_R = 1.35 min (100% A to 100% B in 3.5 min, λ= 214 nm). MS (ESI+): $C_{228}H_{444}N_{62}O_{38}$ calc./obs. 4659.47/4659.47 [M]$^+$.

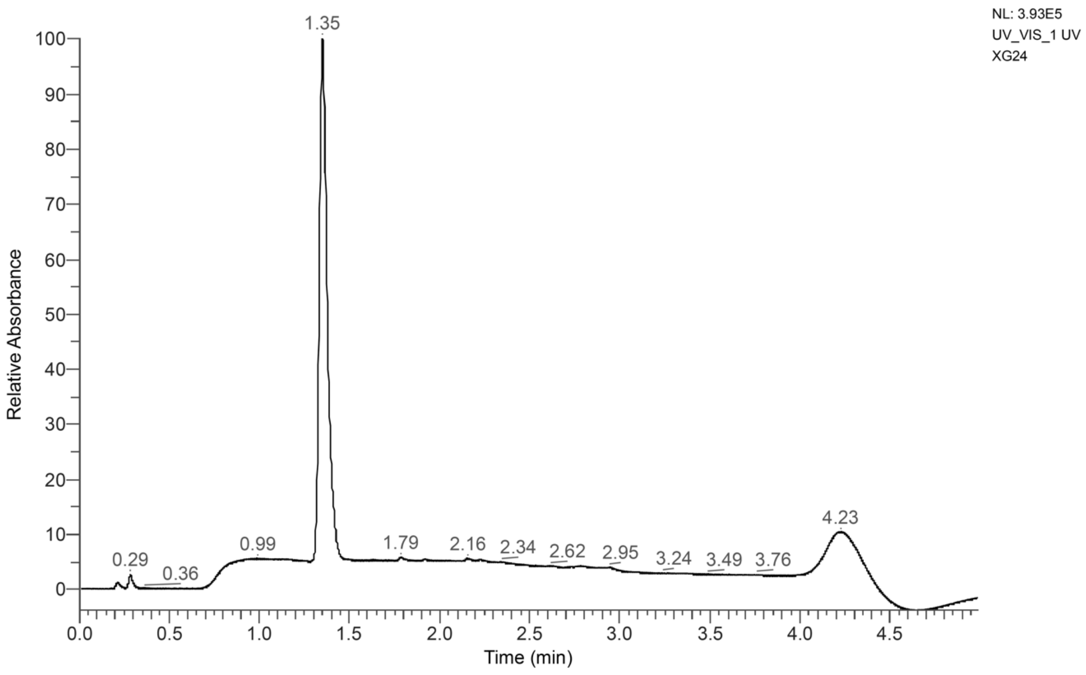

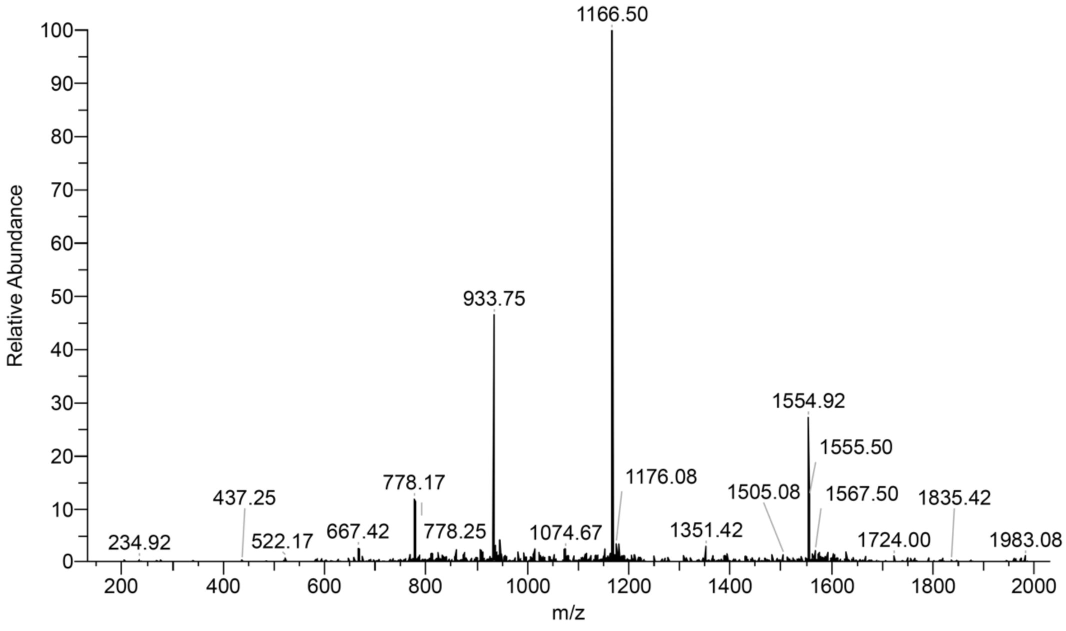

Figure S151. LCMS spectrum.

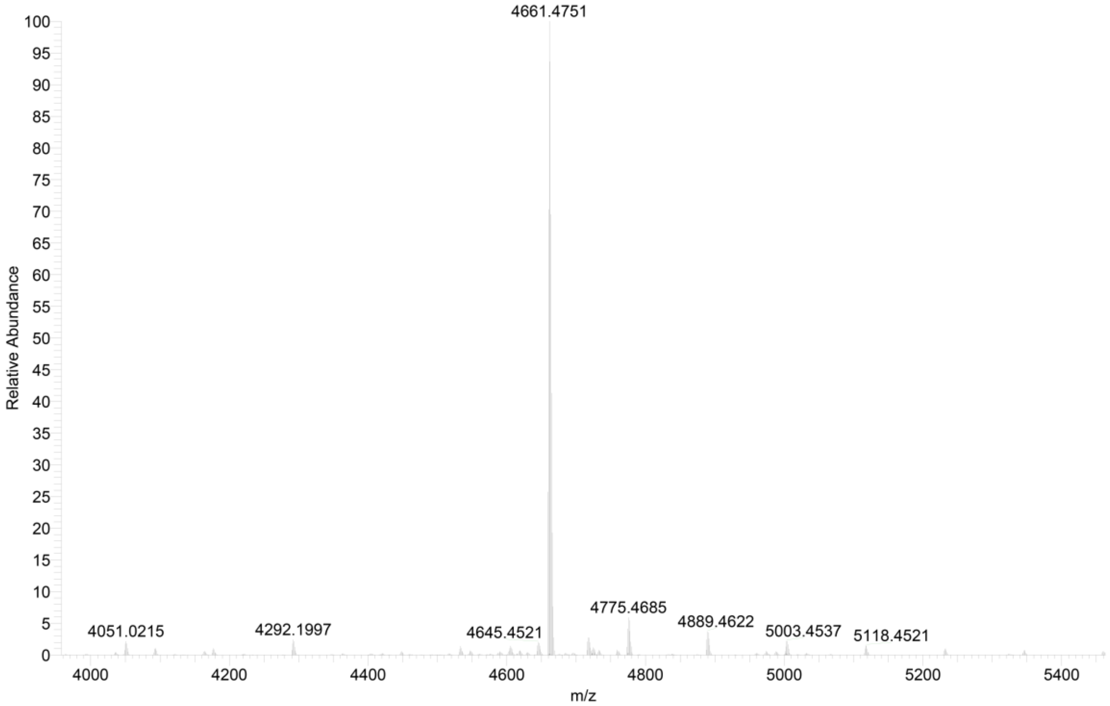

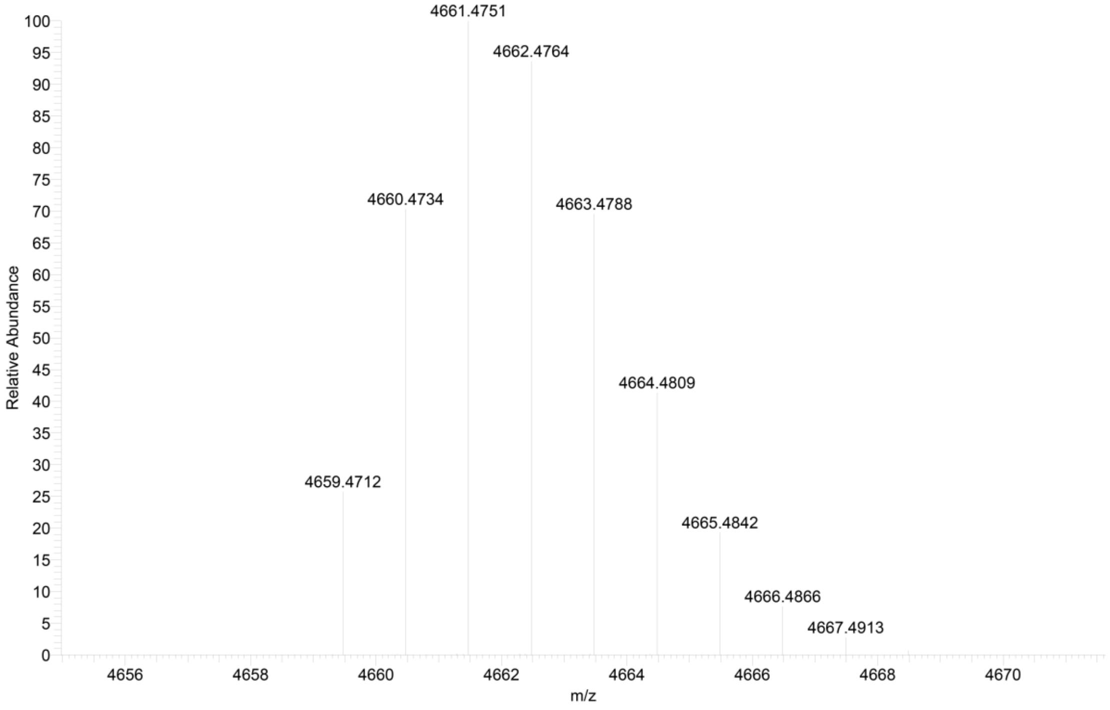

Figure S152. HRMS spectrum.

sr-**X59** ((*K*L)$_8$(*K*KK)$_4$(*K*KL)$_2$*K*LLL) was synthesized by CEM Liberty Blue synthesizer using Rink Amide MBHA resin (320.0 mg, 0.08 mmol, 0.25 mmol·g^{-1}), the dendrimer was obtained as a white foamy solid after preparative RP-HPLC purification (179.7 mg, 29.3%). Analytical RP-HPLC: t_R = 1.29 min (100% A to 100% B in 3.5 min, λ= 214 nm). MS (ESI+): $C_{228}H_{446}N_{64}O_{38}$ calc./obs. 4689.49/4689.51 [M]$^+$.

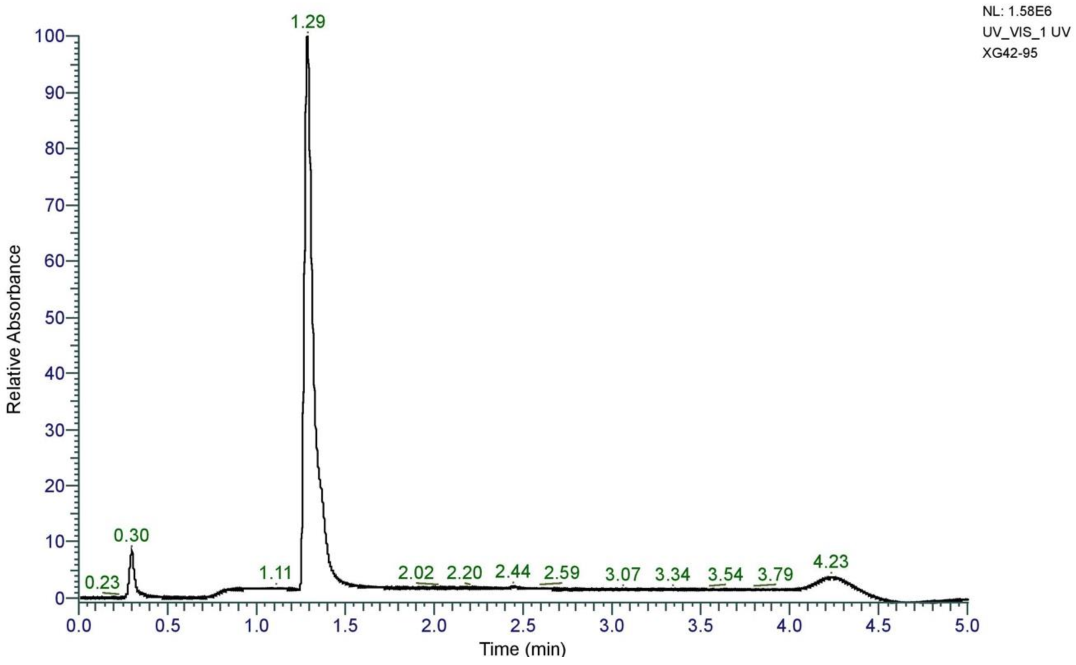

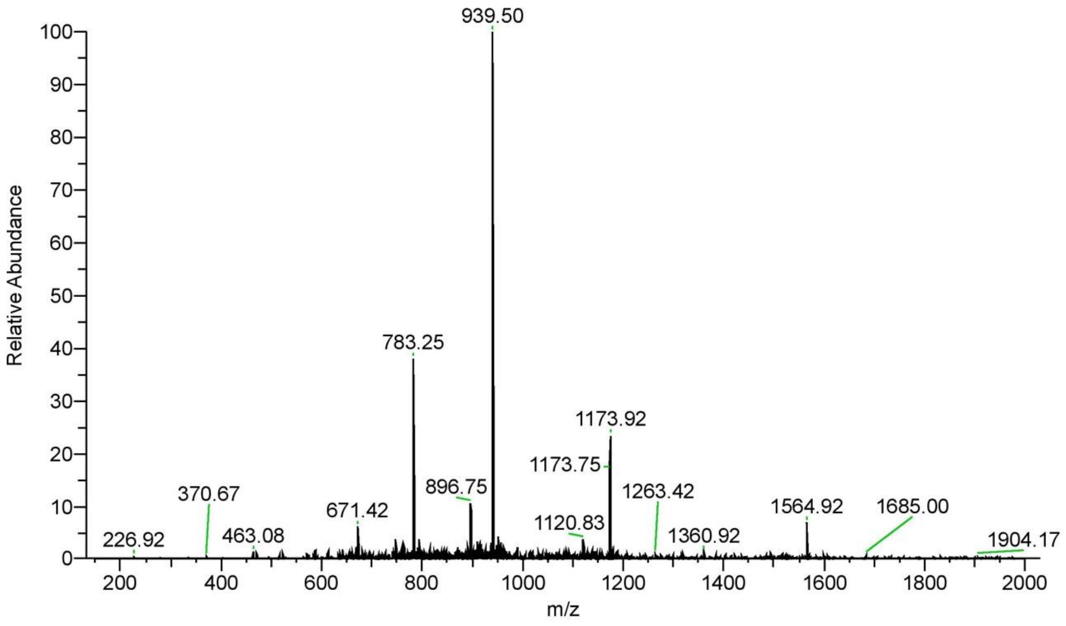

Figure S153. LCMS spectrum.

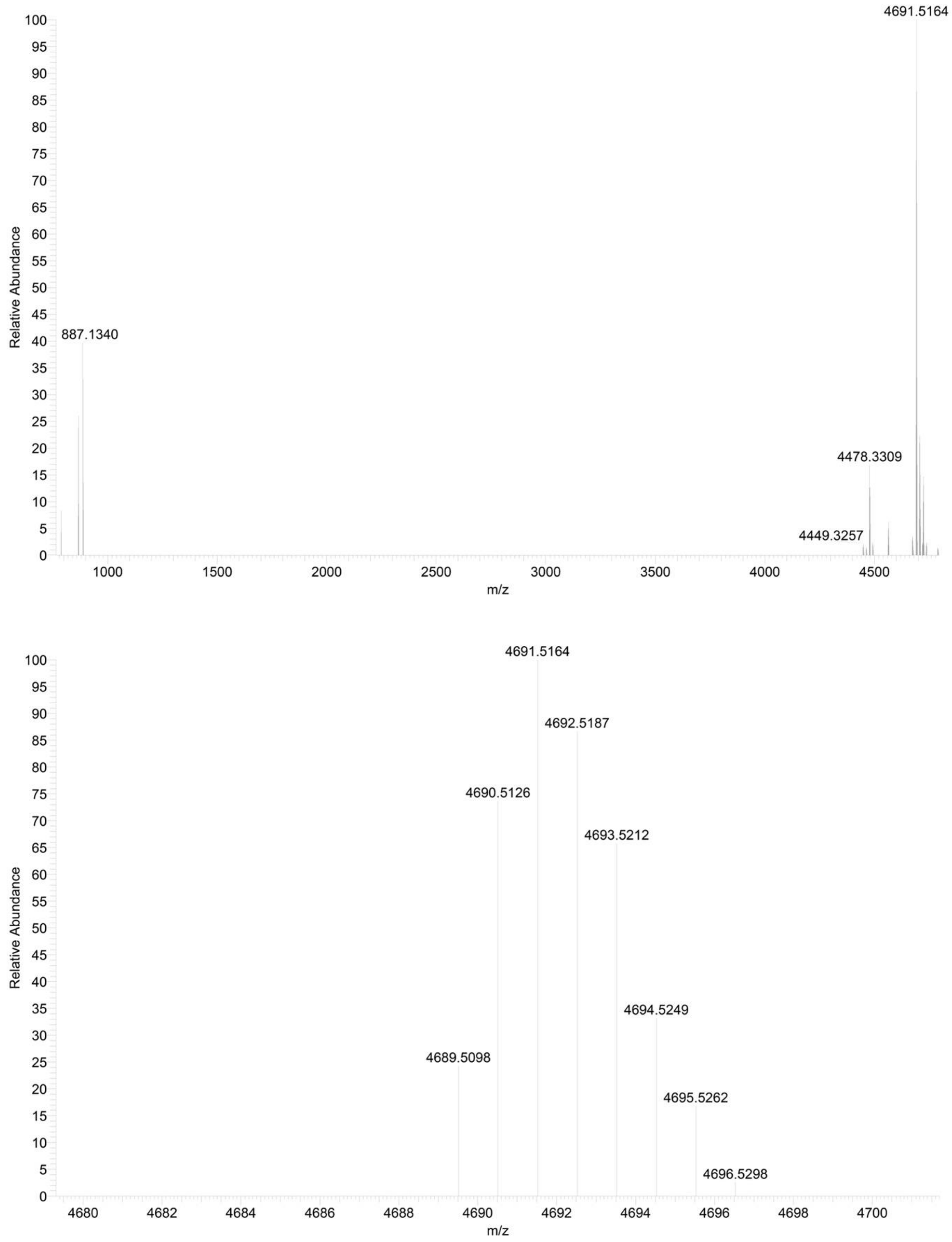

Figure S154. HRMS spectrum.

sr-**X60** ((*K*K)$_8$(*K*LL)$_4$(*K*KL)$_2$*K*KLL) was synthesized by CEM Liberty Blue synthesizer using Rink Amide MBHA resin (320.0 mg, 0.08 mmol, 0.25 mmol·g^{-1}), the dendrimer was obtained as a white foamy solid after preparative RP-HPLC purification (183.0 mg, 29.4%). Analytical RP-HPLC: t_R = 1.32 min (100% A to 100% B in 3.5 min, λ= 214 nm). MS (ESI+): $C_{228}H_{447}N_{65}O_{38}$ calc./obs. 4704.50/4704.50 $[M]^+$.

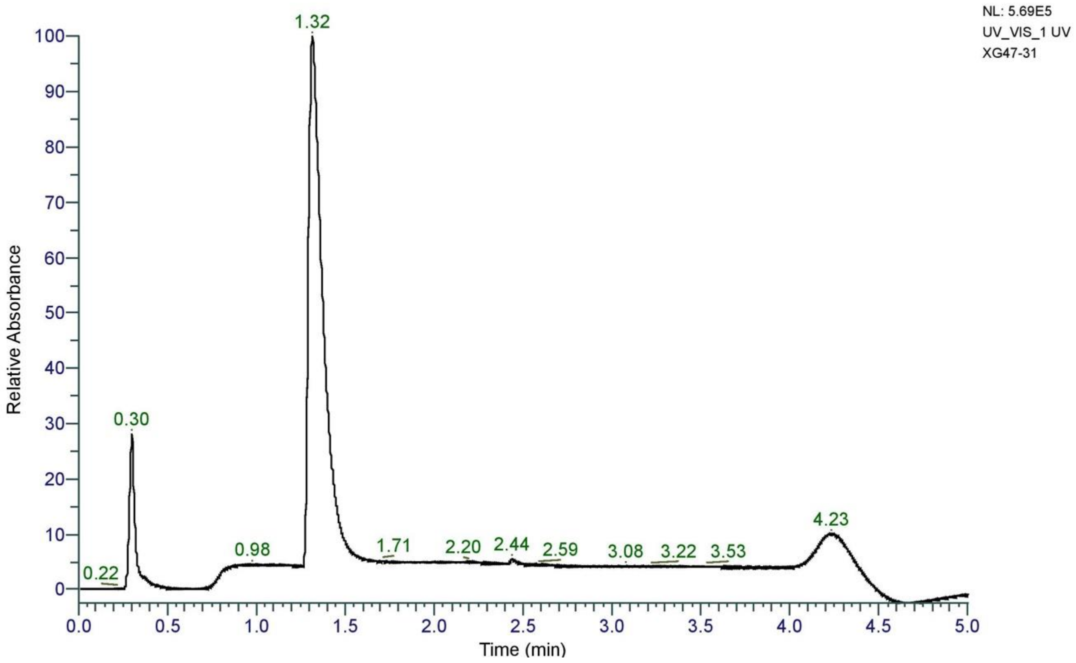

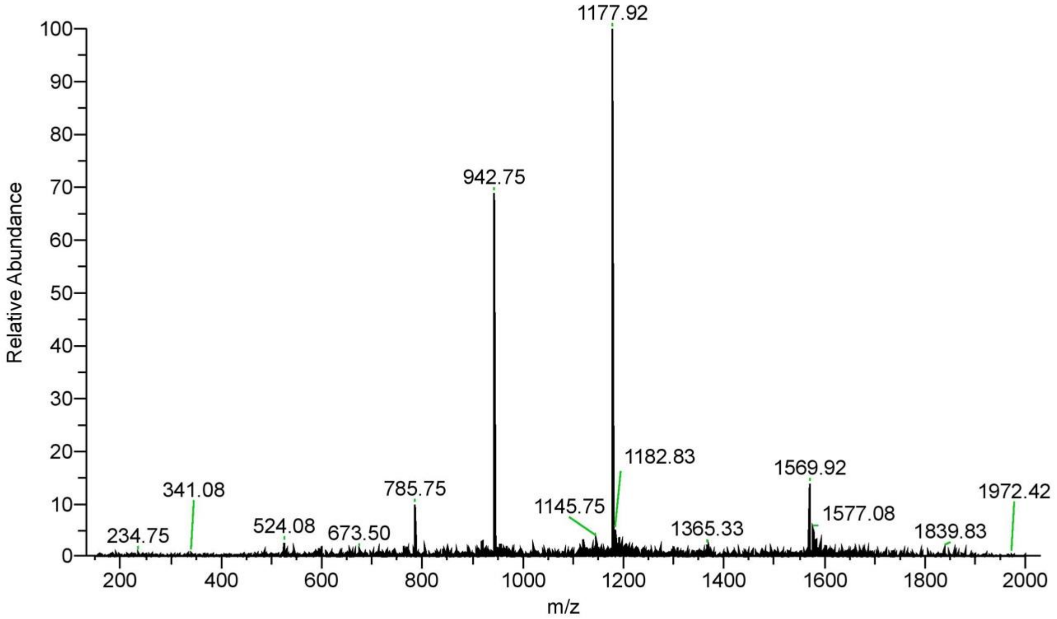

Figure S155. LCMS spectrum.

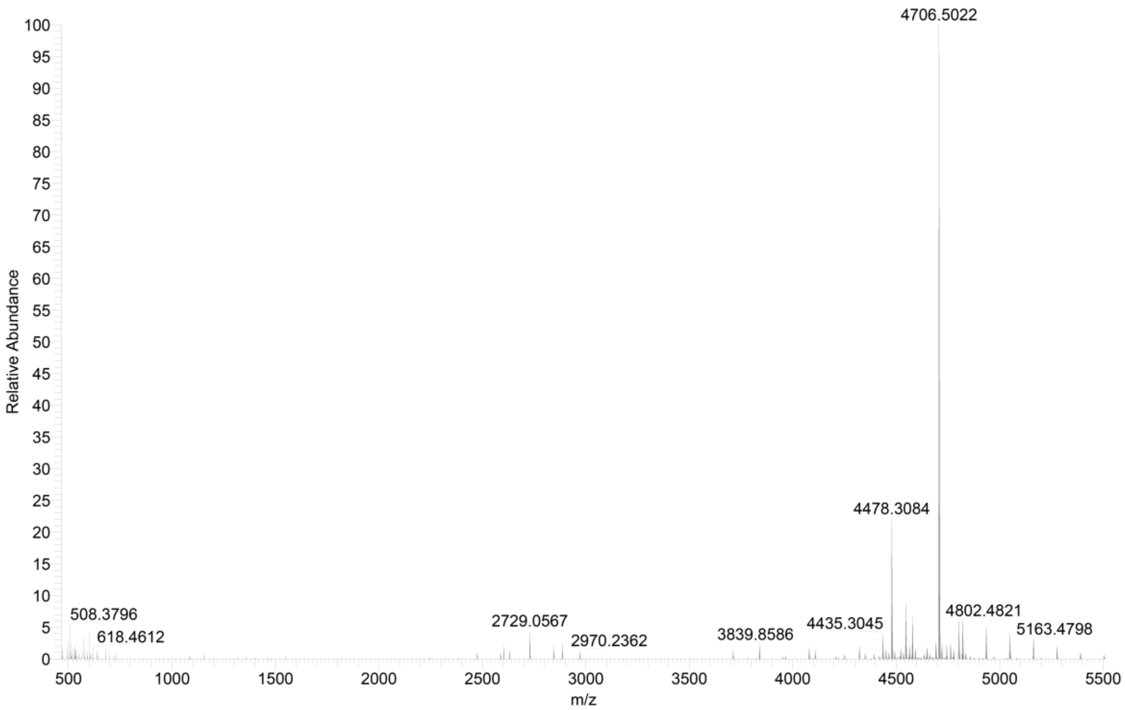

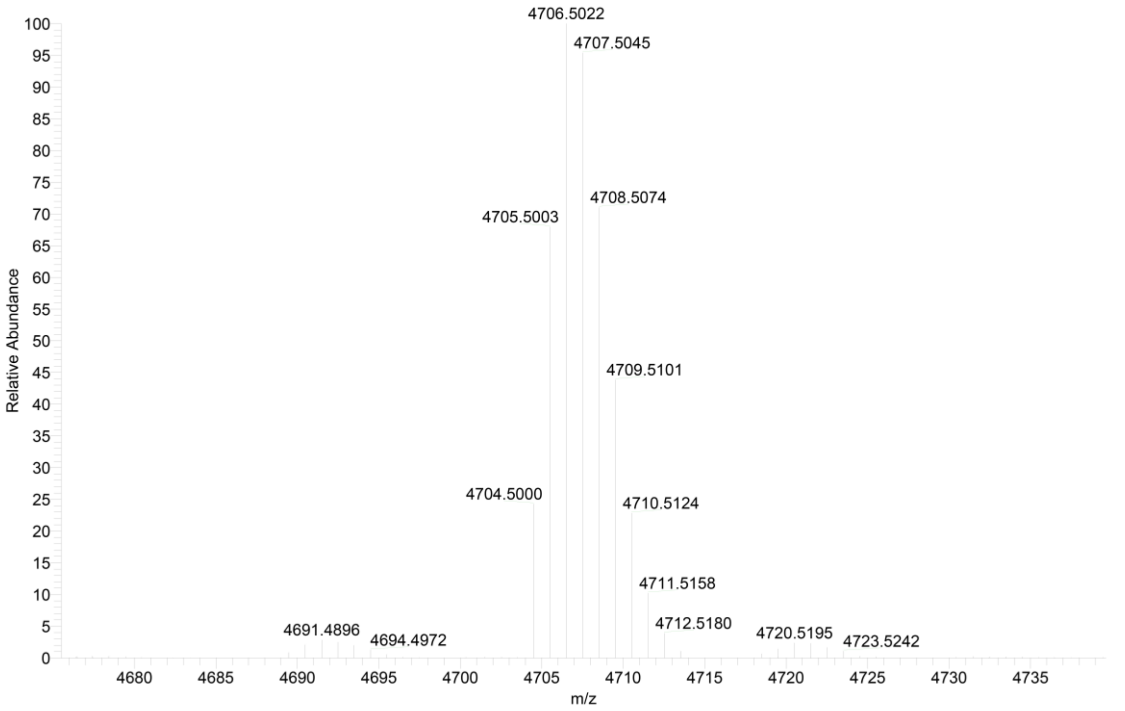

Figure S156. HRMS spectrum.

sr-**X61** ((*K*K)$_8$(*K*LL)$_4$(*K*KL)$_2$*K*KLK) was manually synthesized using TentaGel S RAM resin (393.4 mg, 0.09 mmol, 0.22 mmol·g^{-1}), the dendrimer was obtained as a white foamy solid after preparative RP-HPLC purification (158.5 mg, 25.0%). Analytical RP-HPLC: t_R = 1.27 min (100% A to 100% B in 3.5 min, λ= 214 nm). MS (ESI+): $C_{228}H_{448}N_{66}O_{38}$ calc./obs. 4719.52/4719.51 $[M]^+$.

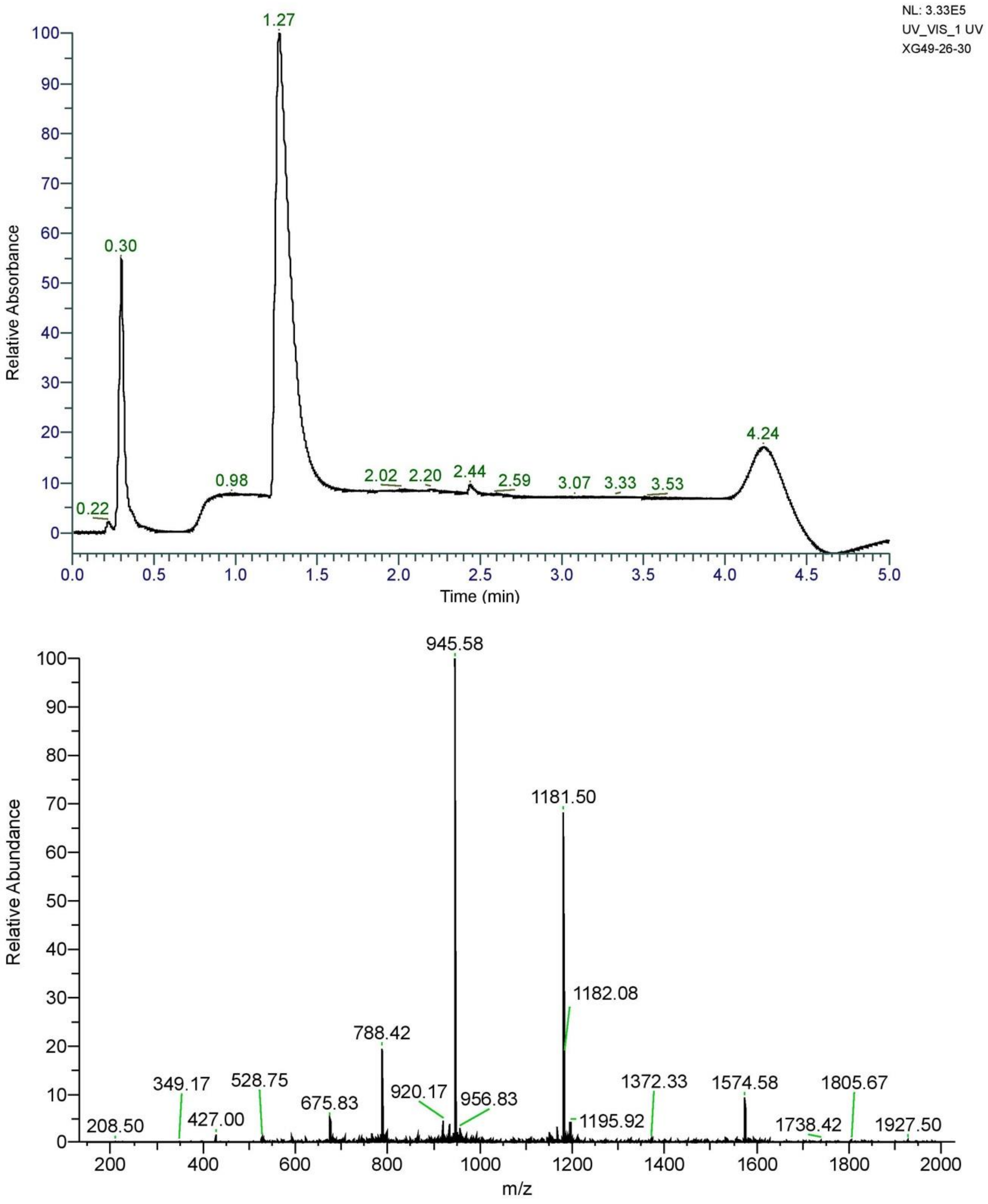

Figure S157. LCMS spectrum.

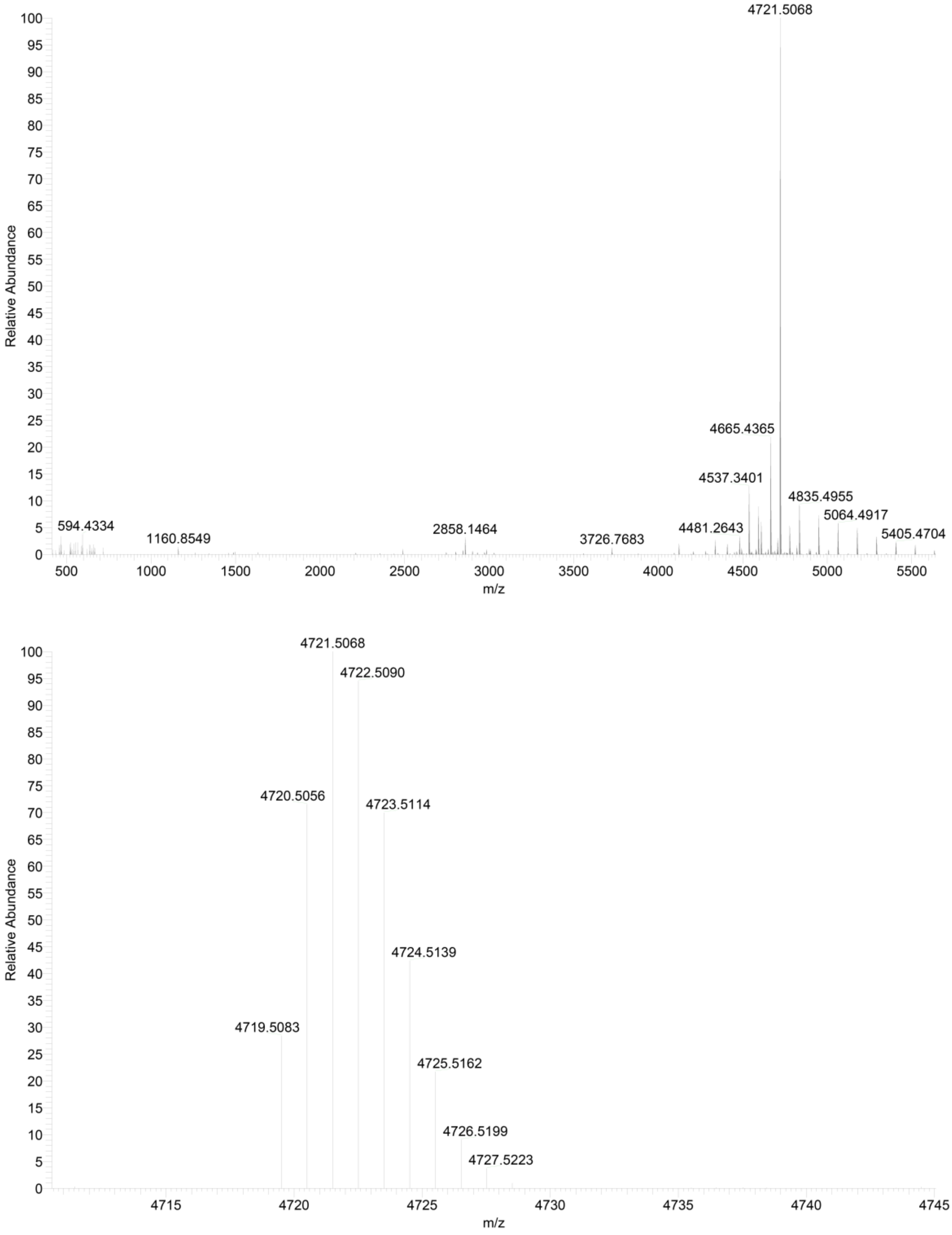

Figure S158. HRMS spectrum.

sr-**X62** ((*K*K)$_8$(*K*LK)$_4$(*K*LL)$_2$*K*KLL) was manually synthesized using TentaGel S RAM resin (393.4 mg, 0.09 mmol, 0.22 mmol·g^{-1}), the dendrimer was obtained as a white foamy solid after preparative RP-HPLC purification (170.4 mg, 26.5%). Analytical RP-HPLC: t_R = 1.29 min (100% A to 100% B in 3.5 min, λ= 214 nm). MS (ESI+): $C_{228}H_{449}N_{67}O_{38}$ calc./obs. 4734.53/4734.53 [M]$^+$.

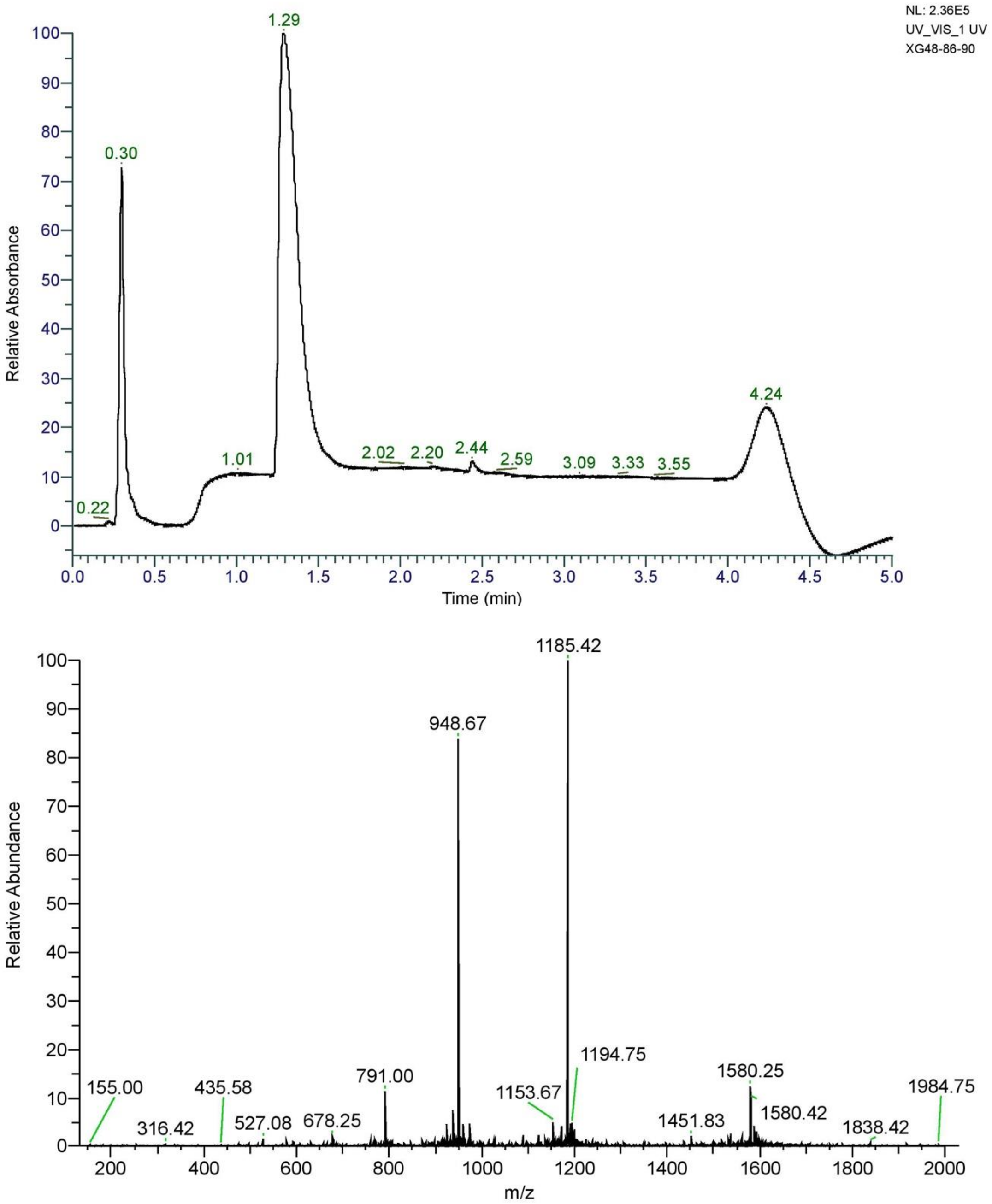

Figure S159. LCMS spectrum.

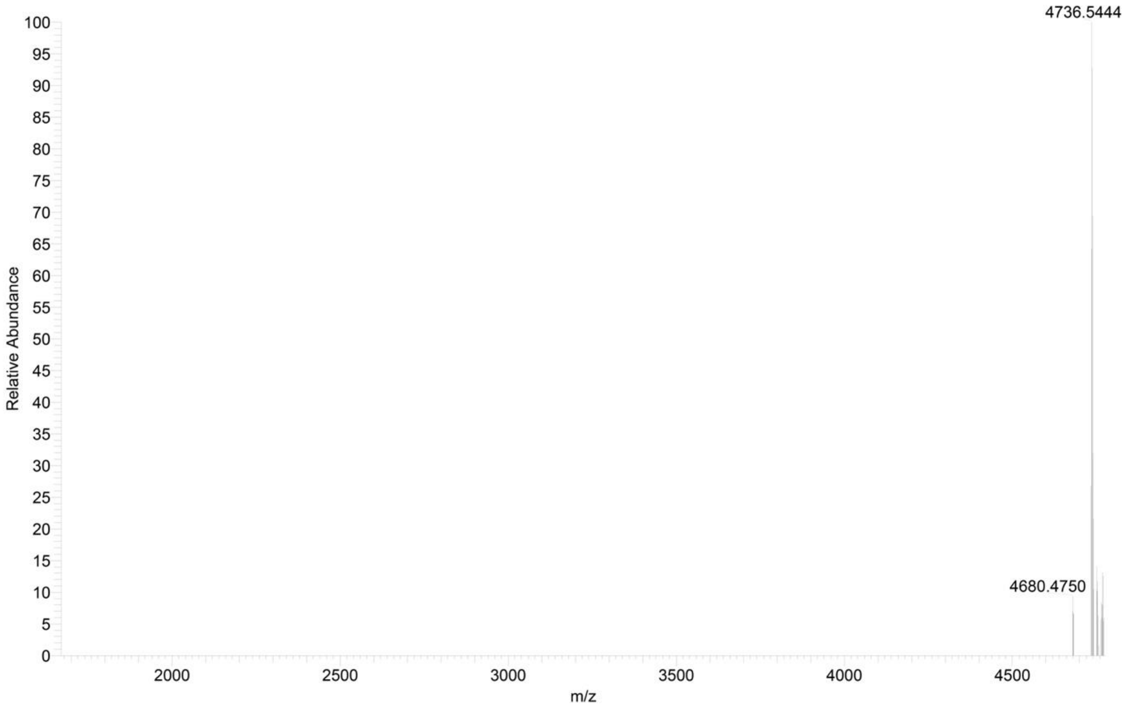

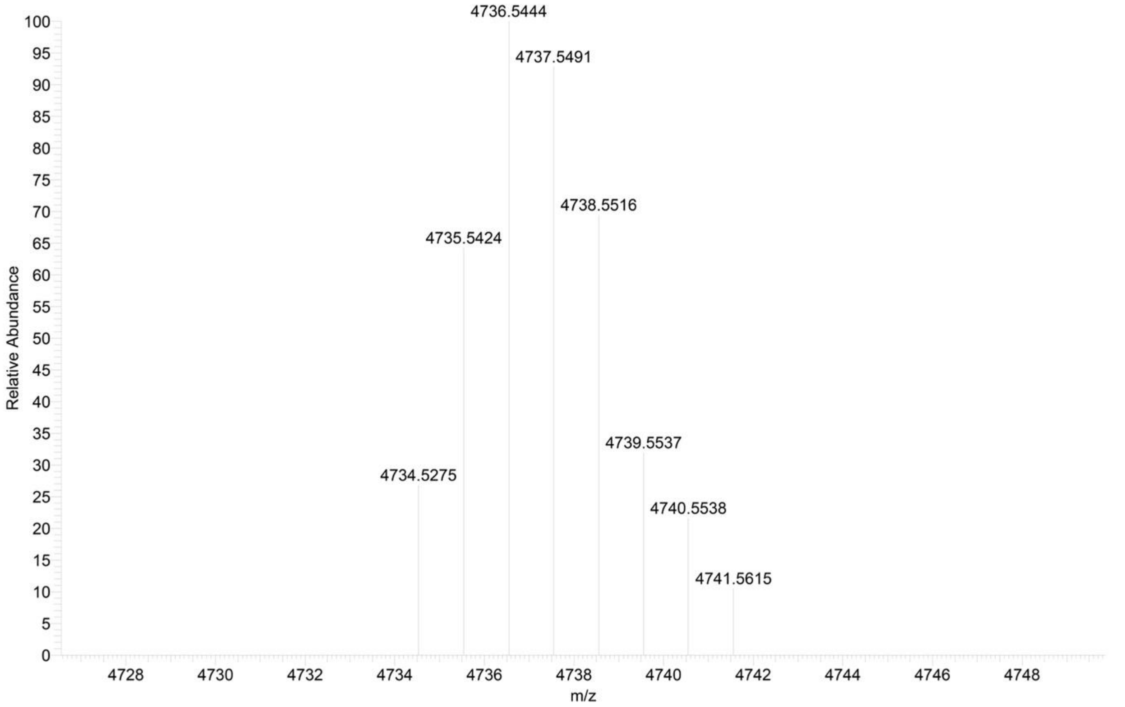

Figure S160. HRMS spectrum.

sr-**X63** ((*K*K)$_8$(*K*KK)$_4$(*K*LL)$_2$*K*LLL) was manually synthesized using TentaGel S RAM resin (393.4 mg, 0.09 mmol, 0.22 mmol·g^{-1}), the dendrimer was obtained as a white foamy solid after preparative RP-HPLC purification (52.1 mg, 7.7%). Analytical RP-HPLC: t_R = 1.22 min (100% A to 100% B in 3.5 min, λ= 214 nm). MS (ESI+): $C_{228}H_{452}N_{70}O_{38}$ calc./obs. 4779.56/4779.56 $[M]^+$.

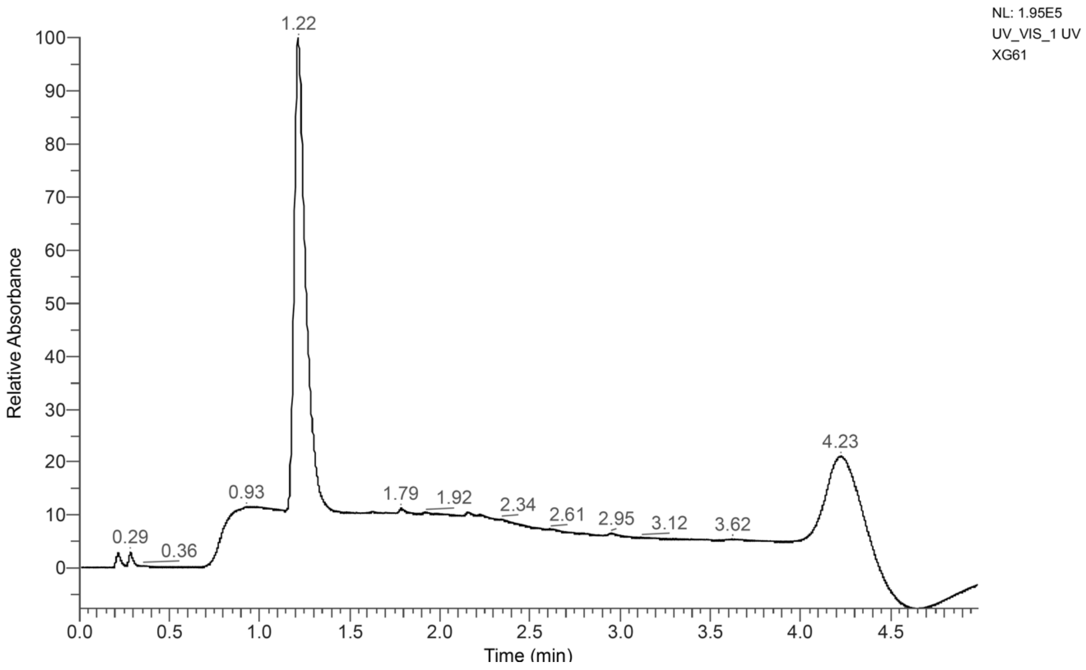

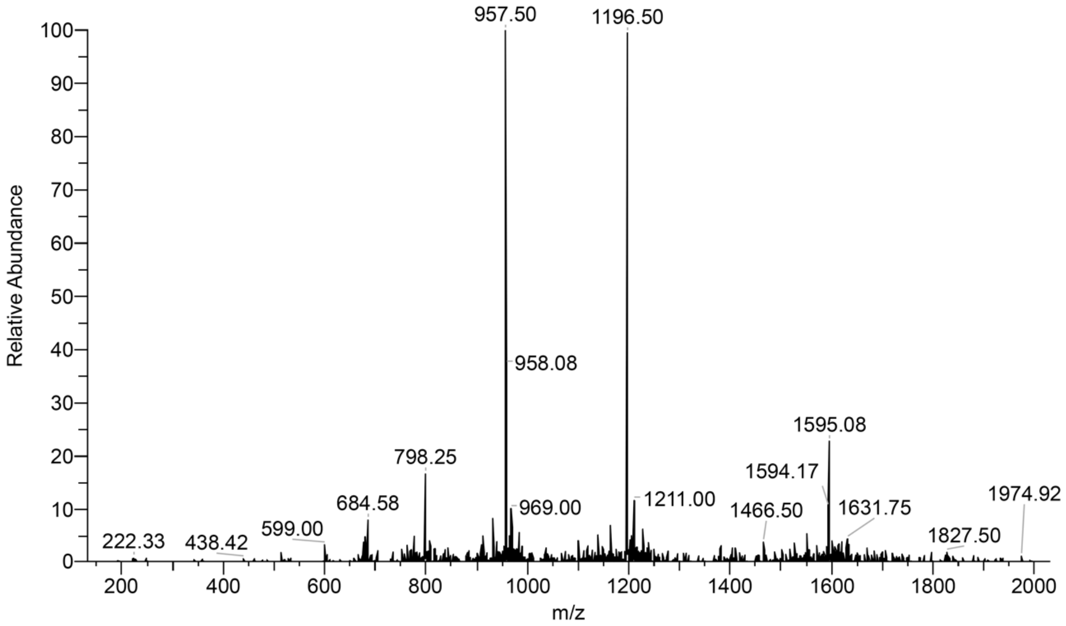

Figure S161. LCMS spectrum.

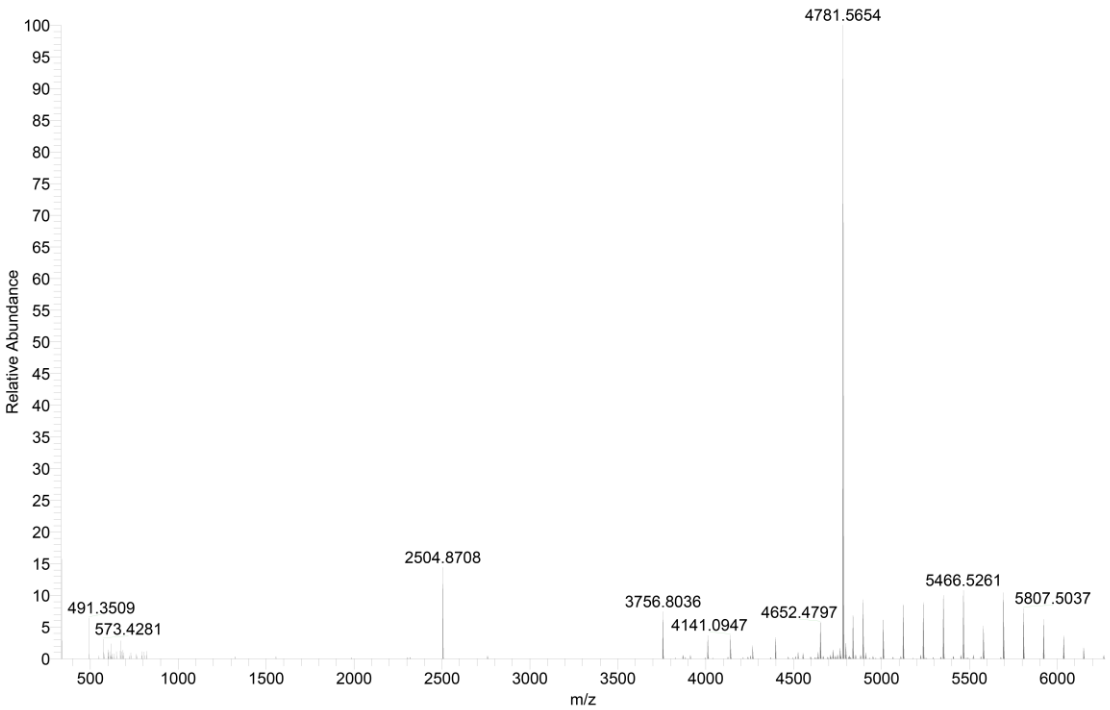

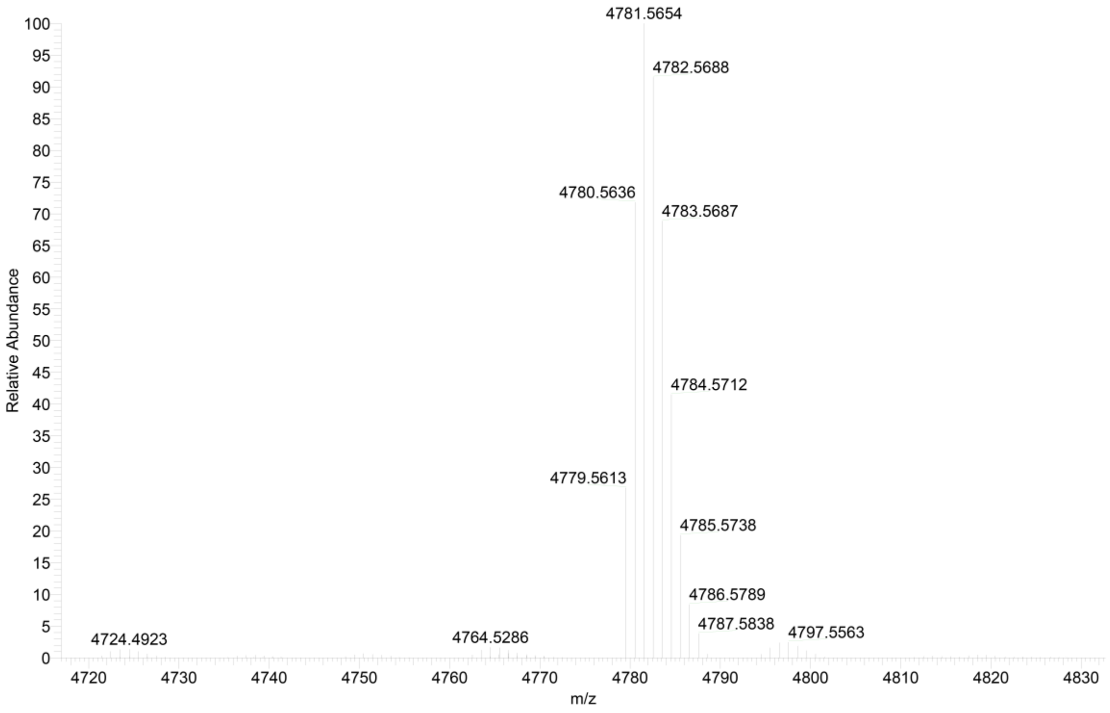

Figure S162. HRMS spectrum.

L-**X18** ((*K*L)$_8$(*K*LK)$_4$(*K*LL)$_2$*K*LLL) was manually synthesized using TentaGel S RAM resin (393.4 mg, 0.09 mmol, 0.22 mmol·g^{-1}), the dendrimer was obtained as a white foamy solid after preparative RP-HPLC purification (187.0 mg, 34.0%). Analytical RP-HPLC: t_R = 1.53 min (100% A to 100% B in 3.5 min, λ= 214 nm). MS (ESI+): $C_{228}H_{440}N_{58}O_{38}$ calc./obs. 4599.43/4599.45 [M]$^+$.

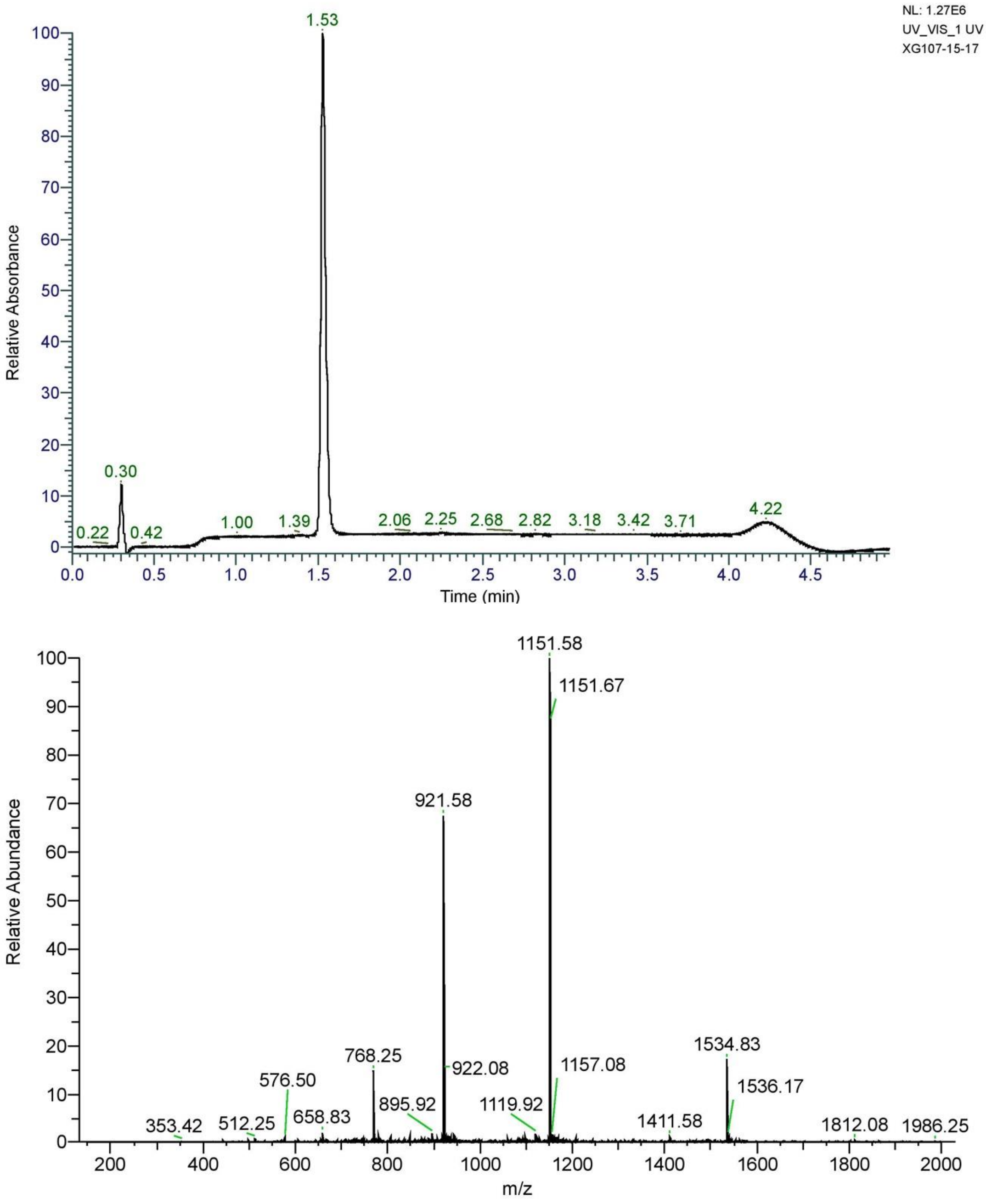

Figure S163. LCMS spectrum.

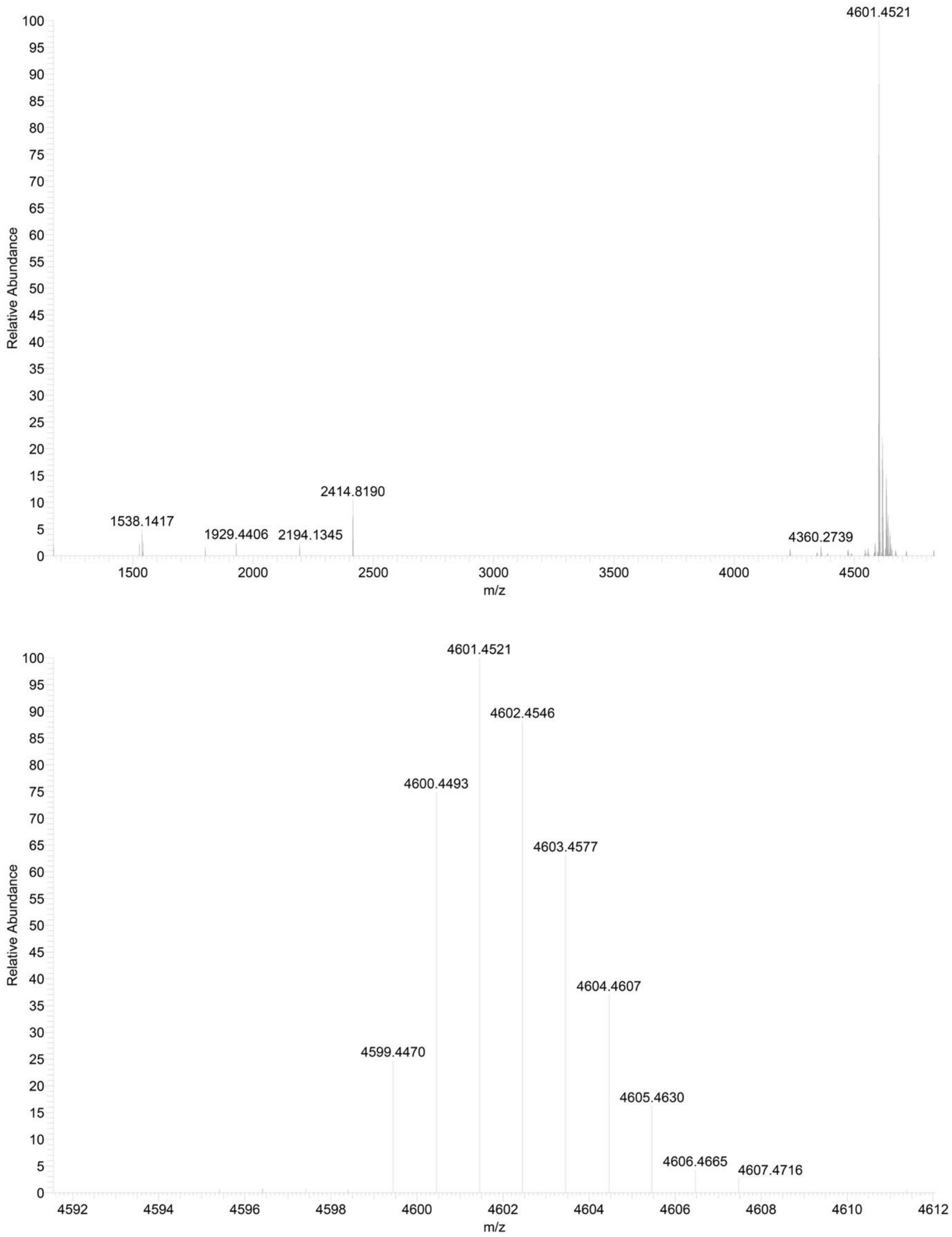

Figure S164. HRMS spectrum.

D-**X18** ((*K*L)$_8$(*K*LK)$_4$(*K*LL)$_2$*K*LLL) was manually synthesized using TentaGel S RAM resin (393.4 mg, 0.09 mmol, 0.22 mmol·g^{-1}), the dendrimer was obtained as a white foamy solid after preparative RP-HPLC purification (112.9 mg, 20.5%). Analytical RP-HPLC: t_R = 1.53 min (100% A to 100% B in 3.5 min, λ= 214 nm). MS (ESI+): $C_{228}H_{440}N_{58}O_{38}$ calc./obs. 4599.43/4599.44 [M]$^+$.

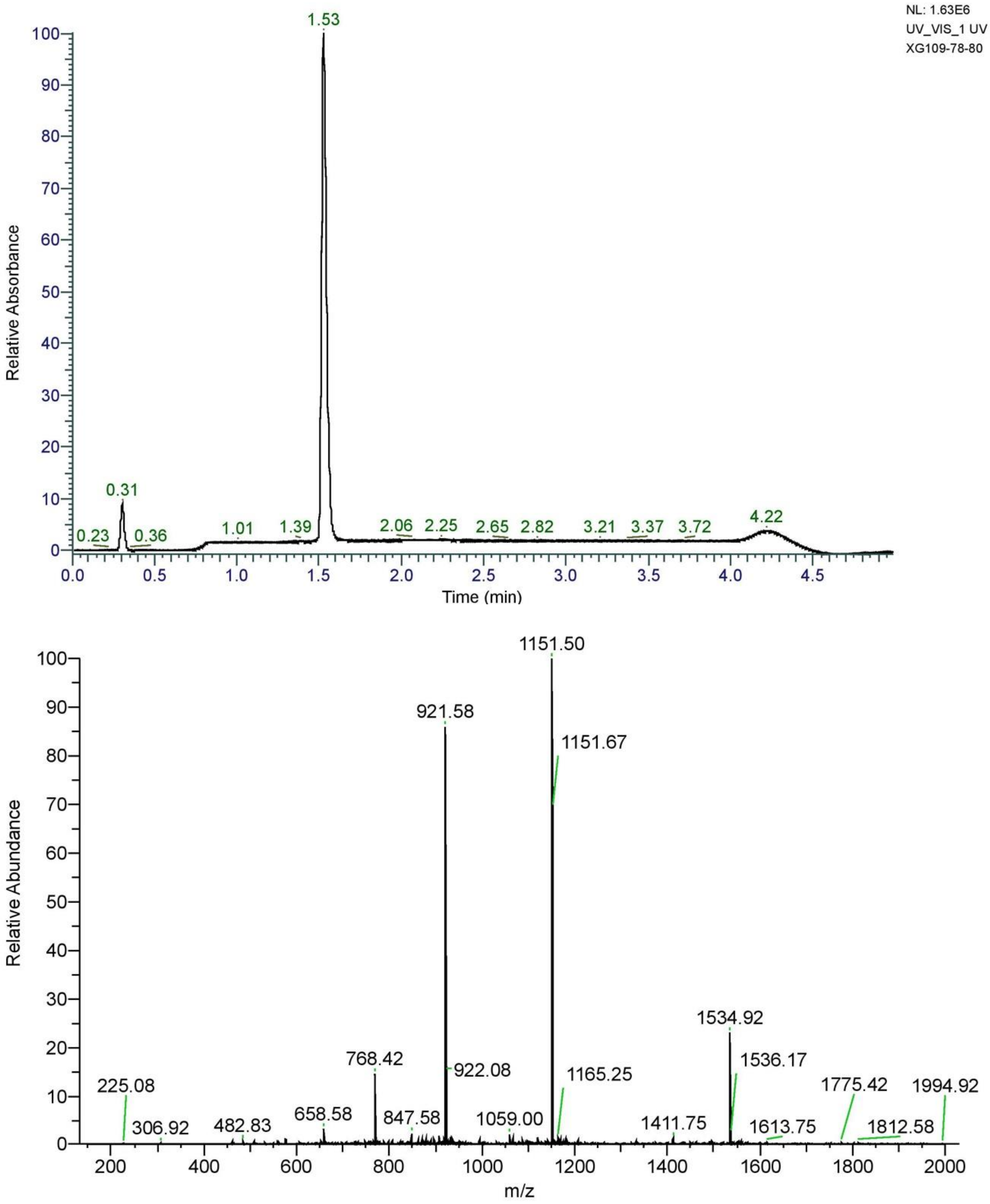

Figure S165. LCMS spectrum.

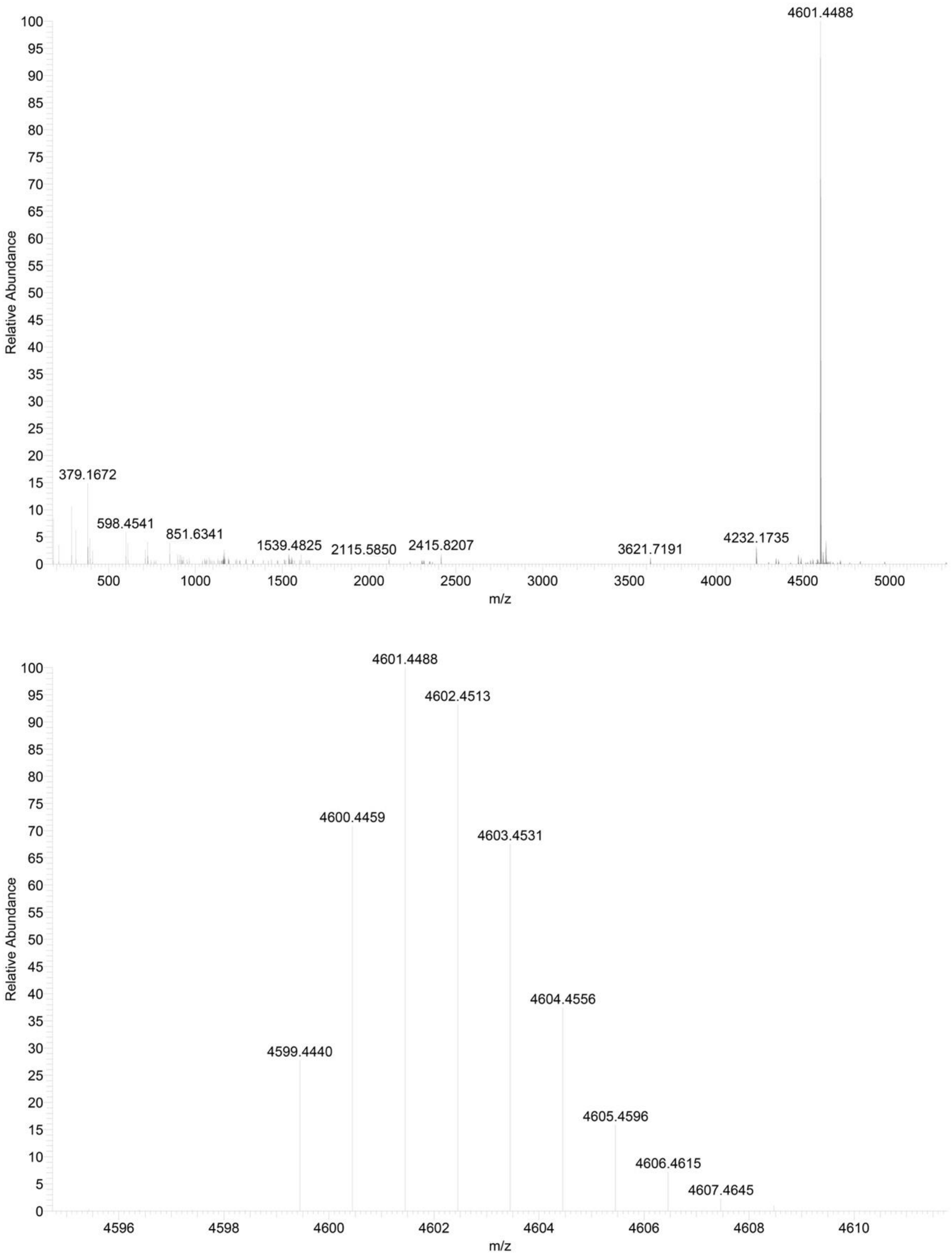

Figure S166. HRMS spectrum.

L-**X22** ((*K*L)$_8$(*K*L)$_4$(*K*KLL)$_2$*K*LKK) was manually synthesized using TentaGel S RAM resin (210.5 mg, 0.08 mmol, 0.38 mmol·g^{-1}), the dendrimer was obtained as a white foamy solid after preparative RP-HPLC purification (37.4 mg, 7.0%). Analytical RP-HPLC: t_R = 1.43 min (100% A to 100% B in 3.5 min, λ= 214 nm). MS (ESI+): $C_{216}H_{418}N_{56}O_{36}$ calc./obs. 4373.26/4373.28 [M]$^+$.

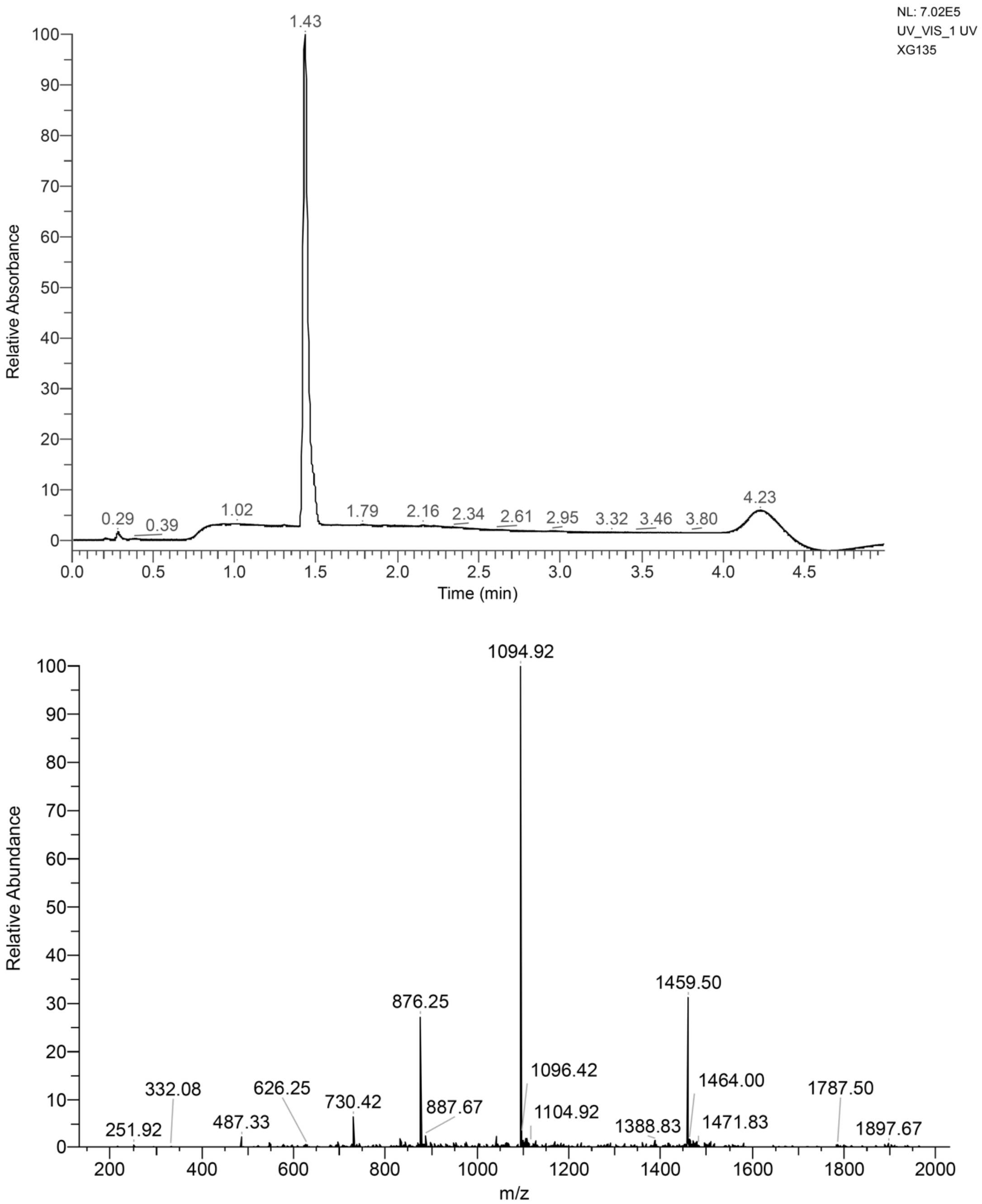

Figure S167. LCMS spectrum.

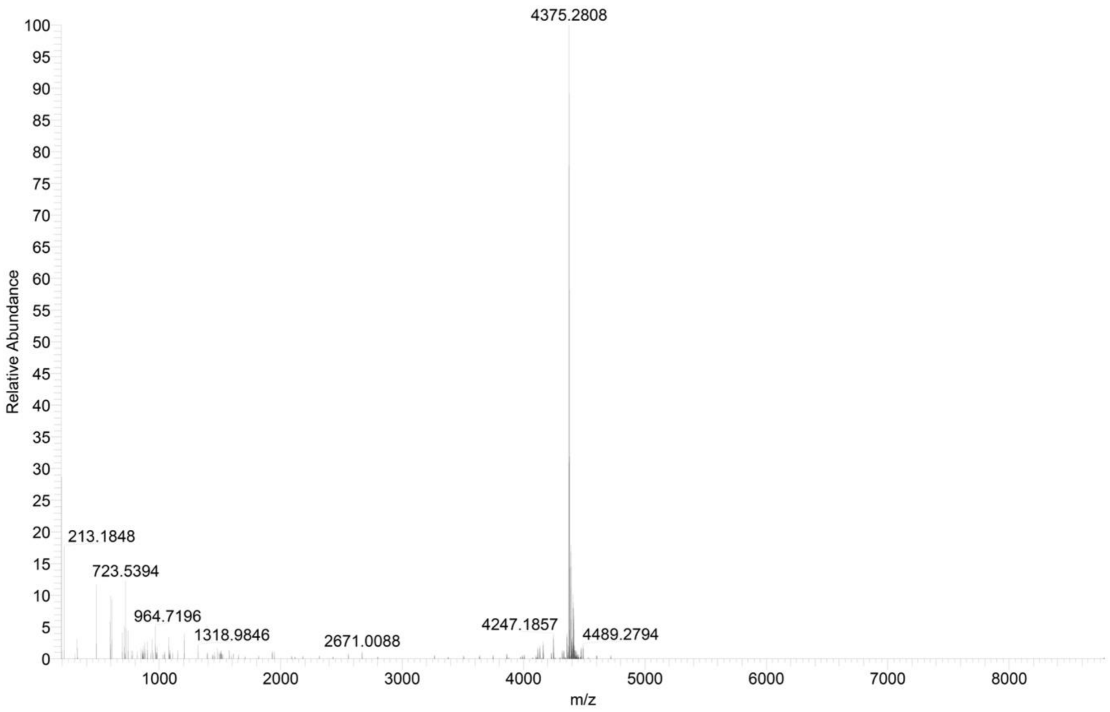

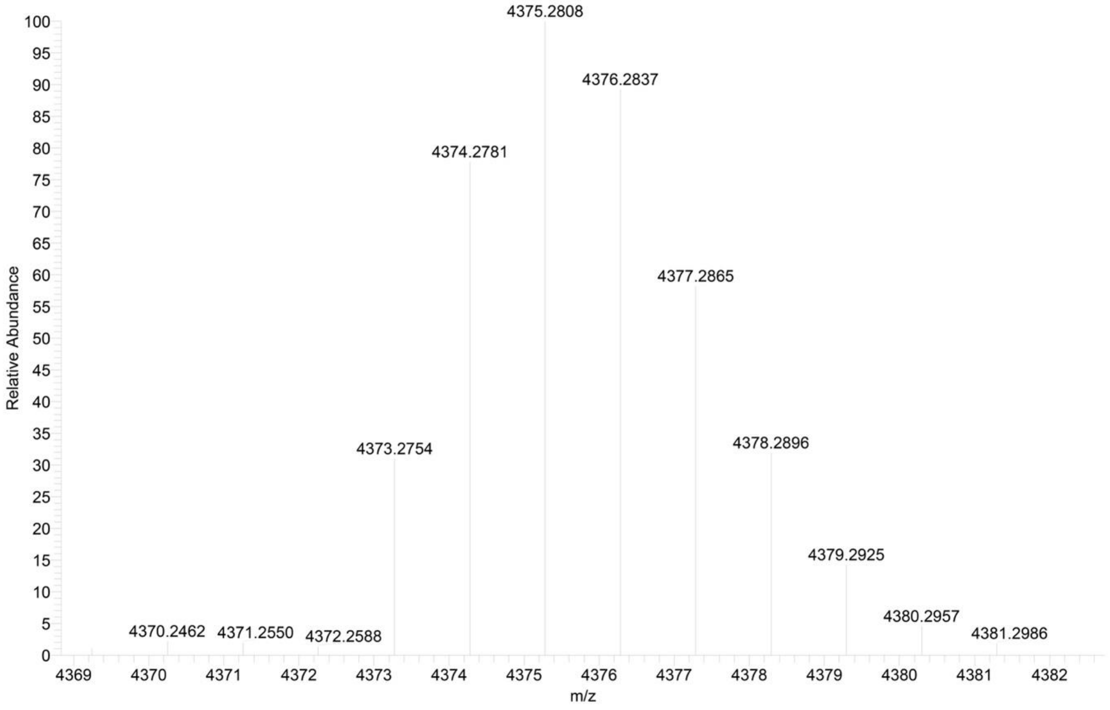

Figure S168. HRMS spectrum.

D-**X22** ((*K*L)$_8$(*K*L)$_4$(*K*KLL)$_2$*K*LKK) was manually synthesized using TentaGel S RAM resin (210.5 mg, 0.08 mmol, 0.38 mmol·g^{-1}), the dendrimer was obtained as a white foamy solid after preparative RP-HPLC purification (171.2 mg, 32.2%). Analytical RP-HPLC: t_R = 1.43 min (100% A to 100% B in 3.5 min, λ= 214 nm). MS (ESI+): $C_{216}H_{418}N_{56}O_{36}$ calc./obs. 4373.26/4373.34 [M]$^+$.

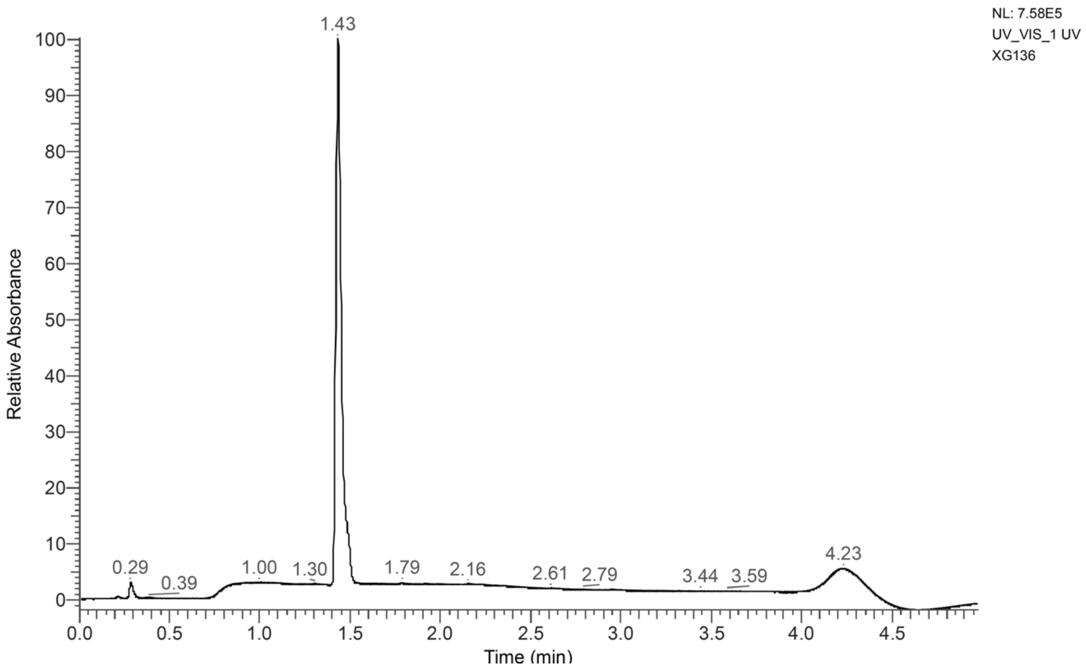

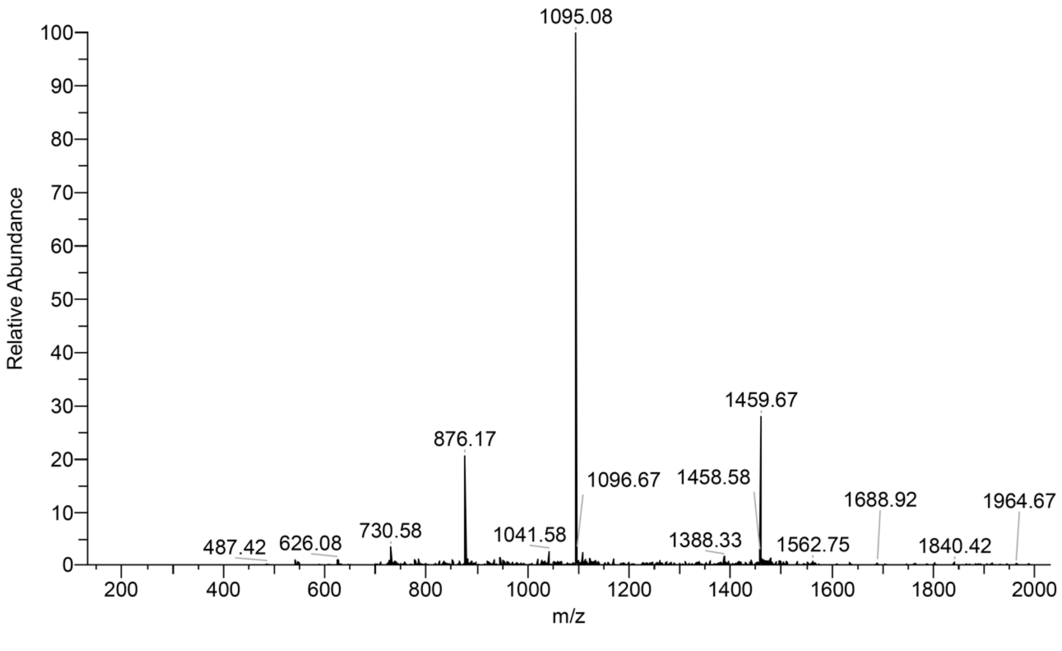

Figure S169. LCMS spectrum.

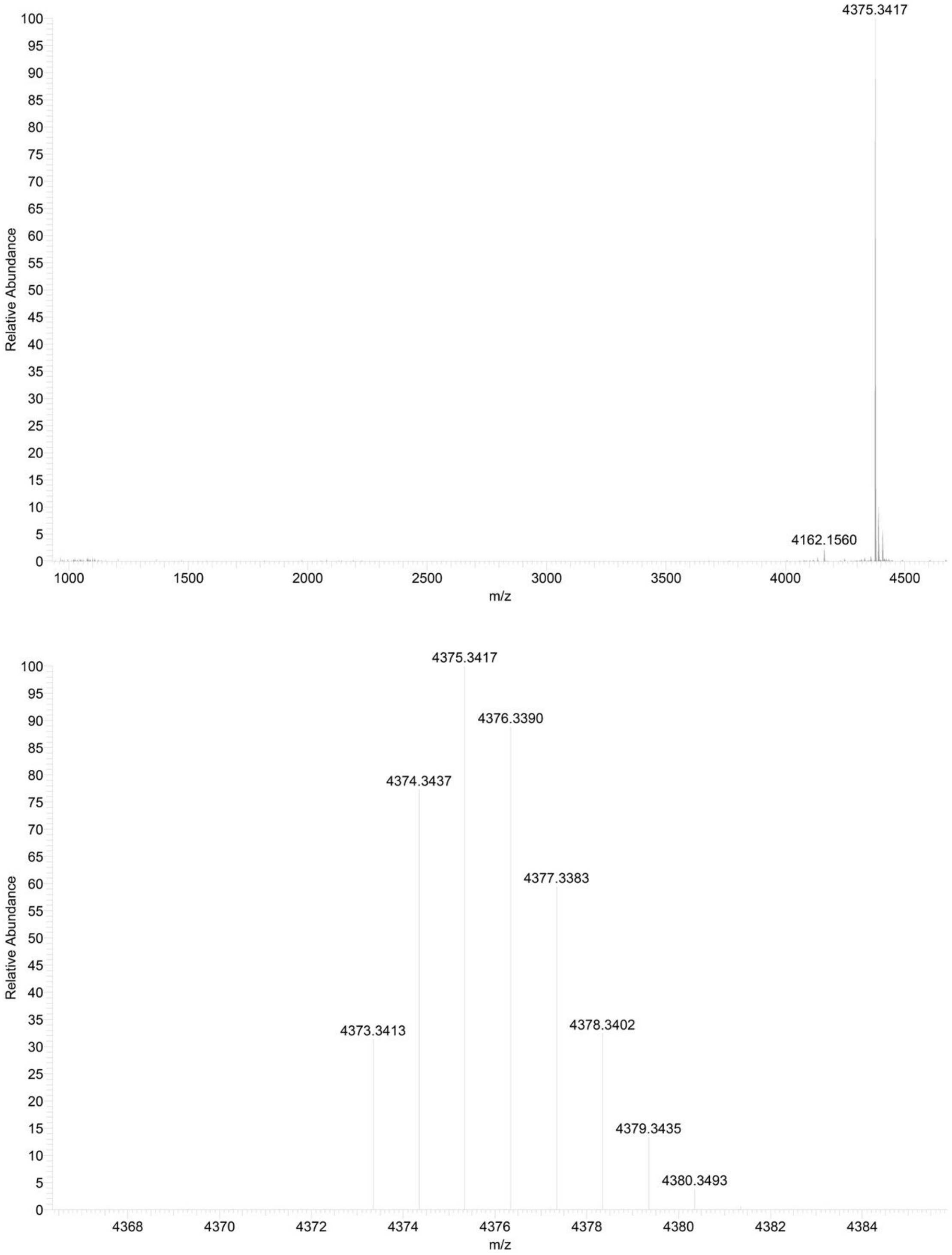

Figure S170. HRMS spectrum.

sr-**aX18** ((*Ahx*L)$_8$(KLK)$_4$(KLL)$_2$KLLL) was manually synthesized using TentaGel S RAM resin (393.4 mg, 0.09 mmol, 0.22 mmol·g^{-1}), the dendrimer was obtained as a white foamy solid after preparative RP-HPLC purification (74.9 mg, 16.0%). Analytical RP-HPLC: t_R = 1.58 min (100% A to 100% B in 3.5 min, λ= 214 nm). MS (ESI+): $C_{228}H_{432}N_{50}O_{38}$ calc./obs. 4479.34/4479.35 $[M]^+$.

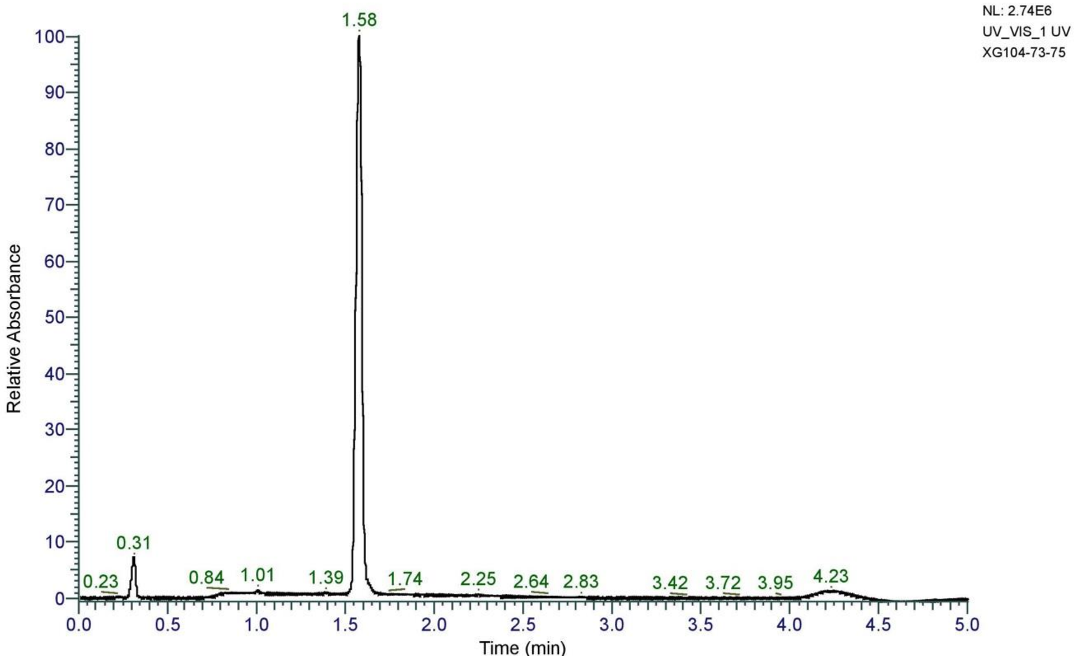

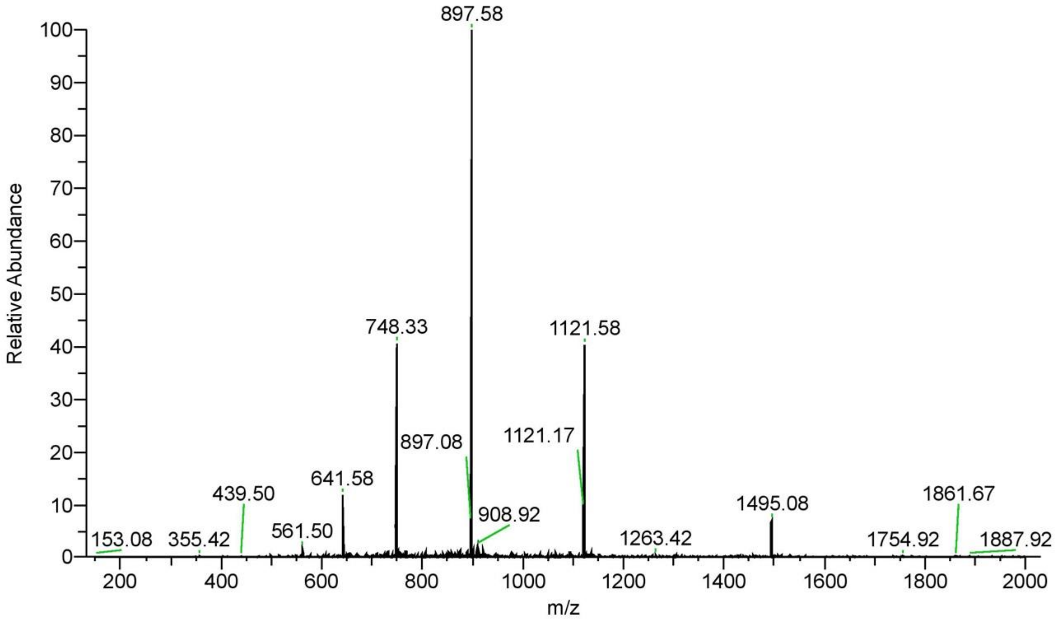

Figure S171. LCMS spectrum.

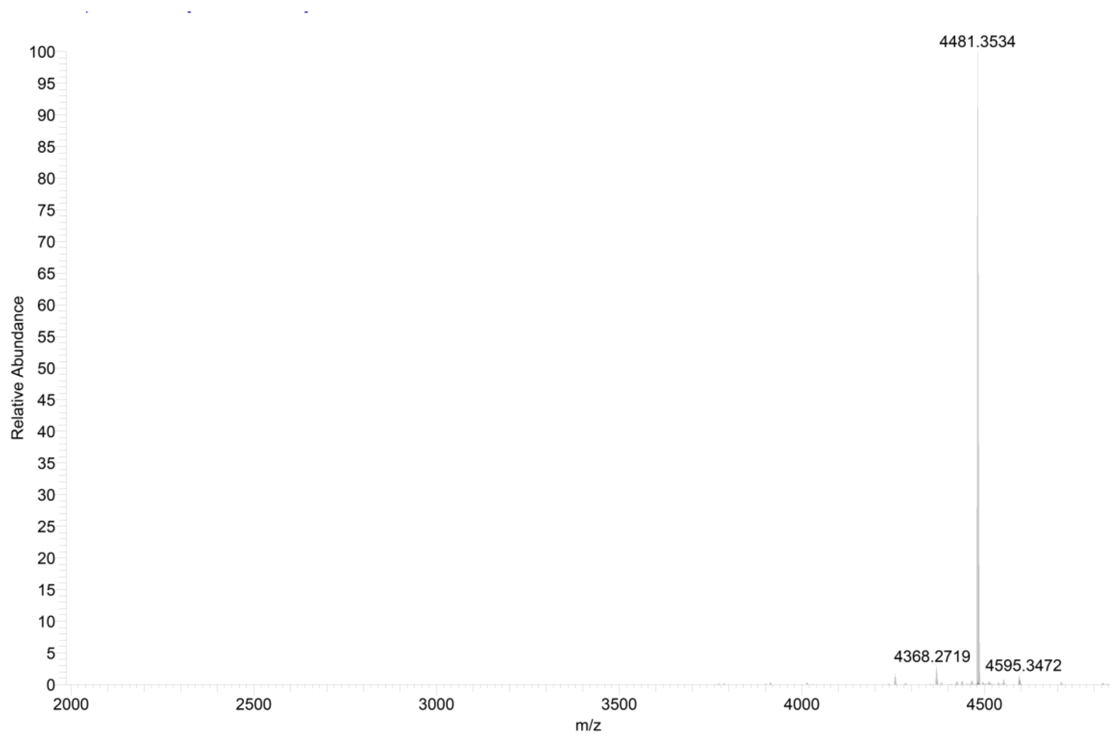

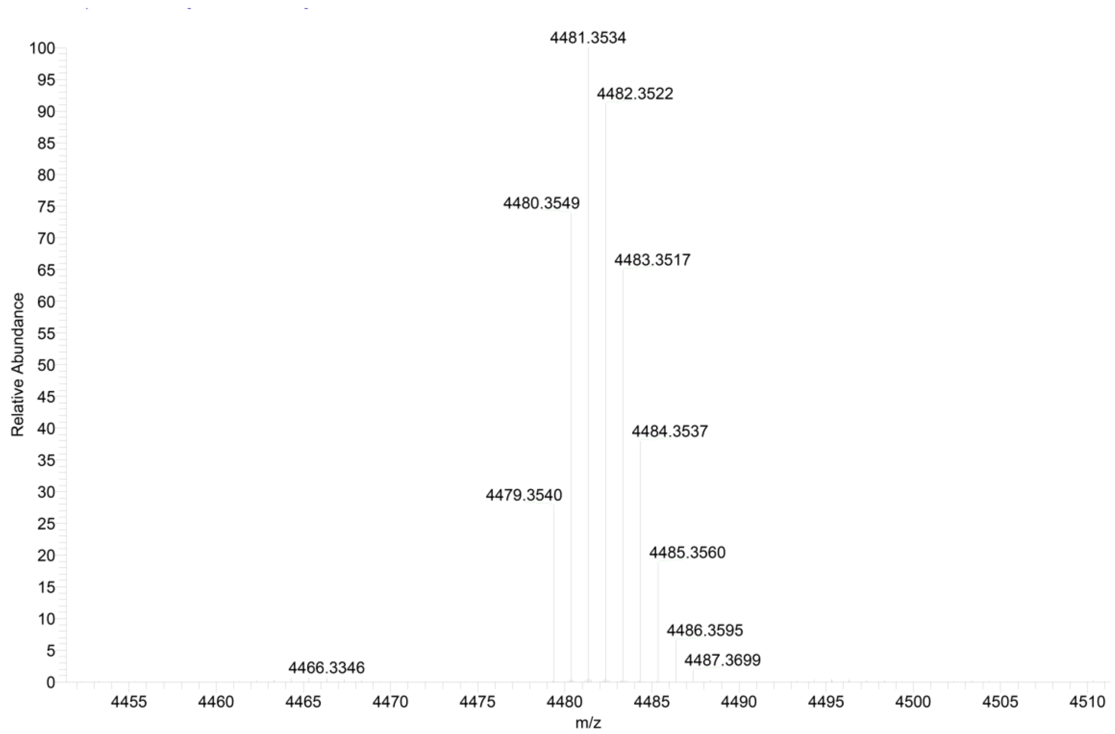

Figure S172. HRMS spectrum.

L-**aX18** ((*Ahx*L)$_8$(KLK)$_4$(KLL)$_2$KLLL) was manually synthesized using TentaGel S RAM resin (393.4 mg, 0.09 mmol, 0.22 mmol·g^{-1}), the dendrimer was obtained as a white foamy solid after preparative RP-HPLC purification (106.3 mg, 22.7%). Analytical RP-HPLC: t_R = 1.68 min (100% A to 100% B in 3.5 min, λ= 214 nm). MS (ESI+): $C_{228}H_{432}N_{50}O_{38}$ calc./obs. 4479.34/4479.35 [M]$^+$.

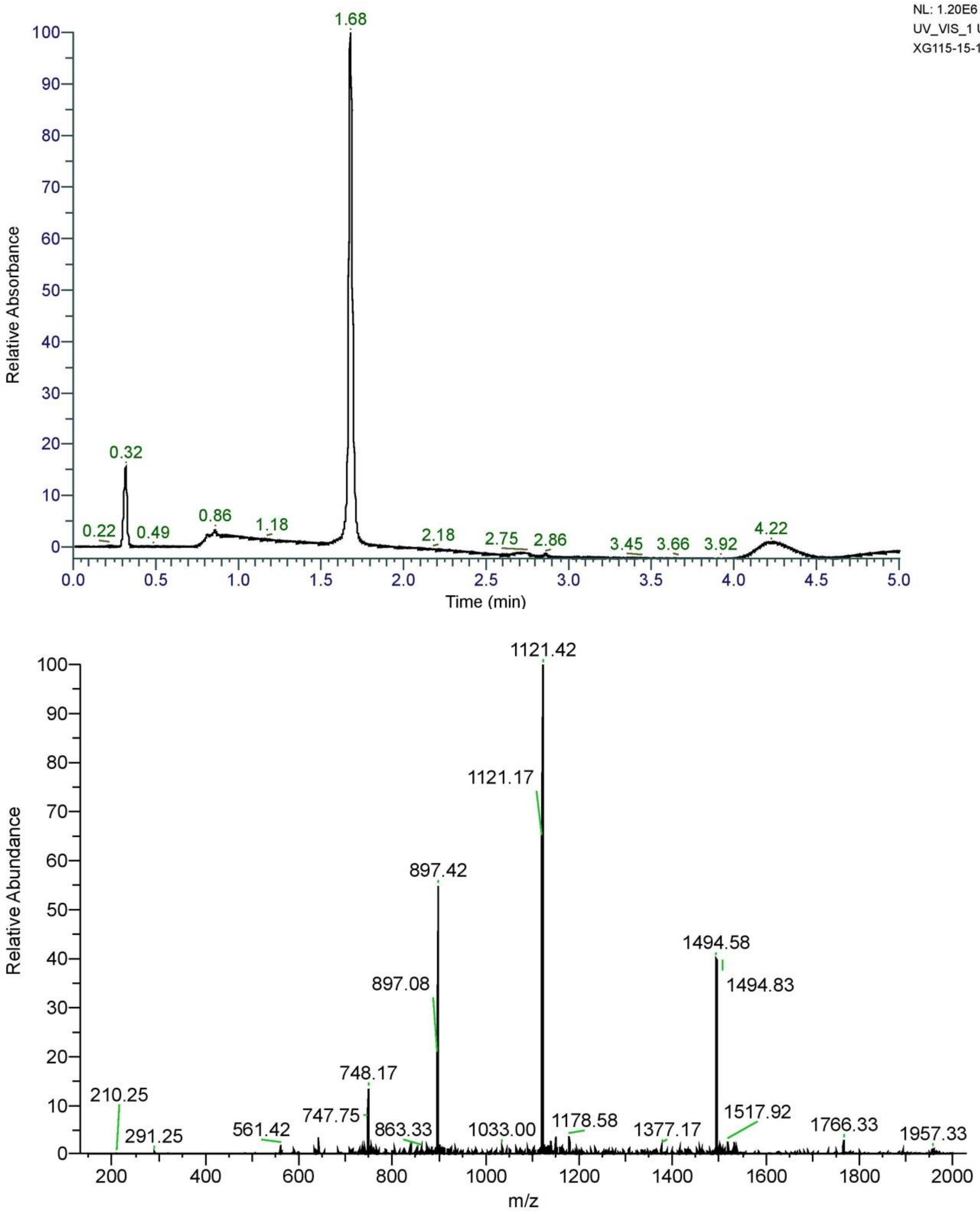

Figure S173. LCMS spectrum.

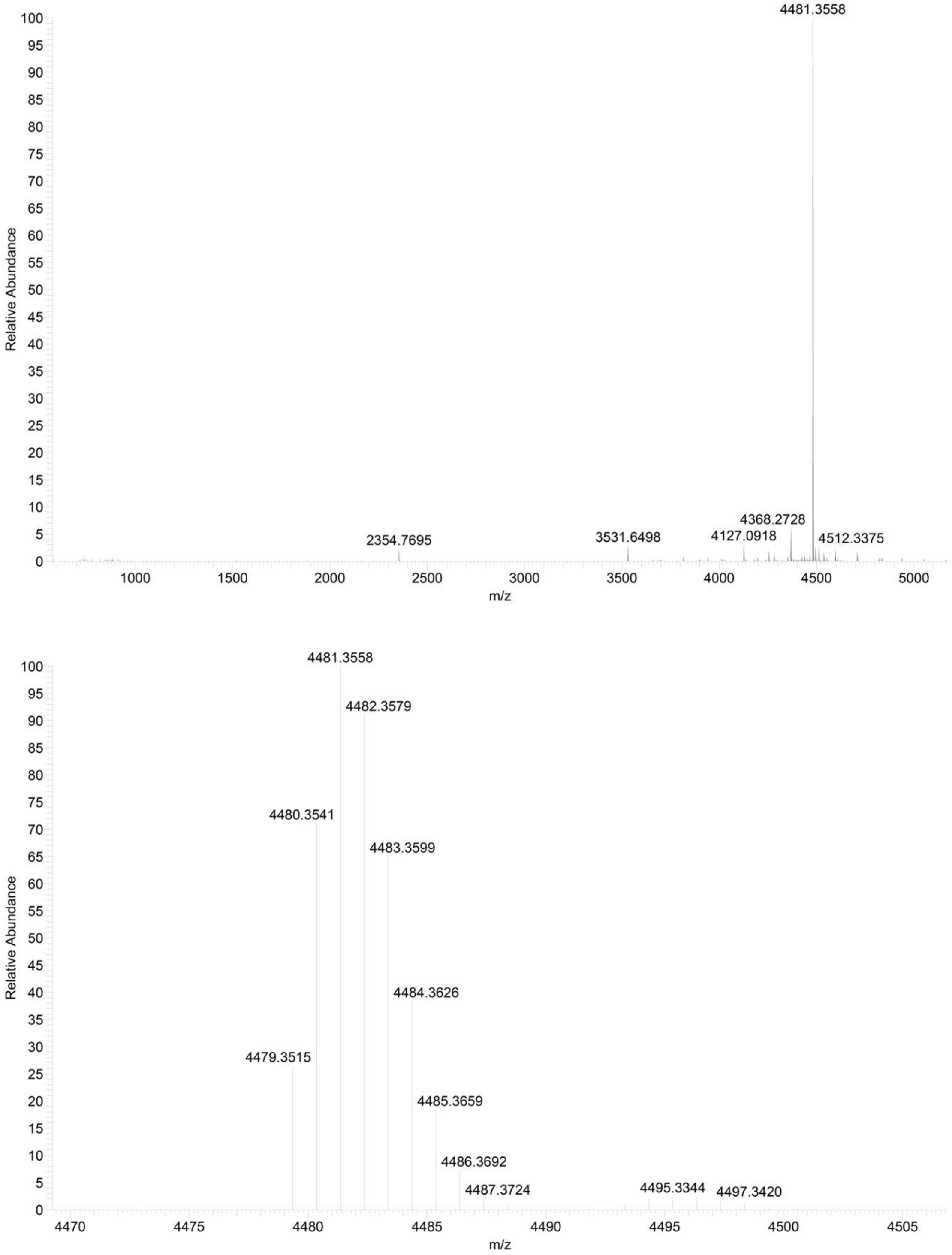

Figure S174. HRMS spectrum.

D-**aX18** ((*Ahx*L)$_8$(KLK)$_4$(KLL)$_2$KLLL) was manually synthesized using TentaGel S RAM resin (393.4 mg, 0.09 mmol, 0.22 mmol·g^{-1}), the dendrimer was obtained as a white foamy solid after preparative RP-HPLC purification (91.2 mg, 19.5%). Analytical RP-HPLC: t_R = 1.68 min (100% A to 100% B in 3.5 min, λ= 214 nm). MS (ESI+): $C_{228}H_{432}N_{50}O_{38}$ calc./obs. 4479.34/4479.36 [M]$^+$.

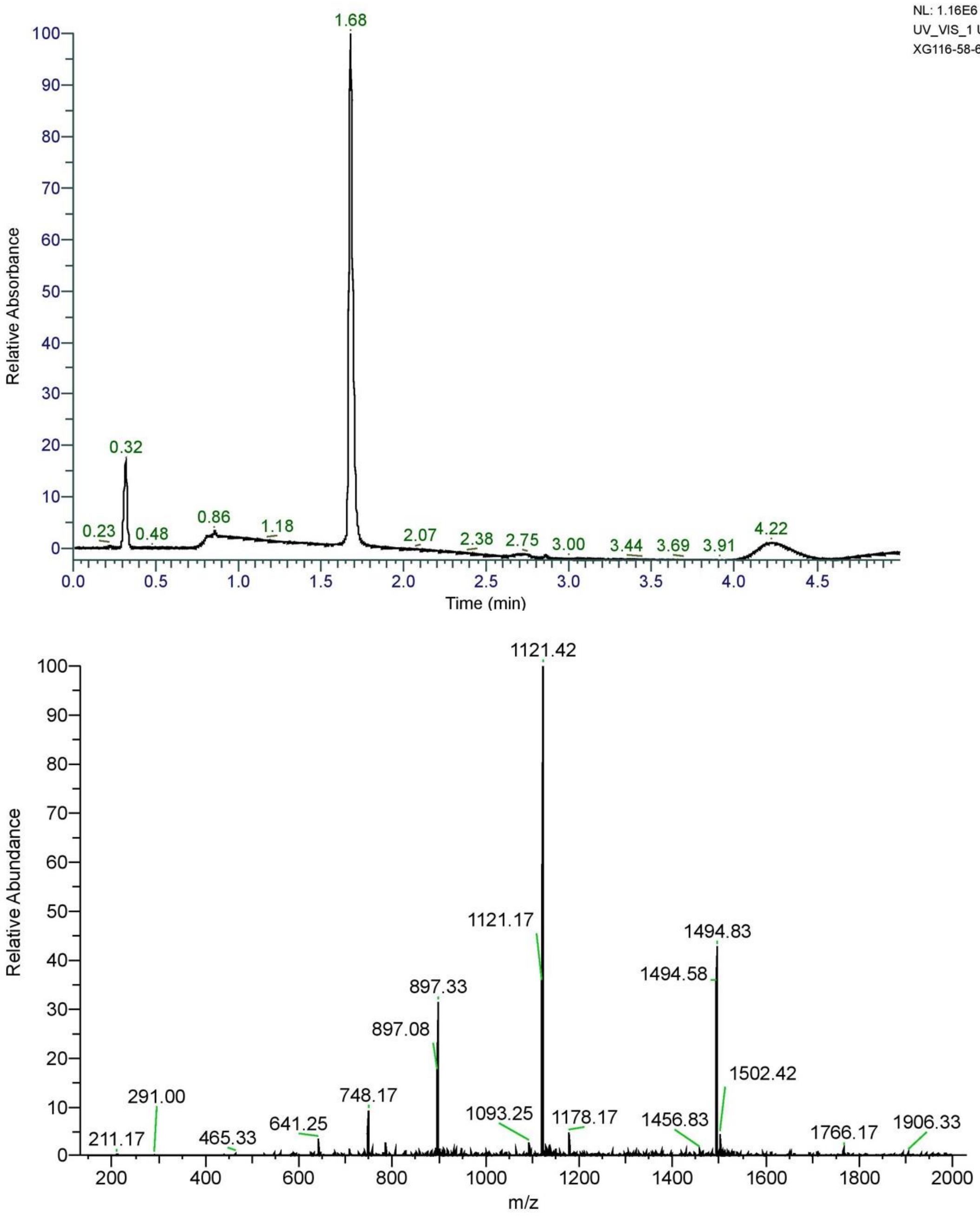

Figure S175. LCMS spectrum.

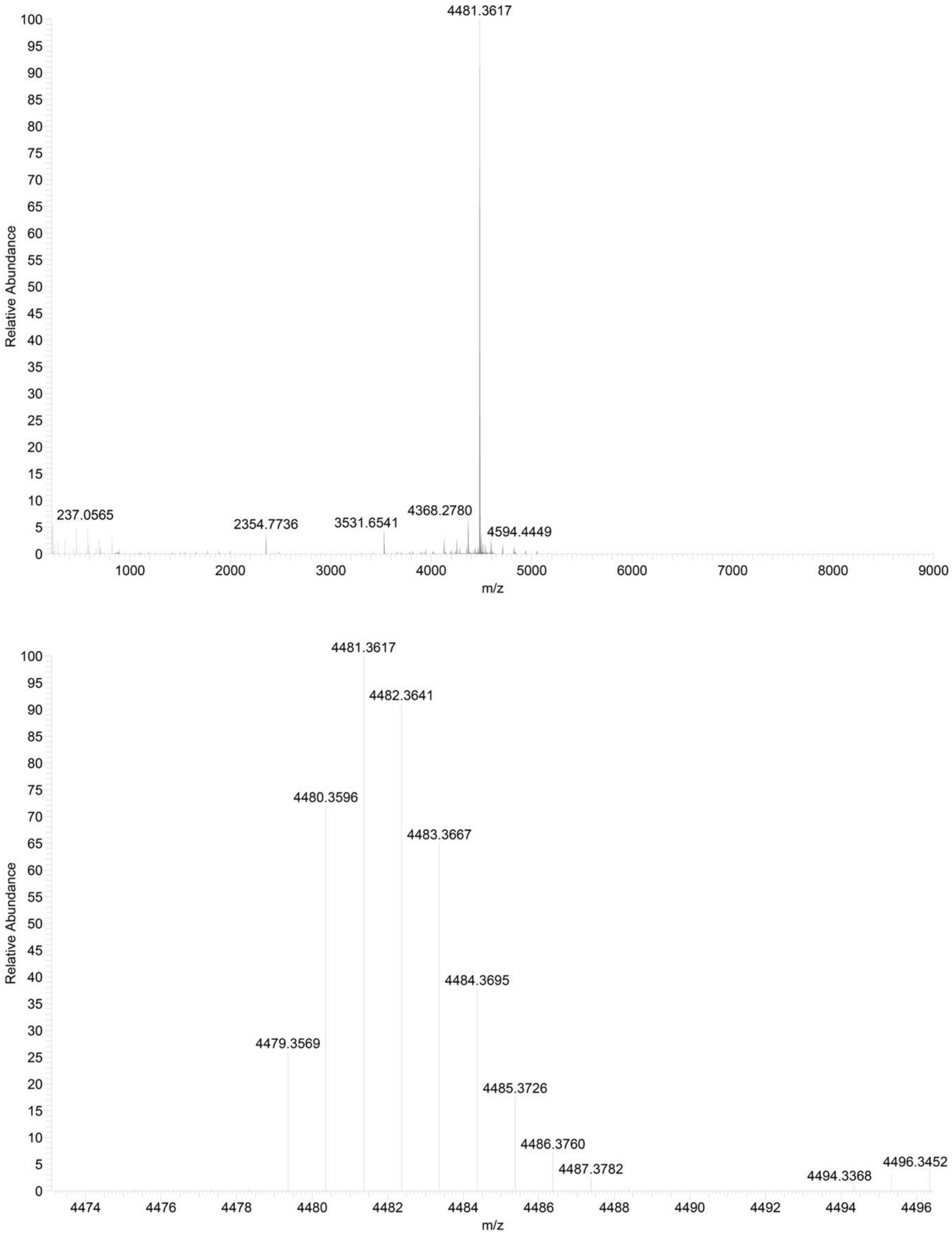

Figure S176. HRMS spectrum.

sr-**aX22** ((*Ahx*-L)$_8$(*K*L)$_4$(*K*KLL)$_2$*K*LKK) was manually synthesized using TentaGel S RAM resin (210.5 mg, 0.08 mmol, 0.38 mmol·g^{-1}), the dendrimer was obtained as a white foamy solid after preparative RP-HPLC purification (141.8 mg, 31.5%). Analytical RP-HPLC: t_R = 1.55 min (100% A to 100% B in 3.5 min, λ= 214 nm). MS (ESI+): $C_{216}H_{410}N_{48}O_{36}$ calc./obs. 4253.17/4253.18 $[M]^+$.

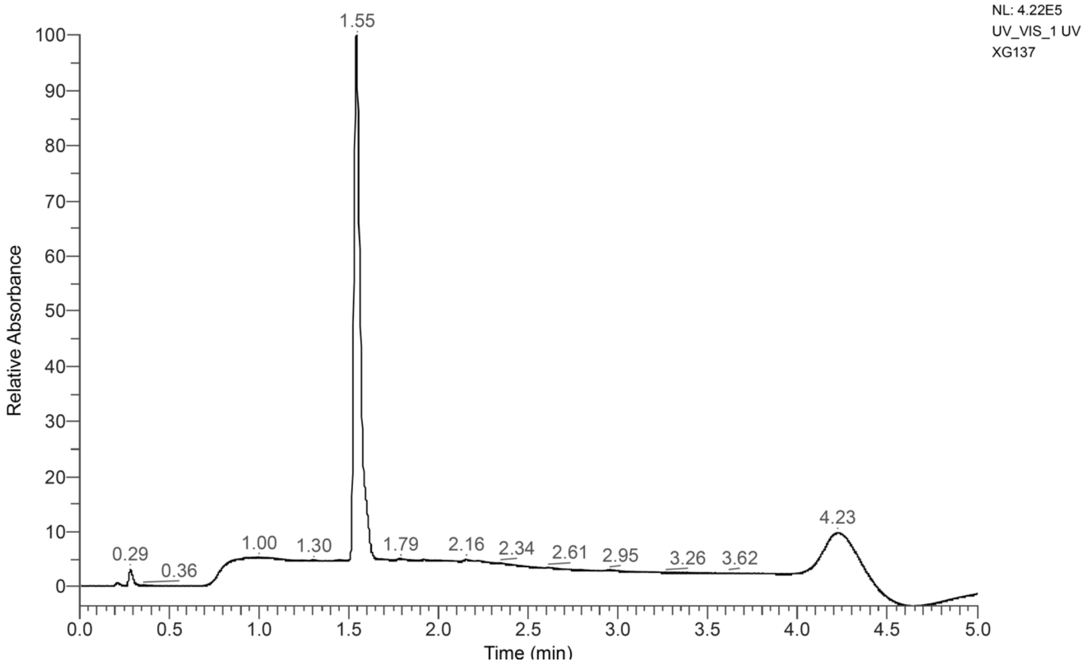

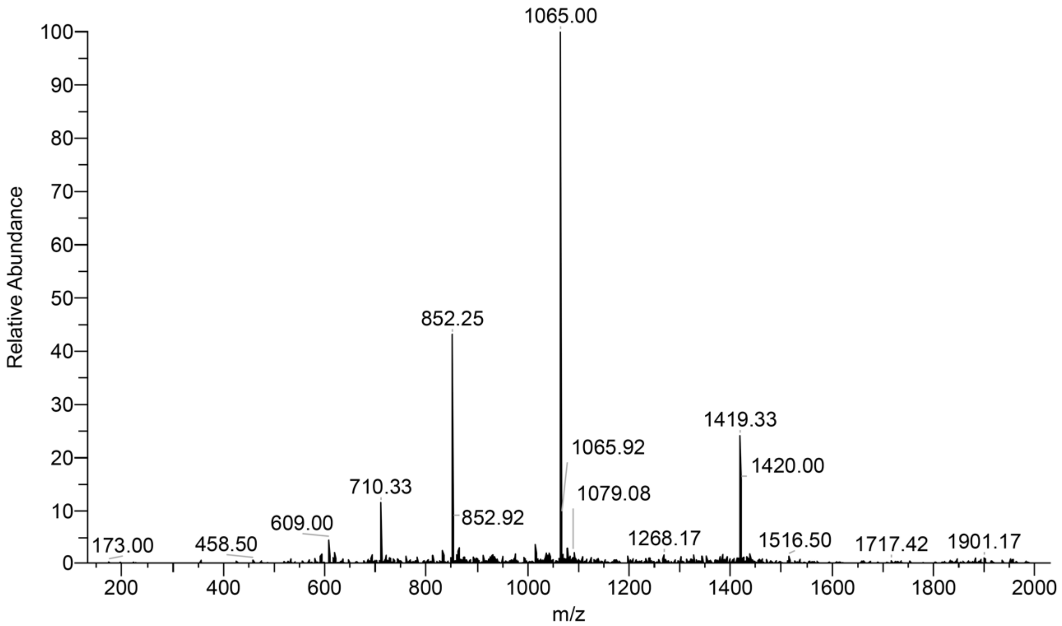

Figure S177. LCMS spectrum.

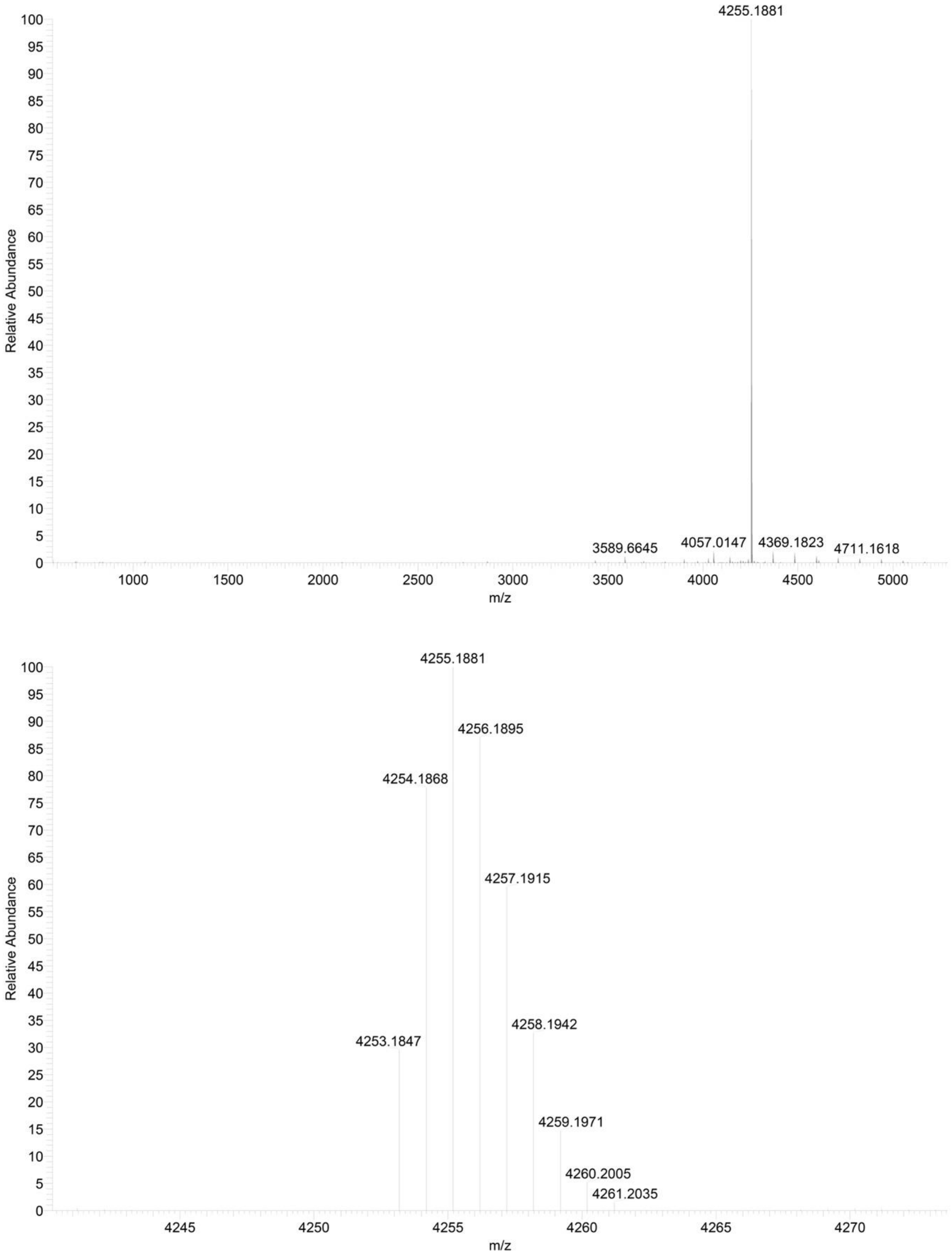

Figure S178. HRMS spectrum.

L-**aX22** ((*Ahx*-L)$_8$(*K*L)$_4$(*K*KLL)$_2$*K*LKK) was manually synthesized using TentaGel S RAM resin (210.5 mg, 0.08 mmol, 0.38 mmol·g^{-1}), the dendrimer was obtained as a white foamy solid after preparative RP-HPLC purification (44.7 mg, 9.9%). Analytical RP-HPLC: t_R = 1.55 min (100% A to 100% B in 3.5 min, λ= 214 nm). MS (ESI+): $C_{216}H_{410}N_{48}O_{36}$ calc./obs. 4253.17/4253.18 [M]$^+$.

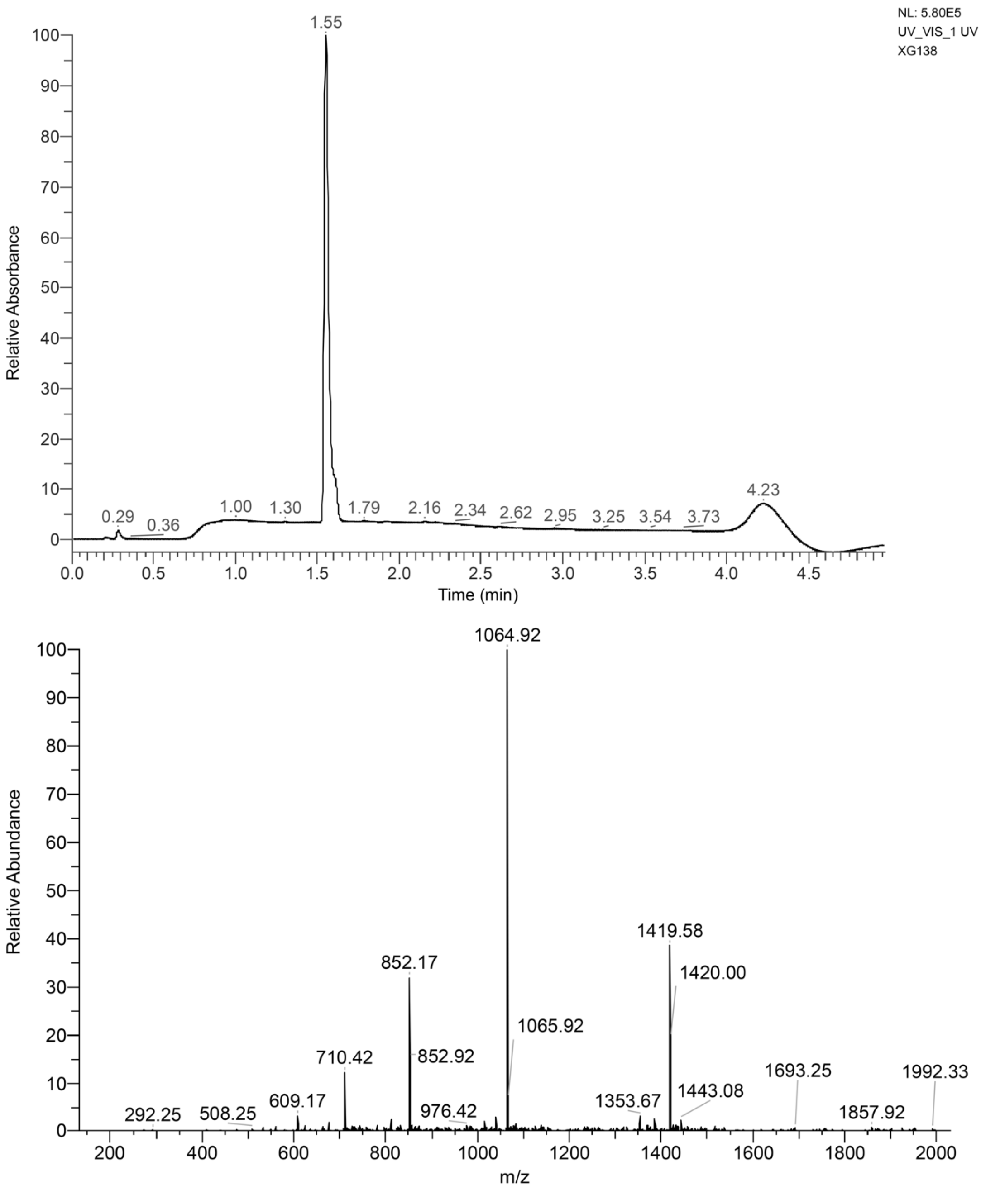

Figure S179. LCMS spectrum.

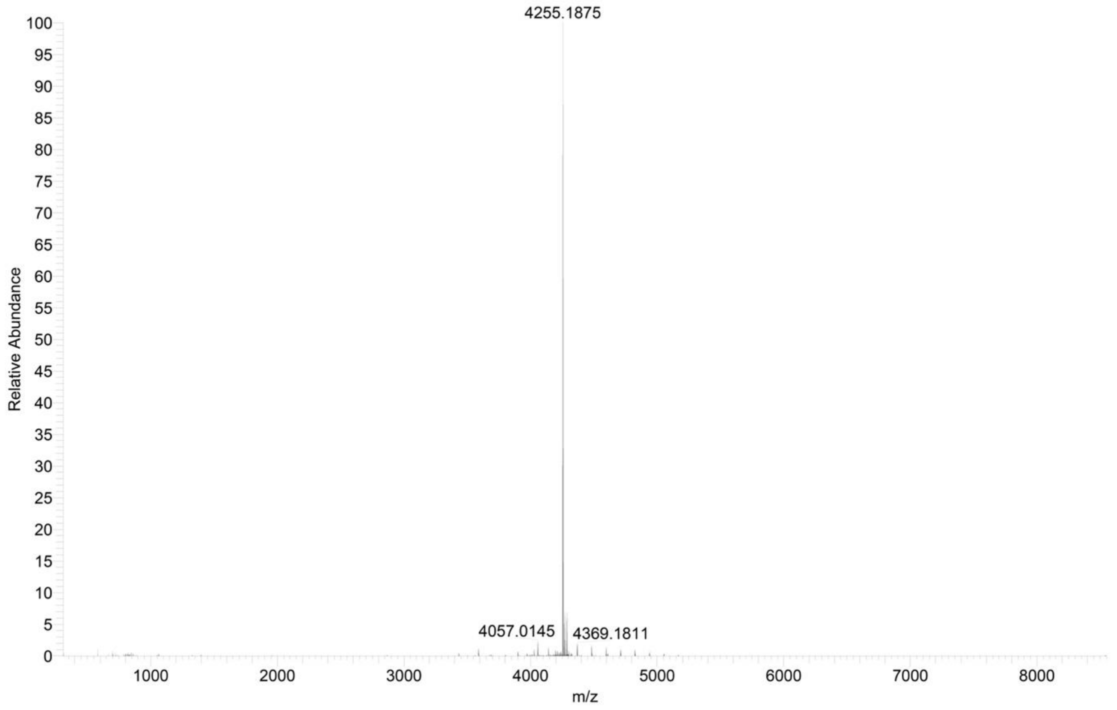

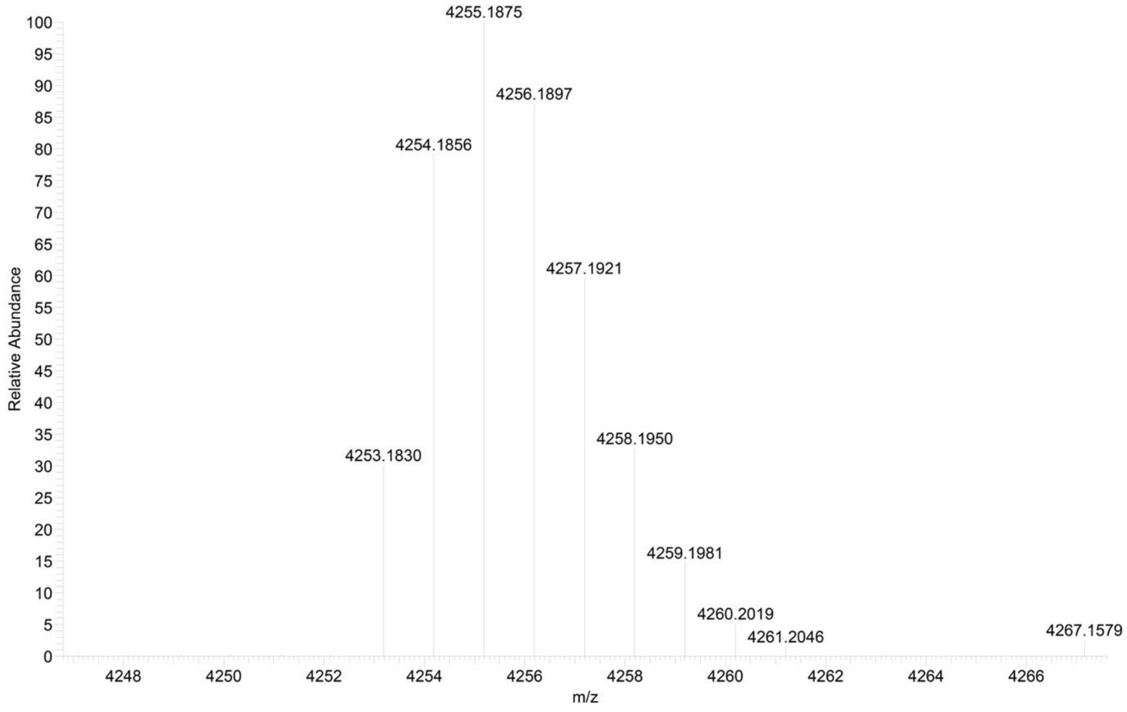

Figure S180. HRMS spectrum.

D-**aX22** ((*Ahx*-L)$_8$(*K*L)$_4$(*K*KLL)$_2$*K*LKK) was manually synthesized using TentaGel S RAM resin (210.5 mg, 0.08 mmol, 0.38 mmol·g^{-1}), the dendrimer was obtained as a white foamy solid after preparative RP-HPLC purification (45.4 mg, 10.1%). Analytical RP-HPLC: t_R = 1.55 min (100% A to 100% B in 3.5 min, λ= 214 nm). MS (ESI+): $C_{216}H_{410}N_{48}O_{36}$ calc./obs. 4253.17/4253.19 [M]$^+$.

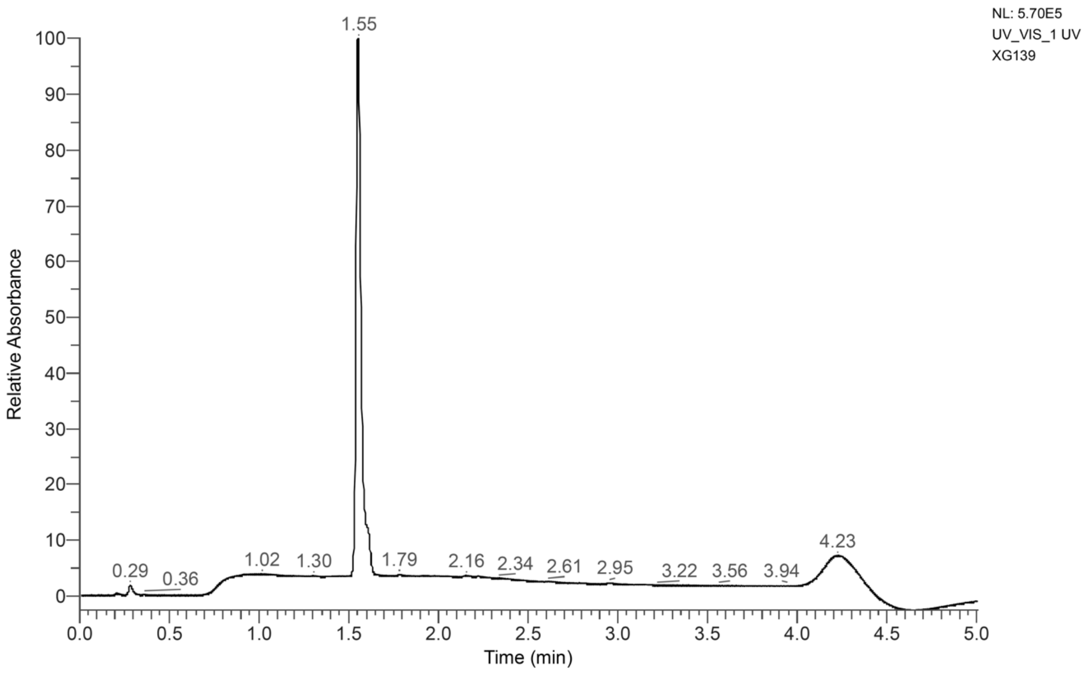

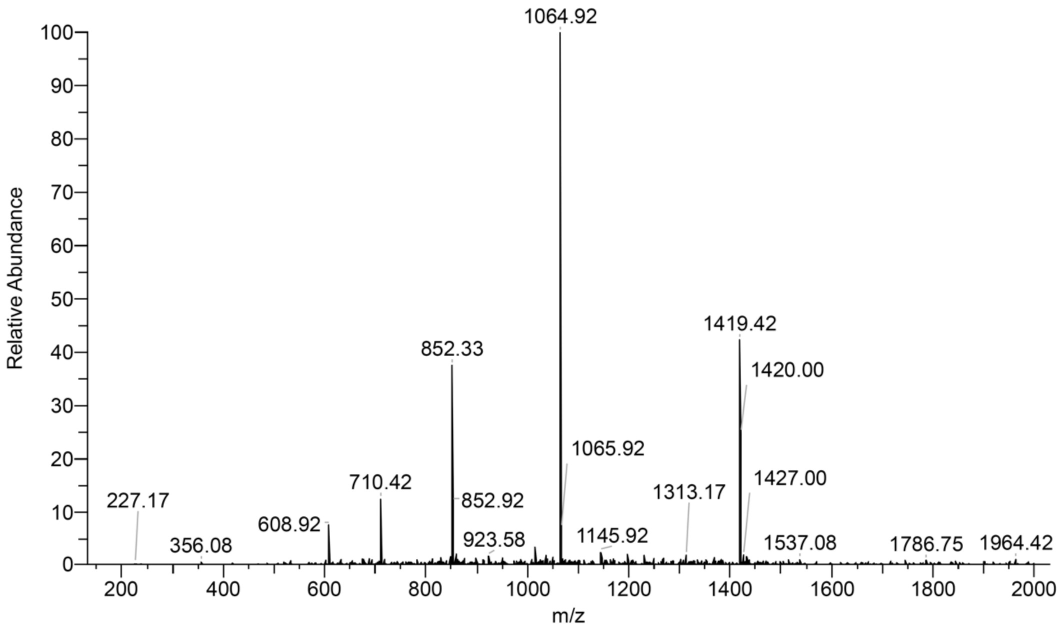

Figure S181. LCMS spectrum.

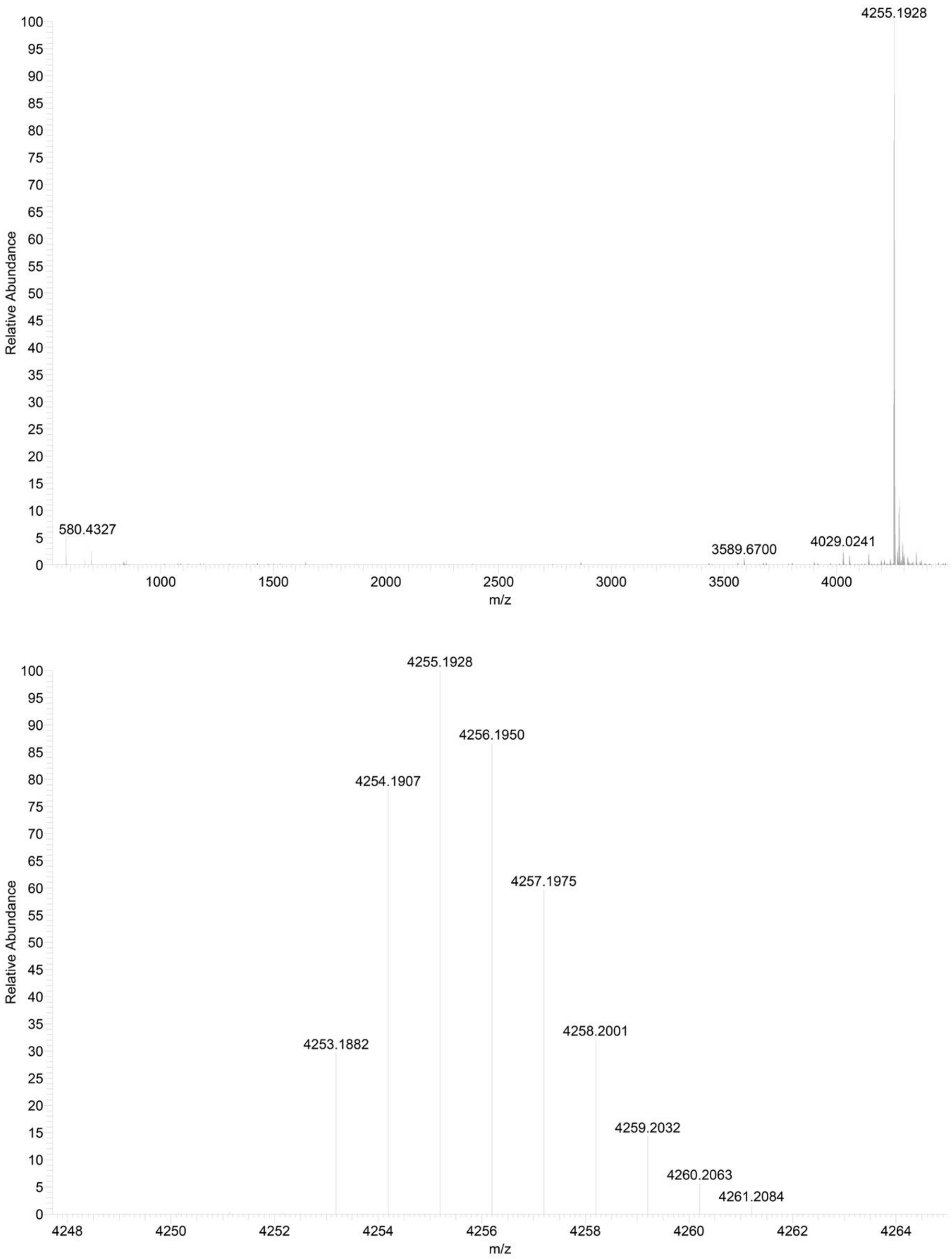

Figure S182. HRMS spectrum.

sr-**T25** ((*K*L)$_8$(*K*KL)$_4$(*K*LL)$_2$*K*KLL) was manually synthesized using TentaGel S RAM resin (345.0 mg, 0.08 mmol, 0.22 mmol·g^{-1}), the dendrimer was obtained as a white foamy solid after preparative RP-HPLC purification (65.4 mg, 12.3%). Analytical RP-HPLC: t_R = 1.42 min (100% A to 100% B in 3.5 min, λ= 214 nm). MS (ESI+): $C_{228}H_{441}N_{59}O_{38}$ calc./obs. 4614.44/4614.44 [M]$^+$.

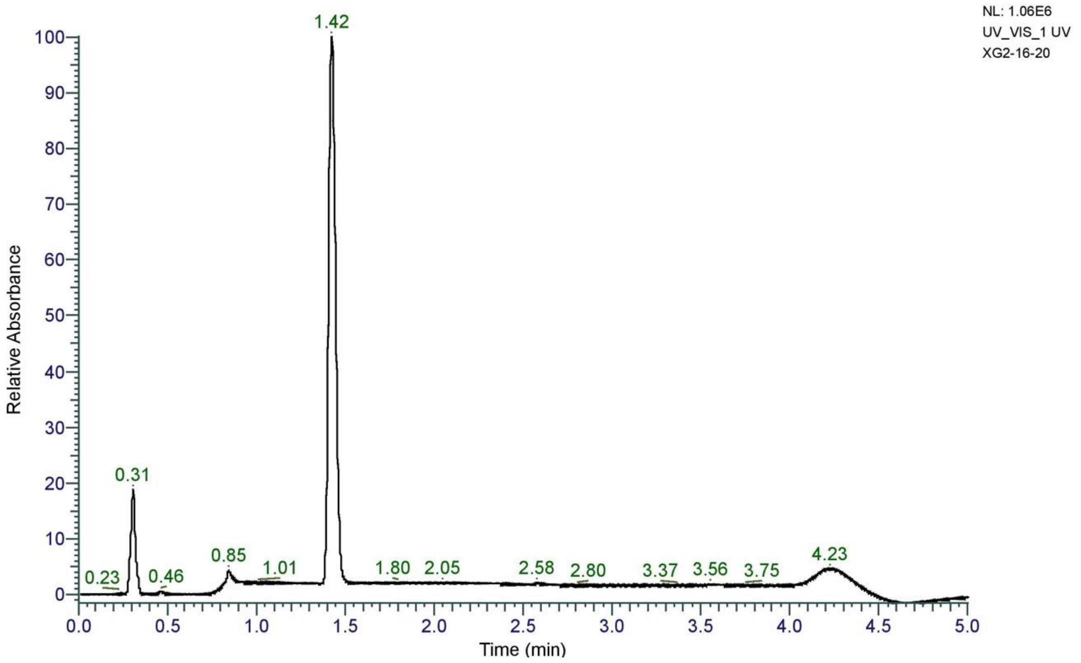

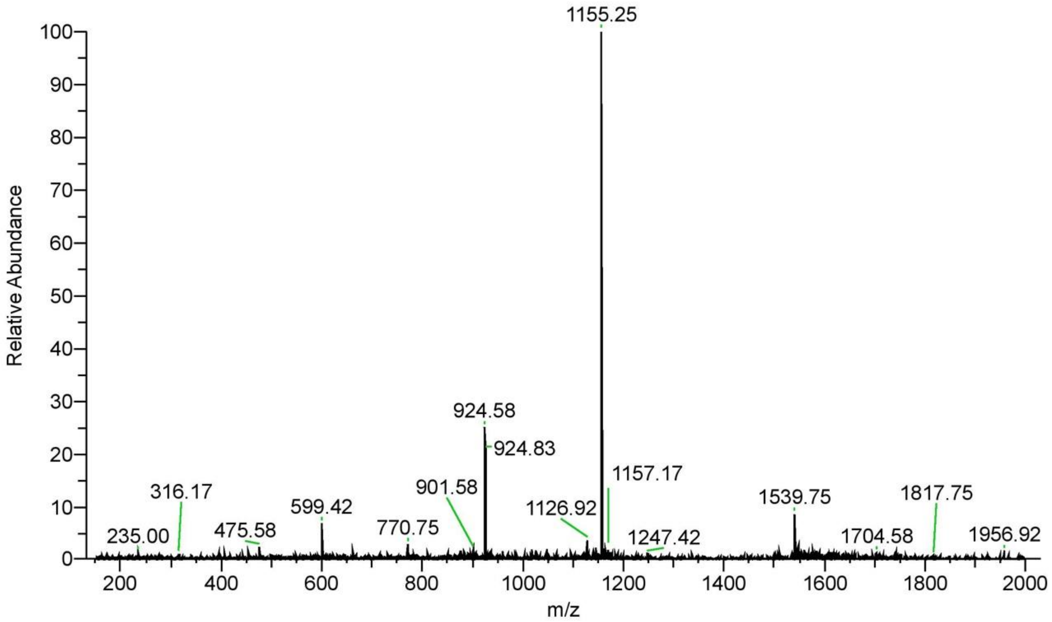

Figure S183. LCMS spectrum.

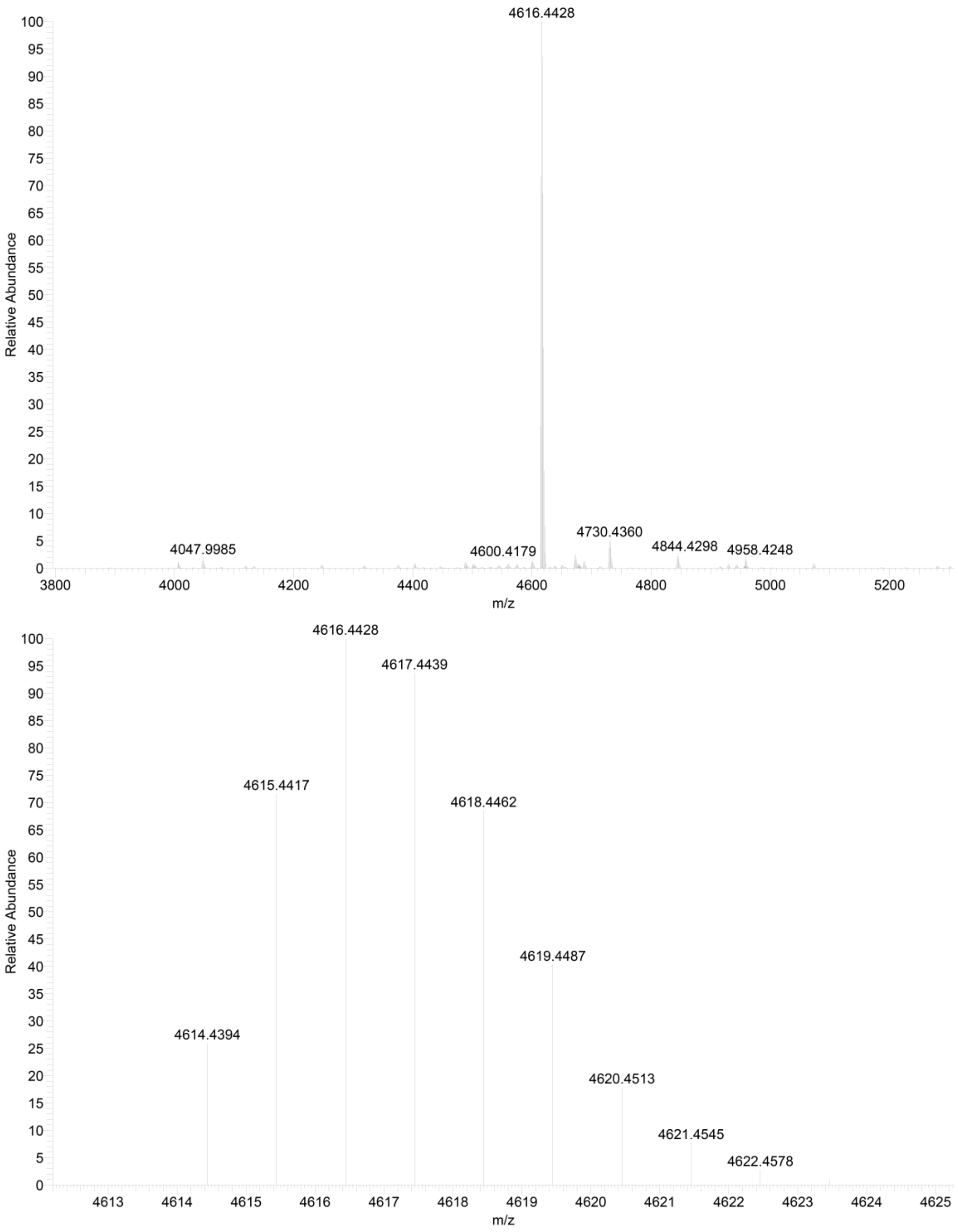

Figure S184. HRMS spectrum.

L-**T25** ((*K*L)$_8$(*K*KL)$_4$(*K*LL)$_2$*K*KLL) was manually synthesized using TentaGel S RAM resin (210.5 mg, 0.08 mmol, 0.38 mmol·g^{-1}), the dendrimer was obtained as a white foamy solid after preparative RP-HPLC purification (40.3 mg, 7.2%). Analytical RP-HPLC: t_R = 1.41 min (100% A to 100% B in 3.5 min, λ= 214 nm). MS (ESI+): $C_{228}H_{441}N_{59}O_{38}$ calc./obs. 4614.44/4614.43 [M]$^+$.

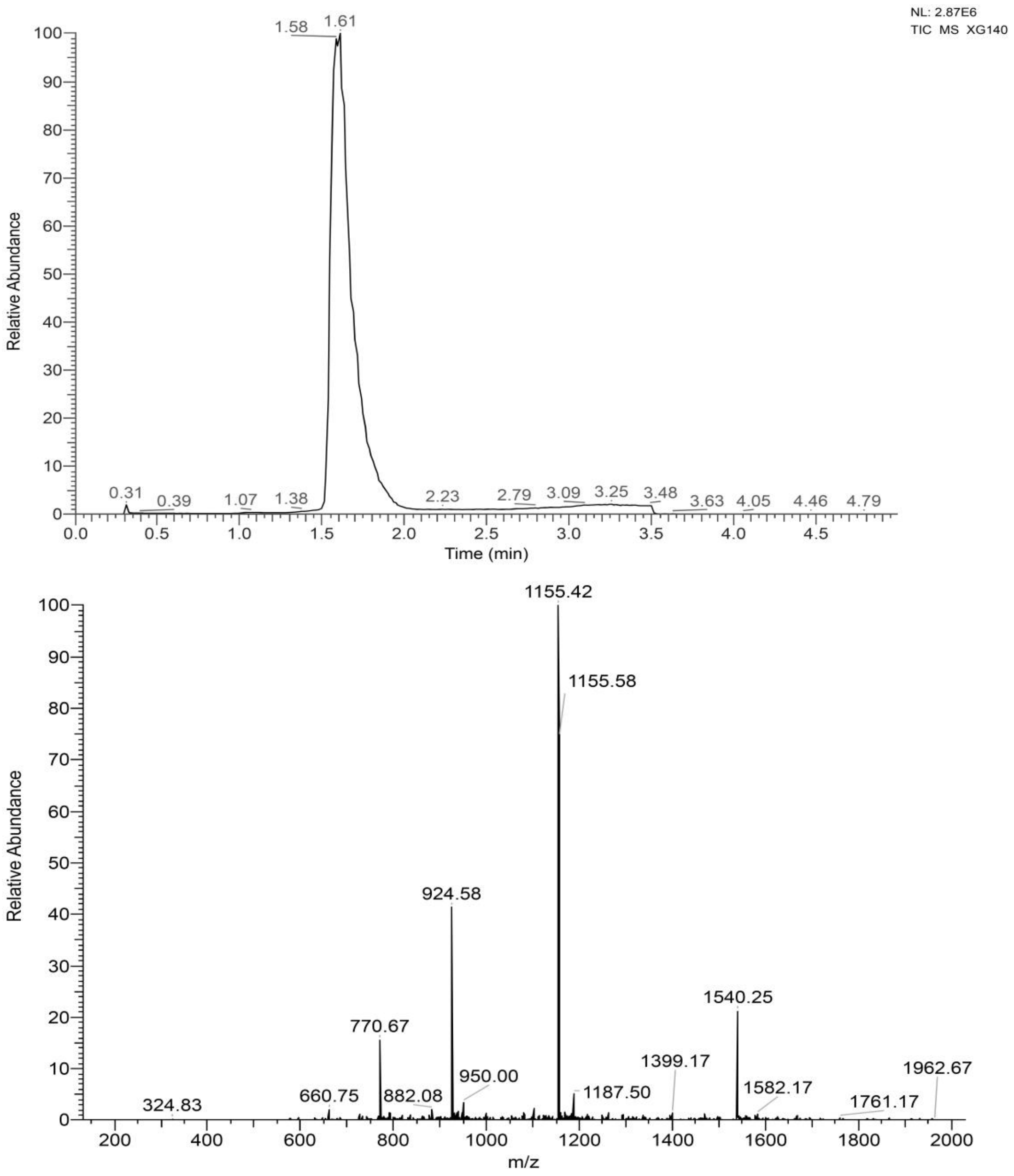

Figure S185. LCMS spectrum.

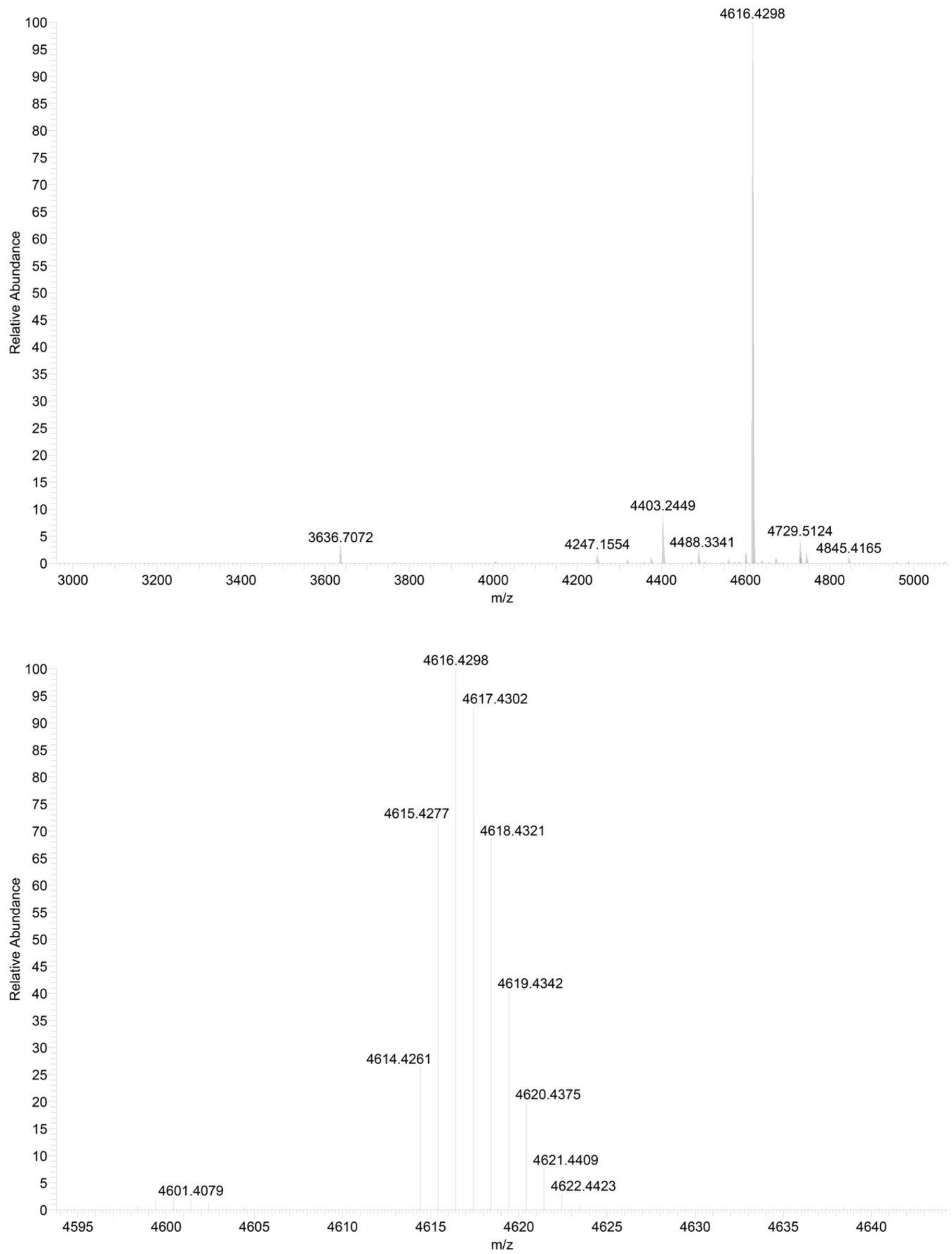

Figure S186. HRMS spectrum.

D-**T25** ((*K*L)$_8$(*K*KL)$_4$(*K*LL)$_2$*K*KLL) was manually synthesized using TentaGel S RAM resin (210.5 mg, 0.08 mmol, 0.38 mmol·g^{-1}), the dendrimer was obtained as a white foamy solid after preparative RP-HPLC purification (85.5 mg, 15.3%). Analytical RP-HPLC: t_R = 1.41 min (100% A to 100% B in 3.5 min, λ= 214 nm). MS (ESI+): $C_{228}H_{441}N_{59}O_{38}$ calc./obs. 4614.44/4614.43 [M]$^+$.

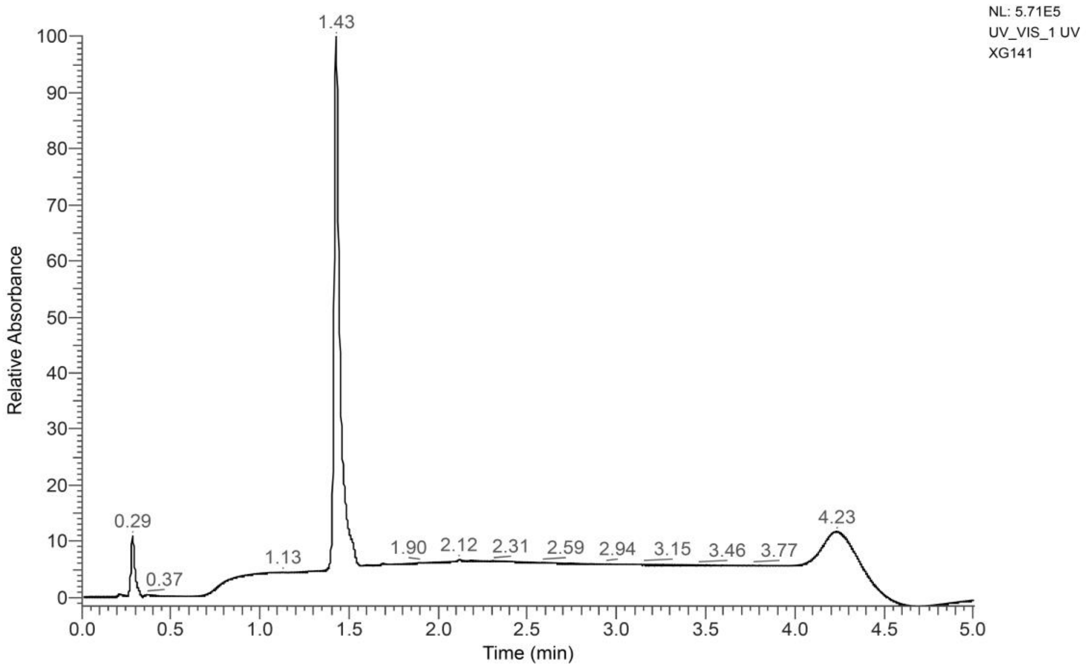

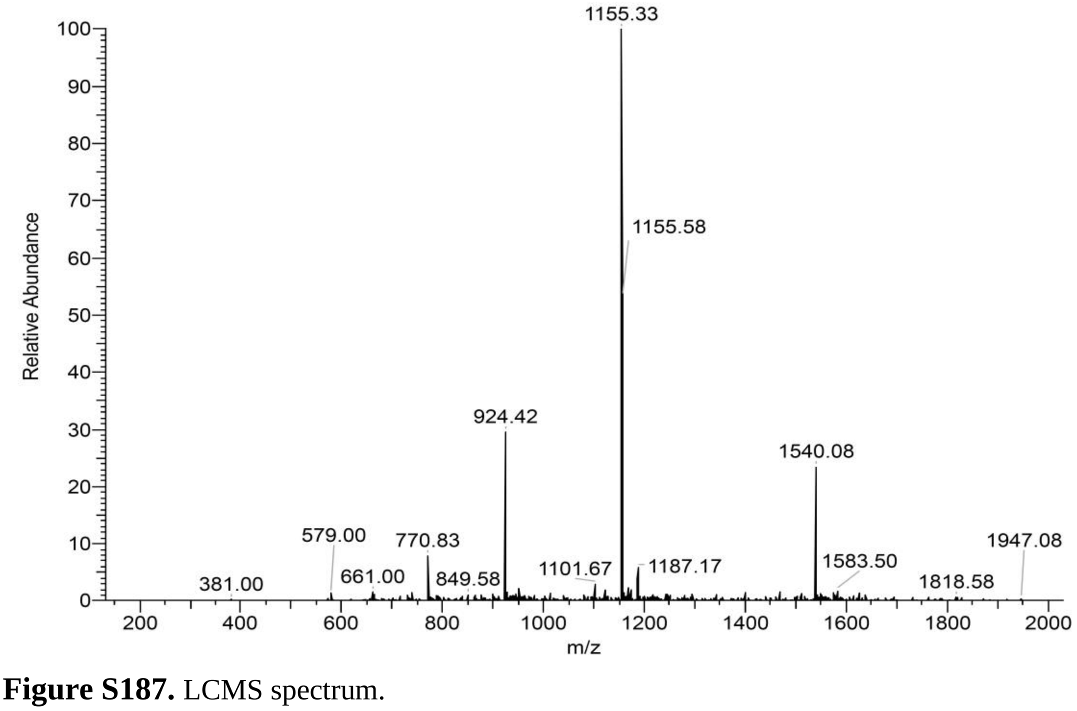

Figure S187. LCMS spectrum.

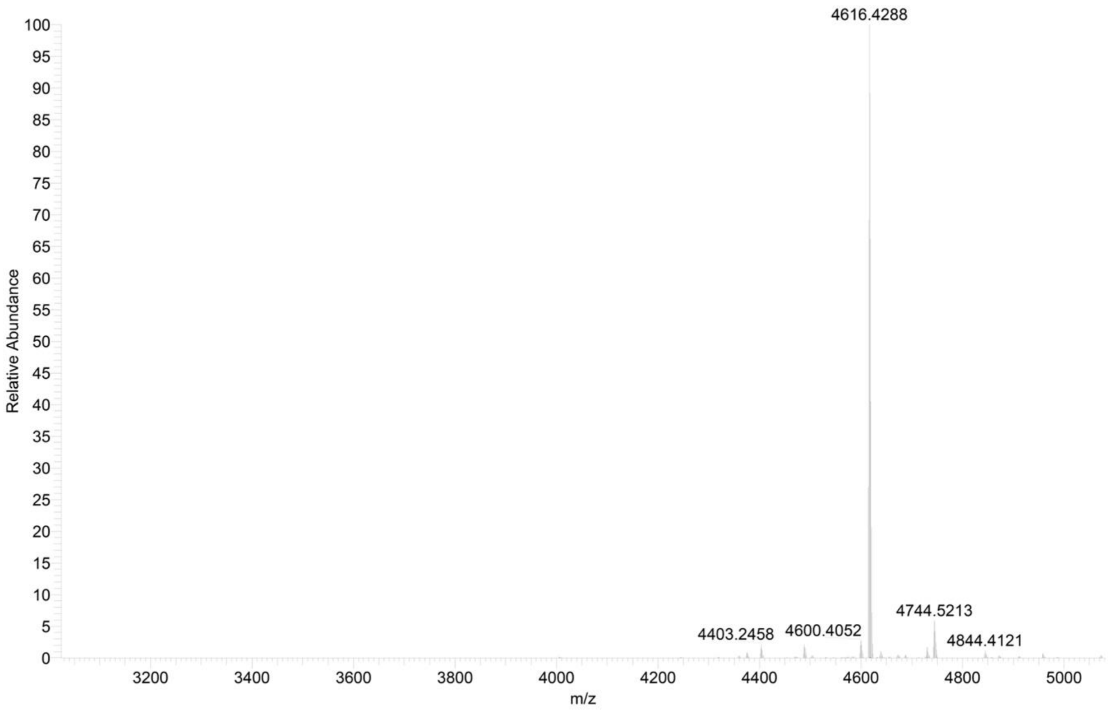

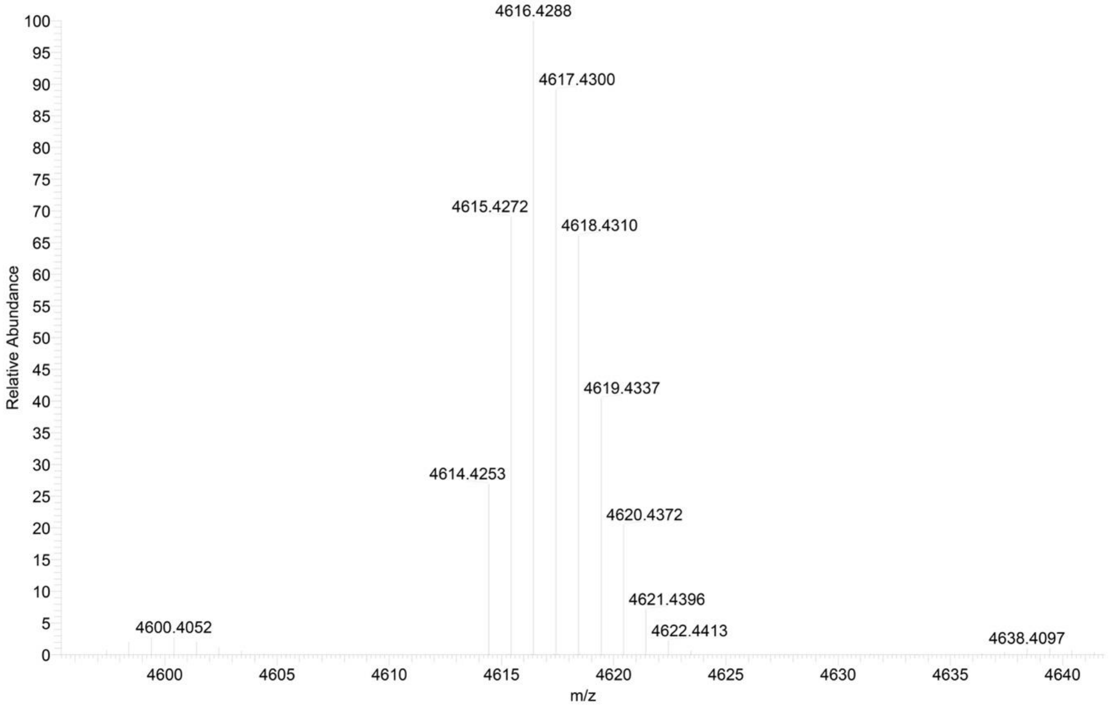

Figure S188. HRMS spectrum.

sr-**aT25** ((*Ahx*-L)$_8$(*K*KL)$_4$(*K*LL)$_2$*K*KLL) was manually synthesized using TentaGel S RAM resin (210.5 mg, 0.08 mmol, 0.38 mmol·g^{-1}), the dendrimer was obtained as a white foamy solid after preparative RP-HPLC purification (129.1 mg, 35.9%). Analytical RP-HPLC: t_R = 1.70 min (100% A to 100% B in 3.5 min, λ= 214 nm). MS (ESI+): $C_{228}H_{443}N_{51}O_{38}$ calc./obs. 4494.35/4494.35 $[M]^+$.

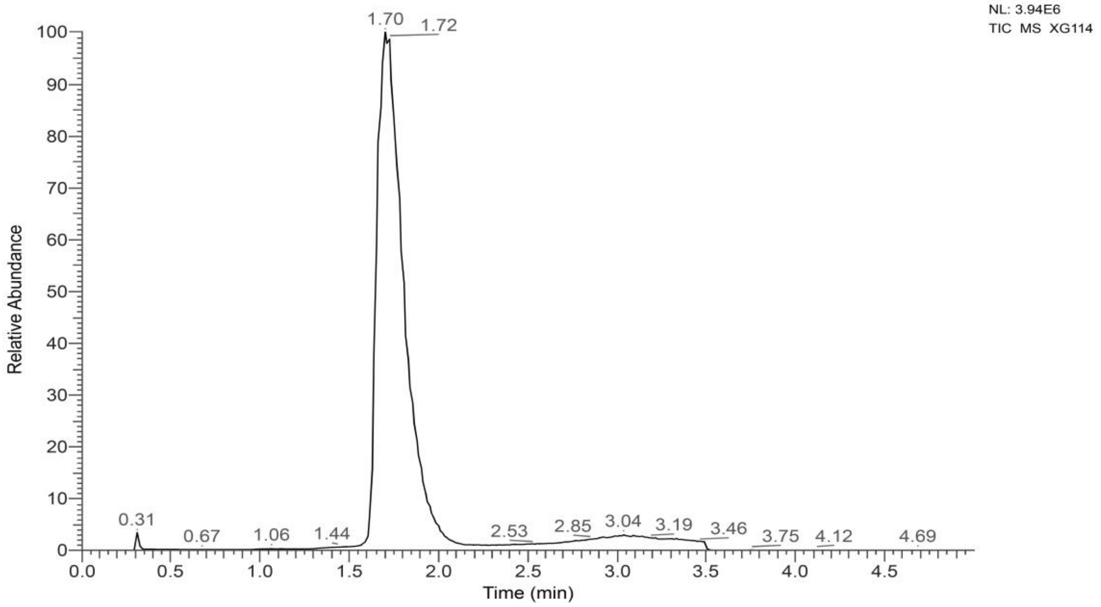

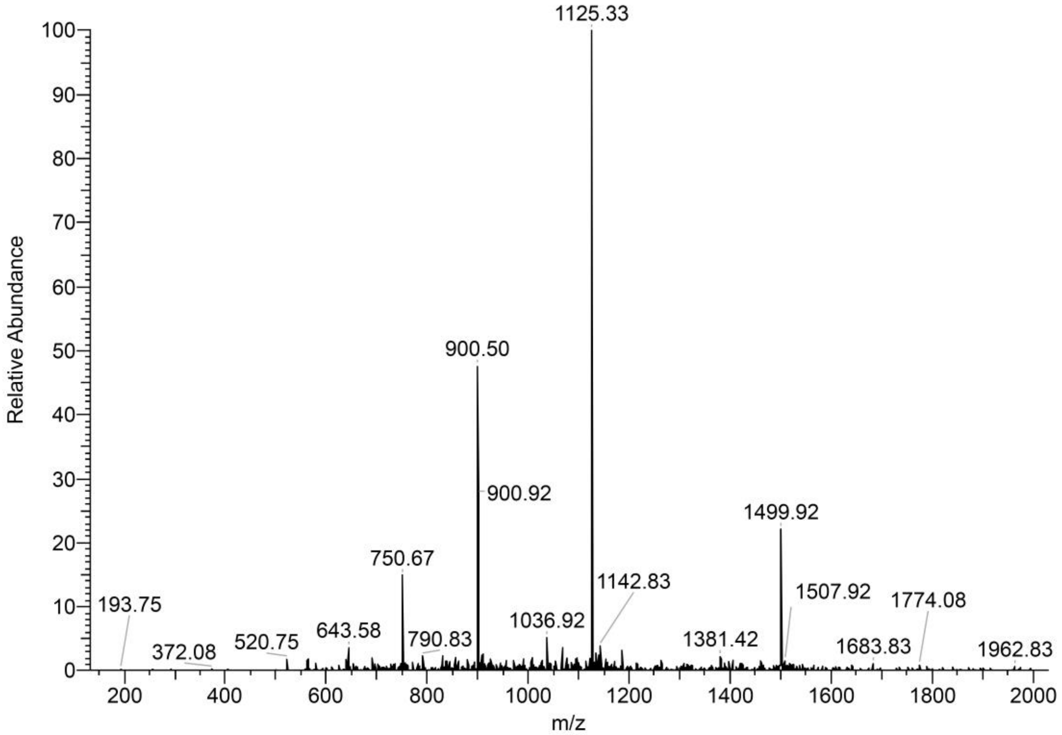

Figure S189. LCMS spectrum.

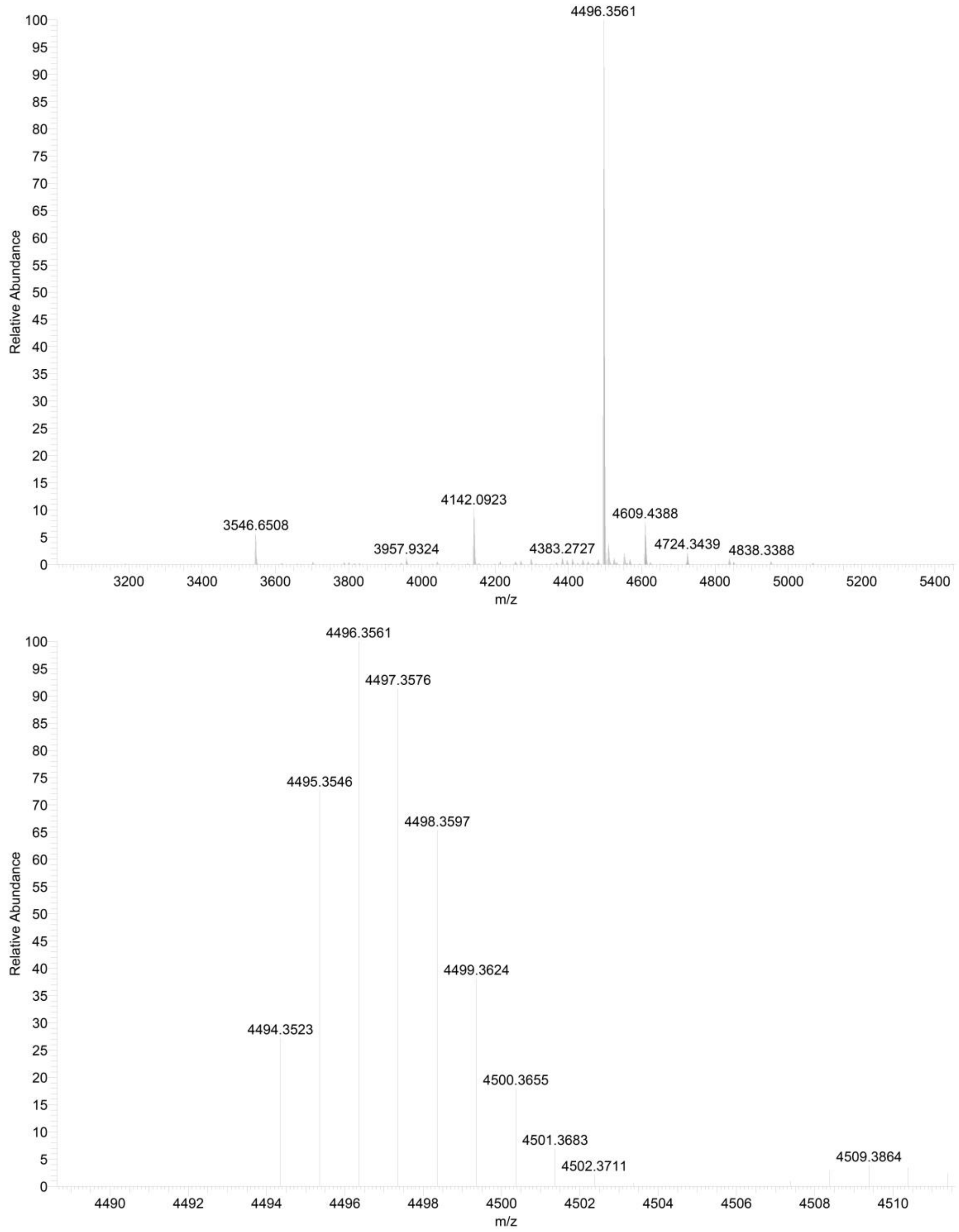

Figure S190. LCMS spectrum.
